# International Expert-Based Consensus Definition, Staging Criteria, and Minimum Data Elements for Osteoradionecrosis of the Jaw: An Inter-Disciplinary Modified Delphi Study

**DOI:** 10.1101/2024.04.07.24305400

**Published:** 2024-04-09

**Authors:** The International ORAL Consortium, Amy C. Moreno, Erin E. Watson, Laia Humbert-Vidan, Douglas E. Peterson, Lisanne V van Dijk, Teresa Guerrero Urbano, Lisa Van den Bosch, Andrew J. Hope, Matthew S. Katz, Frank J.P. Hoebers, Ruth A. Aponte Wesson, James E. Bates, Paolo Bossi, Adeyinka F. Dayo, Mélanie Doré, Eduardo Rodrigues Fregnani, Thomas J. Galloway, Daphna Y. Gelblum, Issa A. Hanna, Christina E. Henson, Sudarat Kiat-amnuay, Anke Korfage, Nancy Y. Lee, Carol M. Lewis, Charlotte Duch Lynggaard, Antti A. Mäkitie, Marco Magalhaes, Yvonne M. Mowery, Carles Muñoz-Montplet, Jeffrey N. Myers, Ester Orlandi, Jaymit Patel, Jillian M. Rigert, Deborah Saunders, Jonathan D. Schoenfeld, Ugur Selek, Efsun Somay, Vinita Takiar, Juliette Thariat, Gerda M. Verduijn, Alessandro Villa, Nick West, Max J.H. Witjes, Alex Won, Mark E. Wong, Christopher M.K.L. Yao, Simon W. Young, Kamal Al-eryani, Carly E.A. Barbon, Doke J.M. Buurman, François J. Dieleman, Theresa M. Hofstede, Abdul Ahad Khan, Adegbenga O. Otun, John C. Robinson, Lauren Hum, Jorgen Johansen, Rajesh Lalla, Alexander Lin, Vinod Patel, Richard J. Shaw, Mark S. Chambers, Daniel Ma, Mabi Singh, Noam Yarom, Abdallah Sherif Radwan Mohamed, Katherine A. Hutcheson, Stephen Y. Lai, Clifton David Fuller

**Affiliations:** 1- Department of Radiation Oncology, The University of Texas MD Anderson Cancer Center, Houston, USA; 2- Department of Dental Oncology/Faculty of Dentistry, Princess Margaret Cancer Centre; University Health Network; University of Toronto, Toronto, CA; 3- Oral Health and Diagnostic Sciences, University of Connecticut, Farmington, USA; 4- Department of Radiation Oncology, University Medical Center Groningen, Groningen, NL; 5- Oncology Department, Guy’s and St Thomas NHS Trust/King’s College London, London, UK; 6- Department of Radiation Oncology, Princess Margaret Cancer Centre; University Health Network; University of Toronto, Toronto, CA; 7- Radiation Oncology, Lowell General Hospital, Lowell, USA; 8- Department of Radiation Oncology, GROW School for Oncology and Reproduction, Maastricht University Medical Centre, Maastricht, NL; 9- Department of Head and Neck Surgery, The University of Texas MD Anderson Cancer Center, Houston, USA; 10- Department of Radiation Oncology, Winship Cancer Institute/Emory University, Atlanta, USA; 11- Medical Oncology, Department of Medical and Surgical Specialties, Humanitas University; IRCCS Humanitas Research Hospital, Milan, IT; 12- Oral Medicine Department, University of Pennsylvania School of Dental Medicine, Philadelphia, USA; 13- Radiotherapy Department, Institut de Cancérologie de l’Ouest, Saint Herblain, FR; 14- Centro de Oncologia Molecular, Hospital Sírio-Libanês, São Paulo, BR; 15- Department of Radiation Oncology, Fox Chase Cancer Center, Philadelphia, USA; 16- Department of Radiation Oncology, Memorial Sloan Kettering Cancer Center, New York, USA; 17- Katz Department of Oral and Maxillofacial Surgery, UTHealth Houston School of Dentistry, Houston, USA; 18- Department of Radiation Oncology, University of Oklahoma Health Sciences Center, Oklahoma City, USA; 19- Department of General Practice and Dental Public Health, UTHealth Houston School of Dentistry, Houston, USA; 20- Department of Oral and Maxillofacial Surgery, University Medical Center Groningen, University of Groningen, Groningen, NL; 21- Department of Otorhinolaryngology, Head and Neck Surgery & Audiology, Rigshospitalet, Copenhagen University Hospital, Copenhagen, DK; 22- Department of Otorhinolaryngology - Head and Neck Surgery, University of Helsinki and Helsinki University Hospital, Helsinki, FI; 23- Faculty of Dentistry, Oral Pathology and Oral Medicine, faculty of Dentistry, University of Toronto, Toronto, CA; 24- Department of Radiation Oncology, UPMC Hillman Cancer Center/University of Pittsburgh, Pittsburgh, USA; 25- Medical Physics and Radiation Protection, Catalan Institute of Oncology-Girona, Girona, ES; 26- Department of Head & Neck Surgery, The University of Texas MD Anderson Cancer Center, Houston, USA; 27- Department of Clinical, Surgical, Diagnostic, and Pediatric Sciences, University of Pavia/Clinical Department, National Center for Oncological Hadrontherapy (Fondazione CNAO), Pavia, IT; 28- Department of Restorative Dentistry, Leeds Teaching Hospitals Trust, Leeds, UK; 29- Department Dental Oncology, Health Sciences North, Northern Ontario School of Medicine University, Sudbury, CA; 30- Department of Radiation Oncology, Dana Farber-Cancer Institute and Brigham and Women’s Hospital, Boston, USA; 31- Department of Radiation Oncology, School of Medicine, Koc University, Istanbul, TR; 32- Department of Oral And Maxillofacial Surgery, Baskent University, Ankara, TR; 33- Department of Radiation Oncology, University of Cincinnati College of Medicine, Cincinnati, USA; 34- Department of Radiation Oncology, Comprehensive Cancer Centre François Baclesse, Caen, FR; 35- Department of Radiation Oncology, Erasmus Medical Center, Rotterdam, NL; 36- Oral Medicine, Oral Oncology, Miami Cancer Institute, Baptist Heath South Florida, Miami, USA; 37- Northern Centre for Cancer Care, Northern Centre for Cancer Care, Newcastle upon Tyne Hospitals Trust, Newcastle, UK; 38- Oral & Maxillofacial Surgery, University Medical Center Groningen, Groningen, NL; 39- Department of Otolaryngology Head and Neck Surgery, University Health Network, Princess Margaret Cancer Center/University Health Network, Toronto, CA; 40- Oral Medicine, Oral Pathology, and Oral Radiology, University of California, San Francisco, San Francisco, USA; 41- Department of Cranio-Maxillofacial Surgery, Maastricht University Medical Centre, Maastricht, NL; 42- Department of Oral and Maxillofacial Surgery and Head and Neck Surgical Oncology, University Medical Center Utrecht Cancer Center, Utrecht, NL; 43- Department of Oral and Maxillofacial Surgery, Odense University Hospital, Odense, DK; 44- Orofacial Sciences, University of California, San Francisco, San Francisco, USA; 45- Department of Oncology, Odense University Hospital, Odense, DK; 46- Oral and Maxillofacial Diagnostics Sciences, University of Connecticut, Farmington, USA; 47- Department of Radiation Oncology, University of Pennsylvania, Philadelphia, USA; 48- Oral Surgery, Guy’s and St Thomas NHS Trust, London, UK; 49- Head & Neck Surgery, Molecular & Clinical Cancer Medicine, The University of Liverpool Cancer Research Centre/Aintree University Hospitals NHS Foundation Trust, Liverpool, UK; 50- Department of Radiation Oncology, Mayo Clinic, Rochester, USA; 51- Division of Oral Medicine, Department of Diagnostic Sciences, Tufts University School of Dental Medicine, Boston, USA; 52- Department of Oral Medicine, Sheba Medical Center, Tel-Hashomer; School of Dental Medicine, Tel-Aviv University, Tel-Aviv, IL; 53- Department of Radiation Oncology, Baylor College of Medicine , Houston, USA; 54- Department of Molecular and Cellular Oncology, The University of Texas MD Anderson Cancer Center, Houston, USA

## Abstract

**Purpose:**

Osteoradionecrosis of the jaw (ORNJ) is a severe iatrogenic disease characterized by bone death after radiation therapy (RT) to the head and neck. With over 9 published definitions and at least 16 diagnostic/staging systems, the true incidence and severity of ORNJ are obscured by lack of a standard for disease definition and severity assessment, leading to inaccurate estimation of incidence, reporting ambiguity, and likely under-diagnosis worldwide. This study aimed to achieve consensus on an explicit definition and phenotype of ORNJ and related precursor states through data standardization to facilitate effective diagnosis, monitoring, and multidisciplinary management of ORNJ.

**Methods:**

The ORAL Consortium comprised 69 international experts, including representatives from medical, surgical, radiation oncology, and oral/dental disciplines. Using a web-based modified Delphi technique, panelists classified descriptive cases using existing staging systems, reviewed systems for feature extraction and specification, and iteratively classified cases based on clinical/imaging feature combinations.

**Results:**

The Consortium ORNJ definition was developed in alignment with SNOMED-CT terminology and recent ISOO-MASCC-ASCO guideline recommendations. Case review using existing ORNJ staging systems showed high rates of inability to classify (up to 76%). Ten consensus statements and nine minimum data elements (MDEs) were outlined for prospective collection and classification of precursor/ORNJ stages.

**Conclusion:**

This study provides an international, consensus-based definition and MDE foundation for standardized ORNJ reporting in cancer survivors treated with RT. Head and neck surgeons, radiation, surgical, medical oncologists, and dental specialists should adopt MDEs to enable scalable health information exchange and analytics. Work is underway to develop both a human- and machine-readable knowledge representation for ORNJ (i.e., ontology) and multidisciplinary resources for dissemination to improve ORNJ reporting in academic and community practice settings.

## INTRODUCTION

Osteoradionecrosis of the jaw (ORNJ) is a morbid iatrogenic disease experienced by cancer patients treated with radiation therapy (RT) to the head and neck region. ORNJ incidence in head and neck cancer (HNC) survivors is estimated to range from 5 to 15% with higher rates associated with risk factors such as poor oral hygiene, pre- and post-RT dental extractions, and high maxillary and/or mandibular radiation dose/volumes.^1–6^ ORNJ can manifest as early as 6 months post-RT, and if not diagnosed and successfully managed at its initial stage, can progress to morbid states of symptom burden and poor quality of life (QOL) via tooth loss, compromised orofacial function, and pain.^7–9^ Financial repercussions of progressive ORNJ are substantial as major medical and surgical interventions for ORNJ can cost up to $170,000 per patient.^10,11^

Until 2023, there was no International Classification of Diseases (ICD) diagnostic code specific to ORNJ, resulting in an inability to formally report and assess ORNJ incidence.^12^ Moreover, there has been no consensus on specific diagnostic criteria that represent different ORNJ disease states, as well as scant agreement regarding severity or grading of ORNJ. This lack of consensus is discernable within existing literature which includes at least 16 ORNJ staging/grading systems published over a span of 4 decades, some of which only include data elements on treatment required or response to therapy in their classification schema.^1,13–27^ This ambiguity in what constitutes minimum data elements (MDEs) to diagnose and stage ORNJ translates to gross variability in estimating the true event rate and poor intelligibility of cross-scale reporting. Ultimately, the state of classification hinders reusability of ORNJ data for comparing outcomes, building data-driven models of disease risk, and facilitating large-scale, multi-institutional interventional trials to mitigate ORNJ.

To address this unmet need, the Orodental Radiotherapy-Associated Late-Effects (ORAL) Consortium was formed, comprised of 69 internationally recognized multidisciplinary experts for consensus formation through a modified Delphi study. The specific aims of this study include:

1. Assessment of contextual overlap between existing ORNJ definitions and disease severity criteria;
2. Characterization of item-specific inter-rater and inter-specialty conceptual agreement across definitional systems for ORNJ;
3. Determination of consensus-derived components of extant and proposed consensus ORNJ grading systems using best informatics principles; and
4. Generation of standardized, clinically relevant criteria (i.e., MDEs) for clinical and radiographic diagnosis, assessment, and reporting of ORNJ across interdisciplinary care.

## METHODS

### The Consensus Process

**Figure 1.**
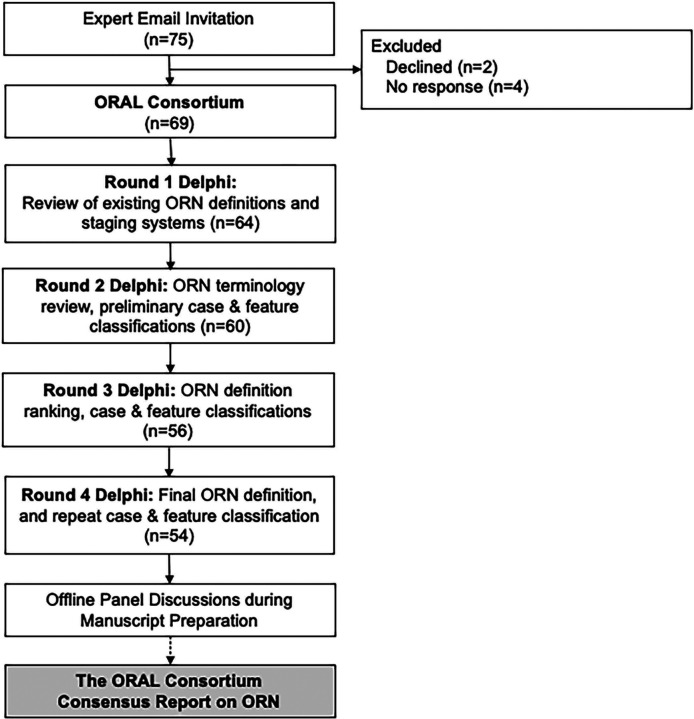
Delphi Consensus Process Flow Chart. Abbreviations: ORN: Osteoradionecrosis

The consensus process was achieved via Delphi method, and is not an update to pre-existing guidelines.^28–30^ A process flow chart for this study is shown in Figure 1, and an Accurate Consensus Reporting Document (ACCORD) guideline checklist for reporting consensus methods in biomedicine can be found in S1_ACCORD.^31^ A group of international multidisciplinary oncology and oral/dental specialists were invited electronically to participate (n=75). Members of the ORAL Consortium participated in at least one Delphi survey (n=69). Surveys were developed in REDCap^®^ and Qualtrics (see S2 series).^32,33^ Each questionnaire included an introduction, primary objectives for the round, and aggregated group feedback for consensus building. After each round, items meeting consensus were reported in the following rounds as “consensus reports,” and no further questions were asked on those items. Details on Delphi methodology and statistical analysis can be found in S3_Methods. No patients were involved, and this study was approved by our Institutional Review Board (MDA PA 2020–1096).

## RESULTS

### The International ORAL Consortium

Characteristics of the ORAL Consortium are shown in Table 1 with representatives from head and neck surgery, radiation oncology, medical oncology, oral and maxillofacial surgery (OMFS), oral oncology/oral medicine, and other specialties. Nearly half were women (43%), and the average age and time in practice were 47 years and 15 years (range, 0–38), respectively. Experts estimated a 7% annual incidence of ORNJ in their practices and treated a median of 4 cases of ORNJ per year. Participation throughout the study remained high, with 64 (93%), 60 (87%), 56 (81%), and 54 (78%) experts responding to rounds 1 through 4, respectively, and 64% of the Consortium participating in all four rounds.

### Consensus-based Definition of ORNJ (high consensus, 86%)

ORNJ is defined *pathognomonically* by the Consortium as “a condition in which there is a loss of blood flow to bone tissue, which causes the bone to die. Findings of bone death may be clinical (i.e., exposed bone) and/or radiographic (i.e., sclerosis, pathologic fracture). It is caused by exposure to ionizing radiation and may occur at some point in time after radiation and in the absence of active disease (i.e., cancer) in the site of bone death.” In contrast to several existing scales, the consensus definition does not require an explicit time duration of exposed bone nor explicit exclusion of concurrent local inflammation or infection (i.e., osteomyelitis). It also incorporates the capacity to formally diagnose ORNJ using imaging criteria; 61/63 (97%) of experts felt it was very/somewhat important to include “radiographic findings” in a formal definition.

Achieving this consensus-based definition required substantial iterative questioning of minimum data elements (MDEs) and derivation of expert- and specialty-specific implicit conceptual frameworks through case-based questioning (see S4_Results). In round 1, six distinct elements were identified; only 3 conceptually included across highly rated definitions/scales: 1) *exposed or necrotic bone*, 2) *RT-induced disorder*, and 3) *absence of tumor* (i.e., primary or recurrence). Round 2 capitalized on MDE identification, asking experts to review MedDRA and SNOMED-CT terminologies/nomenclatures for ORNJ from which 83% (43/52) and 85% (45/53) of panelists agreed that the Consortium’s definition should align with these existing terminologies, respectively. There was also high consensus (80%) that 1) a (then-current) ICD-10 diagnostic code for ORNJ was needed and 2) the National Cancer Institute’s definition for osteonecrosis included a relevant term of ‘vascular insufficiency’ in defining the pathophysiology of bone death.^34^ A total of 87% (52/60) experts agreed this vascular term or ‘devascularization’ should be included in any definition of necrosis. This resulted in the Consortium’s first consensus statement (CS):

CS 1: The Consortium’s definition for ORNJ will reflect features in existing terminologies, including:

1. Bone Disorder
2. Radiation injury and/or caused by ionizing radiation
3. Loss of blow flow or vascular insufficiency AND findings of bone death/necrosis

### The Time Feature for Diagnosing ORNJ Conundrum

**Figure 2.**
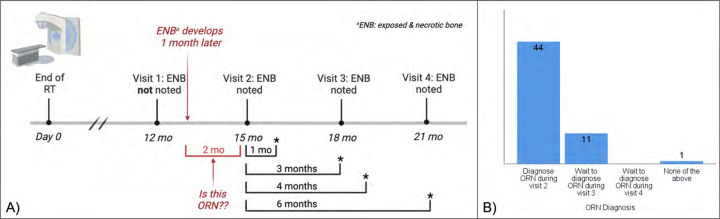
Time Feature and ORNJ Diagnosis Scenario (A) and Expert Panel Consensus (B).

During round 1, a time feature was identified in 6 of 9 published definitions, and 54 (92%) of experts favored including a time component (i.e., duration of exposure) in the Consortium’s definition for ORNJ. However, the minimum duration of exposed bone required for diagnosing ORNJ varies significantly in the literature from 1–6 months.^15,35–37^ Adding a time feature posed a significant challenge when considering different provider surveillance schedules after RT (time bias). Therefore, a time-feature case scenario (Figure 2) was presented during round 2 to further refine the nuances of time and its relevance as a diagnostic feature for ORNJ. After reviewing 3 existing terminologies, which do not include a diagnostic time feature for ORNJ, 70% (41/59) of experts strongly/somewhat agreed that a diagnosis of ORNJ could be met without a time element while 84% (49/58) agreed a time element is useful for assessing response to therapy, distinct from staging. This analysis resulted in the designation of an MDE for **date_of_assessment** (i.e., date of clinical or radiographic evaluation post-RT) that should be included in ORNJ-related databases for iterative surveillance assessments.

CS 2: While valuable to report and reflect the duration of non-healing changes observed in irradiated bone, the time or duration of exposed bone is not a mandatory diagnostic feature for ORNJ.

### Does Exposed Bone Equal Necrotic Bone?

This topic was reviewed to identify differences in conceptualization of exposed bone which may result from other causative agents like trauma after a dental extraction. When asked if all cases of exposed bone automatically equate to necrotic bone, 87% (52/60) disagreed.

CS 3: Not all cases of exposed bone are necessarily considered to be exposed necrotic bone.

### Intact Mucosa and Diagnosing ORNJ

Fifty-six (93%) of experts agreed that ORNJ can be diagnosed in cases with intact mucosa. This is significant from a clinical perspective as most published ORNJ definitions include the presence of clinically exposed bone whereas the Consortium’s definition allows for a clinical or radiographic manifestation of features associated with ORNJ. Possible examples include intact mucosa with new lytic and/or mixed sclerotic changes seen on panoramic radiograph or cortical deconstruction of previously irradiated bone noted on surveillance CT scan.

CS 4: ORNJ can be diagnosed in a patient treated with RT presenting with intact mucosa (i.e., no clinical bone exposure) if there is supporting radiographic evidence of bone death/necrosis.

### Identification of Elements to Stage ORNJ and Precursor Conditions

#### Review of Existing Staging and Grading Systems

Round 1 provided experts with a comprehensive overview of 15 staging/grading systems. After presenting each classification system, experts were asked 1) if they had ever used the staging/grading system before, 2) to rate the effectiveness of each system for classifying ORNJ, and 3) to classify three clinical scenarios with each system.

**Figure 3.**
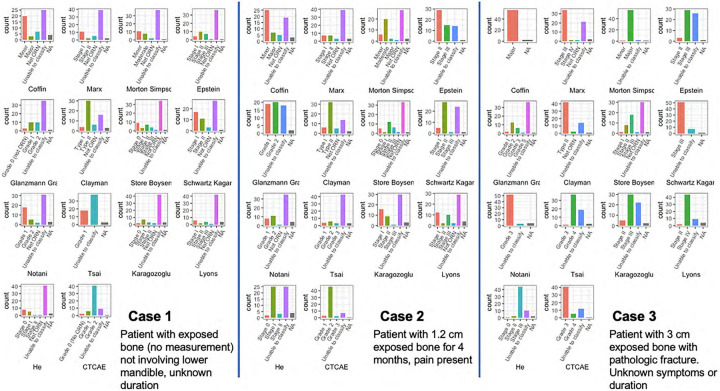
Three Case Classification Using Existing Staging Systems. Magenta bars represent ‘unable to classify’ across all staging systems.

Expert utilization and expert-deemed effectiveness/utility ratings of staging/grading systems can be found in Figure S1. The most commonly used system was the Common Terminology Criteria for Adverse Events (CTCAE)^26^ (n=41, 70%) followed by Notani^20^ (n=18, 32%), and Marx^14^ (n=18, 31%). The inability to consistently classify the text-based cases using existing systems was evident during round 1 (Figure 3). The inability to classify ranged from 16–76% (Case 1), 12–70% (Case 2), and 0–62% (Case 3). Interestingly, the CTCAE resulted in higher completion rates of staging cases 1 and 2 with only 16% and 12% of the Consortium reporting an inability to classify the case. Four additional systems were requested by experts and reviewed during round 2.^38–40^ Of these, MRONJ, a staging system for medication-related osteonecrosis, was considered the most effective for diagnosing ORNJ (57%) but its use should be cautioned as it references a distinct causative agent (i.e., medications, not RT). None of the reviewed classification methods met the consensus threshold for being highly effective at staging ORNJ, reflecting a critical need for developing and adopting a comprehensive and updated classification system with explicit clinical and imaging features specified to clearly differentiate between stages. The Watson et al. risk-based model for ORNJ (ClinRad), which was recently published and shown to outperform existing systems for classifying ORNJ severity,^1^ was presented to the Consortium during round 4 and was evaluated favorably with 92% (48/52) of experts agreeing with the clinical-radiographic system.

#### Guidelines Regarding Application of the CTCAE Grading System for ORNJ

The potential recommendation to use the CTCAE system to stage ORNJ did not meet consensus (52%). Overall, 90% of experts agreed that the CTCAE system is still valuable for toxicity reporting but not comprehensive enough for classifying ORNJ, and 95% agreed it could be used in parallel with another ORNJ classification method.

CS 5: CTCAE is a valuable toxicity grading system that should be used in parallel with, but not replace, an ORNJ classification system inclusive of explicit and stage-specific clinical and radiographic features.

#### Time Feature and ORNJ Staging

Similar time feature-related questions were asked for *staging* disease severity. During round 2, 68% (40/59) agreed that a staging system for ORNJ should be developed without a mandatory inclusion of a time feature. When rephrased to state that a time feature, which is not a clinical exam or radiographic finding, could be an optional modifier but not a necessary feature for staging ORNJ, 83% (47/57) of the Consortium agreed (Figure S2). The Consortium also demonstrated high agreement (85%, 49/58) in considering time features relevant for assessing response to therapy but that therapy response should be separated from a staging system characterizing ORNJ.

CS 6: A time feature is not necessary for staging ORNJ. However, reporting time features may be complementary for monitoring the duration of observed ORNJ alone or in response to any therapy.

#### Symptoms and ORNJ Staging

Specific disease-induced symptoms (i.e., pain) or non-explicit symptom presence has been used for upstaging ORNJ.^15,23,24^ When reviewing symptoms, 72% (41/57) of experts agreed that symptoms are ambiguous and therefore should not be stage-defining. However, documenting clear descriptions of specific symptoms including their onset, temporal profiles, and resolution, if any, is highly encouraged in parallel to explicit clinical/radiographic findings so that a **functional** classification system can be developed in the future. For symptom surveillance, the Consortium recommends using standardized assessment tools for patient-reported outcomes (PROs).^41–44^

CS 7: Symptoms associated with ORNJ should not be used as stage-defining features. However, longitudinal reporting of the presence or absence of concurrent symptoms using validated patient-reported outcomes (PRO) question items is strongly encouraged.

#### Staging Data Element Extraction and Classification

**Figure 4.**
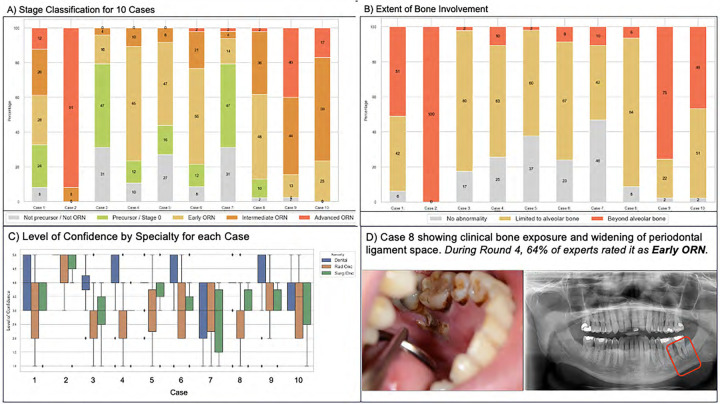
Stage and Bone Extent Classifications and Specialty-Based Levels of Confidence During Round 3.

A data element tracker flowsheet was developed inclusive of all clinical, radiographic, therapy, and treatment response elements identified in reviewed staging/grading systems (Figure S3). Experts were then asked to rate the importance of each feature (not important, somewhat important, very important). Figure S4 shows the distribution of expert responses with only three data elements being considered somewhat/very important by the entire group: pathologic fracture, extent of bone involvement, and exposed bone. A complete description of the MDE development process can be found in S4_Results. Figure 4 shows the heterogeneity in stage and extent of bone involvement classification for ten image-based case studies. A final list of Consortium-approved MDEs for staging ORNJ and precursor stages is shown in Table 2 along with recommended coding standards for building artificial intelligence/machine learning (AI/ML) ready datasets.

CS 8: All cases with clinical and/or radiographic evidence of a pathologic fracture or fistula formation (i.e., oro-cutaneous, oro-antral, oro-nasal) involving previously irradiated bone should be reported as advanced-stage ORNJ. These individual disorders are considered stage-defining MDEs, and each should be reported separately.

### Specialty-Specific Knowledge Siloes & Inter-Rater Reliability

Siloes of knowledge may occur through different knowledge acquisition per specialty-based training programs and/or practice patterns. Within the ORAL Consortium, a significant difference was found in the clinical utilization of different imaging modalities (i.e., CT, MRI, panoramic radiograph; see S4_Results). Educational resources were provided to improve inter-rater reliability between rounds. With regards to the image-focused educational resources, 84% of experts found the resources helpful, and 92% were interested in having an updated, comprehensive, multidimensional atlas as a support tool for case classification.

### Recommendation for Multidisciplinary MDE Adoption

Given substantial variation in the classification of cases among experts, particularly ‘threshold’ cases which may be upstaged based on non-explicit clinical and/or radiographic imaging features (i.e., quantitative measurement of clinical bone exposure), the following consensus statements were developed:

CS 9: The Consortium strongly recommends the adoption of ORNJ-focused MDEs in multidisciplinary clinical practice and clinical trial design to reduce misclassification risks and to facilitate ‘stage migration’ across classification models.

CS 10: Inclusion of serial photographs in a patient’s medical record during post-RT surveillance, especially once changes in the mucosa (i.e., ulceration) or bone (i.e., progressive bone exposure) are detected, is strongly recommended. Caliper or ruler-based measurements of clinical bone exposure should also be recorded for at least the longest dimension in millimeters under the MDE, clinical:exposed_bone_length_in_mm.

## DISCUSSION

In this expert-based, iterative Delphi method study, we have generated an international, multidisciplinary-approved definition for ORNJ along with ten consensus statements and nine distinct minimum data elements that should be serially documented during dental and oncology post-RT appointments for cancer survivors undergoing ORNJ surveillance. These MDEs characterize static (i.e., date of assessment) and dynamic (i.e., progressive radiographic changes) features that can be used for meaningful classification of ORNJ and precursor stages.

During this Delphi modeling, several authors were simultaneously involved in the development and publication of the ClinRad model^1^ and the ISOO-MASCC-ASCO joint guideline for prevention and management of ORNJ.^9^ The Consortium’s definition for ORNJ is in alignment with the new ISOO-MASCC-ASCO guideline, which *operationally* characterizes ORNJ as a “radiographic lytic or mixed sclerotic lesion of bone and/or visibly exposed bone and/or bone probed through a periodontal pocket or fistula occurring within an anatomical site previously exposed to a therapeutic dose of head and neck radiation therapy.” Moreover, both definitions demonstrate a significant departure from using a time-limiting feature for diagnosing ORNJ, thereby addressing current issues with time bias.

The ClinRad system, which outperformed staging methods such as the Notani, LENT-SOMA, and Store systems, incorporates observable clinical (i.e., Probe-to-Bone [PTB] tests) and radiographic features and uses the alveolar bone as a distinguishable threshold for disease.^1^ This new staging model of ORNJ has been adopted by the new ISOO-MASCC-ASCO ORNJ guidelines.^9^ The ORAL Consortium’s favorable review of the ClinRad system prior to the publication of the guidelines further supports its utilization as it incorporates most of the MDEs identified in this study. However, we also demonstrate several staging challenges that should be addressed by providers prior to implementing the ClinRad system. There still exists a conceptualization discordance for MBS, namely whether or not it is related to ORNJ. ^1^The Consortium favors the classification of MBS as a precursor event to ORNJ that has a higher likelihood of resolving over time compared to other MDE features or feature combinations. More importantly, utilization of a quantitative measurement like **clinical:exposed_bone_length_in_mm** and inclusion of clinical photographs in the patient’s medical and dental records can facilitate data harmonization among interprofessional healthcare providers at different centers treating the same patient.

The concept of specialty-specific knowledge siloes is introduced in this study and is important to recognize since diagnosis and management of ORNJ is often a multidisciplinary task. Overall, providers tend to exhibit high agreement in perceiving severe presentations of ORNJ; however, subtle variations in physical exam or radiographic feature interpretations may cascade into differing classifications of disease for the same patient examined by different specialists. One approach to mitigate this discrepancy is by standardizing the use of MDEs across specialties and generating interdisciplinary, multi-modality (i.e., OPG, CT, MRI) image-focused educational materials. Biomarkers for ORNJ and its precursor stages are also being investigated, including dynamic contrast-enhanced (DCE) MRI parameters (K_trans_ and V_e_,) for assessing risk, diagnosis, and progression or treatment response of ORNJ.^45^ The Consortium supports the consideration of DCE-MRI changes indicative of vascular insufficiency in previously irradiated jaw bones as a precursor event to ORNJ. Subsequent clinical guidelines will be necessary for outlining optimal MRI-based ORNJ surveillance regimens and specification of MRI-specific MDEs.

While this interdisciplinary Delphi study has several strengths such as the Consortium size (n=69) and sustained level of engagement, there is underrepresentation of specialties (e.g. radiology (n=1) that may provide additional expertise on identifying stage-defining radiographic features across imaging modalities. Fleiss kappa statistics can provide substantial insight on how reliability experts classify cases (i.e., interrater reliability), but it does not provide information on whether those classifications represent the true disease state (i.e., validity).^46^ Lastly, while several consensus-defining methods exist,^47,48^ we chose a simple agreement threshold as it is commonly used, easy to interpret, and reinforced through iterative requestioning to produce metrics of reliability.

In conclusion, the Consortium’s definition of ORNJ and associated MDEs should be adopted as standards for reporting by head and neck surgery, oncology, radiology, and dental providers in clinical practice, research, and clinical studies. Collectively, these enable scalable health information exchange and AI/ML data readiness for rigorous modeling of the disease and its precursor stages. ORNJ-focused MDEs are also synergistic with recently published guidelines and newer risk-based ORNJ models that recognize the importance of combining clinical and radiographic features for ORNJ characterization. Lastly, additional efforts are underway to formalize an ORNJ ontology, develop radiology standards and automated imaging feature identification and reporting, and formulate and disseminate interdisciplinary educational resources to mitigate barriers to accurate ORNJ staging.

## Supplementary Material

1 Figure S1. Personal Use and Utility Rating of Existing ORN Staging/Grading Systems
Figure S2. The Time Feature and ORN Staging
Figure S3. Data Element Tracker
Figure S4. Rating of ORN Data Elements from Staging/Grading Systems
Figure S5. Round 2 Data Element Classification
Figure S6. Round 2 Scenario Classification

## Figures and Tables

**Table 1.** The ORAL Consortium Expert Characteristics

	Overall (N=69)
**Sex**	
Female	30 (43 %)
Male	39 (57 %)
**Age (years)**	
Mean (SD)	47 (± 9.1)
Missing	7 (10.1%)
**Specialty**	
Medical Oncology	3 (4 %)
OMFS	12 (17 %)
Oral Oncology/Medicine & Dentistry	16 (23 %)
Radiation Oncology & Physics	28 (41 %)
Radiology	2 (3 %)
Surgical Oncology	8 (12 %)
**Specialty Group**	
Dental	28 (41 %)
Other	5 (7 %)
Rad Onc	28 (41 %)
Surg Onc	8 (12 %)
**Practice Community**	
Urban (>75,000 population)	63 (91 %)
Suburban (10,000–75,000)	2 (3 %)
Rural (< 10,000)	0 (0 %)
Missing	4 (5.8%)
**Practice Setting**	
Academic	66 (96 %)
Non-Academic	1 (1 %)
Private	2 (3 %)
**Time in Practice (years)**	
Mean (SD)	16 (± 8.6)
Missing	4 (5.8%)
**Approximate HNC Patient Caseload (Per Month)**	
Mean (SD)	55 (± 70)
Missing	6 (8.7%)
**Have You Evaluated/Treated ORN?**	
Yes	60 (87 %)
No	5 (7 %)
Missing	4 (5.8%)
**Average Patients Evaluated for ORN Annually (N)**	
Mean (SD)	51 (± 170)
Missing	10 (14.5%)
**Average Patients Treated for ORN Annually (%)**	
Mean (SD)	13 (± 21)
Missing	9 (13.0%)
**Average HNC Patients With ORN (%)**	
Mean (SD)	7.2 (± 4.8)
Missing	11 (15.9%)

HNC, Head and Neck Cancer; OMFS, Oral and Maxillofacial Surgery; SD, Standard Deviation

**Table 2.** Minimum Data Element List

Minimum Data Element	Example Value Names	Example SCTID	SCTID Class
***Time***			
date_of_assessment			
***Clinical***			
minor_bone_spicules	Present	52101004	Qualifier value
	Absent	2667000	Qualifier value
exposed_bone_length_in_mm mucosal_status	Present	52101004	Qualifier value
	Absent	2667000	Qualifier value
PTB_test_result	Positive	404684003	Qualifier value
	Negative	260385009	Qualifier value
disorder_present	Ulceration of oral mucosa	26284000	Disorder
	Orocutaneous fistula	472978005	Disorder
	Oroantral fistula	109675004	Disorder
	Oronasal fistula	370485008	Disorder
	Pathologic fracture	268029009	Disorder
***Radiographic***			
imaging type (DICOM standard)			
morphology	Bony sclerosis	37748009	Morphologic abnormality
	Osteolysis	30425001	Morphologic abnormality
	Bony erosion	788917000	Finding
	Thinning (i.e., cortical bone)	29143009	Finding
	Pathologic fracture	22640007	Morphologic abnormality
vertical_ab_abnormality	Above	352730000	Qualifier value
	Below	351726001	Qualifier value

Abbreviations: ab, alveolar bone; DICOM, Digital Imaging and Communications in Medicine; SCTID, Systemized Nomenclature of Medicine-Clinical Terms Identifier.

## Data Availability

In accordance with the *Final NIH Policy for Data Management and Sharing* NOT-OD-21–013, data that support the findings of this study are openly available in an NIH-supported generalist scientific data repository (figshare) at http://doi.org/10.6084/m9.figshare.25546723 no later than the time of an associated publication.
