# Supplementary material for: International Expert-Based Consensus Definition, Staging Criteria, and Minimum Data Elements for Osteoradionecrosis of the Jaw: An Inter-Disciplinary Modified Delphi Study
Source: medRxiv. 2024 Apr 9:2024.04.07.24305400. Preprint. [Version 1] doi: 10.1101/2024.04.07.24305400 (PMC11030490; doi:10.1101/2024.04.07.24305400)
Supplement: 1 — Figure S1. Personal Use and Utility Rating of Existing ORN Staging/Grading Systems Figure S2. The Time Feature and ORN Staging Figure S3. Data Element Tracker Figure S4. Rating of ORN Data Elements from Staging/Grading Systems Figure S5. Round 2 Data Element Classification Figure S6. Round 2 Scenario Classification [file NIHPP2024.04.07.24305400V1-supplement-1.pdf]

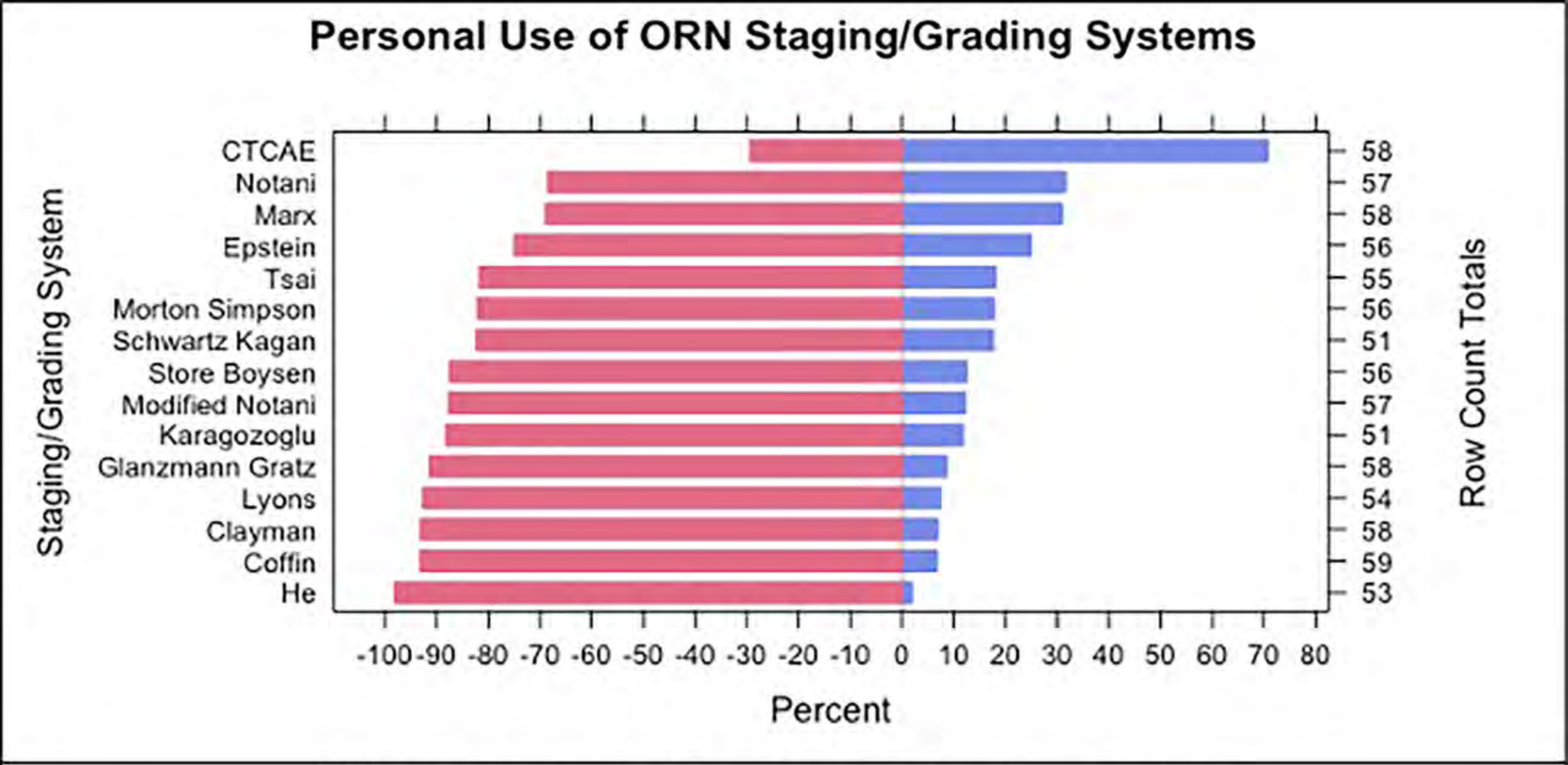

Personal Use of ORN Staging/Grading Systems
Staging/Grading System
CTCAE
Notani
Marx
Epstein
Tsai
Morton Simpson
Schwartz Kagan
Store Boysen
Modified Notani
Karagozoglu
Glanzmann Gratz
Lyons
Clayman
Coffin
He
Row Count Totals
58
57
58
56
55
56
51
56
57
51
58
54
58
59
53
-100 -90 -80 -70 -60 -50 -40 -30 -20 -10 0 10 20 30 40 50 60 70 80
Percent

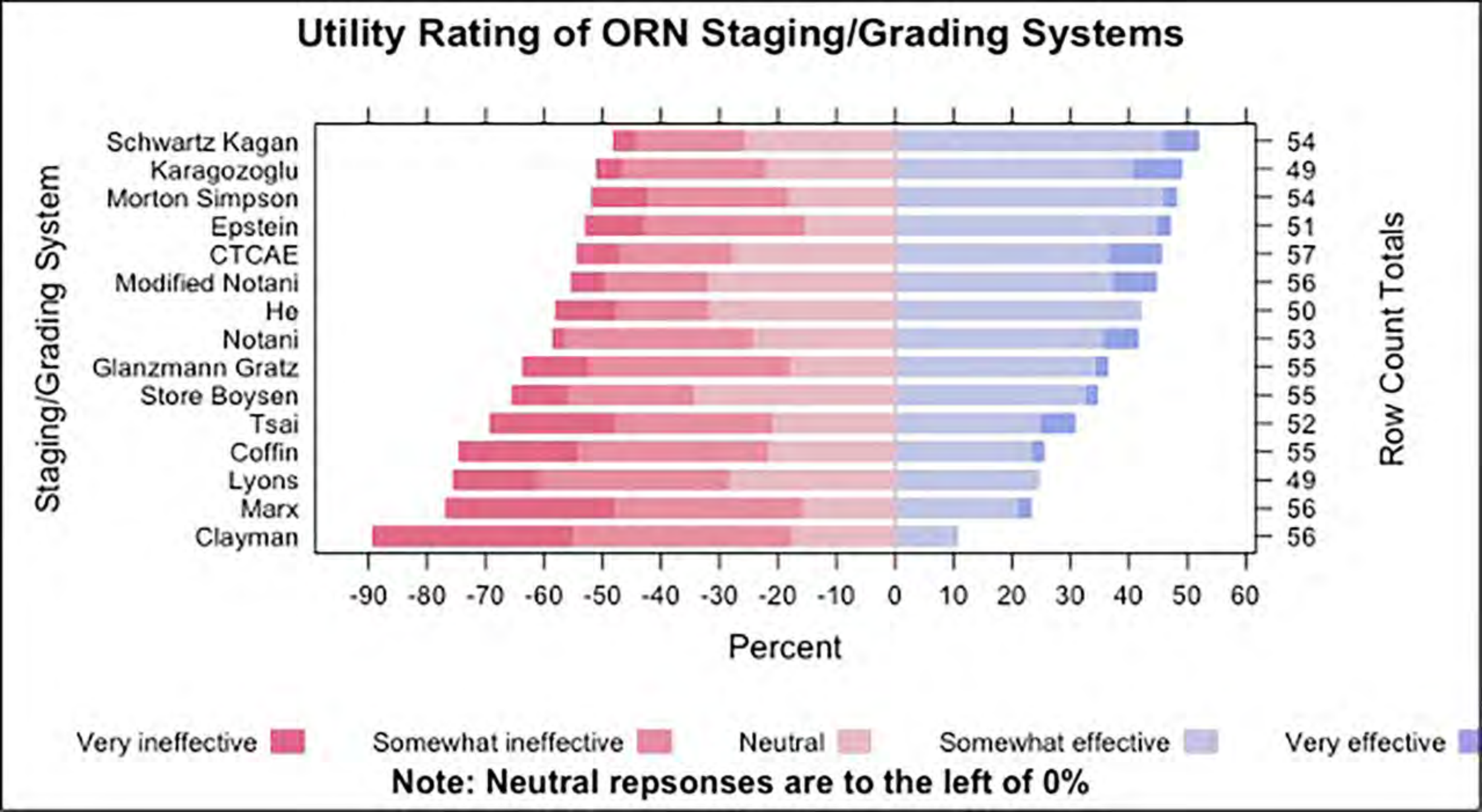

Utility Rating of ORN Staging/Grading Systems
Staging/Grading System
Schwartz Kagan
Karagozoglu
Morton Simpson
Epstein
CTCAE
Modified Notani
He
Notani
Glanzmann Gratz
Store Boysen
Tsai
Coffin
Lyons
Marx
Clayman
Row Count Totals
54
49
54
51
57
56
50
53
55
55
52
55
49
56
56
-90 -80 -70 -60 -50 -40 -30 -20 -10 0 10 20 30 40 50 60
Percent
Very ineffective
Somewhat ineffective
Neutral
Somewhat effective
Very effective
Note: Neutral repsonses are to the left of 0%

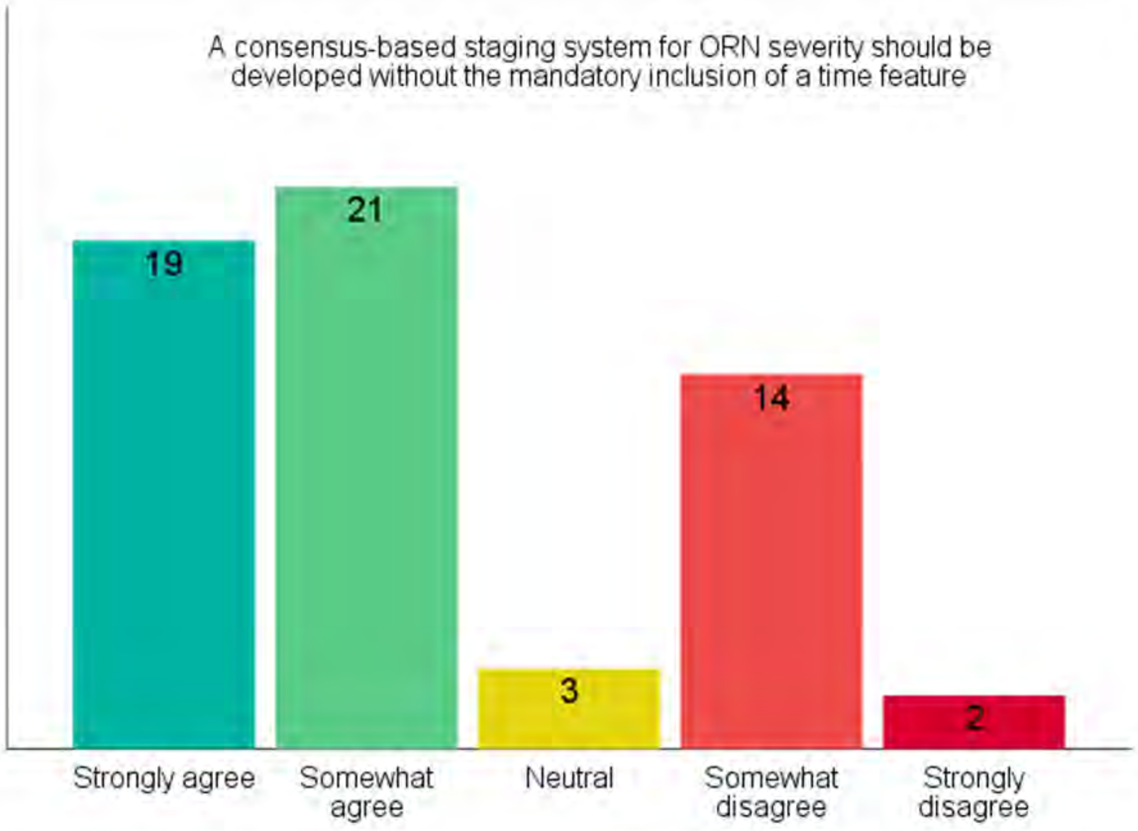
A consensus-based staging system for ORN severity should be developed without the mandatory inclusion of a time feature
19
21
3
14
2
Strongly agree
Somewhat agree
Neutral
Somewhat disagree
Strongly disagree

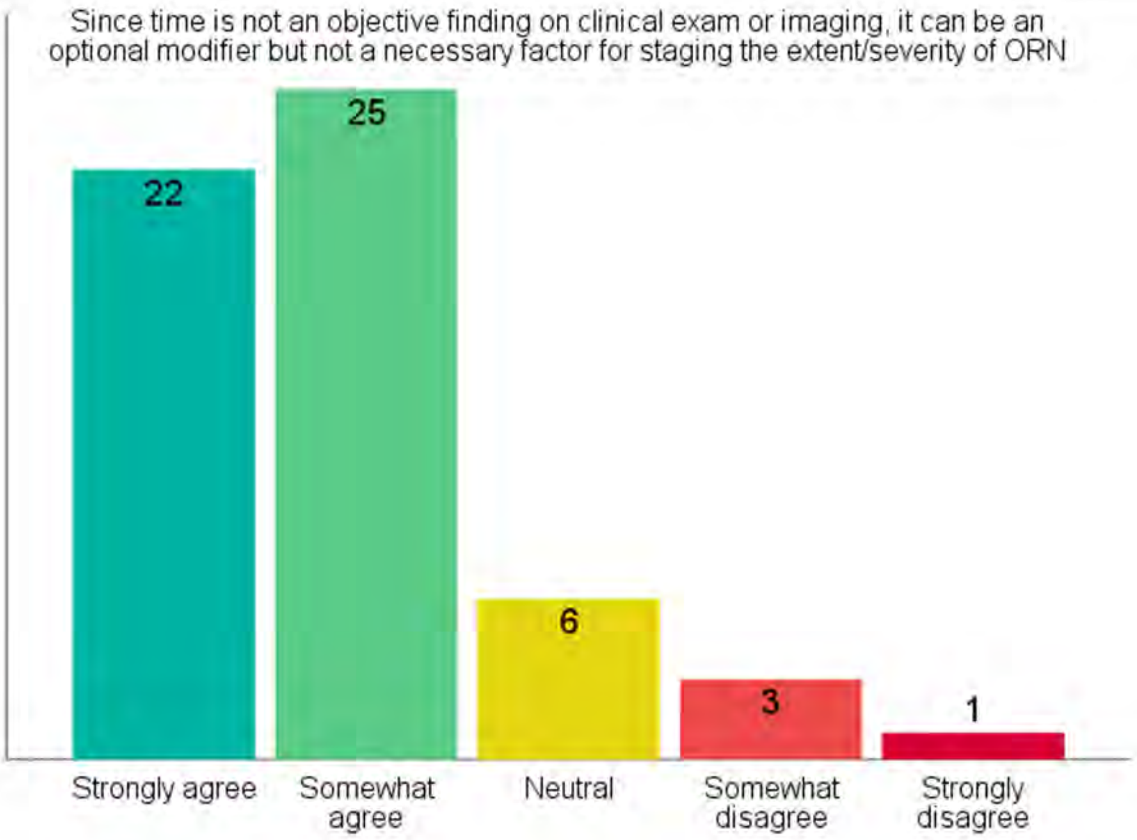
Since time is not an objective finding on clinical exam or imaging, it can be an optional modifier but not a necessary factor for staging the extent/severity of ORN
22
25
6
3
1
Strongly agree
Somewhat agree
Neutral
Somewhat disagree
Strongly disagree

	Characteristics												Treatments				Response to therapy			
Articles	Exposed bone	Extent of mandibular involvement	Ulceration (vs intact mucosa)	Bone spicules	Sequestra	Time	Pathologic Fracture	Orocutaneous Fistula	Sinus Formation	Signs of Infection	Symptoms	Positive radiological signs	Conservative medical therapy (Abx, PENTOCLO)	HBO	Minor debridement/ sequestrectomy	Bone resection/ reconstruction	Exposed bone after therapy	Granulation tissue (mucosal proliferation)	Inflammation	Healing
Notani (2003)	X	Alveolar bone; above/below inf alveolar canal					X	X												
Modified Notani (Shaw 2017)	X (extent: >= 20mm2)	Alveolar bone; above/below inf alveolar canal		X (MBS extent: < 20mm2)		</>= 6 mo	X	X												
Marx (1983)	X						X	X				X		X	X	X	Decrease; Continued/extended	X	X	X
Coffin (1983)	X	Full thickness of jaw or less			X	Wks-Months	X													X
Morton (1986)	X	Small; large areas	X	X		Months	X		X											X
Epstein (1987)	X					Months	X				X	X	X	X		X				X
Glanzmann (1995)	X				X	Months				X						X				
Clayman (1997)	X		X							X										
Store (2000)	X (not req for diagnosis)		X					X		X		X								
Schwartz (2002)	X	Cortical; localized; full thickness	X				X	X												
Tsai (2013)	X													X	X	X				
Karagozoglu (2014)	X	Lower border of mandible involved or not				</>= 1 mo		X			X	X								
Lyons (2014)	X (not req for diagnosis)	</>= 2.5 cm; involvement of inferior dental nerve					X	X			X		X		X	X				
He (2015)	X (not req for diagnosis)		X				X	X			X	X (</>= 2 cm lesion or fracture)								
CTCAE v5.0											X		X			X				
MRONJ (2022)	X	X (Imaging)	X				X	X	X	X		X								
LENT SOMA	X	X			X		X				X	X	X	X	X	X				
PMH ORN	X		X			X	X						X	X	X	X				
RTOG CTC											X	X								
Sum of elements	17	9	7	2	3	7	12	9	2	4	7	8	5	5	5	8	1	1	1	4

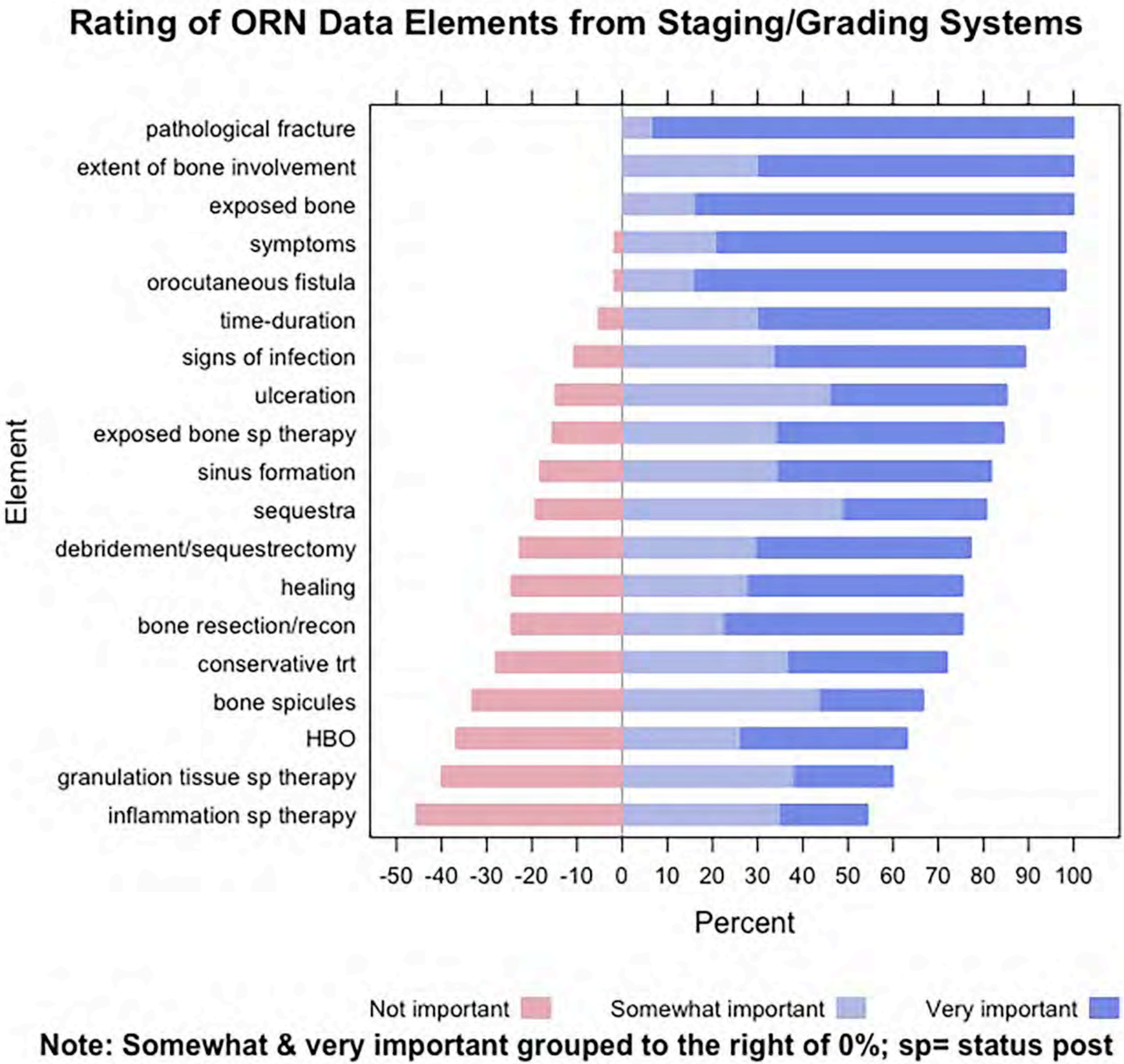

Rating of ORN Data Elements from Staging/Grading Systems
pathological fracture
extent of bone involvement
exposed bone
symptoms
orocutaneous fistula
time-duration
signs of infection
ulceration
exposed bone sp therapy
sinus formation
sequestra
debridement/sequestrectomy
healing
bone resection/recon
conservative trt
bone spicules
HBO
granulation tissue sp therapy
inflammation sp therapy
Element
-50 -40 -30 -20 -10 0 10 20 30 40 50 60 70 80 90 100
Percent
Not important
Somewhat important
Very important
Note: Somewhat & very important grouped to the right of 0%; sp= status post

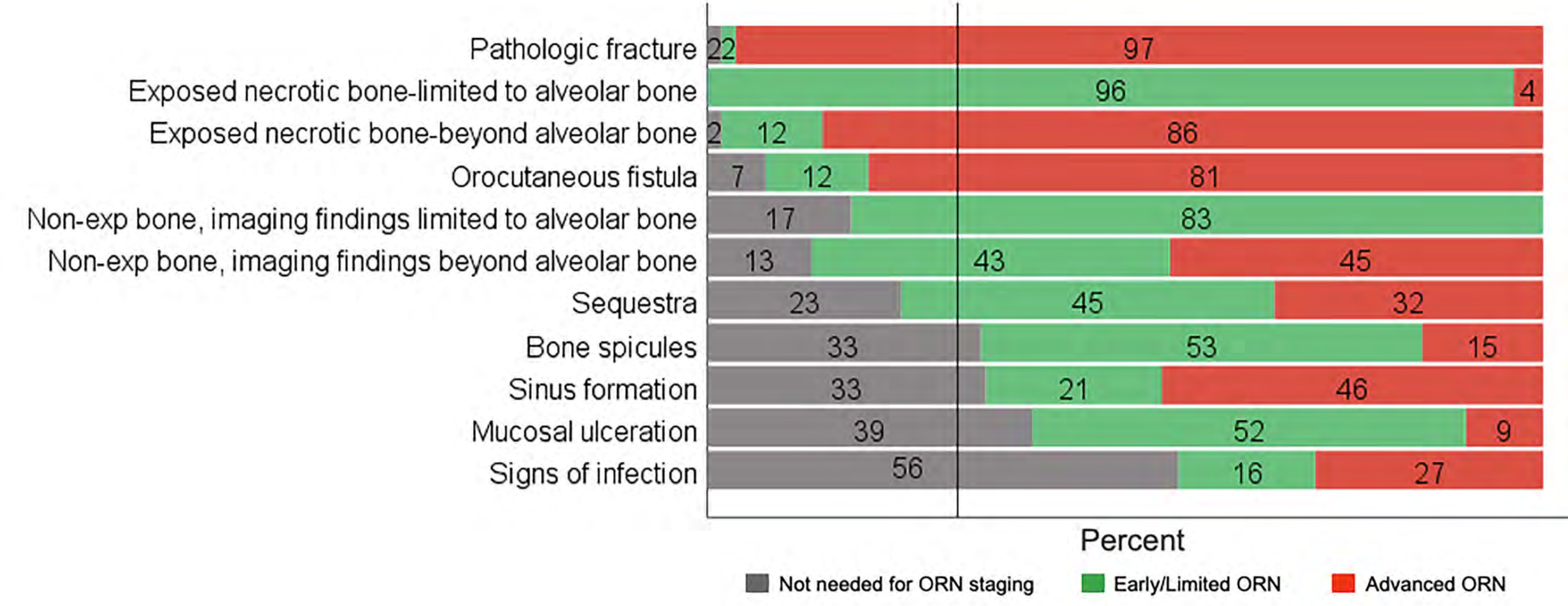

Pathologic fracture
Exposed necrotic bone-limited to alveolar bone
Exposed necrotic bone-beyond alveolar bone
Orocutaneous fistula
Non-exp bone, imaging findings limited to alveolar bone
Non-exp bone, imaging findings beyond alveolar bone
Sequestra
Bone spicules
Sinus formation
Mucosal ulceration
Signs of infection
2 2 97
96 4
2 12 86
7 12 81
17 83
13 43 45
23 45 32
33 53 15
33 21 46
39 52 9
56 16 27
Percent
Not needed for ORN staging
Early/Limited ORN
Advanced ORN

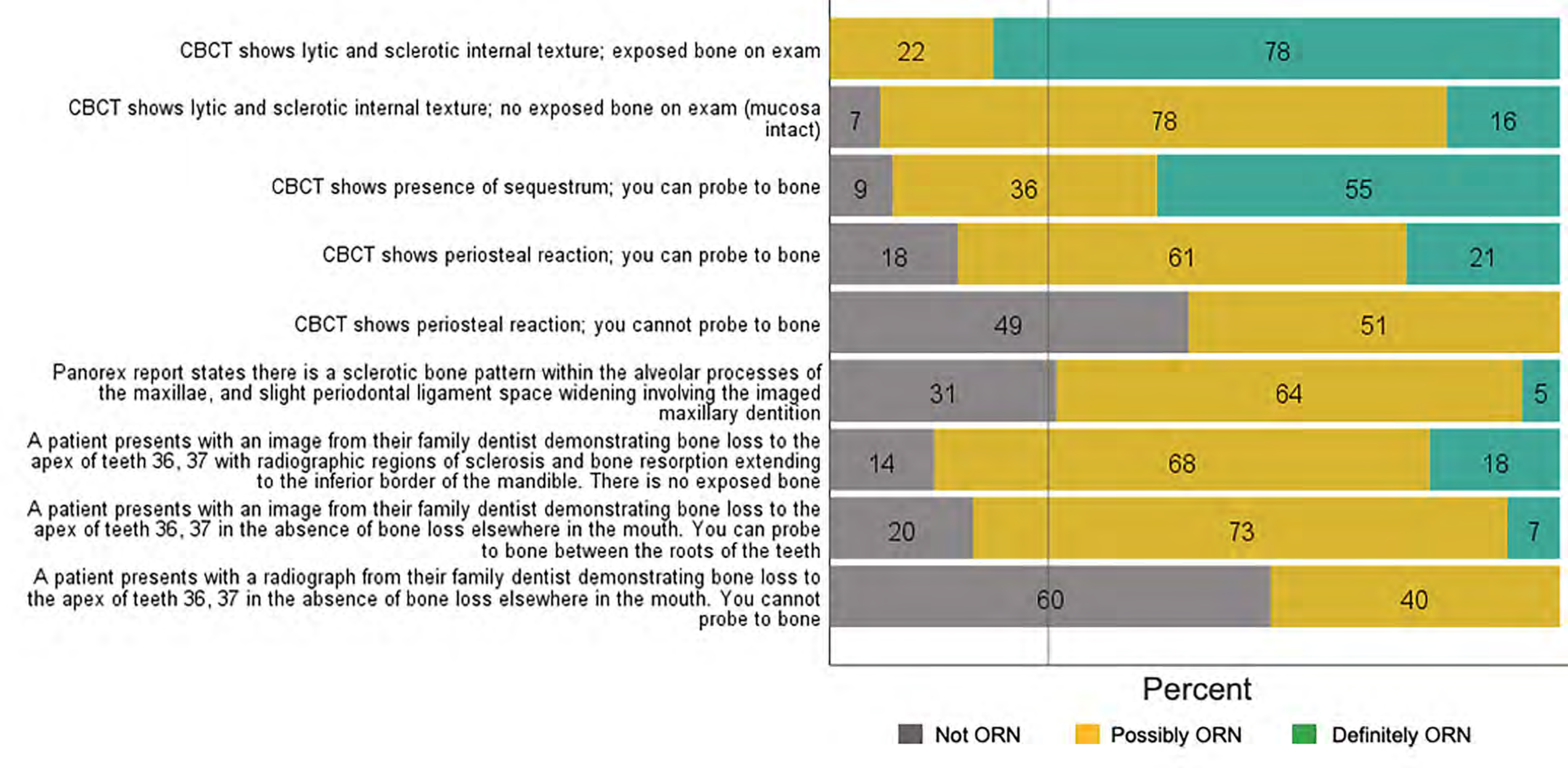
CBCT shows lytic and sclerotic internal texture; exposed bone on exam
22
78
CBCT shows lytic and sclerotic internal texture; no exposed bone on exam (mucosa intact)
7
78
16
CBCT shows presence of sequestrum; you can probe to bone
9
36
55
CBCT shows periosteal reaction; you can probe to bone
18
61
21
CBCT shows periosteal reaction; you cannot probe to bone
49
51
Panorex report states there is a sclerotic bone pattern within the alveolar processes of the maxillae, and slight periodontal ligament space widening involving the imaged maxillary dentition
31
64
5
A patient presents with an image from their family dentist demonstrating bone loss to the apex of teeth 36, 37 with radiographic regions of sclerosis and bone resorption extending to the inferior border of the mandible. There is no exposed bone
14
68
18
A patient presents with an image from their family dentist demonstrating bone loss to the apex of teeth 36, 37 in the absence of bone loss elsewhere in the mouth. You can probe to bone between the roots of the teeth
20
73
7
A patient presents with a radiograph from their family dentist demonstrating bone loss to the apex of teeth 36, 37 in the absence of bone loss elsewhere in the mouth. You cannot probe to bone
60
40
Percent
Not ORN
Possibly ORN
Definitely ORN

ACCORD
Accurate Consensus Reporting Document

Item No.	Section	Checklist Item	Page No.
T1	**Title**	International Expert-based Consensus Definition and Grading Criteria for Osteoradionecrosis of the Jaw: An International Disciplinary Modified Delphi Study	1
I1	**Introduction**	Osteoradionecrosis of the jaw (ORNJ) is a severe iatrogenic disease characterized by bone death after radiation therapy. There is a lack of a standard for disease definition and severity assessment, thereby resulting in mis-estimation of incidence, reporting ambiguity, and likely under-diagnosis on an international level.	3
I2		The aim of this modified Delphi study was to achieve multidisciplinary consensus on an explicit definition and phenotype of ORNJ with associated minimum data elements or MDEs to facilitate data standardization. The modified Delphi consensus method was selected for this study given its advantages in consensus formation including anonymity, iterative group feedback, and ability to perform the study remotely using electronic questionnaires. The intended audience is international multidisciplinary specialists involved in the surveillance and/or management of ORNJ (i.e., head and neck surgery, dentistry, oral maxillofacial surgery, oral medicine/oncology, radiation oncology, etc).	4
I3		This consensus exercise was not an update to pre-existing guidelines.	4
M1	**Methods Registration**	This study protocol has been registered and approved by the IRB at The University of Texas MD Anderson Cancer Center (MDA PA 2020-1096).	4
M2	**Selection of SC and/or panellists**	This study was led by Amy C. Moreno, MD MS and Clifton D. Fuller, MD PhD. Drs Moreno and Fuller are board-certified Radiation Oncologists at MD Anderson specializing in the treatment of patients with head and neck cancers. Dr. Moreno has a Master of Science degree in Clinical Informatics and Dr. Fuller is APBM board-certified in Clinical Informatics. The Fuller-Moreno lab focuses on developing and implementing standards in oncology to facilitate healthcare information exchange and scalable analytics. Dr. Moreno is the PI of a Mentored Career Development award (K01DE03052401A1) and research grant award (R21DE03108201) from the National Institute of Dental and Craniofacial Research (NIDCR) that funded this study.	1
M3		A group of internationally recognized multidisciplinary oncology and dental specialists were invited to participate in the study. Experts who authored or co-authored publications related to existing staging/grading systems for ORNJ or diagnosis, surveillance, and/or management of RT-related toxicities such as ORNJ were invited to participate in the ORAL Consortium. A total of 75 experts were initially approached given an expectation of a 50-75% response rate. The ORAL Consortium included 69 experts who participated in at least one of the Delphi rounds.	4
M4		Recruitment Process: All experts were sent a structured electronic invitation to participate in the study by Dr. Moreno who provided centralised oversight of invitations and the development and deployment of Delphi surveys. Experts were asked and allowed to suggest other members of the panel which were then reviewed and approved by Drs. Moreno and Fuller if their specialization and/or publications were related to the scope of the study. Experts interested in participating were subsequently sent a REDCap®-based survey which included 1) an introduction with details of the study, 2) a consent form, and 3) a link to proceed with round 1 of the ORN Delphi study. The use of electronic surveys allowed for experts residing in different countries and time zones to fully participate in the study. No compensation was advertised or given to participants.	4, S3
M5		No patients were involved in this study.	4
M6	**Preparatory research**	A literature review, which included a PubMed search of existing staging/grading systems for ORN/ORNJ, was performed to generate items used during the consensus exercise. Online queries were performed to identify existing terminologies and standards such as ICD, SNOMED-CT, and MedDRA to formulate items related to the explicit definition of ORNJ. During the study,	S3

		experts were asked to provide examples of clinical/radiographic scenarios of ORNJ or its precursor states to formulate case-based questions used in subsequent rounds for stage and extent of bone classification.	
M7		A PubMed search was performed in February 2023 for "(osteoradionecrosis) AND (staging OR grading)" which identified 447 publications since 1973 to 2023. Titles were screened for relevance resulting in abstract screening of 193 articles. Further screening was performed on full manuscript of original articles or reviews on one or more staging/grading systems for ORN/ORNJ. This resulted in the formal review and rating of 15 grading/staging systems by the ORAL Consortium during round 1. Additional literature searches during the course of the Delphi study were based on expert suggestions (i.e., LENT-SOMA, MRONJ, and ClinRad Model publications).	S3
M8		Existing staging/grading systems for ORN/ORNJ were summarized to panellists in a tabular format, accompanied by questions on personal use and utility of each system for ORN severity assessment. Examples of scientific evidence summarization in round 1 can be found in Supplemental Materials.	S2 series
M9	**Assessing consensus**	A modified Delphi method was used to collect panellist input and reach consensus.	4
M10		Question presentation varied based on the topic of interest but included single or multiple select formatting and use of a likert-type scale for questions on preference or utility of existing staging systems. Free-text items were also included in rounds to provide panellists to explain their responses and/or provide additional feedback for group review in the subsequent iteration of the Delphi surveys. All questionnaires have been provided as Supplemental Materials.	S2, S3
M11		A detailed report on the objectives of each consensus step can be found in S3_Methods. Briefly, round 1 included items on ORNJ definition preference, review of potential minimum data elements (MDEs), and rating of existing staging systems for ORNJ. Rounds 2 through 4 narrowed down the Consortium's consensus-based definition of ORJN. Rounds 3 and 4 provided clinical-radiographic scenarios for review and discussion, with group feedback being used to confirm a list of MDEs and consensus statements related to diagnosing and staging ORNJ.	S3
M12		Consensus during rounds 1 to 3 were prespecified to an agreement percentage of greater than or equal to 70% (i.e., strongly/somewhat agree) while in round 4, the threshold was lowered to 60% to formulate consensus statements on challenging topics. This percentage of agreement threshold was used as it is commonly used in published Delphi studies, easy to interpret by panellists, and can be reinforced through iterative requestioning to produce metrics of reliability. Experts were informed of consensus thresholds prior to initiating each survey.	S3
M13		Items that met the prespecified definition of consensus were not included in subsequent voting rounds.	4, S3
M14		For each step, responses were collected electronically through personalized survey links distributed using REDCap® or Qualtrics. At the beginning of each round, panellists were informed not to share their survey links with anyone.	S3
M15		Descriptive statistics were performed to summarize the Consortium expert demographics and quantitative anonymized group feedback (i.e., stage system ranking). Fleiss' kappa statistics were used to assess inter-rater agreement between all experts when classifying image-based cases on a categorical scale. For free-text responses, qualitative analysis was performed such as theme-based summation provided as anonymized group feedback in iterative surveys.	S3
M16		Survey instruments were piloted by the study coordinator to ensure correct formatting and delivery.	S3
M17		For iterative surveys, anonymized group feedback was provided to experts in the form of descriptive statistics, often reported in tabular format or via graphics. For example, quantitative statistics on ranked items were provided along with qualitative analysis on comments (i.e., thematic grouping of commentary).	S2 series, S3
M18		Anonymity was a major focus of the study design and guaranteed by using personalized electronic links to each survey for each panellist. Only Dr. Moreno had access to individual panellist response data which she used to summarize group feedback for	S3

		presentation to the panel on subsequent rounds. Group feedback did not include identifiable data. No phone call or face-to-face meetings were performed which can compromise anonymity of responses.	
M19		Drs. Moreno and Fuller, as head and neck cancer radiation oncology specialists, also participated in Delphi study as members of the ORAL Consortium. No extra oversight voting rights were given to Moreno and Fuller as consensus was only met if previously defined thresholds were met for each item during each round.	S3
M20	**Participation**	No financial incentives were advertised to reimburse participants for their time. Panelists were, however, provided with the opportunity to serve as group co-authors for related publications of the Delphi study if they met authorship criteria outlined by ICJME.	S3
M21		No adaptions to the surveys were performed (i.e., all surveys were conducted in the English language).	S3
R1	**Results**	Study preparation began in January 2023 with round 1 launching in March 2023, round 2 in May 2023, round 3 in August-September 2023, round 4 in February 2024, and manuscript review in March 2024 with approval by all co-authors completed in April 2024.	S3
R2		No deviations were performed in this study protocol.	S3
R3		There was excellent participation through each step of the Delphi study with 64 (93%), 60 (87%), 56 (81%), and 54 (78%) experts responding to rounds 1 through 4, respectively, and 64% of the Consortium participating in all four rounds. Relevant panel characteristics have been summarized in Table 1.	5, Table 1
R4		Outcome: In this expert-based, iterative Delphi study, we have generated an international, multidisciplinary-approved definition for osteoradionecrosis of the jaws (ORNJ) along with 10 consensus statements and 9 distinct minimum data elements that should be serially documented during dental and oncology post-radiotherapy appointments for cancer survivors undergoing ORNJ surveillance. These MDEs should be a standard for characterizing static (i.e., date of assessment) and dynamic (i.e., progressive radiographic changes) features that can be used for meaningful classification of ORNJ and precursor stages.	11
R5		Topic modification: the definition for ORNJ was iteratively modified throughout the study as described in the results section. This was necessary for consensus formation and full transparency of the process is available through review of the S2 series of questionnaires. A more detailed report of the results can be found in S3_results.	5-11, S2, S3
D1	**Discussion**	Strengths/limitations: This study has several strengths including the Consortium size (n=69) and sustained level of engagement from the experts. Moreover, anonymity amongst responders was maintained throughout the study with only summary statistics or de-identified commentary shared as group feedback during iterative rounds. However, there is underrepresentation of certain specialties like radiology that may have provided additional insight on identification of radiographic-based features of ORNJ. The consensus threshold was also lowered in the fourth round to ensure consensus formation on important topics (i.e., ORNJ definition), but the Consortium was notified of this prior to starting the round.	13
D2		The recommendations of this Delphi study are consistent and synergistic with pre-existing literature including new ISOO-MASCC-ASCO guidelines for ORNJ prevention and management. Our results are significant for the following reasons:<br>1. This study was considered in the development of the new ORNJ Guidelines and provides consensus-based support to the complimentary pathognomonic and operational definitions for ORNJ.<br>2. We identified and addressed a critical gap in staging criteria for the ASCO-endorsed ClinRad (Watson et al) classification model for ORNJ. Specifically, no criteria for extent of bone exposure noted on clinical exam is included in the ClinRad model. When testing experts with classifying cases with the same extent of radiographic bony changes but progressive clinical bone exposure, there was a significant shift towards reclassifying the more progressive stages as “advanced ORNJ”	2, 11

		instead of "intermediate ORNJ." This poses a significant problem in stage mis-classification which we discuss and provide a solution of incorporating a quantitative MDE for bone exposure which should be adopted with any ORNJ staging system.<br>3. To our knowledge, this is the largest consensus-derived study on ORNJ with 69 international, multidisciplinary specialists serving in the ORAL Consortium and contributing to the outlined recommendations.	
O1	**Other information**	No organizations have endorsed this study. However, members of the ISOO-MASCC-ASCO ORN Guideline Committee served as experts in this Delphi study.	COI forms
O2		Potential conflicts of interest: All co-author/expert conflicts of interests have been disclosed in this publication.	
O3		Funding Statement: This work was supported directly or in part by funding/resources from the National Institutes of Health (NIH) National Institute for Dental and Craniofacial Research (K01DE030524, U01DE032168, R21DE031082, R56/R01DE025248, R01DE028290); NIH National Cancer Institute (K12CA088084, P30CA016672); the NIH National Institute of Biomedical Imaging and Bioengineering (R25EB025787); the University of Texas MD Anderson Cancer Center Charles and Daneen Stiefel Center for Head and Neck Cancer Oropharyngeal Cancer Research Program; and the MD Anderson Image-guided Cancer Therapy Program.	Title Page

From: PLoS Med 21(1): e1004326. https://doi.org/10.1371/journal.pmed.1004326 For more information see: https://www.ismpp.org/accord

medRxiv preprint doi: https://doi.org/10.1101/2024.04.07.24305400; this version posted April 9, 2024. The copyright holder for this preprint (which was not certified by peer review) is the author/funder, who has granted medRxiv a license to display the preprint in perpetuity.
It is made available under a CC-BY 4.0 International license .

Data Dictionary Codebook

RADMAP-ORN Delphi Final 3.1.2023 (PID: 16807)

04/03/2024 3:30pm

Instruments

	#	Variable / Field Name	Field Label<br>*Field Note*	Field Attributes (Field Type, Validation, Choices, Calculations, etc.)
Instrument: **Round 2 ORN** (round_2_orn) Enabled as survey				
	1	[id]	Participant ID	text
	2	[table1]	Section Header: *Dear Oral Consortium Members, Thank you for your valuable responses to Round 1 of the ORN-RADMAP Delphi study! We received similar feedback from many of you in that Round 1 posed important questions and challenges with our current ability to 1) diagnose ORN due to the lack of a consensus-based explicit definition and 2) classify various cases of potential ORN given 15+ existing staging/grading systems. Using group feedback from Round 1, our main goals for Round 2 are: Begin formulation of an explicit concept definition for ORN. Differentiate between features of bone-based disorders (for ORN staging) and potential modifiers of disease severity. Summarize group feedback for RADMAP with secondary questions on visualizations. Note: For Likert scale-type questions (i.e., strongly disagree to strongly agree), please try to minimize the use of 'neutral' in order to assist with consensus formation in future rounds. Also, some questions may sound repetitive but are useful for consensus processes. Thank you! The focus of Round 3 will be confirmation of consensus-based ORN diagnostic criteria, and the build of a staging system/ontology based on Consortium-endorsed data elements.*<br>Table 1 summarizes the Consortium characteristics (for those who partially/fully completed Round 1 ORN/RADMAP surveys). Note: This table may be updated based on additional responses from Consortium members.	descriptive<br>(Attachment: Table1.PanelCharacteristics.JPG, Display format: Inline image/PDF)
	3	[def_groupfeedback]	Section Header: *SECTION 1: FORMULATION OF AN EXPLICIT DEFINITION FOR ORNGroup Feedback and Review of Existing Diagnostic Standards• None of the 9 published ORN definitions were selected by even 25% of the group as the most representative for the disease entity (see Figure 1). The top 4 candidates were: Harris (n=13, 22%), Schwartz (n=13, 22%), Karagozoglu (n=10, 17%), and Wong (n=8, 14%).*<br>From the top 4 definitions (Figure 1), 6 distinct features could be extracted with only 3 features included in all four: exposed [vs. necrotic] bone, RT-induced disorder [i.e., irradiated bone], and absence of tumor. Table 2: ORN Features per existing definitions and based on free text comments from the group Features Count in Top 4 definitions Favored in definition* (n, (%)) Exposed bone (vs. bone necrosis) 4 57 (100%) Caused by radiation therapy 4 Not asked, but recommended in comments Absence of tumor 4 Not asked, but recommended in comments Time feature 3 54 (92%) Imaging feature 1 56 (95%) Soft tissue necrosis (ulceration) 1 49 (83%) * Over 70% of the group rated this attribute as 'somewhat important' or 'very important' Figure 1. Rating of Existing ORN Definitions	descriptive<br>(Attachment: Fig 1. Published ORN definitions.jpg, Display format: Inline image/PDF)
	4	[desc_standards]	To date, there is no existing International Classification of Disease (ICD) diagnostic code specific to ORN. Other relevant internationally standardized medical classification terminologies and ontologies that do include codes for ORN include: Medical Dictionary for Regulatory Activities (MedDRA) Systemized Nomenclature of Medicine- Clinical Terms (SNOMED-CT) These definitions can serve as a foundation to build the Consortium's explicit definition for ORN. Let's first review how each standard defines disorders such as osteonecrosis (ON) and ORN.	descriptive
	5	[desc_meddra]	MedDRA is a 'clinically-validated international terminology' with a standardized hierarchy as described below (SOC --> HLGT --> HLT --> PT --> LLT). More specific diagnoses are typically coded as a 'Lowest level term' or LLT. MedDRA Hierarchy description from https://www.meddra.org/how-to-use/basics/hierarchy : "The structure of MedDRA is very logical. There are five levels to the MedDRA hierarchy,	descriptive<br>(Attachment: MedDRA codes for ON and ORN.jpg, Display format: Inline image/PDF)

medRxiv preprint doi: https://doi.org/10.1101/2024.04.07.24305400; this version posted April 9, 2024. The copyright holder for this preprint (which was not certified by peer review) is the author/funder, who has granted medRxiv a license to display the preprint in perpetuity.
It is made available under a CC-BY 4.0 International license .

			arranged from very specific to very general. At the most specific level, called "Lowest Level Terms" (LLTs), there are more than 80,000 terms which parallel how information is communicated. These LLTs reflect how an observation might be reported in practice.... Each member of the next level, "Preferred Terms" (PTs) is a distinct descriptor (single medical concept) for a symptom, sign, disease diagnosis, therapeutic indication, investigation, surgical or medical procedure, and medical social or family history characteristic. Each LLT is linked to only one PT. Each PT has at least one LLT (itself) as well as synonyms and lexical variants (e.g., abbreviations, different word order). Related PTs are grouped together into "High Level Terms" (HLTs) based upon anatomy, pathology, physiology, aetiology or function. HLTs, related to each other by anatomy, pathology, physiology, aetiology or function, are in turn linked to "High Level Group Terms" (HLGTs). Finally, HLGTs are grouped into "System Organ Classes" (SOCs) which are groupings by aetiology (e.g., Infections and infestations), manifestation site (e.g., Gastrointestinal disorders) or purpose (e.g., Surgical and medical procedures)..." MedDRA Code Examples: Code for osteonecrosis: 10031264 Code for osteoRADIOnecrosis: 10067352 Code for medication-related osteonecrosis of jaw: 10084881 Key takeaway points: ORN is an LLT to 'radiation injury', 'bone disorders NEC (not elsewhere classified)', and 'necrosis and vascular insufficiency' Figure 2: MedDRA Hierarchy for ORN and ON	
	6	[yn_meddra]	Do you agree that the Oral Consortium definition for ORN should be in alignment with the existing MedDRA hierarchical definition?	yesno<br>1 Yes<br>0 No
	7	[desc_snomedct]	SNOMED-CT is a comprehensive standard clinical terminology/ontology with concepts (aka. "a clinical meaning identified by a unique numeric identifier") formally defined by detailed relationships with other concepts. Concepts and relationships, or attributes, are represented in SNOMED-CT via standardized concept diagrams, similar to the one below. For more information on diagram symbol definitions, please refer to this Diagramming Guideline: https://confluence.ihtsdotools.org/download/attachments/29951081/doc_DiagrammingGuideline_Current-en-US_INT_20140131.pdf?api=v2 SNOMED-CT Code Examples: Code for osteonecrosis: 240196003 Code for osteoRADIOnecrosis: 109333005 Code for ORN of mandible: 109716001 Code for ORN of maxilla: 109715002 Key takeaway points: SNOMED-CT allows for 'preferred' or 'acceptable' terms for the same concept such as 'radiation necrosis of bone' and 'osteoradionecrosis'. All share a 'finding site' in a bone structure and an associated morphology (attribute) of radiation injury WITH necrosis The 'causative agent (attribute)' is ionizing radiation and 'due to (attribute)' relationship is to exposure to ionizing radiation. Figure 3: SNOMED-CT Concept Diagrams for ORN of mandible and maxilla	descriptive<br>(Attachment: Fig- SNOMED ORN mandible n maxilla.jpg, Display format: Inline image/PDF)
	8	[yn_snomed]	Do you agree that the Oral Consortium definition for ORN should be in alignment with the existing SNOMED-CT concept definition for ORN?	yesno<br>1 Yes<br>0 No
	9	[desc_icd]	International Classification of Disease - Clinical Modficiation (ICD-CM) ICD diagnostic codes range from 3 to 7 characters with the first character always being an alpha (i.e. letter). Longer codes reflect more specific diagnoses. See Figure 4. ICD-10-CM Code Examples: Code for osteonecrosis: M87.9 Code for osteonecrosis, secondary necrosis (NEC), due to drugs: M87.10 Part of the definition for osteonecrosis (M87) from icd10data.com:Clinical Information A disorder characterized by necrotic changes in the bone tissue due to interruption of blood supply. Most often affecting the	descriptive<br>(Attachment: ICD-ON.JPG, Display format: Inline image/PDF)

medRxiv preprint doi: https://doi.org/10.1101/2024.04.07.24305400; this version posted April 9, 2024. The copyright holder for this preprint (which was not certified by peer review) is the author/funder, who has granted medRxiv a license to display the preprint in perpetuity.
It is made available under a CC-BY 4.0 International license .

			epiphysis of the long bones, the necrotic changes result in the collapse and the destruction of the bone structure. Death of a bone or part of a bone Death of a bone or part of a bone, either atraumatic or posttraumatic. Death of bone tissue caused by loss of blood supply to the bone. Death of bone tissue due to traumatic or nontraumatic causes. Of note, the ICD-10-CM Diagnosis Code 'M27.2' is often used for ORN of the jaw, which broadly captures 'inflammatory conditions of jaws'. Reference: https://icd10cmtool.cdc.gov/?fy=FY2023&query=osteonecrosis Figure 4: ICD-10-CM Index to ON	
	10	[standardrate_icd]	If building an ICD-10-CM code for ORN, do you agree that it should be nested under the M87 code for osteonecrosis, similar to ON secondary to drugs (i.e., M87.xx)?	yesno<br>1 Yes<br>0 No
	11	[desc_nci_on]	Section Header: *Defining (Osteo)Necrosis As necrosis is in several of the above definitions for ORN, consensus on the term 'necrosis' itself is needed.*<br>Attaining consensus on the definition of necrosis itself is important when developing a formal definition for ORN. The National Cancer Institute (NCI) Dictionary of Cancer Terms describes osteoNECROSIS as the following:"A condition in which there is a loss of blood flow to bone tissue, which causes the bone to die. It is most common in the hips, knees, shoulders, and ankles. It may be caused by long-term use of steroid medicines, alcohol abuse, joint injuries, and certain diseases, such as cancer and arthritis. It may also occur at some point in time after cancer treatment that included methotrexate, bisphosphonates, or corticosteroids. Also called aseptic necrosis, avascular necrosis, and ischemic necrosis."Reference: https://www.cancer.gov/publications/dictionaries/cancer-terms/def/osteonecrosis	descriptive
	12	[likert_nci_on]	Do you agree with the NCI's definition for osteonecrosis?	radio<br>1 Strongly agree<br>2 Somewhat agree<br>3 Neutral<br>4 Somewhat disagree<br>5 Strongly disagree
	13	[likert_nci_on_disagree]<br>Show the field ONLY if: [likert_nci_on] = '4' or [likert_nci_on] = '5'	If you disagree, please state why	text
	14	[likert_nec_vasc]	Do you agree with the definition of necrosis requiring loss/impairment of blood flow, or vascular insufficiency, or devascularization to any tissue?	radio<br>1 Strongly agree<br>2 Somewhat agree<br>3 Neutral<br>4 Somewhat disagree<br>5 Strongly disagree
	15	[likert_nec_vasc_disagree]<br>Show the field ONLY if: [likert_nec_vasc] = '4' or [likert_nec_vasc] = '5'	If you disagree, please state why	text
	16	[yn_ebnb]	Are ALL cases of exposed bone also automatically necrotic bone?	yesno<br>1 Yes<br>0 No
	17	[yn_intactmucdx]	Can ORN be diagnosed in cases with intact mucosa (i.e., diagnosis is supported by imaging findings)?	yesno<br>1 Yes

medRxiv preprint doi: https://doi.org/10.1101/2024.04.07.24305400; this version posted April 9, 2024. The copyright holder for this preprint (which was not certified by peer review) is the author/funder, who has granted medRxiv a license to display the preprint in perpetuity.
It is made available under a CC-BY 4.0 International license .

				0 No
	18	[cases_nb]	Disregarding a time feature, how would you classify the following scenarios related to a HN cancer patient treated with RT? For all cases, assume there is no evidence of active cancer in the evaluated site of irradiated bone. Questions are meant to vary on clinical and imaging findings. Please read each scenario closely and answer to your best ability with the information provided. CBCT shows lytic and sclerotic internal texture in mandible; exposed bone on exam. {cases_nb_1} CBCT shows lytic and sclerotic internal texture in mandible; no exposed bone on exam (mucosa intact). {cases_nb_2} CBCT shows presence of sequestrum; you can probe to bone. {cases_nb_3} CBCT shows periosteal reaction; you can probe to bone. {cases_nb_4} CBCT shows periosteal reaction; you cannot probe to bone. {cases_nb_5} Panorex report states "there is a sclerotic bone pattern within the alveolar processes of the maxillae, and slight periodontal ligament space widening involving the imaged maxillary dentition. The appearance is suggestive of changes related to therapeutic radiation exposure. " {cases_nb_6} A patient presents with a radiograph from their family dentist demonstrating bone loss to the apex of teeth 36, 37 in the absence of bone loss elsewhere in the mouth. You cannot probe to bone. {cases_nb_7} A patient presents with an image from their family dentist demonstrating bone loss to the apex of teeth 36, 37 in the absence of bone loss elsewhere in the mouth. You can probe to bone between the roots of the teeth. {cases_nb_8} A patient presents with an image from their family dentist demonstrating bone loss to the apex of teeth 36, 37 with radiographic regions of sclerosis and bone resorption extending to the inferior border of the mandible. There is no exposed bone. {cases_nb_9}	descriptive
	19	[cases_nb_1]	1. CBCT shows lytic and sclerotic internal texture; exposed bone on exam	radio<br>1 Definitely ORN<br>2 Possibly ORN<br>3 Not ORN
	20	[cases_nb_2]	2. CBCT shows lytic and sclerotic internal texture; no exposed bone on exam (mucosa intact)	radio<br>1 Definitely ORN<br>2 Possibly ORN<br>3 Not ORN
	21	[cases_nb_3]	3. CBCT shows presence of sequestrum; you can probe to bone	radio<br>1 Definitely ORN<br>2 Possibly ORN<br>3 Not ORN
	22	[cases_nb_4]	4. CBCT shows periosteal reaction; you can probe to bone	radio<br>1 Definitely ORN<br>2 Possibly ORN<br>3 Not ORN
	23	[cases_nb_5]	5. CBCT shows periosteal reaction; you cannot probe to bone	radio<br>1 Definitely ORN<br>2 Possibly ORN<br>3 Not ORN
	24	[cases_nb_6]	6. Panorex report states "there is a sclerotic bone pattern within the alveolar processes of the maxillae, and slight periodontal ligament space widening involving the imaged maxillary dentition."	radio<br>1 Definitely ORN<br>2 Possibly ORN<br>3 Not ORN

medRxiv preprint doi: https://doi.org/10.1101/2024.04.07.24305400; this version posted April 9, 2024. The copyright holder for this preprint (which was not certified by peer review) is the author/funder, who has granted medRxiv a license to display the preprint in perpetuity.
It is made available under a CC-BY 4.0 International license .

	25	[cases_nb_7]	7. A patient presents with a radiograph from their family dentist demonstrating bone loss to the apex of teeth 36, 37 in the absence of bone loss elsewhere in the mouth. You cannot probe to bone.	radio<br>1 Definitely ORN<br>2 Possibly ORN<br>3 Not ORN
	26	[cases_nb_8]	8. A patient presents with an image from their family dentist demonstrating bone loss to the apex of teeth 36, 37 in the absence of bone loss elsewhere in the mouth. You can probe to bone between the roots of the teeth.	radio<br>1 Definitely ORN<br>2 Possibly ORN<br>3 Not ORN
	27	[cases_nb_9]	9. A patient presents with an image from their family dentist demonstrating bone loss to the apex of teeth 36, 37 with radiographic regions of sclerosis and bone resorption extending to the inferior border of the mandible. There is no exposed bone.	radio<br>1 Definitely ORN<br>2 Possibly ORN<br>3 Not ORN
	28	[yn_more_cases]	Do you wish to provide additional case scenarios for the Consortium to review during Round 3?	yesno<br>1 Yes<br>0 No
	29	[case_scenarios]<br><br>Show the field ONLY if:<br>[yn_more_cases] = '1'	Please elaborate on case scenarios to review.	notes
	30	[yn_ebnb_2]	Repeated Q: Are ALL cases of exposed bone also automatically necrotic bone?	yesno<br>1 Yes<br>0 No
	31	[yn_intactmucdx_2]	Repeated Q: Can ORN be diagnosed in cases with intact mucosa (i.e., diagnosis is supported by imaging findings)?	yesno<br>1 Yes<br>0 No
	32	[desc_time]	Section Header: *The 'Time Feature' and Refining the ORN Definition*<br><br>The duration of ORN, while regarded as a highly important feature, remains controversial (i.e., when to use it and/or how to define useful parameters). Despite its inclusion in 6 of the 14 ORN staging/grading systems reviewed during Round 1, there is no consensus on the explicit definition for the time feature to declare a diagnosis of ORN (present/absent). Round 1 Summary: Harris, Schwartz, and Karagozoglu all reported different 'minimum' time periods of bone exposure in an irradiated field to diagnose ORN. Disagreement with the time feature was also seen in the group's response to Case 1 (1.5 cm of exposed bone for 2 months) whereby only 27 (46%) of members would diagnose the patient with ORN during that clinic visit. The majority (83%) agreed on diagnosing Case 2 with ORN, given a much longer time window (0.5 cm exposed bone for 7 months). When asked to provide an explicit time window (in months) for diagnosing ORN, 47% of panelists left this question blank while the remaining panelists listed 3 months (41%), 4 months (3%), or 6 months (9%). Additional considerations with regards to time: Standardized diagnostic systems (i.e., ICD, MedDRA, SNOMED-CT, etc) do NOT include a time feature in their disease/disorder definitions. The true duration of necrotic bone (seen either clinically and/or on imaging) is difficult to measure as our observations heavily rely on the timing and frequency of patient visits (which can vary among providers). Consider the attached image scenario where clinical exams and imaging are performed every 3 months for a patient treated with RT. The asterisks represent suggested 'minimal duration of exposed bone' time windows after which one can 'diagnose' ORN. The red wording and time window represents changes occurring in between visits. Figure 5: Case Scenario	descriptive<br>(Attachment: TimeScenario.png, Display format: Inline image/PDF)

medRxiv preprint doi: https://doi.org/10.1101/2024.04.07.24305400; this version posted April 9, 2024. The copyright holder for this preprint (which was not certified by peer review) is the author/funder, who has granted medRxiv a license to display the preprint in perpetuity.
It is made available under a CC-BY 4.0 International license .

	33	[time_case]	If the patient informs you during Visit #2 that he noticed exposed bone 2 months prior to that visit, would you:	radio<br>1 Diagnose ORN during visit 2<br>2 Wait to diagnose ORN during visit 3<br>3 Wait to diagnose ORN during visit 4<br>4 None of the above
	34	[time_explain]<br>Show the field ONLY if:<br>[time_case] = '4'	Please explain	text
	35	[desc_timedx]	With the information and group feedback provided above, please rate your level of agreement with the following statements: The diagnostic criteria for a bone-based disorder like ORN can be met without the inclusion of a time feature. {time_q_1} A consensus-based staging system for ORN severity can be developed without the mandatory inclusion of a time feature (i.e., time features may be supplemental but not mandatory for describing ORN severity). {time_q_2} A time feature would be useful for assessing response to therapy. Response to therapy should be separated from a staging system for the disease entity, ORN. {time_q_3}	descriptive
	36	[time_q_1]	The diagnostic criteria for a bone-based disorder like ORN can be met without the inclusion of a time feature.	radio<br>1 Strongly agree<br>2 Somewhat agree<br>3 Neutral<br>4 Somewhat disagree<br>5 Strongly disagree
	37	[time_comment_1]<br>Show the field ONLY if:<br>[time_q_1] = '4' or [time_q_1] = '5'	Please comment on the question above, if possible.	text
	38	[time_q_2]	A consensus-based staging system for ORN severity should be developed without the mandatory inclusion of a time feature (i.e., time features may be supplemental but not mandatory for describing ORN severity).	radio<br>1 Strongly agree<br>2 Somewhat agree<br>3 Neutral<br>4 Somewhat disagree<br>5 Strongly disagree
	39	[time_comment_2]<br>Show the field ONLY if:<br>[time_q_2] = '4' or [time_q_2] = '5'	Please comment on the question above, if possible.	text
	40	[time_q_3]	A time feature would be useful for assessing response to therapy. Response to therapy should be separated from a staging system for the disease entity, ORN.	radio<br>1 Strongly agree<br>2 Somewhat agree<br>3 Neutral<br>4 Somewhat disagree<br>5 Strongly disagree
	41	[time_comment_3]<br>Show the field ONLY if:<br>[time_q_3] = '4' or [time_q_3] = '5'	Please comment on the question above, if possible.	text
	42	[vote_deforn]	Based on NCI's definition for osteonecrosis and existing diagnostic standards, please select which statement BEST defines ORN. For reference again from NCI: "A condition in which there is a loss of blood flow to bone tissue, which causes the bone to die. It is most common in the hips, knees, shoulders, and ankles. It may be caused by long-	radio<br>1 A condition in which there is a loss of blood flow to bone tissue, which causes the bone to die. It is caused by exposure to ionizing radiation.

medRxiv preprint doi: https://doi.org/10.1101/2024.04.07.24305400; this version posted April 9, 2024. The copyright holder for this preprint (which was not certified by peer review) is the author/funder, who has granted medRxiv a license to display the preprint in perpetuity.
It is made available under a CC-BY 4.0 International license .

			term use of steroid medicines, alcohol abuse, joint injuries, and certain diseases, such as cancer and arthritis. It may also occur at some point in time after cancer treatment that included methotrexate, bisphosphonates, or corticosteroids. Also called aseptic necrosis, avascular necrosis, and ischemic necrosis."	2 A condition in which there is a loss of blood flow to bone tissue, which causes the bone to die. It is caused by exposure to ionizing radiation and may occur at some point in time after radiation.<br>3 A condition in which there is a loss of blood flow to bone tissue, which causes the bone to die. It is caused by exposure to ionizing radiation and occurs in the absence of active disease (i.e., cancer) in the site of bone death.<br>4 A condition in which there is a loss of blood flow to bone tissue, which causes the bone to die. It is caused by exposure to ionizing radiation and may occur at some point in time after radiation and in the absence of active disease (i.e., cancer) in the site of bone death.
	43	[comments_orndeffinal]	Any additional comments on the diagnostic criteria for ORN (not staging/grading)?	notes
	44	[desc_use_rate_systems]	Section Header: *Staging Elements for Reporting Extent and Severity of ORN*<br>During Round 1, a total of 15 staging/grading systems were reviewed. Members were asked to 1) state personal use of each system, 2) rate the utility of the system, and 3) apply the system to categorizing 3 different case scenarios. Personal use: The top 3 systems used in practice were: CTCAE (n=41; 70%), Notani (n=18, 32%), and Marx (n=18, 31%). Rating of effectiveness for classifying ORN: The top-rated systems, defined as 'somewhat/very important', for ORN were not in the top ones for personal use and included: Shwartz & Kagan (n=28, 52%), Karagozoglu (n=24, 49%), and Morton & Simpson (n=26, 48%). CTCAE, Notani, and Marx were considered effective by 46%, 42%, and 23% of respondants, repsectively. Figure 6: Personal Use and Effectiveness Rating of Existing Staging/Grading Systems	descriptive (Attachment: SystemUsenRating.jpg, Display format: Inline image/PDF)
	45	[desc_cases]	Group feedback continued:During Round 1, members were asked to categorize the following 3 cases using each staging/grading system. Case 1: Patient with exposed bone (no measurement) not involving lower mandible, unknown duration. Pain present. Case 2: Patient with 1.2 cm exposed bone for 4 months, pain present. Case 3: Patient with 3 cm exposed bone with pathologic fracture. Unknown symptoms or duration. Fom a total of 59 responders: The inability to classify cases was 57% for Case 1, 45% for Case 2, and 26% for Case 3. Pathological fracture (a finding in Case 3) was commonly classified as advanced ORN (major or stage III). Missing responses (i.e., no option was selected) was low at 3-4%. The distribution of case classification per system is shown in Figure 7. Overall, none of the existing systems were rated useful by more than 70% of the responders (the consensus threshold; top rated one was 52%), and the inconsistent classification of cases using these systems demonstrates an ongoing need for ORN data standardization. Please click on the link below for Figure 7.	descriptive (Attachment: Round1CasesbySystem.png, Display format: Link)
	46	[desc_addsystems]	Additional grading systems recommended for review during Round 1:In the comments section, four additional osteonecrosis / ORN systems were recommended for review. They include: MRONJ (Medication-Related Osteonecrosis of the Jaws) LENT SOMA Scale Princess Margaret Cancer Center ORN Scoring System RTOG CTC (Common Toxicity Criteria) Summary: MRONJ defines a non-exposed bone variant (stage 0), explicitly states 'exposed and necrotic bone or fistula' for advanced stages, and incorporates imaging findings. LENT-SOMA uses extent of exposed bone (2cm) or presence of limited sequestration or fracture for upgrading PMH defines grades as loss of mucosa with exposed bone requiring particular therapies for increasing time. Pathologic bone fracture is a grade 4. RTOG CTC: Vague and symptom	descriptive (Attachment: ADDITIONAL GRADING SYSTEMS FOR REVIEW.docx, Display format: Link)

medRxiv preprint doi: https://doi.org/10.1101/2024.04.07.24305400; this version posted April 9, 2024. The copyright holder for this preprint (which was not certified by peer review) is the author/funder, who has granted medRxiv a license to display the preprint in perpetuity.
It is made available under a CC-BY 4.0 International license .

			based. In alignment with CTCAE Please review them in the attached file.	
	47	[use_4systems]	Have you used any of the osteonecrosis/ ORN systems listed?	checkbox<br>1 use_4systems___1 MRONJ (Medication-Related Osteonecrosis of the Jaws)<br>2 use_4systems___2 LENT SOMA Scale<br>3 use_4systems___3 Princess Margaret Cancer Center ORN Scoring System<br>4 use_4systems___4 RTOG CTC (Common Toxicity Criteria)
	48	[use_4systems_efficacy]	Which of these systems, if any, do you find highly effective for staging ORN? Please select all that apply.	checkbox<br>1 use_4systems_efficacy___1 MRONJ (Medication-Related Osteonecrosis of the Jaws)<br>2 use_4systems_efficacy___2 LENT SOMA Scale<br>3 use_4systems_efficacy___3 Princess Margaret Cancer Center ORN Scoring System<br>4 use_4systems_efficacy___4 RTOG CTC (Common Toxicity Criteria)
	49	[desc_ctcae]	Clarification on the widely used Common Terminology Criteria for Adverse Events (CTCAE)CTCAE is often used by the group (70%) but it is unclear if this is for staging (reporting ORN extent/severity) and/or toxicity grading (AEs after therapy). For reference, the NCI states the following about CTCAE: it is a "descriptive terminology which can be utilized for Adverse Event (AE) reporting. A grading (severity) scale is provided for each AE term. An Adverse Event (AE) is any unfavorable and unintended sign (including an abnormal laboratory finding), symptom, or disease temporarily associated with the use of a medical treatment or procedure that may or may not be considered related to the medical treatment or procedure. An AE is a term that is a unique representation of a specific event used for medical documentation and scientific analyses." Reference: https://ctep.cancer.gov/protocoldevelopment/electronic_applications/docs/ctcae_v5_quick_reference_5x7.pdf	descriptive
	50	[desc_ctcaeq]	Given the CTCAE description, do you agree with the following statements? CTCAE is a treatment-related toxicity (not disease) grading system {ctcae_1} CTCAE can be used to stage ORN {ctcae_2} CTCAE can be used in parallel with an ORN staging system for medical documentation after use of medical treatments or procedures {ctcae_3}	descriptive
	51	[ctcae_1]	1. CTCAE is a toxicity (not disease) grading system.	yesno<br>1 Yes<br>0 No
	52	[ctcae_2]	2. CTCAE can be used to stage ORN	yesno<br>1 Yes<br>0 No
	53	[ctcae_3]	3. CTCAE can be used in parallel with an ORN staging system for medical documentation after use of medical treatments or procedures.	yesno<br>1 Yes<br>0 No
	54	[desc_stagingelements]	Rating of Data Elements in Existing ORN Staging/Grading Systems During Round 1, the group was asked to rate the level of importance for each element extracted from all reviewed staging and grading systems. The results are shown in Figure 8. The only elements (n=3) that were rated as 'somewhat/very important' by 100% of the group	descriptive<br>(Attachment: RatingFxofORN.jpeg, Display format: Inline image/PDF)

medRxiv preprint doi: https://doi.org/10.1101/2024.04.07.24305400; this version posted April 9, 2024. The copyright holder for this preprint (which was not certified by peer review) is the author/funder, who has granted medRxiv a license to display the preprint in perpetuity.
It is made available under a CC-BY 4.0 International license .

			were all bone-related and included pathological fracture, exposed bone, and extent of exposed bone. Elements found to be of least importance when staging ORN included treatment (HBO, surgery, conservative therapy) and response to therapy (with the exception of persistent bone exposure). Comments from members: I think treatment can be considered separate from the classification as that may depend on what the patient or health care provider feels most comfortable with. A staging system based on primarily clinical factors is more useful, can consider including need for antibiotic treatment. If including response to therapy, would consider response to vitamin E/Trental as that defines conservative management vs surgical management. The classification should be independent on the response to treatment. There is limited evidence on the ideal treatment for ORN. It is therefore generally not appropriate to include these in a classification. The descriptors of extent of ORN should be sufficiently broad to be applicable to a wide range of cases (including 'pre-ORN') whilst also evaluating progression. Applying just to the mandible also limits external validity for ORN affecting maxilla or free flap. Figure 8	
	55	[desc_clarification]	Element Clarification If there is evidence of infection, then: {clarification_1} Since symptoms are not objective findings on clinical exam or imaging, they can be optional modifiers but not necessary factors for staging the extent/severity of ORN. {clarification_2} Since time is not an objective finding on clinical exam or imaging, it can be an optional modifier but not a necessary factor for staging the extent/severity of ORN. Note: this question relates to staging ORN, not diagnosing ORN which we previously covered. {clarification_3}	descriptive
	56	[clarification_1]	If there is e/o infection, then:	radio<br>1 It can still be ORN, but infection is NOT an upstaging factor<br>2 It can still be ORN, but infection makes it a higher grade/severity<br>3 It is NOT ORN, it is osteomyelitis (at least until the infection clears)
	57	[clarification_2]	Since symptoms are not objective findings on clinical exam or imaging, they can be optional modifiers but not necessary factors for staging the extent/severity of ORN. Of note, symptoms are included in toxicity grading scales like CTCAE.	radio<br>4 Strongly agree<br>5 Somewhat agree<br>6 Neutral<br>7 Somewhat disagree<br>8 Strongly disagree
	58	[clarification_3]	Since time is not an objective finding on clinical exam or imaging, it can be an optional modifier but not a necessary factor for staging the extent/severity of ORN. Note: this question relates to staging ORN, not diagnosing ORN which we previously covered.	radio<br>4 Strongly agree<br>5 Somewhat agree<br>6 Neutral<br>7 Somewhat disagree<br>8 Strongly disagree
	59	[desc_elementreview]	Going through the objective elements rated within existing staging/grading systems, please state whether each most often represents early/limited ORN, advanced ORN, or neither (the latter suggesting that they should not be upstaging factors for ORN). Please leave a comment if desired for each. Finding (based on exam and/or imaging) Staging Influence Comments Pathologic fracture {elements_1} {elementcomment_1} Exposed and necrotic bone extent- limited to alveolar bone {elements_2} {elementcomment_2} Exposed and necrotic bone extent- beyond alveolar bone {elements_3} {elementcomment_3}	descriptive

medRxiv preprint doi: https://doi.org/10.1101/2024.04.07.24305400; this version posted April 9, 2024. The copyright holder for this preprint (which was not certified by peer review) is the author/funder, who has granted medRxiv a license to display the preprint in perpetuity.
It is made available under a CC-BY 4.0 International license .

			Non-exposed bone but imaging findings limited to alveolar bone {elements_4} {elementcomment_4} Non-exposed bone but imaging findings beyond alveolar bone {elements_5} {elementcomment_5} Orocutaneous fistula {elements_6} {elementcomment_6} Signs of Infection {elements_7} {elementcomment_7} Mucosal ulceration {elements_8} {elementcomment_8} Sinus formation {elements_9} {elementcomment_9} Sequestra {elements_10} {elementcomment_10} Bone spicules {elements_11} {elementcomment_11}	
	60	[elements_1]	Q1	radio<br>1 Not needed for ORN staging<br>2 Early/limited ORN<br>3 Advanced ORN
	61	[elements_2]	Q2	radio<br>1 Not needed for ORN staging<br>2 Early/limited ORN<br>3 Advanced ORN
	62	[elements_3]	Q3	radio<br>1 Not needed for ORN staging<br>2 Early/limited ORN<br>3 Advanced ORN
	63	[elements_4]	Q4	radio<br>1 Not needed for ORN staging<br>2 Early/limited ORN<br>3 Advanced ORN
	64	[elements_5]	Q5	radio<br>1 Not needed for ORN staging<br>2 Early/limited ORN<br>3 Advanced ORN
	65	[elements_6]	Q6	radio<br>1 Not needed for ORN staging<br>2 Early/limited ORN<br>3 Advanced ORN
	66	[elements_7]	Q7	radio<br>1 Not needed for ORN staging<br>2 Early/limited ORN<br>3 Advanced ORN
	67	[elements_8]	Q8	radio<br>1 Not needed for ORN staging<br>2 Early/limited ORN<br>3 Advanced ORN
	68	[elements_9]	Q9	radio<br>1 Not needed for ORN staging<br>2 Early/limited ORN<br>3 Advanced ORN
	69	[elements_10]	Q10	radio<br>1 Not needed for ORN staging<br>2 Early/limited ORN<br>3 Advanced ORN
	70	[elements_11]	Q11	radio

medRxiv preprint doi: https://doi.org/10.1101/2024.04.07.24305400; this version posted April 9, 2024. The copyright holder for this preprint (which was not certified by peer review) is the author/funder, who has granted medRxiv a license to display the preprint in perpetuity.
It is made available under a CC-BY 4.0 International license .

				1 Not needed for ORN staging<br>2 Early/limited ORN<br>3 Advanced ORN
	71	[elementcomment_1]	Comments	text
	72	[elementcomment_2]	Comments	text
	73	[elementcomment_3]	Comments	text
	74	[elementcomment_4]	Comments	text
	75	[elementcomment_5]	Comments	text
	76	[elementcomment_6]	Comments	text
	77	[elementcomment_7]	Comments	text
	78	[elementcomment_8]	Comments	text
	79	[elementcomment_9]	Comments	text
	80	[elementcomment_10]	Comments	text
	81	[elementcomment_11]	Comments	text
	82	[horizeb_yn]	Extent of necrotic bone exposure revisited Several systems include a vertical extent of disease (i.e., MRONJ, Notani) for staging and usually includes ORN limited to or extending beyond the alveolar bone/canal. Should the extent of horizontal/superficial exposed & necrotic bone be considered and reported in ORN staging?	yesno<br>1 Yes<br>0 No
	83	[horizontbone_comments]<br>Show the field ONLY if:<br>[horizeb_yn] = '1'	Please comment on how to report horizontal/superficial extent of exposed & necrotic bone	text
	84	[comment_eb]	Other considerations on how to stage ORN based on exposed & necrotic bone extent? Please provide thresholds if possible, and include imaging type if relevant.	notes
	85	[add_smoking]	Section Header: *RADMAP: Radiation Dose Mapping to an Odontogram A total of 55 panelists of the Consortium provided a response to the RADMAP (Radiation Odontogram) Section of Round 1. Consensus was defined as 70% of more agreement and percentages are based on the total count of responses per question. The following table is a breakdown of the dental specialists within the oral consortium. For reference to Round 1, the panel reviewed the following figure of a potential RADMAP report which displayed patient, cancer, and treatment information as well as the radiation odontogram and a snapshot of the RT plan. Findings The currentlayout for patient, cancer, and treatment is clear according to 85% (n=44) of the group. After reviewing comments left in Round 1, panelists pointed out a need to include chemotherapy history for the treatment information portion as well as any teeth missing or extracted on the RADMAP visual for each patient. In addition, panelists also made note of general material to add onto the patient information section.*<br>If available, do you agree with adding smoking history/status to the patient information section of the RADMAP report?	yesno<br>1 Yes<br>0 No
	86	[add_gender]	If available, do you agree with adding gender to the patient information section to the RADMAP report?	yesno<br>1 Yes<br>0 No
	87	[add_cancerplan]	If available, do you agree wtih adding chemotherapy to the treatment information section of the RADMAP report?	yesno<br>1 Yes<br>0 No
	88	[add_teeth]	If available, do you agree with adding missing or extracted teeth to the RADMAP visual for each patient?	yesno<br>1 Yes<br>0 No
	89	[practice_teethdata]	In your institution/practice, how easy is it for you to acquire data on missing teeth (i.e., tooth number)?	radio<br>1 Very easy<br>2 Somewhat easy

medRxiv preprint doi: https://doi.org/10.1101/2024.04.07.24305400; this version posted April 9, 2024. The copyright holder for this preprint (which was not certified by peer review) is the author/funder, who has granted medRxiv a license to display the preprint in perpetuity.
It is made available under a CC-BY 4.0 International license .

				3 Neutral<br>4 Somewhat difficult<br>5 Very difficult
	90	[dose_result]	Radiation Dose Heat Maps Group Feedback from Round 1: 89% (n=48) of the group agreed that a heat map over an odontogram (i.e., radiation odontogram) is a clinically useful visualization. Action: We will proceed with the build of a radiation odontogram For cases with missing teeth, 96% (n=50) agreed that visualizing dose data to segmented/ tooth-bearing regions of the mandible and/or maxilla is helpful. When faxing or printing in only in black and white, 70% (n=38) agreed that grayscale colors may impede providers from accurately interpreting radiation dose distribution. Action: We will provide an accompanying table with dose data. 80% (n=43) of the group responded that they are very confident or somewhat confident in their ability to interpret radiation doses on the radiation odontogram. The estimated mean and median dose delivered to tooth #31 was 14 Gy and 13 Gy, respectively (acceptable with one outlier of dmax ~35Gy). Action: No need for additional educational tools with RADMAP report	descriptive
	91	[teeth_visual]	When comparing options A and B, 59% preferred option A whereas 32% (n=17) chose option B. A minority of 9% (n=5) opted for neither option stating that dose data to each tooth can be presented in the format of a table. Action: We will proceed with Option A as the background of the radiation odontogram.	descriptive<br>(Attachment: Screen Shot 2023-05-08 at 10.53.44 PM.png, Display format: Inline image/PDF)
	92	[comprehension8_result]	Evaluating dose data from an RT plan snapshot 96% (n=51) of the group reported seeing this type of radiation treatment plan snapshot before 72% (n=39) routinely request or review radiation therapy plans for evaluating post-RT care. When asked to evaluate the radiation dose delivered to tooth #8 (labeled A on image above), the mean, median and mode were 1000 cGy, 1000 cGy, 2000 cGy. Reported range: 0 to 2200 cGy.	descriptive
	93	[idl_read]	Based on the findings above, how strongly do you agree with the following statement?RT isodose lines on a treatment plan are potentially not as easy to interpret as a radiation odontogram heatmap/ table.	radio<br>1 Strongly Agree<br>2 Somewhat Agree<br>3 Neutral<br>4 Somewhat Disagree<br>5 Strongly Disagree
	94	[heatmap_add]	77% (n=41) of the group agreed that a 'heat map' of radiation dose on a table is useful. If we were to apply a heat map, which parameter do you agree with incorporating? Please select all that apply.	checkbox<br>1 heatmap_add___1 Use a single-hue color palette of varying saturation<br>2 heatmap_add___2 Use a diverging color palette<br>3 heatmap_add___3 Use a different color than red<br>4 heatmap_add___4 Other<br>Custom alignment: LV
	95	[comments_heatmap]<br>Show the field ONLY if:<br>[heatmap_add(4)] = '1'	Please comment on question above	text<br>Custom alignment: LV
	96	[parameters]	In terms of presented dental dose data, dmean and dmax were ranked the highest in preference by 56% of the group (total). See the breakdown below. Dosimetric Parameter Count (%) Mean dose 30 (31%) Max point dose 24 (25%) Dose going to 0.03cc (D0.033cc) 17 (18%) Dose going to 95% of the tooth (D95) 15 (16%) Dose going to 50% of the tooth (D50) 10 (10%) If only given one option, which metric is desired the MOST?	radio<br>1 Mean dose (dmean) to each tooth/ tooth-bearing region<br>2 Max dose (dmax) to each tooth / tooth-bearing region<br>Custom alignment: LV

medRxiv preprint doi: https://doi.org/10.1101/2024.04.07.24305400; this version posted April 9, 2024. The copyright holder for this preprint (which was not certified by peer review) is the author/funder, who has granted medRxiv a license to display the preprint in perpetuity.
It is made available under a CC-BY 4.0 International license .

	97	[round2_lastquestions]	Any last questions or comments for Round 2?	descriptive
	98	[round_2_orn_complete]	Section Header: *Form Status*<br>Complete?	dropdown<br>0 Incomplete<br>1 Unverified<br>2 Complete

Instrument: Round 1 Introduction And Panel Info (round_1_introduction_and_panel_info) **Enabled as survey**

	99	[official_consent]	Section Header: *Welcome to the Orodental ontologies for reporting Radiotherapy-induced Adverse sequeLae (ORAL) Consortium Thank you all for expressing interest in joining the ORAL Consortium! As noted in the email invitation, the primary goals of this initial work are to 1) develop expert-based orodental data standardization guidelines, and 2) provide input on designs for 3D-to-2D visualizations of doses to teeth (aka 'radiation odontogram' [RADMAP]). These efforts aim to facilitate scalable and comprehensive information sharing among multidisciplinary providers managing patients with head and neck cancers (HNC). The ORAL Consortium includes international representatives from Radiation Oncology, Head & Neck Surgery, Oral Oncology or Medicine, Dentistry, Radiation Physics, and Symptom Research. A bit of my background: I'm one of the Head & Neck Radiation Oncologists at MD Anderson Cancer Center in Houston, TX. I am also an informatician with NIDCR funded projects focused on defining machine- and human-readable ontologies to relate dental dose from radiotherapy (RT) and treatment-associated HN toxicities, such as osteoradionecrosis (ORN). ORN is known to be a severe iatrogenic disease that is experienced by 5-20% of HNC survivors. Fundamental understanding of the natural history and mechanistic progression of ORN remains a significant under-explored domain due to heterogeneous definitions of the true disease state and numerous staging/grading systems which can lead to under-reporting or misclassification of ORN severity. Ontologies are "formal, explicit specifications of a shared conceptualizaton." In other words, they are machine-readable (formal), agreed upon by a group (shared), and an abstract model of a particular field of knowledge (conceptualization). Building an ontology for ORN would carry significant clinical and research advantages, including the ability to relateexisting ORN staging/grading systems (i.e., a Notani stage X = Lyons stage Y). What should you expect? Using a remote, modified Delphi technique (i.e., iterative surveys for consensus formation), we will evaluate existing ORN definitions and scales for extraction and explicit definition of classes and relations that are essential to build an ORN ontology. A total of 3 to 4 "rounds" of surveys are expected to achieve Consortium consensus. Each survey is expected to take 15-20 minutes to complete and can be completed at your own pace within a 2-week timeframe from initial survey release. Time between each survey once completed is about 2-to-3 weeks to allow for data analysis and generation of the next survey. Should you need a break while working on a survey while it's still active, you can return to where you left off by clicking on the same survey link from the email with the "round X" invite. Friendly email reminders will be sent automatically every 4 days for up to 3 times to participants who have not completed the survey.Surveys will be automatically closed at 11:59 PM (CST) on the 14th day of survey release. Rounds and Analysis: This is Round 1. Round 1 includes three main sections: 1. Consortium Member Information and Acknowledgements 2. Review of Definitions and Classifications of ORN 3. Review of Radiation Ondontogram (RADMAP) draft designs Subseqent rounds will include annonymized group feedback and statistics as well as further consolidation of questions for appraching consensus. Consensus statements in later Rounds will be "confirmed" by an agreement of 70% or more of Consortium members. Thank you again for your willingness to serve as an expert!*<br>CONSENT FOR PARTICIPATION IN THIS DELPHI I have read the description of the study above, and I have decided to partcipate in the research project described. I understand that my continued participation throughout the entire Delphi study is crucial for robust analysis and consensus formation. I understand that my responses to survey questions will be collected via REDCap where data will be stored in a password-protected electronic format with access only available to Dr. Amy Moreno and her research team. My answers will remain anonymous to the entire expert panel and on future reports of this study. I also understand that I may refuse to answer any (or all) of the questions at this time or any other time. Should I wish to withdraw at any time or have my personal information removed from future publications, I can email Dr. Moreno with my specific request(s) at akmoreno@mdanderson.org By clicking on the "Yes" button below, I certify that I have read the above information and voluntarily agree to participate in this ORAL Consortium study as an Expert.	yesno, Required<br>1 Yes<br>0 No<br><br>Custom alignment: LV<br>Stop actions on 0

medRxiv preprint doi: https://doi.org/10.1101/2024.04.07.24305400; this version posted April 9, 2024. The copyright holder for this preprint (which was not certified by peer review) is the author/funder, who has granted medRxiv a license to display the preprint in perpetuity.
It is made available under a CC-BY 4.0 International license .

	100	[levelofack]	Publications are expected related to this work. Please let us know to what degree you would like to be acknowledged. Please note that in order to be included in the 'ORAL Consortium' group authorship list, you must provide 'substantial contributions' (i.e., complete at least one survey to qualify for interpretation of data).	radio<br>1 No acknowledgments (keep me anonymous in publications and/or presentations)<br>2 I'd like my name to be in the acknowledgement section only<br>3 I'd like to be a co-author (included in the group authorship list)<br>4 Unsure at the moment
	101	[update]	Please express your level of interest in being involved in manuscript preparations.	radio<br>1 I don't want to be involved in manuscript writing or review<br>2 I want to be involved/updated on final manuscript review only<br>3 I want to be involved/updated on initial manuscript drafting and final manuscript review
	102	[age]	Section Header: *Member Information*<br>What is your age?	text (integer, Min: 18, Max: 100)
	103	[sex]	What is your gender?	radio<br>0 Female<br>1 Male<br>2 Nonbinary<br>3 Prefer not to say
	104	[degree]	What degree(s) do you currently have?	checkbox<br>0 degree___0 DDS<br>1 degree___1 DMD<br>2 degree___2 MD<br>3 degree___3 DO<br>5 degree___5 PhD<br>4 degree___4 Other
	105	[employer]	Select the option that best describes your practice setting. Check all that apply	checkbox<br>0 employer___0 Academic Medical Center (involved in graduate/medical education and/or research)<br>1 employer___1 Nonacademic Hospital<br>5 employer___5 Government-affiliated<br>2 employer___2 Independent/Private Practice<br>3 employer___3 Locum tenens<br>4 employer___4 Other
	106	[other_employer]<br>Show the field ONLY if: [specialty(6)] = '1'	If other, please describe	notes
	107	[practice]	Please write the name of your affiliated institution/practice as you desire it to be shown in publications. (i.e., The University of Texas MD Anderson Cancer Center)	text
	108	[department]	Please write your affiliated department (or specialty) (i.e., Radiation Oncology)	text
	109	[specialty]	For those in oral medicine, oral surgery, or dentistry, how would you best describe your specialty? Select all that apply	checkbox<br>0 specialty___0 General Dentist<br>1 specialty___1 Periodontist<br>2 specialty___2 Endodontist<br>3 specialty___3 Prosthodontist

medRxiv preprint doi: https://doi.org/10.1101/2024.04.07.24305400; this version posted April 9, 2024. The copyright holder for this preprint (which was not certified by peer review) is the author/funder, who has granted medRxiv a license to display the preprint in perpetuity.
It is made available under a CC-BY 4.0 International license .

				4 specialty___4 Oral and Maxillofacial Surgeon (OMFS)<br>5 specialty___5 Oral Medicine<br>6 specialty___6 Other
	110	[other_specialty]<br>Show the field ONLY if:<br>[specialty(6)] = '1'	If other, please describe	notes
	111	[advanced_edu]	Have you completed any advanced education not described above?	radio<br>1 General Practice Residency (GPR)<br>2 Advanced Education in General Dentistry (AEGD)<br>3 Other<br>4 None
	112	[other_advanced_edu]<br>Show the field ONLY if:<br>[specialty(6)] = '1'	If other, please describe	notes
	113	[country]	What country do you work in?	text
	114	[community]	Select the option that best describes the community in which you work	radio<br>3 Urban (>75,000 population)<br>4 Suburban (10,000-75,000)<br>5 Rural (< 10,000)
	115	[years_practice]	Approximate years in clinical practice following completion of training	text (number, Min: 0)
	116	[caseload]	In your practice, approximately how many head and neck cancer patients do you evaluate and/or manage monthly (symptom management included)?	text (integer)
	117	[roles]	In your routine practice, what roles do you perform for patients receiving head and neck radiation therapy? Please select all that apply	checkbox<br>0 roles___0 PRE-radiation dental evaluations<br>2 roles___2 Perform pRE-radiation invasive interventions (dental extraction, oral surgery, etc.)<br>3 roles___3 Design fluoride trays or stents<br>4 roles___4 POST-radiation dental evaluations<br>5 roles___5 Perform POST-radiation invasive interventions (dental extraction, oral surgery, etc.)<br>6 roles___6 Perform HN surgeries when indicated<br>7 roles___7 Plan and oversee radiation therapy<br>8 roles___8 QA radiation therapy plans<br>9 roles___9 Other
	118	[roles_other]<br>Show the field ONLY if:<br>[roles(9)] = '1'	If other, please describe	text
	119	[round_1_introduction_and_panel_info_complete]	Section Header: *Form Status*<br>Complete?	dropdown<br>0 Incomplete<br>1 Unverified<br>2 Complete

Instrument: Round 1 Defining And Classifying ORN (round_1_defining_and_classifying_orn) Enabled as survey

	120	[patient_orn]	Section Header: *SECTION 1: Defining and Classifying Osteoradionecrosis (ORN) This section focuses on review of existing definitions and staging/grading systems for ORN.Sample cases of ORN are classified by system. Data elements within staging/grading systems are extracted for review and rating. The utility of imaging in diagnosing ORN is also reviewed.*	yesno<br>1 Yes<br>0 No

medRxiv preprint doi: https://doi.org/10.1101/2024.04.07.24305400; this version posted April 9, 2024. The copyright holder for this preprint (which was not certified by peer review) is the author/funder, who has granted medRxiv a license to display the preprint in perpetuity.
It is made available under a CC-BY 4.0 International license .

			Have you ever evaluated and/or treated a patient with ORN?	
	121	[evaluated_orn]<br><br>Show the field ONLY if:<br>[patient_orn] = '1'	On average, how many patients have you evaluated per year?	text (integer)
	122	[treated_orn]<br><br>Show the field ONLY if:<br>[patient_orn] = '1'	On average, how many patients have you treated per year? Treatments include conservative medical management (i.e., Vitamin E), HBO therapy, debridement, or major surgery. Please enter zero if you do not treat ORN.	text (integer, Min: 0)
	123	[percentage_orn]<br><br>Show the field ONLY if:<br>[patient_orn] = '1'	As an estimate, what percentage of your HNC patients treated with RT have had ORN? Please enter a number between 0-100.	text (integer, Min: 0, Max: 100)
	124	[table1_orndefinitions]	Section Header: *Definitions for ORN*<br><br>Example Definitions for ORN: While variations on extent of ORN, symptoms, and management are included in most staging systems, explicit definitions of ORN are not consistent (or may be absent). Please review the following published definitions for ORN. Time features (i.e., persistence of ORN) are bolded if present. Authors (Year) Diagnostic Criteria Marx (1983) An area greater than 1 cm of exposed bone in a field of irradiation that has failed to show and evidence of healing for at least 6 months Beumer (1983) Exposure of bone of the maxilla or mandible within the radiation treatment volume persisting for more than 3 months Marx and Johnson (1987) Exposure of nonviable bone which fails to heal without intervention Epstein (1987) An ulceration or necrosis of the mucous membrane, with exposure of necrotic bone for more than 3 months Widmark (1989) A non-healing mucous or cutaneous ulcer with denuded bone, lasting for more than 3 months Harris (1992) Exposed irradiated bone that has failed to heal over a period of 3 months in the absence of local tumor Wong (1997) A slow-healing radiation-induced ischemic necrosis of bone with associated soft tissue necrosis of variable extent occurring in the absence of local primary tumor necrosis, recurrence, or metastatic disease Schwartz (2002) A condition in which devitalized, irradiated bone becomes exposed through a wound in the overlying skin or mucosa. No tumor recurrence and it must persist for 3 to 6 months Karagozoglu (2014) Radiation induced necrosis of bone. Exposed bone with or without changes on plain radiograph having excluded presence of tumor tissue. Definitive ORN diagnosis if exposed bone persists for at least 1 month	descriptive
	125	[orndef_pref]	Out of the definitions for ORN, which one do you feel is most representative for diagnosing ORN?	radio<br>1 Marx<br>2 Beumer<br>3 Marx and Johnson<br>4 Epstein<br>5 Widmark<br>6 Harris<br>7 Wong<br>8 Schwartz<br>9 Karagozoglu
	126	[define_orn_ebone]	Please state your level of agreement with the following statement: "Exposed bone" should be required for the diagnosis of ORN (i.e., radiological evidence with intact mucosa is not ORN)	radio<br>1 Strongly Agree<br>2 Somewhat Agree<br>3 Neutral<br>4 Somewhat Disagree

medRxiv preprint doi: https://doi.org/10.1101/2024.04.07.24305400; this version posted April 9, 2024. The copyright holder for this preprint (which was not certified by peer review) is the author/funder, who has granted medRxiv a license to display the preprint in perpetuity.
It is made available under a CC-BY 4.0 International license .

				5 Strongly Disagree
	127	[numeric_exposedbone_orn]	The minimum length of exposed bone to qualify as ORN is explicity stated in Marx's definition (> 1cm). Do you agree that a formal definition for ORN should incorporate a specific numerical threshhold measurement of exposed bone?	radio<br>1 Strongly Agree<br>2 Somewhat Agree<br>3 Neutral<br>4 Somewhat Disagree<br>5 Strongly Disagree
	128	[define_orn_ebone_2]	Please state your level of agreement with the following statement: A time feature should be required for the diagnosis of ORN (i.e., persistence of exposed bone reported in months)	radio<br>1 Strongly Agree<br>2 Somewhat Agree<br>3 Neutral<br>4 Somewhat Disagree<br>5 Strongly Disagree
	129	[dx_orn_case1]	Example case 1: A patient in clinic is noted to have 1.5 cm of exposed bone 2 months after HN radiotherapy. Do you diagnose this patient with ORN during this visit?	yesno<br>1 Yes<br>0 No
	130	[dx_orn_timewindow]<br>Show the field ONLY if:<br>[dx_orn_case1] = '0'	For this case, what is the minimum amount of time (in months) from end of RT that the patient must have exposed bone to be considered as ORN?	text (integer, Min: 0)
	131	[dx_orn_case2]	Example case 2: A patient in clinic is noted to have 0.5 cm of exposed bone 7 months after HN radiotherapy. Do you diagnose this patient with ORN during this visit?	yesno<br>1 Yes<br>0 No
	132	[matrix_def]	For each element below, please rate how important you think it is to use it in the formal definition of ORN. Mucosal ulceration {dxorn_ulc} Exposed bone {dxorn_expbone} Exposed bone measurement (in mm) {dxorn_expbonemeasure} Duration of exposure (i.e., weeks, months) {dxorn_duration} Radiologic findings (state presence or absence) {dxorn_imaging}	descriptive
	133	[dxorn_ulc]	Importance of mucosal ulceration in definition of ORN	radio<br>1 Very important<br>2 Somewhat important<br>3 Not important
	134	[dxorn_expbone]	Importance of exposed bone in definition of ORN	radio<br>1 Very important<br>2 Somewhat important<br>3 Not important
	135	[dxorn_expbonemeasure]	Importance of exposed bone measurement in definition of ORN	radio<br>1 Very important<br>2 Somewhat important<br>3 Not important
	136	[dxorn_duration]	Importance of exposed bone duration in definition of ORN	radio<br>1 Very important<br>2 Somewhat important<br>3 Not important
	137	[dxorn_imaging]	Importance of reporting imaging findings in definition of ORN	radio<br>1 Very important<br>2 Somewhat important<br>3 Not important

medRxiv preprint doi: https://doi.org/10.1101/2024.04.07.24305400; this version posted April 9, 2024. The copyright holder for this preprint (which was not certified by peer review) is the author/funder, who has granted medRxiv a license to display the preprint in perpetuity.
It is made available under a CC-BY 4.0 International license .

	138	[additionalterms_orn]	What additional core elements do you think are needed for diagnosing/defining ORN? Leave blank if none.	notes
	139	[stage_coffin]	Section Header: *Several staging/grading systems for classifying the extent of ORN have been published, and 15 will be reviewed in this section. Please note differences in criteria which may be related to clinical findings, radiological findings, disease progression, or response to therapy. Additionally, 3 potential ORN case scenarios will be linked to each system. In consideration of efforts to reference a system's knowledge to another, please do your best at classifying these cases according to the specific staging/grading system in question.Select "unable to classify" only if very unsure on how to classify the patient. Abbreviations: Hyperbaric oxygen therapy (HBO)*<br>Have you ever used the Coffin (1983) classification system before? Primary basis for classification: Clinical findings Stages Description Minor Series of small sequestra which separate spontaneously over time (weeks or months) Major Bone necrosis extending entire thickness of the jaw; pathological fracture may be present	yesno<br>1 Yes<br>0 No<br>Custom alignment: LV
	140	[coffin_rate]	How effective do you find the Coffin system to be for classifying ORN?	radio<br>0 Very effective<br>1 Somewhat effective<br>2 Neutral<br>3 Somewhat ineffective<br>4 Very ineffective<br>Custom alignment: LV
	141	[matrix_cases_coffin_2]	For each case of potential ORN, please classify the patient using the Coffin system. Assume all completed HN RT. Case 1: Patient with exposed bone (no measurement) not involving lower mandible, unknown duration. Pain present Case 2: Patient with 1.2 cm exposed bone for 4 months, pain present Case 3: Patient with 3 cm exposed bone with pathologic fracture. Unknown symptoms or duration {case1_coffin} {case2_coffin} {case3_coffin}	descriptive
	142	[case1_coffin]	Case 1: Patient with exposed bone (no measurement) not involving lower mandible, unknown duration. Pain present	radio<br>1 Minor<br>2 Major<br>3 Not ORN<br>4 Unable to classify
	143	[case2_coffin]	Case 2: Patient with 1.2 cm exposed bone for 4 months, pain present	radio<br>1 Minor<br>2 Major<br>3 Not ORN<br>4 Unable to classify
	144	[case3_coffin]	Case 3: Patient with 3 cm exposed bone with pathologic fracture. Unknown symptoms or duration	radio<br>1 Minor<br>2 Major<br>3 Not ORN<br>4 Unable to classify
	145	[comments_coffin]	Comments on this staging system (if any)	text<br>Custom alignment: LV
	146	[stage_marx]	Section Header:<br>Have you ever used the Marx (1983) staging system before? Primary basis for classification: Response to therapy (i.e., HBO) Stages Description I 30 HBO dives -> re-examine -> 30 more dives if responder for mucosal recovery II Stage I non-responders; transoral alveolar sequestrectomy + HBO III Stage II non-responders OR initial presentation with either 1) pathologic fracture, 2) orocutaneous fistula, or 3) radiographic evidence of	yesno<br>1 Yes<br>0 No<br>Custom alignment: LV

medRxiv preprint doi: https://doi.org/10.1101/2024.04.07.24305400; this version posted April 9, 2024. The copyright holder for this preprint (which was not certified by peer review) is the author/funder, who has granted medRxiv a license to display the preprint in perpetuity.
It is made available under a CC-BY 4.0 International license .

			resportion to inferior border. Treatment: Bone resection + HBO until mucosal recovery (or 60 dives) IV Additional HBO prior to bone graft	
	147	[marx_rate]	How effective do you find the Marx system to be for classifying ORN?	radio<br>0 Very effective<br>1 Somewhat effective<br>2 Neutral<br>3 Somewhat ineffective<br>4 Very ineffective<br><br>Custom alignment: LV
	148	[matrix_cases_marx]	For each case of potential ORN, please classify the patient using the Marx system. Assume all completed HN RT. Case 1: Patient with exposed bone (no measurement) not involving lower mandible, unknown duration. Pain present Case 2: Patient with 1.2 cm exposed bone for 4 months, pain present Case 3: Patient with 3 cm exposed bone with pathologic fracture. Unknown symptoms or duration {case1_marx} {case2_marx} {case3_marx}	descriptive
	149	[case1_marx]	Case 1 marx	radio<br>1 Stage I<br>2 Stage II<br>3 Stage III<br>4 Stage IV<br>5 Not ORN<br>6 Unable to classify
	150	[case2_marx]	Case 2 marx	radio<br>1 Stage I<br>2 Stage II<br>3 Stage III<br>4 Stage IV<br>5 Not ORN<br>6 Unable to classify
	151	[case3_marx]	Case 3 marx	radio<br>1 Stage I<br>2 Stage II<br>3 Stage III<br>4 Stage IV<br>5 Not ORN<br>6 Unable to classify
	152	[comments_marx]	Comments on this staging system (if any)	text<br>Custom alignment: LV
	153	[stage_morton_simpson]	Section Header:<br>Have you ever used the Morton and Simpson (1986) classification system before? Primary basis for classification: Clinical findings and/or response over time Stage Description Minor Ulceration with exposed bone and history of loss of bony spicules which healed spontaneously over a period of months Moderate Exposed bone and small sequestra limited in nature and healing spontaneously or with conservative treatment within 6-12 months Major Large areas of exposed bone with formation of large sequestra, possible fracture, and sinus formation	yesno<br>1 Yes<br>0 No<br><br>Custom alignment: LV
	154	[morton_simpson_rate]	How effective do you find the Morton system to be for classifying ORN?	radio<br>0 Very effective

medRxiv preprint doi: https://doi.org/10.1101/2024.04.07.24305400; this version posted April 9, 2024. The copyright holder for this preprint (which was not certified by peer review) is the author/funder, who has granted medRxiv a license to display the preprint in perpetuity.
It is made available under a CC-BY 4.0 International license .

				1 Somewhat effective<br>2 Neutral<br>3 Somewhat ineffective<br>4 Very ineffective<br><br>Custom alignment: LV
	155	[matrix_cases_morton]	For each case of potential ORN, please classify the patient using the Morton system. Assume all completed HN RT. Case 1: Patient with exposed bone (no measurement) not involving lower mandible, unknown duration. Pain present Case 2: Patient with 1.2 cm exposed bone for 4 months, pain present Case 3: Patient with 3 cm exposed bone with pathologic fracture. Unknown symptoms or duration {case1_morton} {case2_morton} {case3_morton}	descriptive
	156	[case1_morton]	Case 1 morton	radio<br>1 Minor<br>2 Moderate<br>3 Major<br>4 Not ORN<br>5 Unable to classify
	157	[case2_morton]	Case 2 morton	radio<br>1 Minor<br>2 Moderate<br>3 Major<br>4 Not ORN<br>5 Unable to classify
	158	[case3_morton]	Case 3 morton	radio<br>1 Minor<br>2 Moderate<br>3 Major<br>4 Not ORN<br>5 Unable to classify
	159	[comments_morton]	Comments on this staging system (if any)	text<br>Custom alignment: LV
	160	[stage_epstein]	Section Header:<br>Have you ever used the Epstein (1987) staging system before? Primary basis for classification: Clinical and/or radiographic findings (latter not defined) Stage Description Treatment I Healed, resolved ORN. No symptoms. Ia: No pathologic fracture Ib: Pathologic fracture Surveillance, conservative therapy Surgery (jaw reconstruction) II Chronic, persistent (> 3 months) ORN with stable symptoms (non-progressive paresthesia; pain-free or controlled) IIa: No pathologic fracture IIb: Pathologic fracture Local wound care, conservative therapy Surgery (jaw reconstruction) III Active progressive ORN with progressive symptoms. IIIa: No pathologic fracture IIIb: Pathologic fracture Conservative therapy -> HBO and surgery if non-responders Surgery (jaw reconstruction)	yesno<br>1 Yes<br>0 No<br><br>Custom alignment: LV
	161	[epstein_rate]	How effective do you find the Epstein system to be for classifying ORN?	radio<br>0 Very effective<br>1 Somewhat effective<br>2 Neutral<br>3 Somewhat ineffective<br>4 Very ineffective

medRxiv preprint doi: https://doi.org/10.1101/2024.04.07.24305400; this version posted April 9, 2024. The copyright holder for this preprint (which was not certified by peer review) is the author/funder, who has granted medRxiv a license to display the preprint in perpetuity.
It is made available under a CC-BY 4.0 International license .

				Custom alignment: LV
	162	[matrix_cases_epstein]	For each case of potential ORN, please classify the patient using the Morton system. Assume all completed HN RT. Case 1: Patient with exposed bone (no measurement) not involving lower mandible, unknown duration. Pain present Case 2: Patient with 1.2 cm exposed bone for 4 months, pain present Case 3: Patient with 3 cm exposed bone with pathologic fracture. Unknown symptoms or duration {case1_eptsein} {case2_eptsein} {case3_eptsein}	descriptive
	163	[case1_eptsein]	Case 1 epstein	radio<br>1 Stage I<br>2 Stage II<br>3 Stage III<br>4 Not ORN<br>5 Unable to classify
	164	[case2_eptsein]	Case 2 epstein	radio<br>1 Stage I<br>2 Stage II<br>3 Stage III<br>4 Not ORN<br>5 Unable to classify
	165	[case3_eptsein]	Case 3 epstein	radio<br>1 Stage I<br>2 Stage II<br>3 Stage III<br>4 Not ORN<br>5 Unable to classify
	166	[comments_epstein]	Comments on this staging system (if any)	text<br>Custom alignment: LV
	167	[stage_glanzmann_gratz]	Section Header:<br>Have you ever used the Glanzmann and Gratz (1995) grading system before? Primary basis for classification: Clinical findings and response to surgery Grade Description 1 Bone exposure without signs of infection and persisting for at least 3 months 2 Bone exposure with signs of infection or sequester and w/o signs of G3-5 3 Bone necrosis treated with mandibular resection with satisfactory result 4 Bone necrosis treated with mandibular resection with persisting problems 5 Death due to ORN	yesno<br>1 Yes<br>0 No<br>Custom alignment: LV
	168	[glanzmann_gratz_rate]	How effective do you find the Glantzmann system to be for classifying ORN?	radio<br>0 Very effective<br>1 Somewhat effective<br>2 Neutral<br>3 Somewhat ineffective<br>4 Very ineffective<br>Custom alignment: LV
	169	[matrix_cases_glantzmann]	For each case of potential ORN, please classify the patient using the Glantzmann system. Assume all completed HN RT. Case 1: Patient with exposed bone (no measurement) not involving lower mandible, unknown duration. Pain present Case 2: Patient with 1.2 cm exposed bone for 4 months, pain present Case 3: Patient with 3 cm exposed bone with pathologic fracture. Unknown symptoms or duration {case1_glantz} {case2_glantz} {case3_glantz}	descriptive

medRxiv preprint doi: https://doi.org/10.1101/2024.04.07.24305400; this version posted April 9, 2024. The copyright holder for this preprint (which was not certified by peer review) is the author/funder, who has granted medRxiv a license to display the preprint in perpetuity.
It is made available under a CC-BY 4.0 International license .

	170	[case1_glantz]	Case 1 glantzmann	radio<br>1 Grade 0 (not ORN)<br>2 Grade I<br>3 Grade 2<br>4 Grade 3<br>5 Grade 4<br>6 Grade 5<br>7 Unable to classify
	171	[case2_glantz]	Case 2 glantzmann	radio<br>1 Grade 0 (not ORN)<br>2 Grade I<br>3 Grade 2<br>4 Grade 3<br>5 Grade 4<br>6 Grade 5<br>7 Unable to classify
	172	[case3_glantz]	Case 3 glantzmann	radio<br>1 Grade 0 (not ORN)<br>2 Grade I<br>3 Grade 2<br>4 Grade 3<br>5 Grade 4<br>6 Grade 5<br>7 Unable to classify
	173	[comments_glantzmann]	Comments on this staging system (if any)	text<br>Custom alignment: LV
	174	[stage_clayman]	Section Header:<br>Have you ever used the Clayman (1997) staging system before? Primary basis for classification: Clinical findings Used Marx definition of ORN: "Nonhealing, nonseptic lesion of bone in which volume and density cannot be maintained by the hypocellular, hypovascular, hypoxic tissue which cannot adequately meet its metabolic demands." Type Description I ORN presenting with bone lysis under intact gingiva or mucosa II Aggressive ORN. Soft tissue breakdown, bone exposed to saliva, and secondary contamination occurs; 'radiation osteomyelitis'	yesno<br>1 Yes<br>0 No<br><br>Custom alignment: LV
	175	[clayman_rate]	How effective do you find the Clayman system to be for classifying ORN?	radio<br>0 Very effective<br>1 Somewhat effective<br>2 Neutral<br>3 Somewhat ineffective<br>4 Very ineffective<br><br>Custom alignment: LV
	176	[matrix_cases_clayman]	For each case of potential ORN, please classify the patient using the Clayman system. Assume all completed HN RT. Case 1: Patient with exposed bone (no measurement) not involving lower mandible, unknown duration. Pain present Case 2: Patient with 1.2 cm exposed bone for 4 months, pain present Case 3: Patient with 3 cm exposed bone with pathologic fracture. Unknown symptoms or duration {case1_clayman} {case2_clayman} {case3_clayman}	descriptive
	177	[case1_clayman]	Case 1 clayman	radio<br>1 Type I

medRxiv preprint doi: https://doi.org/10.1101/2024.04.07.24305400; this version posted April 9, 2024. The copyright holder for this preprint (which was not certified by peer review) is the author/funder, who has granted medRxiv a license to display the preprint in perpetuity.
It is made available under a CC-BY 4.0 International license .

				2 Type II<br>3 Not ORN<br>4 Unable to classify
	178	[case2_clayman]	Case 2 clayman	radio<br>1 Type I<br>2 Type II<br>3 Not ORN<br>4 Unable to classify
	179	[case3_clayman]	Case 3 clayman	radio<br>1 Type I<br>2 Type II<br>3 Not ORN<br>4 Unable to classify
	180	[comments_clayman]	Comments on this staging system (if any)	text<br>Custom alignment: LV
	181	[stage_store_boysen]	Section Header:<br>Have you ever used the Store and Boysen (2000) staging system before? Primary basis for classification: Clinical and radiographic findings (latter not defined) Stage Description 0 Mucosal defects only (denuded bone intra-orally without any positive radiological signs) I Radiological evidence of bone necrosis with intact mucosa II Exposed non-vital bone and positive radiological signs, but without any sign of infection III Exposed bone, radiological evidence, extraoral fistula and infection	yesno<br>1 Yes<br>0 No<br><br>Custom alignment: LV
	182	[store_boysen_rate]	How effective do you find the Store staging system to be for classifying ORN?	radio<br>0 Very effective<br>1 Somewhat effective<br>2 Neutral<br>3 Somewhat ineffective<br>4 Very ineffective<br><br>Custom alignment: LV
	183	[matrix_cases_store]	For each case of potential ORN, please classify the patient using the Store system. Assume all completed HN RT. Case 1: Patient with exposed bone (no measurement) not involving lower mandible, unknown duration. Pain present Case 2: Patient with 1.2 cm exposed bone for 4 months, pain present Case 3: Patient with 3 cm exposed bone with pathologic fracture. Unknown symptoms or duration {case1_store} {case2_store} {case3_store}	descriptive
	184	[case1_store]	Case 1 store	radio<br>1 Stage 0<br>2 Stage I<br>3 Stage II<br>4 Stage III<br>5 Not ORN<br>6 Unable to classify
	185	[case2_store]	Case 2 store	radio<br>1 Stage 0<br>2 Stage I<br>3 Stage II<br>4 Stage III<br>5 Not ORN

medRxiv preprint doi: https://doi.org/10.1101/2024.04.07.24305400; this version posted April 9, 2024. The copyright holder for this preprint (which was not certified by peer review) is the author/funder, who has granted medRxiv a license to display the preprint in perpetuity.
It is made available under a CC-BY 4.0 International license .

				6 Unable to classify
	186	[case3_store]	Case 3 store	radio<br>1 Stage 0<br>2 Stage I<br>3 Stage II<br>4 Stage III<br>5 Not ORN<br>6 Unable to classify
	187	[comments_store]	Comments on this staging system (if any)	text<br>Custom alignment: LV
	188	[stage_schwartz_kagan]	Section Header:<br>Have you ever used the Schwartz and Kagan (2002) staging system before? Primary basis for classification: Clinical findings Definition for ORN: "A condition in which devitalized, irradiated bone becomes exposed through a wound in the overlying skin or mucosa. Such a wound must not be caused by tumor recurrence, or by tumor necrosis during radiation therapy, and it must persist without healing for 3 to 6 months." Associated with general treatment approach Stage Description I Superficial involvement of the mandible only. Soft-tissue ulceration is minimal. Only exposed cortical bone is necrotic. II Localized involvement of the mandible. The exposed cortical bone and also a portion of the underlying medullary bone are necrotic. Division A: Soft-tissue ulceration is minimal Division B: There is soft-tissue necrosis, including orocutaneous fistulation III Diffuse involvement of the mandible. The full-thickness segment of bone is involved, including the lower border. Pathologic fracture may occur. Division A: Soft-tissue ulceration is minimal Division B: There is soft-tissue necrosis, including orocutaneous fistulation	yesno<br>1 Yes<br>0 No<br><br>Custom alignment: LV
	189	[schwartz_kagan_rate]	How effective do you find the Schwartz staging system to be for classifying ORN?	radio<br>0 Very effective<br>1 Somewhat effective<br>2 Neutral<br>3 Somewhat ineffective<br>4 Very ineffective<br><br>Custom alignment: LV
	190	[matrix_cases_schwartz]	For each case of potential ORN, please classify the patient using the Schwartz system. Assume all completed HN RT. Case 1: Patient with exposed bone (no measurement) not involving lower mandible, unknown duration. Pain present Case 2: Patient with 1.2 cm exposed bone for 4 months, pain present Case 3: Patient with 3 cm exposed bone with pathologic fracture. Unknown symptoms or duration {case1_schwartz} {case2_schwartz} {case3_schwartz}	descriptive
	191	[case1_schwartz]	Case 1 schwartz	radio<br>1 Stage I<br>2 Stage II<br>3 Stage III<br>4 Not ORN<br>5 Unable to classify
	192	[case2_schwartz]	Case 2 schwartz	radio<br>1 Stage I<br>2 Stage II<br>3 Stage III

medRxiv preprint doi: https://doi.org/10.1101/2024.04.07.24305400; this version posted April 9, 2024. The copyright holder for this preprint (which was not certified by peer review) is the author/funder, who has granted medRxiv a license to display the preprint in perpetuity.
It is made available under a CC-BY 4.0 International license .

				4 Not ORN<br>5 Unable to classify
	193	[case3_schwartz]	Case 3 schwartz	radio<br>1 Stage I<br>2 Stage II<br>3 Stage III<br>4 Not ORN<br>5 Unable to classify
	194	[comments_schwartz]	Comments on this staging system (if any)	text<br>Custom alignment: LV
	195	[stage_notani]	Section Header:<br>Have you ever used the Notani (2003) grading system before? Primary basis for classification: Clinical findings (extent of lesion) Grades Description I ORN confined to alveolar bone II ORN limited to alveolar bone and/or the mandible above the level of the inferior alveolar canal III ORN extending under the level of the inferior alveolar canal or ORN with skin fistula and/or pathological fracture	yesno<br>1 Yes<br>0 No<br><br>Custom alignment: LV
	196	[notani_rate]	How effective do you find the Notani system to be for classifying ORN?	radio<br>0 Very effective<br>1 Somewhat effective<br>2 Neutral<br>3 Somewhat ineffective<br>4 Very ineffective<br><br>Custom alignment: LV
	197	[matrix_cases_notani]	For each case of potential ORN, please classify the patient using the Notani system. Assume all completed HN RT. Case 1: Patient with exposed bone (no measurement) not involving lower mandible, unknown duration. Pain present Case 2: Patient with 1.2 cm exposed bone for 4 months, pain present Case 3: Patient with 3 cm exposed bone with pathologic fracture. Unknown symptoms or duration {case1_notani} {case2_notani} {case3_notani}	descriptive
	198	[case1_notani]	Case 1 notani	radio<br>1 Grade 1<br>2 Grade 2<br>3 Grade 3<br>4 Not ORN<br>5 Unable to classify
	199	[case2_notani]	Case 2 notani	radio<br>1 Grade 1<br>2 Grade 2<br>3 Grade 3<br>4 Not ORN<br>5 Unable to classify
	200	[case3_notani]	Case 3 notani	radio<br>1 Grade 1<br>2 Grade 2<br>3 Grade 3<br>4 Not ORN<br>5 Unable to classify
	201	[comments_notani]	Comments on this staging system (if any)	text<br>Custom alignment: LV

medRxiv preprint doi: https://doi.org/10.1101/2024.04.07.24305400; this version posted April 9, 2024. The copyright holder for this preprint (which was not certified by peer review) is the author/funder, who has granted medRxiv a license to display the preprint in perpetuity.
It is made available under a CC-BY 4.0 International license .

	202	[modifiednotanipic]	Shaw et al. (2017) proposed a modified Notani ORN classification for use in clinical trials. As shown below, this system adds duration of exposed bone (6 month threshold) and minor bone spicules (MBS), defined as 'not ORN' with a surface area of < 20mm^2.	descriptive<br>(Attachment: Screen Shot 2023-02-28 at 11.07.34 PM.png, Display format: Inline image/PDF)
	203	[modnotani_use]	Have you ever used the modified Notani classification before?	yesno<br>1 Yes<br>0 No
	204	[modnotani_rate]	How effective do you find the modified Notani system to be for classifying ORN?	radio<br>0 Very effective<br>1 Somewhat effective<br>2 Neutral<br>3 Somewhat ineffective<br>4 Very ineffective<br><br>Custom alignment: LV
	205	[comments_modnotani]	Comments on this staging system (if any)	text<br>Custom alignment: LV
	206	[stage_tsai]	Section Header:<br>Have you ever used the Tsai (2013) grading system before? Primary basis for classification: Clinical findings or treatment needed Grade Description 1 Minimal bone exposure with conservative management only 2 Minor debridement received 3 HBO needed 4 Major surgery required	yesno<br>1 Yes<br>0 No<br><br>Custom alignment: LV
	207	[tsai_rate]	How effective do you find the Tsai system to be for classifying ORN?	radio<br>0 Very effective<br>1 Somewhat effective<br>2 Neutral<br>3 Somewhat ineffective<br>4 Very ineffective<br><br>Custom alignment: LV
	208	[matrix_cases_tsai]	For each case of potential ORN, please classify the patient using the Tsai system. Assume all completed HN RT. Case 1: Patient with exposed bone (no measurement) not involving lower mandible, unknown duration. Pain present Case 2: Patient with 1.2 cm exposed bone for 4 months, pain present Case 3: Patient with 3 cm exposed bone with pathologic fracture. Unknown symptoms or duration {case1_tsai} {case2_tsai} {case3_tsai}	descriptive
	209	[case1_tsai]	Case 1 tsai	radio<br>1 Grade 1<br>2 Grade 2<br>3 Grade 3<br>4 Grade 4<br>5 Not ORN<br>6 Unable to classify
	210	[case2_tsai]	Case 2 tsai	radio<br>1 Grade 1<br>2 Grade 2<br>3 Grade 3<br>4 Grade 4<br>5 Not ORN<br>6 Unable to classify

medRxiv preprint doi: https://doi.org/10.1101/2024.04.07.24305400; this version posted April 9, 2024. The copyright holder for this preprint (which was not certified by peer review) is the author/funder, who has granted medRxiv a license to display the preprint in perpetuity.
It is made available under a CC-BY 4.0 International license .

	211	[case3_tsai]	Case 3 tsai	radio<br>1 Grade 1<br>2 Grade 2<br>3 Grade 3<br>4 Grade 4<br>5 Not ORN<br>6 Unable to classify
	212	[comments_tsai]	Comments on this staging system (if any)	text<br>Custom alignment: LV
	213	[stage_karagozoglu]	Section Header:<br>Have you ever used the Karagozoglu (2014) staging system before? Primary basis for classification: Clinical and/or imaging findings (panoramic radiograph or periapical films recommended) Definition for ORN: "Radiation induced necrosis of bone. A diagnosis of ORN was rendered in the presence of exposed bone, with or without changes on plain radiograph, having excluded the presence of tumour tissue, either being a second primary or a recurrence. For the purposes of this study a definitive diagnosis of ORN has been made in case of presence of exposed bone for at least one month." Stage Description 0 Exposure of mandibular bone for less than 1 month; no distinct changes on plain radiographs (panoramic radiograph or periapical film) I Exposure of mandibular bone for at least 1 month; no distinct changes on plain radiographs. IA: Asymptomatic (no pain or presence of cutaneous fistulas) IB: Symptomatic (pain or presence of cutaneous fistulas) II Exposure of mandibular bone for at least 1 month; distinct changes present on plain radiographs, but no involving the lower border of the mandible. IIA: Asymptomatic IIB: Symptomatic III Exposure of mandibular bone for at least 1 month; distinct changes on plain radiographs, involving the lower border of the mandible, irrespective of any other signs or symptoms	yesno<br>1 Yes<br>0 No<br><br>Custom alignment: LV
	214	[karagozoglu_rate]	How effective do you find the Karragozoglu system to be for classifying ORN?	radio<br>0 Very effective<br>1 Somewhat effective<br>2 Neutral<br>3 Somewhat ineffective<br>4 Very ineffective<br><br>Custom alignment: LV
	215	[matrix_cases_kar]	For each case of potential ORN, please classify the patient using the Karagozoglu system. Assume all completed HN RT. Case 1: Patient with exposed bone (no measurement) not involving lower mandible, unknown duration. Pain present Case 2: Patient with 1.2 cm exposed bone for 4 months, pain present Case 3: Patient with 3 cm exposed bone with pathologic fracture. Unknown symptoms or duration {case1_kar} {case2_kar} {case3_kar}	descriptive
	216	[case1_kar]	Case 1 kar	radio<br>1 Stage 0<br>2 Stage I<br>3 Stage II<br>4 Stage III<br>5 Not ORN<br>6 Unable to classify
	217	[case2_kar]	Case 2 kar	radio<br>1 Stage 0

medRxiv preprint doi: https://doi.org/10.1101/2024.04.07.24305400; this version posted April 9, 2024. The copyright holder for this preprint (which was not certified by peer review) is the author/funder, who has granted medRxiv a license to display the preprint in perpetuity.
It is made available under a CC-BY 4.0 International license .

				2 Stage I<br>3 Stage II<br>4 Stage III<br>5 Not ORN<br>6 Unable to classify
	218	[case3_kar]	Case 3 kar	radio<br>1 Stage 0<br>2 Stage I<br>3 Stage II<br>4 Stage III<br>5 Not ORN<br>6 Unable to classify
	219	[comments_karr]	Comments on this staging system (if any)	text<br>Custom alignment: LV
	220	[stage_lyons]	Section Header:<br>Have you ever used the Lyons (2014) staging system before? Primary basis for classification: Clinical findings and treatment approach Definition for ORN: Incorporated into the staging system. Authors acknowledged definitions for ORN which may or may not include bony exposure (i.e., Store classification of radiological evidence of bone necrosis without bony exposure). In the Lyons staging system, affected bone can be "damaged or exposed". Stage Clinical Findings by Stage Treatment Recommendation by Stage I < 2.5 cm length of bone affected (damaged or exposed); asymptomatic Medical treatment only II >2.5 cm length of bone; asymptomatic, including pathological fracture or involvement of inferior dental nerve, or both Medical treatment only unless there is dental sepsis or obviously loose, necrotic bone III >2.5 cm length of bone; symptomatic, but with no other features despite medical treatment Consider debridement of loose or necrotic bone, and local pedicled flap IV >2.5 cm length of bone; pathological fracture, involvement of inferior dental nerve, or orocutaneous fistula, or a combination Reconstruction with free flap if patient's overall condition allows	yesno<br>1 Yes<br>0 No<br><br>Custom alignment: LV
	221	[lyons_rate]	How effective do you find the Lyons system to be for classifying ORN?	radio<br>0 Very effective<br>1 Somewhat effective<br>2 Neutral<br>3 Somewhat ineffective<br>4 Very ineffective<br><br>Custom alignment: LV
	222	[matrix_cases_lyons]	For each case of potential ORN, please classify the patient using the Lyons system. Assume all completed HN RT. Case 1: Patient with exposed bone (no measurement) not involving lower mandible, unknown duration. Pain present Case 2: Patient with 1.2 cm exposed bone for 4 months, pain present Case 3: Patient with 3 cm exposed bone with pathologic fracture. Unknown symptoms or duration {case1_lyons} {case2_lyons} {case3_lyons}	descriptive
	223	[case1_lyons]	Case 1 lyons	radio<br>1 Stage I<br>2 Stage II<br>3 Stage III<br>4 Stage IV

medRxiv preprint doi: https://doi.org/10.1101/2024.04.07.24305400; this version posted April 9, 2024. The copyright holder for this preprint (which was not certified by peer review) is the author/funder, who has granted medRxiv a license to display the preprint in perpetuity.
It is made available under a CC-BY 4.0 International license .

				5 Not ORN<br>6 Unable to classify
	224	[case2_lyons]	Case 2 lyons	radio<br>1 Stage I<br>2 Stage II<br>3 Stage III<br>4 Stage IV<br>5 Not ORN<br>6 Unable to classify
	225	[case3_lyons]	Case 3 lyons	radio<br>1 Stage I<br>2 Stage II<br>3 Stage III<br>4 Stage IV<br>5 Not ORN<br>6 Unable to classify
	226	[comments_lyons]	Comments on this staging system (if any)	text<br>Custom alignment: LV
	227	[stage_lyons_2]	Section Header:<br>Have you ever used the He (2015) staging system before? Primary basis for classification: Clinical and imaging findings (both required for each stage) Definition for ORN: Authors acknowledged definitions for ORN which may or may not include bony exposure (i.e., Store classification of radiological evidence of bone necrosis without bony exposure). In the He staging system, ORN can be present without the precondition of bone exposure. Stage Description 0 No evident signs or only osteolytic images on radiography; symptomatic (bone exposure or pain) I < 2cm radiographic lesion and: B1S0: No mucosa or skin defect (intact mucosa) B1S1: Intraoral mucosa defect or external skin fistula alone B1S2: Through-and-through defect (both intraoral & skin defect) II >2 cm radiographic lesion and: B2S0: No mucosa or skin defect (intact mucosa) B2S1: Intraoral mucosa defect or external skin fistula alone B2S2: Through-and-through defect (both intraoral & skin defect) III A pathologic fracture identified on radiographic and: B3S0: No mucosa or skin defect (intact mucosa) B3S1: Intraoral mucosa defect or external skin fistula alone B3S2: Through-and-through defect (both intraoral & skin defect)	yesno<br>1 Yes<br>0 No<br><br>Custom alignment: LV
	228	[he_rate]	How effective do you find the He system to be for classifying ORN?	radio<br>0 Very effective<br>1 Somewhat effective<br>2 Neutral<br>3 Somewhat ineffective<br>4 Very ineffective<br><br>Custom alignment: LV
	229	[matrix_cases_he]	For each case of potential ORN, please classify the patient using the He system. Assume all completed HN RT. Case 1: Patient with exposed bone (no measurement) not involving lower mandible, unknown duration. Pain present Case 2: Patient with 1.2 cm exposed bone for 4 months, pain present Case 3: Patient with 3 cm exposed bone with pathologic fracture. Unknown symptoms or duration {case1_he} {case2_he} {case3_he}	descriptive
	230	[case1_he]	Case 1 He	radio

medRxiv preprint doi: https://doi.org/10.1101/2024.04.07.24305400; this version posted April 9, 2024. The copyright holder for this preprint (which was not certified by peer review) is the author/funder, who has granted medRxiv a license to display the preprint in perpetuity.
It is made available under a CC-BY 4.0 International license .

				1 Stage 0<br>2 Stage I<br>3 Stage II<br>4 Stage III<br>5 Not ORN<br>6 Unable to classify
	231	[case2_he]	Case 2 He	radio<br>1 Stage 0<br>2 Stage I<br>3 Stage II<br>4 Stage III<br>5 Not ORN<br>6 Unable to classify
	232	[case3_he]	Case 3 He	radio<br>1 Stage 0<br>2 Stage I<br>3 Stage II<br>4 Stage III<br>5 Not ORN<br>6 Unable to classify
	233	[comments_he]	Comments on this staging system (if any)	text<br>Custom alignment: LV
	234	[stage_ctcae]	Section Header:<br>Have you ever used the Common Terminology Criteria for Adverse Events (CTCAE) for ORN before? Version 5.0 shown below for osteonecrosis of jaw Definition of Osteonecrosis: "A disorder characterized by a necrotic process occuring in the bone of the mandible." Grade Description 0 No ORN 1 Asymptomatic; clinical or diagnostic observations only; intervention not indicated 2 Symptomatic; medical intervention indicated (e.g., topical agents); limiting instrumental ADL 3 Severe symptoms; limiting self care ADL; elective operative intervention indicated 4 Life-threatening consequences; urgent intervention indicated 5 Death	yesno<br>1 Yes<br>0 No<br><br>Custom alignment: LV
	235	[ctcae_rate]	How effective do you find CTCAE v5.0 to be for classifying ORN?	radio<br>0 Very effective<br>1 Somewhat effective<br>2 Neutral<br>3 Somewhat ineffective<br>4 Very ineffective<br><br>Custom alignment: LV
	236	[matrix_cases_ctcae]	For each case of potential ORN, please classify the patient using the CTCAE criteria. Assume all completed HN RT. Case 1: Patient with exposed bone (no measurement) not involving lower mandible, unknown duration. Pain present Case 2: Patient with 1.2 cm exposed bone for 4 months, pain present Case 3: Patient with 3 cm exposed bone with pathologic fracture. Unknown symptoms or duration {case1_ctcae} {case2_ctcae} {case3_ctcae}	descriptive
	237	[case1_ctcae]	Case 1 ctcae	radio<br>1 Grade 0 (No ORN)<br>2 Grade 1<br>3 Grade 2

medRxiv preprint doi: https://doi.org/10.1101/2024.04.07.24305400; this version posted April 9, 2024. The copyright holder for this preprint (which was not certified by peer review) is the author/funder, who has granted medRxiv a license to display the preprint in perpetuity.
It is made available under a CC-BY 4.0 International license .

				4 Grade 3<br>5 Grade 4<br>6 Grade 5<br>7 Unable to classify
	238	[case2_ctcae]	Case 2 ctcae	radio<br>1 Grade 0 (No ORN)<br>2 Grade 1<br>3 Grade 2<br>4 Grade 3<br>5 Grade 4<br>6 Grade 5<br>7 Unable to classify
	239	[case3_ctcae]	Case 3 ctcae	radio<br>1 Grade 0 (No ORN)<br>2 Grade 1<br>3 Grade 2<br>4 Grade 3<br>5 Grade 4<br>6 Grade 5<br>7 Unable to classify
	240	[add_staging_orn]	Are there any additional grading/staging systems for ORN that you want the Consortium to review?	yesno<br>1 Yes<br>0 No<br><br>Custom alignment: LV
	241	[staging_condition]<br><br>Show the field ONLY if:<br>[add_staging_orn] = '1'	If yes, please describe	notes<br>Custom alignment: LV
	242	[table1_system_elements]	Section Header: *Staging/grading data elements review*<br><br>Please click on the link to find an overview of reported data elements per staging/grading systems. You may want to have this opened separately while answering the next series of questions.	descriptive<br>(Attachment: ORN elements summarized-Table 1.png, Display format: Link)
	243	[exposed_bone]	Section Header: *Please rate the level of importance for each. Consider items labeled as "very important" for mandatory documentation during follow ups on all HNC cases treated with RT, and/or for inclusion in an ORN ontology.*<br><br>Exposed bone	radio (Matrix)<br>1 Not important<br>2 Somewhat important<br>3 Very important
	244	[ebone_extent]	Extend of mandibular involvement	radio (Matrix)<br>1 Not important<br>2 Somewhat important<br>3 Very important
	245	[ulceration]	Ulceration	radio (Matrix)<br>1 Not important<br>2 Somewhat important<br>3 Very important
	246	[bone_spicules]	Bone spicules	radio (Matrix)<br>1 Not important<br>2 Somewhat important<br>3 Very important
	247	[sequestra]	Sequestra	radio (Matrix)

medRxiv preprint doi: https://doi.org/10.1101/2024.04.07.24305400; this version posted April 9, 2024. The copyright holder for this preprint
(which was not certified by peer review) is the author/funder, who has granted medRxiv a license to display the preprint in perpetuity.
It is made available under a CC-BY 4.0 International license .

				1 Not important<br>2 Somewhat important<br>3 Very important
	248	[time]	Time (duration of exposure)	radio (Matrix)<br>1 Not important<br>2 Somewhat important<br>3 Very important
	249	[path_fracture]	Pathological fracture	radio (Matrix)<br>1 Not important<br>2 Somewhat important<br>3 Very important
	250	[fistula]	Orocutaneous fistula	radio (Matrix)<br>1 Not important<br>2 Somewhat important<br>3 Very important
	251	[sinus_form]	Sinus formation	radio (Matrix)<br>1 Not important<br>2 Somewhat important<br>3 Very important
	252	[infection]	Signs of infection	radio (Matrix)<br>1 Not important<br>2 Somewhat important<br>3 Very important
	253	[symptoms]	Symptoms	radio (Matrix)<br>1 Not important<br>2 Somewhat important<br>3 Very important
	254	[cons_trt]	Conservative medical therapy	radio (Matrix)<br>1 Not important<br>2 Somewhat important<br>3 Very important
	255	[hbo]	Hyperbaric oxygen therapy (HBO)	radio (Matrix)<br>1 Not important<br>2 Somewhat important<br>3 Very important
	256	[debridement]	Debridement/sequestrectomy	radio (Matrix)<br>1 Not important<br>2 Somewhat important<br>3 Very important
	257	[surgery]	Bone resection/reconstruction	radio (Matrix)<br>1 Not important<br>2 Somewhat important<br>3 Very important
	258	[ebone_post]	Exposed bone after therapy	radio (Matrix)<br>1 Not important<br>2 Somewhat important<br>3 Very important

medRxiv preprint doi: https://doi.org/10.1101/2024.04.07.24305400; this version posted April 9, 2024. The copyright holder for this preprint (which was not certified by peer review) is the author/funder, who has granted medRxiv a license to display the preprint in perpetuity.
It is made available under a CC-BY 4.0 International license .

	259	[gran_tissue_post]	Granulation tissue after therapy	radio (Matrix)<br>1 Not important<br>2 Somewhat important<br>3 Very important
	260	[inflam_post]	Inflammation after therapy	radio (Matrix)<br>1 Not important<br>2 Somewhat important<br>3 Very important
	261	[healing_post]	Healing (clinical improvement)	radio (Matrix)<br>1 Not important<br>2 Somewhat important<br>3 Very important
	262	[dx_localizeoptions]	Currently there are no spatial definitions for ORN (i.e., where is the 1.5cm of exposed bone located?). If possible, which option(s) would you prefer for localizing ORN? Select all that apply.	checkbox<br>1 dx_localizeoptions___1 Auto-segmentation of the mandible (regardless of presence/absence of teeth)<br>2 dx_localizeoptions___2 Based on tooth number<br>3 dx_localizeoptions___3 Based on tooth type (i.e., molar vs. non-molar)<br>4 dx_localizeoptions___4 Based on imaging<br>5 dx_localizeoptions___5 Not required<br>6 dx_localizeoptions___6 Other
	263	[localize_text]<br>Show the field ONLY if:<br>[dx_localizeoptions(6)] = '1'	If other, please describe	text
	264	[add_stagingelements]	Are there additional elements that should be considered for classifying extent/severity of ORN? Leave blank if none.	text
	265	[ct_orn]	Section Header: *Imaging correlates of ORN*<br>Based on your knowledge and experience, how effective is CT imaging for diagnosing ORN?	radio<br>0 Very effective<br>1 Somewhat effective<br>2 Neutral<br>3 Somewhat ineffective<br>4 Very ineffective<br>Custom alignment: LV
	266	[use_ct_orn]	In the past 12 months, have you used CT imaging to evaluate for ORN after RT?	yesno<br>1 Yes<br>0 No<br>Custom alignment: LV
	267	[feature_ct_orn]	What features of CT imaging do you associate with ORN? Select all that apply.	checkbox<br>0 feature_ct_orn___0 Bone erosion<br>1 feature_ct_orn___1 Pathological fracture<br>2 feature_ct_orn___2 Soft tissue thickening<br>3 feature_ct_orn___3 Other<br>Custom alignment: LV
	268	[features_ct_condition]<br>Show the field ONLY if:<br>[feature_ct_orn(3)] = '1'	If other, please describe	notes

medRxiv preprint doi: https://doi.org/10.1101/2024.04.07.24305400; this version posted April 9, 2024. The copyright holder for this preprint (which was not certified by peer review) is the author/funder, who has granted medRxiv a license to display the preprint in perpetuity.
It is made available under a CC-BY 4.0 International license .

	269	[mri_orn]	Based on your knowledge and experience, how effective is MRI for diagnosing ORN?	radio<br>0 Very effective<br>1 Somewhat effective<br>2 Neutral<br>3 Somewhat ineffective<br>4 Very ineffective<br>Custom alignment: LV
	270	[use_mri_orn]	In the past 12 months, have you used MRI to evaluate for ORN after RT?	yesno<br>1 Yes<br>0 No<br>Custom alignment: LV
	271	[imaging_sequence_orn]	What imaging sequences do you use for evaluating ORN?	checkbox<br>0 imaging_sequence_orn___0 Dynamic contrast-enhanced (DCE)<br>1 imaging_sequence_orn___1 T1-weighted without contrast<br>2 imaging_sequence_orn___2 T1-weighted with contrast<br>3 imaging_sequence_orn___3 T2-weighted (T2W)<br>4 imaging_sequence_orn___4 Diffusion-weighted imaging (DWI)<br>5 imaging_sequence_orn___5 Other<br>Custom alignment: LV
	272	[imaging_sequence_condition]<br>Show the field ONLY if: [imaging_sequence_orn(5)] = '1'	If other, please describe	notes
	273	[mri_parameters]	What quantitative MRI parameters do you think are important for assessment of risk, diagnosis, progression, and/or treatment response of ORN? Leave blank if none.	text<br>Custom alignment: LV
	274	[xray_orn]	Based on your knowledge and experience, how effective are orthopantomograms (OPG) for diagnosing ORN?	radio<br>0 Very effective<br>1 Somewhat effective<br>2 Neutral<br>3 Somewhat ineffective<br>4 Very ineffective<br>Custom alignment: LV
	275	[xray_use_orn]	In the past 12 months, have you used OPGs to evaluate for ORN after RT?	yesno<br>1 Yes<br>0 No<br>Custom alignment: LV
	276	[opg_fx]	What features are you looking for on OPGs to diagnose ORN? Leave blank if none.	text<br>Custom alignment: LV
	277	[additional_imaging_orn]	Are there additional imaging methods you use for diagnosing ORN?	yesno<br>1 Yes<br>0 No<br>Custom alignment: LV
	278	[add_image_condition]	If yes, please describe	notes

medRxiv preprint doi: https://doi.org/10.1101/2024.04.07.24305400; this version posted April 9, 2024. The copyright holder for this preprint (which was not certified by peer review) is the author/funder, who has granted medRxiv a license to display the preprint in perpetuity.
It is made available under a CC-BY 4.0 International license .

		Show the field ONLY if: [additional_imaging_orn] = '1'		
	279	[conclusion]	Are there any additional references or questions on ORN that you would like the Consortium to review during round 2? If references, please provide a PMID or reference link.	notes
	280	[round_1_defining_and_classifying_orn_complete]	Section Header: *Form Status*<br>Complete?	dropdown<br>0 Incomplete<br>1 Unverified<br>2 Complete
**Instrument: Round 1 Radmap Visualization** (round_1_radmap_visualization) Enabled as survey [collapsed]				
**Instrument: Participant Agreement** (participant_agreement) Enabled as survey				
	305	[first_name]	First Name	text, Required, Identifier
	306	[last_name]	Last Name	text, Required, Identifier
	307	[email_address]	Email Address to use for study (i.e., surveys, updates)	text (email), Required, Identifier
	308	[consent]	I want to participate in this study as an expert in the ORAL Consortium	yesno, Required<br>1 Yes<br>0 No
	309	[extra_experts2]	Do you recommend another expert for us to reach out to for this study?	yesno<br>1 Yes<br>0 No
	310	[extraexpert_name1]<br>Show the field ONLY if: [extra_experts2] = '1'	Expert's First and Last Name	text
	311	[exteremail1]<br>Show the field ONLY if: [extra_experts2] = '1'	Expert's Email Address	text
	312	[consent_no2]<br>Show the field ONLY if: [consent] = '0'	Thank you for your response and have a great day!	descriptive
	313	[participant_agreement_complete]	Section Header: *Form Status*<br>Complete?	dropdown<br>0 Incomplete<br>1 Unverified<br>2 Complete

Round 1: Introduction and Panel Info

medRxiv preprint doi: https://doi.org/10.1101/2024.04.07.24305400; this version posted April 9, 2024. The copyright holder for this preprint (which was not certified by peer review) is the author/funder, who has granted medRxiv a license to display the preprint in perpetuity.
It is made available under a CC-BY 4.0 International license .

Welcome to the

Orodental ontologies for reporting Radiotherapy-induced Adverse sequeLae (ORAL) Consortium

Thank you all for expressing interest in joining the ORAL Consortium! As noted in the email invitation, the primary goals of this initial work are to 1) develop expert-based orodental data standardization guidelines, and 2) provide input on designs for 3D-to-2D visualizations of doses to teeth (aka 'radiation odontogram' [RADMAP]). These efforts aim to facilitate scalable and comprehensive information sharing among multidisciplinary providers managing patients with head and neck cancers (HNC).

The ORAL Consortium includes international representatives from Radiation Oncology, Head & Neck Surgery, Oral Oncology or Medicine, Dentistry, Radiation Physics, and Symptom Research. A bit of my background: I'm one of the Head & Neck Radiation Oncologists at MD Anderson Cancer Center in Houston, TX. I am also an informatician with NIDCR funded projects focused on defining machine- and human-readable ontologies to relate dental dose from radiotherapy (RT) and treatment-associated HN toxicities, such as osteoradionecrosis (ORN).

ORN is known to be a severe iatrogenic disease that is experienced by 5-20% of HNC survivors. Fundamental understanding of the natural history and mechanistic progression of ORN remains a significant under-explored domain due to heterogeneous definitions of the true disease state and numerous staging/grading systems which can lead to under-reporting or misclassification of ORN severity.

Ontologies are "formal, explicit specifications of a shared conceptualizaton." In other words, they are machine-readable (formal), agreed upon by a group (shared), and an abstract model of a particular field of knowledge (conceptualization). Building an ontology for ORN would carry significant clinical and research advantages, including the ability to relate existing ORN staging/grading systems (i.e., a Notani stage X = Lyons stage Y).

What should you expect?

Using a remote, modified Delphi technique (i.e., iterative surveys for consensus formation), we will evaluate existing ORN definitions and scales for extraction and explicit definition of classes and relations that are essential to build an ORN ontology. A total of 3 to 4 "rounds" of surveys are expected to achieve Consortium consensus. Each survey is expected to take 15-20 minutes to complete and can be completed at your own pace within a 2-week timeframe from initial survey release. Time between each survey once completed is about 2-to-3 weeks to allow for data analysis and generation of the next survey.

Should you need a break while working on a survey while it's still active, you can return to where you left off by clicking on the same survey link from the email with the "round X" invite.

medRxiv preprint doi: https://doi.org/10.1101/2024.04.07.24305400; this version posted April 9, 2024. The copyright holder for this preprint (which was not certified by peer review) is the author/funder, who has granted medRxiv a license to display the preprint in perpetuity.
It is made available under a CC-BY 4.0 International license .

Friendly email reminders will be sent automatically every 4 days for up to 3 times to participants who have not completed the survey. Surveys will be automatically closed at 11:59 PM (CST) on the 14th day of survey release.

Rounds and Analysis:

This is Round 1. Round 1 includes three main sections:

1. Consortium Member Information and Acknowledgements

2. Review of Definitions and Classifications of ORN

3. Review of Radiation Ondontogram (RADMAP) draft designs

Subseqent rounds will include annonymized group feedback and statistics as well as further consolidation of questions for appraching consensus. Consensus statements in later Rounds will be "confirmed" by an agreement of 70% or more of Consortium members.

Thank you again for your willingness to serve as an expert!

CONSENT FOR PARTICIPATION IN THIS DELPHI

I have read the description of the study above, and I have decided to partcipate in the research project described. I understand that my continued participation throughout the entire Delphi study is crucial for robust analysis and consensus formation. I understand that my responses to survey questions will be collected via REDCap where data will be stored in a password-protected electronic format with access only available to Dr. Amy Moreno and her research team. My answers will remain anonymous to the entire expert panel and on future reports of this study. I also understand that I may refuse to answer any (or all) of the questions at this time or any other time. Should I wish to withdraw at any time or have my personal information removed from future publications, I can email Dr. Moreno with my specific request(s) at akmoreno@mdanderson.org

By clicking on the "Yes" button below, I certify that I have read the above information and voluntarily agree to participate in this ORAL Consortium study as an Expert.

◯ Yes
◯ No

Publications are expected related to this work. Please let us know to what degree you would like to be acknowledged. Please note that in order to be included in the 'ORAL Consortium' group authorship list, you must provide 'substantial contributions' (i.e., complete at least one survey to qualify for interpretation of data).

◯ No acknowledgments (keep me anonymous in publications and/or presentations)
◯ I'd like my name to be in the acknowledgement section only
◯ I'd like to be a co-author (included in the group authorship list)
◯ Unsure at the moment

medRxiv preprint doi: https://doi.org/10.1101/2024.04.07.24305400; this version posted April 9, 2024. The copyright holder for this preprint (which was not certified by peer review) is the author/funder, who has granted medRxiv a license to display the preprint in perpetuity. It is made available under a CC-BY 4.0 International license .

Please express your level of interest in being involved in manuscript preparations.

- ◯ I don't want to be involved in manuscript writing or review
- ◯ I want to be involved/updated on final manuscript review only
- ◯ I want to be involved/updated on initial manuscript drafting and final manuscript review

medRxiv preprint doi: https://doi.org/10.1101/2024.04.07.24305400; this version posted April 9, 2024. The copyright holder for this preprint (which was not certified by peer review) is the author/funder, who has granted medRxiv a license to display the preprint in perpetuity.
It is made available under a CC-BY 4.0 International license .

Member Information

What is your age?

____

What is your gender?

- ◯ Female
- ◯ Male
- ◯ Nonbinary
- ◯ Prefer not to say

What degree(s) do you currently have?

- ☐ DDS
- ☐ DMD
- ☐ MD
- ☐ DO
- ☐ PhD
- ☐ Other

Select the option that best describes your practice setting. Check all that apply

- ☐ Academic Medical Center (involved in graduate/medical education and/or research)
- ☐ Nonacademic Hospital
- ☐ Government-affiliated
- ☐ Independent/Private Practice
- ☐ Locum tenens
- ☐ Other

If other, please describe

____

Please write the name of your affiliated institution/practice as you desire it to be shown in publications.

(i.e., The University of Texas MD Anderson Cancer Center)

____

Please write your affiliated department (or specialty)

(i.e., Radiation Oncology)

____

For those in oral medicine, oral surgery, or dentistry, how would you best describe your specialty? Select all that apply

- ☐ General Dentist
- ☐ Periodontist
- ☐ Endodontist
- ☐ Prosthodontist
- ☐ Oral and Maxillofacial Surgeon (OMFS)
- ☐ Oral Medicine
- ☐ Other

If other, please describe

____

Have you completed any advanced education not described above?

- ◯ General Practice Residency (GPR)
- ◯ Advanced Education in General Dentistry (AEGD)
- ◯ Other
- ◯ None

If other, please describe

medRxiv preprint doi: https://doi.org/10.1101/2024.04.07.24305400; this version posted April 9, 2024. The copyright holder for this preprint (which was not certified by peer review) is the author/funder, who has granted medRxiv a license to display the preprint in perpetuity.
It is made available under a CC-BY 4.0 International license .

____

What country do you work in?

____

Select the option that best describes the community in which you work

- ◯ Urban (>75,000 population)
- ◯ Suburban (10,000-75,000)
- ◯ Rural (< 10,000)

Approximate years in clinical practice following completion of training

____

In your practice, approximately how many head and neck cancer patients do you evaluate and/or manage monthly (symptom management included)?

____

In your routine practice, what roles do you perform for patients receiving head and neck radiation therapy? Please select all that apply

- ☐ PRE-radiation dental evaluations
- ☐ Perform pRE-radiation invasive interventions (dental extraction, oral surgery, etc.)
- ☐ Design fluoride trays or stents
- ☐ POST-radiation dental evaluations
- ☐ Perform POST-radiation invasive interventions (dental extraction, oral surgery, etc.)
- ☐ Perform HN surgeries when indicated
- ☐ Plan and oversee radiation therapy
- ☐ QA radiation therapy plans
- ☐ Other

If other, please describe

____

Round 1 Defining And Classifying ORN

medRxiv preprint doi: https://doi.org/10.1101/2024.04.07.24305400; this version posted April 9, 2024. The copyright holder for this preprint (which was not certified by peer review) is the author/funder, who has granted medRxiv a license to display the preprint in perpetuity.
It is made available under a CC-BY 4.0 International license .

Please complete the survey below.

Thank you!

SECTION 1: Defining and Classifying Osteoradionecrosis (ORN)

This section focuses on review of existing definitions and staging/grading systems for ORN. Sample cases of ORN are classified by system. Data elements within staging/grading systems are extracted for review and rating. The utility of imaging in diagnosing ORN is also reviewed.

Have you ever evaluated and/or treated a patient with ORN?	◯ Yes<br>◯ No
On average, how many patients have you evaluated per year?	________________
On average, how many patients have you treated per year? Treatments include conservative medical management (i.e., Vitamin E), HBO therapy, debridement, or major surgery.<br><br>Please enter zero if you do not treat ORN.	________________
As an estimate, what percentage of your HNC patients treated with RT have had ORN?<br><br>Please enter a number between 0-100.	________________

Definitions for ORN

medRxiv preprint doi: https://doi.org/10.1101/2024.04.07.24305400; this version posted April 9, 2024. The copyright holder for this preprint (which was not certified by peer review) is the author/funder, who has granted medRxiv a license to display the preprint in perpetuity.
It is made available under a CC-BY 4.0 International license .

Example Definitions for ORN: While variations on extent of ORN, symptoms, and management are included in most staging systems, explicit definitions of ORN are not consistent (or may be absent). Please review the following published definitions for ORN. Time features (i.e., persistence of ORN) are bolded if present.

Authors (Year)

Diagnostic Criteria

Marx (1983)

An area greater than 1 cm of exposed bone in a field of irradiation that has failed to show and evidence of healing for at least 6 months

Beumer (1983)

Exposure of bone of the maxilla or mandible within the radiation treatment volume persisting for more than 3 months

Marx and Johnson (1987)

Exposure of nonviable bone which fails to heal without intervention

Epstein (1987)

An ulceration or necrosis of the mucous membrane, with exposure of necrotic bone for more than 3 months

Widmark (1989)

A non-healing mucous or cutaneous ulcer with denuded bone, lasting for more than 3 months

Harris (1992)

Exposed irradiated bone that has failed to heal over a period of 3 months in the absence of local tumor

Wong (1997)

A slow-healing radiation-induced ischemic necrosis of bone with associated soft tissue necrosis of variable extent occurring in the absence of local primary tumor necrosis, recurrence, or metastatic disease

Schwartz (2002)

A condition in which devitalized, irradiated bone becomes exposed through a wound in the overlying skin or mucosa. No tumor recurrence and it must persist for 3 to 6 months

Karagozoglu (2014)

Radiation induced necrosis of bone. Exposed bone with or without changes on plain radiograph having excluded presence of tumor tissue. Definitive ORN diagnosis if exposed bone persists for at least 1 month

medRxiv preprint doi: https://doi.org/10.1101/2024.04.07.24305400; this version posted April 9, 2024. The copyright holder for this preprint (which was not certified by peer review) is the author/funder, who has granted medRxiv a license to display the preprint in perpetuity.
It is made available under a CC-BY 4.0 International license .

Out of the definitions for ORN, which one do you feel is most representative for diagnosing ORN?	◯ Marx<br>◯ Beumer<br>◯ Marx and Johnson<br>◯ Epstein<br>◯ Widmark<br>◯ Harris<br>◯ Wong<br>◯ Schwartz<br>◯ Karagozoglu
Please state your level of agreement with the following statement: "Exposed bone" should be required for the diagnosis of ORN (i.e., radiological evidence with intact mucosa is not ORN)	◯ Strongly Agree<br>◯ Somewhat Agree<br>◯ Neutral<br>◯ Somewhat Disagree<br>◯ Strongly Disagree
The minimum length of exposed bone to qualify as ORN is explicity stated in Marx's definition (> 1cm). Do you agree that a formal definition for ORN should incorporate a specific numerical threshhold measurement of exposed bone?	◯ Strongly Agree<br>◯ Somewhat Agree<br>◯ Neutral<br>◯ Somewhat Disagree<br>◯ Strongly Disagree
Please state your level of agreement with the following statement: A time feature should be required for the diagnosis of ORN (i.e., persistence of exposed bone reported in months)	◯ Strongly Agree<br>◯ Somewhat Agree<br>◯ Neutral<br>◯ Somewhat Disagree<br>◯ Strongly Disagree
Example case 1: A patient in clinic is noted to have 1.5 cm of exposed bone 2 months after HN radiotherapy. Do you diagnose this patient with ORN during this visit?	◯ Yes<br>◯ No
For this case, what is the minimum amount of time (in months) from end of RT that the patient must have exposed bone to be considered as ORN?	__________________________________
Example case 2: A patient in clinic is noted to have 0.5 cm of exposed bone 7 months after HN radiotherapy. Do you diagnose this patient with ORN during this visit?	◯ Yes<br>◯ No

For each element below, please rate how important you think it is to use it in the formal definition of ORN.

Mucosal ulceration ______
Exposed bone ______
Exposed bone measurement (in mm) ______
Duration of exposure (i.e., weeks, months) ______
Radiologic findings (state presence or absence) ______

What additional core elements do you think are needed for diagnosing/defining ORN? Leave blank if none.	____

medRxiv preprint doi: https://doi.org/10.1101/2024.04.07.24305480; this version posted April 9, 2024. The copyright holder for this preprint (which was not certified by peer review) is the author/funder, who has granted medRxiv a license to display the preprint in perpetuity. It is made available under a CC-BY 4.0 International license .

Several staging/grading systems for classifying the extent of ORN have been published, and 15 will be reviewed in this section. Please note differences in criteria which may be related to clinical findings, radiological findings, disease progression, or response to therapy.

Additionally, 3 potential ORN case scenarios will be linked to each system. In consideration of efforts to reference a system's knowledge to another, please do your best at classifying these cases according to the specific staging/grading system in question. Select "unable to classify" only if very unsure on how to classify the patient.

Abbreviations: Hyperbaric oxygen therapy (HBO)

Have you ever used the Coffin (1983) classification system before?

Primary basis for classification: Clinical findings   Stages Description
Minor

Series of small sequestra which separate spontaneously over time (weeks or months)
Major

Bone necrosis extending entire thickness of the jaw; pathological fracture may be present

◯ Yes
◯ No

How effective do you find the Coffin system to be for classifying ORN?

◯ Very effective
◯ Somewhat effective
◯ Neutral
◯ Somewhat ineffective
◯ Very ineffective

For each case of potential ORN, please classify the patient using the Coffin system. Assume all completed HN RT.

Case 1: Patient with exposed bone (no measurement) not involving lower mandible, unknown duration. Pain present Case 2: Patient with 1.2 cm exposed bone for 4 months, pain present Case 3: Patient with 3 cm exposed bone with pathologic fracture. Unknown symptoms or duration

______ ______ ______

Comments on this staging system (if any)

____

Have you ever used the Marx (1983) staging system before?

Primary basis for classification: Response to therapy (i.e., HBO)   Stages Description
I

30 HBO dives -> re-examine -> 30 more dives if responder for mucosal recovery
II

Stage I non-responders; transoral alveolar sequestrectomy + HBO
III

Stage II non-responders OR initial presentation with either 1) pathologic fracture, 2) orocutaneous fistula, or 3) radiographic evidence of resportion to inferior border.

Treatment: Bone resection + HBO until mucosal recovery (or 60 dives)

IV

medRxiv preprint doi: https://doi.org/10.1101/2024.04.07.24305400; this version posted April 9, 2024. The copyright holder for this preprint (which was not certified by peer review) is the author/funder, who has granted medRxiv a license to display the preprint in perpetuity.
It is made available under a CC-BY 4.0 International license .

Additional HBO prior to bone graft

◯ Yes
◯ No

How effective do you find the Marx system to be for classifying ORN?

◯ Very effective
◯ Somewhat effective
◯ Neutral
◯ Somewhat ineffective
◯ Very ineffective

For each case of potential ORN, please classify the patient using the Marx system. Assume all completed HN RT.

Case 1: Patient with exposed bone (no measurement) not involving lower mandible, unknown duration. Pain present Case 2: Patient with 1.2 cm exposed bone for 4 months, pain present Case 3: Patient with 3 cm exposed bone with pathologic fracture. Unknown symptoms or duration

______ ______ ______

Comments on this staging system (if any)

____

Have you ever used the Morton and Simpson (1986) classification system before?

Primary basis for classification: Clinical findings and/or response over time Stage

Description

Minor

Ulceration with exposed bone and history of loss of bony spicules which healed spontaneously over a period of months

Moderate

Exposed bone and small sequestra limited in nature and healing spontaneously or with conservative treatment within 6-12 months

Major

Large areas of exposed bone with formation of large sequestra, possible fracture, and sinus formation

◯ Yes
◯ No

medRxiv preprint doi: https://doi.org/10.1101/2024.04.07.24305400; this version posted April 9, 2024. The copyright holder for this preprint (which was not certified by peer review) is the author/funder, who has granted medRxiv a license to display the preprint in perpetuity. It is made available under a CC-BY 4.0 International license .

How effective do you find the Morton system to be for classifying ORN?

◯ Very effective
◯ Somewhat effective
◯ Neutral
◯ Somewhat ineffective
◯ Very ineffective

For each case of potential ORN, please classify the patient using the Morton system. Assume all completed HN RT.

Case 1: Patient with exposed bone (no measurement) not involving lower mandible, unknown duration. Pain present Case 2: Patient with 1.2 cm exposed bone for 4 months, pain present Case 3: Patient with 3 cm exposed bone with pathologic fracture. Unknown symptoms or duration

______ ______ ______

Comments on this staging system (if any)

____

Have you ever used the Epstein (1987) staging system before?

Primary basis for classification: Clinical and/or radiographic findings (latter not defined) Stage

Description

Treatment

I

Healed, resolved ORN. No symptoms.

Ia: No pathologic fracture

Ib: Pathologic fracture

Surveillance, conservative therapy

Surgery (jaw reconstruction)

II

Chronic, persistent (> 3 months) ORN with stable symptoms (non-progressive paresthesia; pain-free or controlled)

IIa: No pathologic fracture

IIb: Pathologic fracture

Local wound care, conservative therapy

Surgery (jaw reconstruction)

III

Active progressive ORN with progressive symptoms.

IIIa: No pathologic fracture

IIIb: Pathologic fracture

medRxiv preprint doi: https://doi.org/10.1101/2024.04.07.24305400; this version posted April 9, 2024. The copyright holder for this preprint (which was not certified by peer review) is the author/funder, who has granted medRxiv a license to display the preprint in perpetuity.
It is made available under a CC-BY 4.0 International license .

Conservative therapy -> HBO and surgery if non-responders

Surgery (jaw reconstruction)

◯ Yes
◯ No

How effective do you find the Epstein system to be for classifying ORN?

◯ Very effective
◯ Somewhat effective
◯ Neutral
◯ Somewhat ineffective
◯ Very ineffective

For each case of potential ORN, please classify the patient using the Morton system. Assume all completed HN RT.

Case 1: Patient with exposed bone (no measurement) not involving lower mandible, unknown duration. Pain present Case 2: Patient with 1.2 cm exposed bone for 4 months, pain present Case 3: Patient with 3 cm exposed bone with pathologic fracture. Unknown symptoms or duration

______ ______ ______

Comments on this staging system (if any)

____

Have you ever used the Glanzmann and Gratz (1995) grading system before?

Primary basis for classification: Clinical findings and response to surgery

Grade	Description
1	Bone exposure without signs of infection and persisting for at least 3 months
2	Bone exposure with signs of infection or sequester and w/o signs of G3-5
3	Bone necrosis treated with mandibular resection with satisfactory result
4	Bone necrosis treated with mandibular resection with persisting problems
5	Death due to ORN

◯ Yes
◯ No

medRxiv preprint doi: https://doi.org/10.1101/2024.04.07.24305400; this version posted April 9, 2024. The copyright holder for this preprint (which was not certified by peer review) is the author/funder, who has granted medRxiv a license to display the preprint in perpetuity.
It is made available under a CC-BY 4.0 International license .

How effective do you find the Glantzmann system to be for classifying ORN?

◯ Very effective
◯ Somewhat effective
◯ Neutral
◯ Somewhat ineffective
◯ Very ineffective

For each case of potential ORN, please classify the patient using the Glantzmann system. Assume all completed HN RT.

Case 1: Patient with exposed bone (no measurement) not involving lower mandible, unknown duration. Pain present Case 2: Patient with 1.2 cm exposed bone for 4 months, pain present Case 3: Patient with 3 cm exposed bone with pathologic fracture. Unknown symptoms or duration

______ ______ ______

Comments on this staging system (if any)

____

Have you ever used the Clayman (1997) staging system before?

Primary basis for classification: Clinical findings Used Marx definition of ORN: "Nonhealing, nonseptic lesion of bone in which volume and density cannot be maintained by the hypocellular, hypovascular, hypoxic tissue which cannot adequately meet its metabolic demands." Type

Description

I

ORN presenting with bone lysis under intact gingiva or mucosa

II

Aggressive ORN. Soft tissue breakdown, bone exposed to saliva, and secondary contamination occurs; 'radiation osteomyelitis'

◯ Yes
◯ No

How effective do you find the Clayman system to be for classifying ORN?

◯ Very effective
◯ Somewhat effective
◯ Neutral
◯ Somewhat ineffective
◯ Very ineffective

For each case of potential ORN, please classify the patient using the Clayman system. Assume all completed HN RT.

Case 1: Patient with exposed bone (no measurement) not involving lower mandible, unknown duration. Pain present Case 2: Patient with 1.2 cm exposed bone for 4 months, pain present Case 3: Patient with 3 cm exposed bone with pathologic fracture. Unknown symptoms or duration

______ ______ ______

Comments on this staging system (if any)

medRxiv preprint doi: https://doi.org/10.1101/2024.04.07.24305400; this version posted April 9, 2024. The copyright holder for this preprint (which was not certified by peer review) is the author/funder, who has granted medRxiv a license to display the preprint in perpetuity.
It is made available under a CC-BY 4.0 International license .

____

Have you ever used the Store and Boysen (2000) staging system before?

Primary basis for classification: Clinical and radiographic findings (latter not defined) Stage

Description

0

Mucosal defects only (denuded bone intra-orally without any positive radiological signs)

I

Radiological evidence of bone necrosis with intact mucosa

II

Exposed non-vital bone and positive radiological signs, but without any sign of infection

III

Exposed bone, radiological evidence, extraoral fistula and infection

◯ Yes
◯ No

How effective do you find the Store staging system to be for classifying ORN?

◯ Very effective
◯ Somewhat effective
◯ Neutral
◯ Somewhat ineffective
◯ Very ineffective

For each case of potential ORN, please classify the patient using the Store system. Assume all completed HN RT.

Case 1: Patient with exposed bone (no measurement) not involving lower mandible, unknown duration. Pain present Case 2: Patient with 1.2 cm exposed bone for 4 months, pain present Case 3: Patient with 3 cm exposed bone with pathologic fracture. Unknown symptoms or duration

______ ______ ______

Comments on this staging system (if any)

____

Have you ever used the Schwartz and Kagan (2002) staging system before?

medRxiv preprint doi: https://doi.org/10.1101/2024.04.07.24305400; this version posted April 9, 2024. The copyright holder for this preprint (which was not certified by peer review) is the author/funder, who has granted medRxiv a license to display the preprint in perpetuity. It is made available under a CC-BY 4.0 International license .

Primary basis for classification: Clinical findings Definition for ORN: "A condition in which devitalized, irradiated bone becomes exposed through a wound in the overlying skin or mucosa. Such a wound must not be caused by tumor recurrence, or by tumor necrosis during radiation therapy, and it must persist without healing for 3 to 6 months." Associated with general treatment approach Stage

Description

I

Superficial involvement of the mandible only.

Soft-tissue ulceration is minimal. Only exposed cortical bone is necrotic.

II

Localized involvement of the mandible. The exposed cortical bone and also a portion of the underlying medullary bone are necrotic.

Division A: Soft-tissue ulceration is minimal

Division B: There is soft-tissue necrosis, including orocutaneous fistulation

III

Diffuse involvement of the mandible. The full-thickness segment of bone is involved, including the lower border. Pathologic fracture may occur.

Division A: Soft-tissue ulceration is minimal

Division B: There is soft-tissue necrosis, including orocutaneous fistulation

◯ Yes
◯ No

How effective do you find the Schwartz staging system to be for classifying ORN?

◯ Very effective
◯ Somewhat effective
◯ Neutral
◯ Somewhat ineffective
◯ Very ineffective

For each case of potential ORN, please classify the patient using the Schwartz system. Assume all completed HN RT.

Case 1: Patient with exposed bone (no measurement) not involving lower mandible, unknown duration. Pain present Case 2: Patient with 1.2 cm exposed bone for 4 months, pain present Case 3: Patient with 3 cm exposed bone with pathologic fracture. Unknown symptoms or duration

______ ______ ______

Comments on this staging system (if any)

____

medRxiv preprint doi: https://doi.org/10.1101/2024.04.07.24305400; this version posted April 9, 2024. The copyright holder for this preprint (which was not certified by peer review) is the author/funder, who has granted medRxiv a license to display the preprint in perpetuity. It is made available under a CC-BY 4.0 International license .

Have you ever used the Notani (2003) grading system before?

Primary basis for classification: Clinical findings (extent of lesion) Grades Description

I

ORN confined to alveolar bone

II

ORN limited to alveolar bone and/or the mandible above the level of the inferior alveolar canal

III

ORN extending under the level of the inferior alveolar canal or ORN with skin fistula and/or pathological fracture

◯ Yes
◯ No

How effective do you find the Notani system to be for classifying ORN?

◯ Very effective
◯ Somewhat effective
◯ Neutral
◯ Somewhat ineffective
◯ Very ineffective

For each case of potential ORN, please classify the patient using the Notani system. Assume all completed HN RT.

Case 1: Patient with exposed bone (no measurement) not involving lower mandible, unknown duration. Pain present Case 2: Patient with 1.2 cm exposed bone for 4 months, pain present Case 3: Patient with 3 cm exposed bone with pathologic fracture. Unknown symptoms or duration

______ ______ ______

Comments on this staging system (if any)

____

medRxiv preprint doi: https://doi.org/10.1101/2024.04.07.24305400; this version posted April 9, 2024. The copyright holder for this preprint (which was not certified by peer review) is the author/funder, who has granted medRxiv a license to display the preprint in perpetuity. It is made available under a CC-BY 4.0 International license .

Shaw et al. (2017) proposed a modified Notani ORN classification for use in clinical trials. As shown below, this system adds duration of exposed bone (6 month threshold) and minor bone spicules (MBS), defined as "not ORN" with a surface area of < 20mm^2.

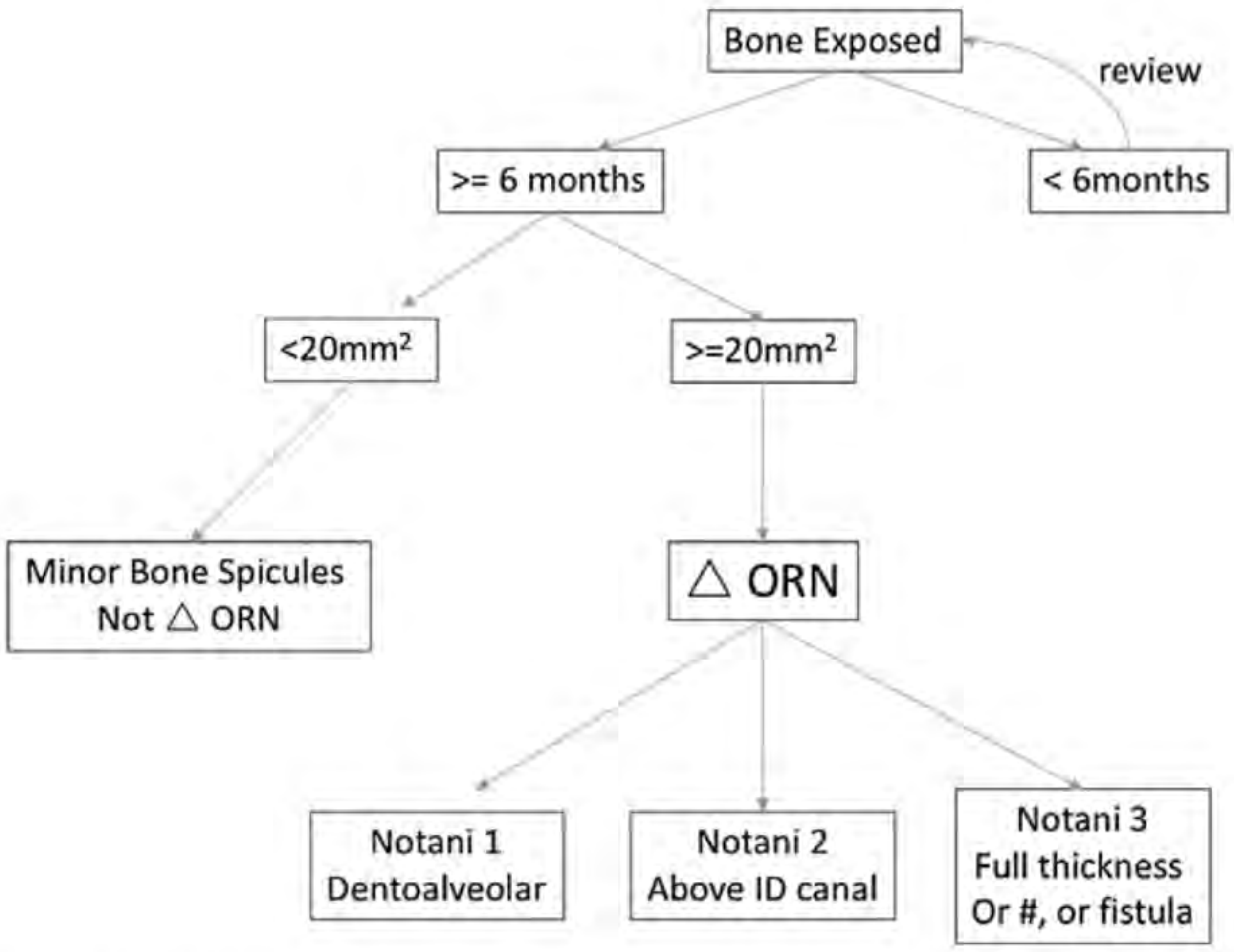

Fig. 4. Modified Notani ORN classification incorporating MBS. Not ORN: mucosal healing, or MBS <20 mm^2, or < 6 months. ORN: exposed bone ⩾20 mm^2 and ⩾6 months through oral mucosa or facial skin, excluding malignancy, within field of prior radiotherapy, further classified: Notani 1: ORN confined to alveolar bone. Notani 2: limited to the alveolar bone and/or above the level of the inferior alveolar canal. Notani 3: ORN under the lower part of the inferior alveolar canal, with fistula or bone fracture (Differentiation between Notani 1, 2 and 3 will usually be dependent on concurrent clinical and radiographic evidence).

Have you ever used the modified Notani classification before?

◯ Yes
◯ No

medRxiv preprint doi: https://doi.org/10.1101/2024.04.07.24305400; this version posted April 9, 2024. The copyright holder for this preprint (which was not certified by peer review) is the author/funder, who has granted medRxiv a license to display the preprint in perpetuity.
It is made available under a CC-BY 4.0 International license .

How effective do you find the modified Notani system to be for classifying ORN?

◯ Very effective
◯ Somewhat effective
◯ Neutral
◯ Somewhat ineffective
◯ Very ineffective

Comments on this staging system (if any)

____

Have you ever used the Tsai (2013) grading system before?

Primary basis for classification: Clinical findings or treatment needed Grade

Description

1

Minimal bone exposure with conservative management only

2

Minor debridement received

3

HBO needed

4

Major surgery required

◯ Yes
◯ No

How effective do you find the Tsai system to be for classifying ORN?

◯ Very effective
◯ Somewhat effective
◯ Neutral
◯ Somewhat ineffective
◯ Very ineffective

For each case of potential ORN, please classify the patient using the Tsai system. Assume all completed HN RT.

Case 1: Patient with exposed bone (no measurement) not involving lower mandible, unknown duration. Pain present Case 2: Patient with 1.2 cm exposed bone for 4 months, pain present Case 3: Patient with 3 cm exposed bone with pathologic fracture. Unknown symptoms or duration

______ ______ ______

Comments on this staging system (if any)

medRxiv preprint doi: https://doi.org/10.1101/2024.04.07.24305400; this version posted April 9, 2024. The copyright holder for this preprint (which was not certified by peer review) is the author/funder, who has granted medRxiv a license to display the preprint in perpetuity.
It is made available under a CC-BY 4.0 International license .

____

Have you ever used the Karagozoglu (2014) staging system before?

Primary basis for classification: Clinical and/or imaging findings (panoramic radiograph or periapical films recommended) Definition for ORN: "Radiation induced necrosis of bone. A diagnosis of ORN was rendered in the presence of exposed bone, with or without changes on plain radiograph, having excluded the presence of tumour tissue, either being a second primary or a recurrence. For the purposes of this study a definitive diagnosis of ORN has been made in case of presence of exposed bone for at least one month." Stage

Description

0

Exposure of mandibular bone for less than 1 month; no distinct changes on plain radiographs (panoramic radiograph or periapical film)

I

Exposure of mandibular bone for at least 1 month; no distinct changes on plain radiographs.

IA: Asymptomatic (no pain or presence of cutaneous fistulas)

IB: Symptomatic (pain or presence of cutaneous fistulas)

II

Exposure of mandibular bone for at least 1 month; distinct changes present on plain radiographs, but no involving the lower border of the mandible.

IIA: Asymptomatic

IIB: Symptomatic

III

Exposure of mandibular bone for at least 1 month; distinct changes on plain radiographs, involving the lower border of the mandible, irrespective of any other signs or symptoms

◯ Yes
◯ No

How effective do you find the Karragozoglu system to be for classifying ORN?

◯ Very effective
◯ Somewhat effective
◯ Neutral
◯ Somewhat ineffective
◯ Very ineffective

For each case of potential ORN, please classify the patient using the Karagozoglu system. Assume all completed HN RT.

Case 1: Patient with exposed bone (no measurement) not involving lower mandible, unknown duration. Pain present Case 2: Patient with 1.2 cm exposed bone for 4 months, pain present Case 3: Patient with 3 cm exposed bone with pathologic fracture. Unknown symptoms or duration

______ ______ ______

Comments on this staging system (if any):

medRxiv preprint doi: https://doi.org/10.1101/2024.04.07.24305400; this version posted April 9, 2024. The copyright holder for this preprint (which was not certified by peer review) is the author/funder, who has granted medRxiv a license to display the preprint in perpetuity.
It is made available under a CC-BY 4.0 International license .

____

Have you ever used the Lyons (2014) staging system before?

Primary basis for classification: Clinical findings and treatment approach Definition for ORN: Incorporated into the staging system. Authors acknowledged definitions for ORN which may or may not include bony exposure (i.e., Store classification of radiological evidence of bone necrosis without bony exposure). In the Lyons staging system, affected bone can be "damaged or exposed". Stage

Clinical Findings by Stage

Treatment Recommendation by Stage

I

< 2.5 cm length of bone affected (damaged or exposed); asymptomatic

Medical treatment only

II

>2.5 cm length of bone; asymptomatic, including pathological fracture or involvement of inferior dental nerve, or both

Medical treatment only unless there is dental sepsis or obviously loose, necrotic bone

III

>2.5 cm length of bone; symptomatic, but with no other features despite medical treatment

Consider debridement of loose or necrotic bone, and local pedicled flap

IV

>2.5 cm length of bone; pathological fracture, involvement of inferior dental nerve, or orocutaneous fistula, or a combination

Reconstruction with free flap if patient's overall condition allows

◯ Yes
◯ No

How effective do you find the Lyons system to be for classifying ORN?

◯ Very effective
◯ Somewhat effective
◯ Neutral
◯ Somewhat ineffective
◯ Very ineffective

For each case of potential ORN, please classify the patient using the Lyons system. Assume all completed HN RT.

Case 1: Patient with exposed bone (no measurement) not involving lower mandible, unknown duration. Pain present Case 2: Patient with 1.2 cm exposed bone for 4 months, pain present Case 3: Patient with 3 cm exposed bone with pathologic fracture. Unknown symptoms or duration

______ ______ ______

medRxiv preprint doi: https://doi.org/10.1101/2024.04.07.24305400; this version posted April 9, 2024. The copyright holder for this preprint (which was not certified by peer review) is the author/funder, who has granted medRxiv a license to display the preprint in perpetuity.
It is made available under a CC-BY 4.0 International license .

Comments on this staging system (if any)

____

Have you ever used the He (2015) staging system before?

Primary basis for classification: Clinical and imaging findings (both required for each stage) Definition for ORN: Authors acknowledged definitions for ORN which may or may not include bony exposure (i.e., Store classification of radiological evidence of bone necrosis without bony exposure). In the He staging system, ORN can be present without the precondition of bone exposure. Stage

Description

0

No evident signs or only osteolytic images on radiography; symptomatic (bone exposure or pain)

I

< 2cm radiographic lesion and:

B1S0: No mucosa or skin defect (intact mucosa)

B1S1: Intraoral mucosa defect or external skin fistula alone

B1S2: Through-and-through defect (both intraoral & skin defect)

II

>2 cm radiographic lesion and:

B2S0: No mucosa or skin defect (intact mucosa)

B2S1: Intraoral mucosa defect or external skin fistula alone

B2S2: Through-and-through defect (both intraoral & skin defect)

III

A pathologic fracture identified on radiographic and:

B3S0: No mucosa or skin defect (intact mucosa)

B3S1: Intraoral mucosa defect or external skin fistula alone

B3S2: Through-and-through defect (both intraoral & skin defect)

◯ Yes
◯ No

medRxiv preprint doi: https://doi.org/10.1101/2024.04.07.24305400; this version posted April 9, 2024. The copyright holder for this preprint (which was not certified by peer review) is the author/funder, who has granted medRxiv a license to display the preprint in perpetuity.
It is made available under a CC-BY 4.0 International license .

How effective do you find the He system to be for classifying ORN?

◯ Very effective
◯ Somewhat effective
◯ Neutral
◯ Somewhat ineffective
◯ Very ineffective

For each case of potential ORN, please classify the patient using the He system. Assume all completed HN RT.

Case 1: Patient with exposed bone (no measurement) not involving lower mandible, unknown duration. Pain present Case 2: Patient with 1.2 cm exposed bone for 4 months, pain present Case 3: Patient with 3 cm exposed bone with pathologic fracture. Unknown symptoms or duration

______ ______ ______

Comments on this staging system (if any)

____

Have you ever used the Common Terminology Criteria for Adverse Events (CTCAE) for ORN before?

Version 5.0 shown below for osteonecrosis of jaw Definition of Osteonecrosis: "A disorder characterized by a necrotic process occuring in the bone of the mandible." Grade Description
0 No ORN
1 Asymptomatic; clinical or diagnostic observations only; intervention not indicated
2 Symptomatic; medical intervention indicated (e.g., topical agents); limiting instrumental ADL
3 Severe symptoms; limiting self care ADL; elective operative intervention indicated
4 Life-threatening consequences; urgent intervention indicated
5 Death

◯ Yes
◯ No

How effective do you find CTCAE v5.0 to be for classifying ORN?

◯ Very effective
◯ Somewhat effective
◯ Neutral
◯ Somewhat ineffective
◯ Very ineffective

For each case of potential ORN, please classify the patient using the CTCAE criteria. Assume all completed HN RT.

Case 1: Patient with exposed bone (no measurement) not involving lower mandible, unknown duration. Pain present Case 2: Patient with 1.2 cm exposed bone for 4 months, pain present Case 3: Patient with 3 cm exposed bone with pathologic fracture. Unknown symptoms or duration

______ ______ ______

Are there any additional grading/staging systems for ORN that you want the Consortium to review?

◯ Yes
◯ No

If yes, please describe

Staging/grading data elements review

medRxiv preprint doi: https://doi.org/10.1101/2024.04.07.24305400; this version posted April 9, 2024. The copyright holder for this preprint (which was not certified by peer review) is the author/funder, who has granted medRxiv a license to display the preprint in perpetuity.
It is made available under a CC-BY 4.0 International license .

Please click on the link to find an overview of reported data elements per staging/grading systems.

You may want to have this opened separately while answering the next series of questions.

[Attachment: "ORN elements summarized-Table 1.png"]

medRxiv preprint doi: https://doi.org/10.1101/2024.04.07.24305400; this version posted April 9, 2024. The copyright holder for this preprint (which was not certified by peer review) is the author/funder, who has granted medRxiv a license to display the preprint in perpetuity. It is made available under a CC-BY 4.0 International license.

Please rate the level of importance for each. Consider items labeled as "very important" for mandatory documentation during follow ups on all HNC cases treated with RT, and/or for inclusion in an ORN ontology.

	Not important	Somewhat important	Very important
Exposed bone	◯	◯	◯
Extend of mandibular involvement	◯	◯	◯
Ulceration	◯	◯	◯
Bone spicules	◯	◯	◯
Sequestra	◯	◯	◯
Time (duration of exposure)	◯	◯	◯
Pathological fracture	◯	◯	◯
Orocutaneous fistula	◯	◯	◯
Sinus formation	◯	◯	◯
Signs of infection	◯	◯	◯
Symptoms	◯	◯	◯
Conservative medical therapy	◯	◯	◯
Hyperbaric oxygen therapy (HBO)	◯	◯	◯
Debridement/sequestrectomy	◯	◯	◯
Bone resection/reconstruction	◯	◯	◯
Exposed bone after therapy	◯	◯	◯
Granulation tissue after therapy	◯	◯	◯
Inflammation after therapy	◯	◯	◯
Healing (clinical improvement)	◯	◯	◯

Currently there are no spatial definitions for ORN (i.e., where is the 1.5cm of exposed bone located?).

If possible, which option(s) would you prefer for localizing ORN? Select all that apply.

- ☐ Auto-segmentation of the mandible (regardless of presence/absence of teeth)
- ☐ Based on tooth number
- ☐ Based on tooth type (i.e., molar vs. non-molar)
- ☐ Based on imaging
- ☐ Not required
- ☐ Other

If other, please describe

____

Are there additional elements that should be considered for classifying extent/severity of ORN? Leave blank if none.

____

medRxiv preprint doi: https://doi.org/10.1101/2024.04.07.24305400; this version posted April 9, 2024. The copyright holder for this preprint (which was not certified by peer review) is the author/funder, who has granted medRxiv a license to display the preprint in perpetuity.
It is made available under a CC-BY 4.0 International license.

Imaging correlates of ORN

Based on your knowledge and experience, how effective is CT imaging for diagnosing ORN?

◯ Very effective
◯ Somewhat effective
◯ Neutral
◯ Somewhat ineffective
◯ Very ineffective

In the past 12 months, have you used CT imaging to evaluate for ORN after RT?

◯ Yes
◯ No

What features of CT imaging do you associate with ORN? Select all that apply.

☐ Bone erosion
☐ Pathological fracture
☐ Soft tissue thickening
☐ Other

If other, please describe

____

Based on your knowledge and experience, how effective is MRI for diagnosing ORN?

◯ Very effective
◯ Somewhat effective
◯ Neutral
◯ Somewhat ineffective
◯ Very ineffective

In the past 12 months, have you used MRI to evaluate for ORN after RT?

◯ Yes
◯ No

What imaging sequences do you use for evaluating ORN?

☐ Dynamic contrast-enhanced (DCE)
☐ T1-weighted without contrast
☐ T1-weighted with contrast
☐ T2-weighted (T2W)
☐ Diffusion-weighted imaging (DWI)
☐ Other

If other, please describe

____

What quantitative MRI parameters do you think are important for assessment of risk, diagnosis, progression, and/or treatment response of ORN? Leave blank if none.

____

medRxiv preprint doi: https://doi.org/10.1101/2024.04.07.24305480; this version posted April 9, 2024. The copyright holder for this preprint (which was not certified by peer review) is the author/funder, who has granted medRxiv a license to display the preprint in perpetuity.
It is made available under a CC-BY 4.0 International license .

Based on your knowledge and experience, how effective are orthopantomograms (OPG) for diagnosing ORN?

◯ Very effective
◯ Somewhat effective
◯ Neutral
◯ Somewhat ineffective
◯ Very ineffective

In the past 12 months, have you used OPGs to evaluate for ORN after RT?

◯ Yes
◯ No

What features are you looking for on OPGs to diagnose ORN? Leave blank if none.

____

Are there additional imaging methods you use for diagnosing ORN?

◯ Yes
◯ No

If yes, please describe

____

Are there any additional references or questions on ORN that you would like the Consortium to review during round 2? If references, please provide a PMID or reference link.

____

Round 2 ORN

medRxiv preprint doi: https://doi.org/10.1101/2024.04.07.24305400; this version posted April 9, 2024. The copyright holder for this preprint (which was not certified by peer review) is the author/funder, who has granted medRxiv a license to display the preprint in perpetuity.
It is made available under a CC-BY 4.0 International license .

Please complete the survey below.

Thank you!

Dear Oral Consortium Members,

Thank you for your valuable responses to Round 1 of the ORN-RADMAP Delphi study!

We received similar feedback from many of you in that Round 1 posed important questions and challenges with our current ability to 1) diagnose ORN due to the lack of a consensus-based explicit definition and 2) classify various cases of potential ORN given 15+ existing staging/grading systems.

Using group feedback from Round 1, our main goals for Round 2 are:

Begin formulation of an explicit concept definition for ORN. Differentiate between features of bone-based disorders (for ORN staging) and potential modifiers of disease severity. Summarize group feedback for RADMAP with secondary questions on visualizations. Note: For Likert scale-type questions (i.e., strongly disagree to strongly agree), please try to minimize the use of 'neutral' in order to assist with consensus formation in future rounds. Also, some questions may sound repetitive but are useful for consensus processes. Thank you!

The focus of Round 3 will be confirmation of consensus-based ORN diagnostic criteria, and the build of a staging system/ontology based on Consortium-endorsed data elements.

medRxiv preprint doi: https://doi.org/10.1101/2024.04.07.24305400; this version posted [illegible] 9, 2024. The copyright holder for this preprint (which was not certified by peer review) is the author/funder, who has granted medRxiv a license to display the preprint in perpetuity. It is made available under a CC-BY 4.0 International license .

Table 1 summarizes the Consortium characteristics (for those who partially/fully completed Round 1 ORN/RADMAP surveys). Note: This table may be updated based on additional responses from Consortium members.

Table 1: Consortium Panel Characteristics

	Overall (N=59)
**Sex**	
Female	27 (45.8%)
Male	32 (54.2%)
Nonbinary	0 (0%)
Prefer not to say	0 (0%)
**Age (years)**	
Mean (SD)	46.2 (8.53)
Median [Min, Max]	46.0 [33.0, 68.0]
Missing	3 (5.1%)
**Specialty**	
HN Surgery	9 (15.3%)
Medical Oncology	4 (6.8%)
OMFS	11 (18.6%)
Oral Oncology/Medicine	8 (13.6%)
Physics	2 (3.4%)
Radiation Oncology	24 (40.7%)
Radiology	1 (1.7%)
**Practice Community**	
Urban (>75,000 population)	57 (96.6%)
Suburban (10,000-75,000)	2 (3.4%)
Rural (< 10,000)	0 (0%)
**Time in practice (years)**	
Mean (SD)	15.3 (8.02)
Median [Min, Max]	16.0 [1.00, 35.0]
**Approx. HNC patient caseload (per month)**	
Mean (SD)	47.7 (42.3)
Median [Min, Max]	45.0 [0, 200]
Missing	1 (1.7%)
**Have you evaluated/treated ORN?**	
Yes	55 (93.2%)
No	4 (6.8%)
**Avg. pts evaluated for ORN annually (n)**	
Mean (SD)	52.6 (182)
Median [Min, Max]	11.0 [0, 1320]
Missing	5 (8.5%)
**Avg. pts treated for ORN annually (%)**	
Mean (SD)	13.4 (21.8)
Median [Min, Max]	4.00 [0, 100]
Missing	4 (6.8%)
**Avg. HNC pts with ORN (%)**	
Mean (SD)	7.06 (4.84)
Median [Min, Max]	6.00 [0, 25.0]
Missing	5 (8.5%)

SECTION 1: FORMULATION OF AN EXPLICIT DEFINITION FOR ORN

medRxiv preprint doi: https://doi.org/10.1101/2024.04.07.24305400; this version posted April 9, 2024. The copyright holder for this preprint (which was not certified by peer review) is the author/funder, who has granted medRxiv a license to display the preprint in perpetuity. It is made available under a CC-BY 4.0 International license .

Group Feedback and Review of Existing Diagnostic Standards

• None of the 9 published ORN definitions were selected by even 25% of the group as the most representative for the disease entity (see Figure 1). The top 4 candidates were: Harris (n=13, 22%), Schwartz (n=13, 22%), Karagozoglu (n=10, 17%), and Wong (n=8, 14%).

From the top 4 definitions (Figure 1), 6 distinct features could be extracted with only 3 features included in all four: exposed [vs. necrotic] bone, RT-induced disorder [i.e., irradiated bone], and absence of tumor.

Table 2: ORN Features per existing definitions and based on free text comments from the group

Features	Count in Top 4 definitions	Favored in definition* (n, (%))
Exposed bone (vs. bone necrosis)	4	57 (100%)
Caused by radiation therapy	4	Not asked, but recommended in comments
Absence of tumor	4	Not asked, but recommended in comments
Time feature	3	54 (92%)
Imaging feature	1	56 (95%)
Soft tissue necrosis (ulceration)	1	49 (83%)

* Over 70% of the group rated this attribute as 'somewhat important' or 'very important'

Figure 1. Rating of Existing ORN Definitions

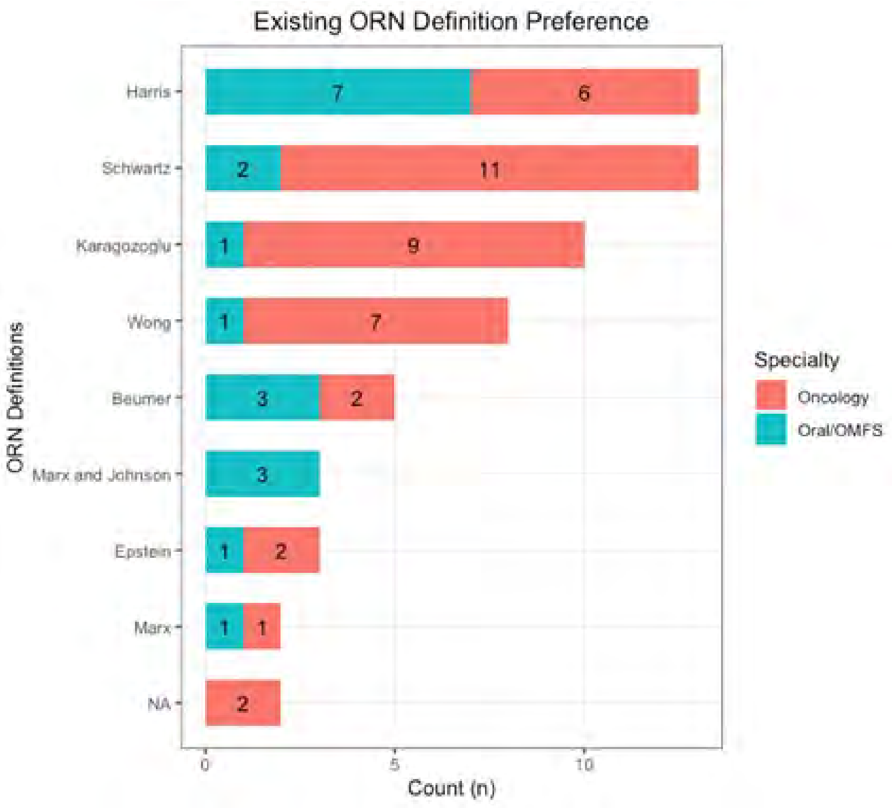

medRxiv preprint doi: https://doi.org/10.1101/2024.04.07.24305400; this version posted April 9, 2024. The copyright holder for this preprint (which was not certified by peer review) is the author/funder, who has granted medRxiv a license to display the preprint in perpetuity.
It is made available under a CC-BY 4.0 International license .

To date, there is no existing International Classification of Disease (ICD) diagnostic code specific to ORN. Other relevant internationally standardized medical classification terminologies and ontologies that do include codes for ORN include:

Medical Dictionary for Regulatory Activities (MedDRA) Systemized Nomenclature of Medicine- Clinical Terms (SNOMED-CT) These definitions can serve as a foundation to build the Consortium's explicit definition for ORN. Let's first review how each standard defines disorders such as osteonecrosis (ON) and ORN.

medRxiv preprint doi: https://doi.org/10.1101/2024.04.07.24305400; this version posted April 9, 2024. The copyright holder for this preprint (which was not certified by peer review) is the author/funder, who has granted medRxiv a license to display the preprint in perpetuity. It is made available under a CC-BY 4.0 International license .

MedDRA is a clinically-validated international terminology with a standardized hierarchy as described below (SOC --> HLGT --> HLT --> PT --> LLT). More specific diagnoses are typically coded as a "Lowest level term" or LLT.

MedDRA Hierarchy description from https://www.meddra.org/how-to-use/basics/hierarchy :

"The structure of MedDRA is very logical. There are five levels to the MedDRA hierarchy, arranged from very specific to very general. At the most specific level, called "Lowest Level Terms" (LLTs), there are more than 80,000 terms which parallel how information is communicated. These LLTs reflect how an observation might be reported in practice.... Each member of the next level, "Preferred Terms" (PTs) is a distinct descriptor (single medical concept) for a symptom, sign, disease diagnosis, therapeutic indication, investigation, surgical or medical procedure, and medical social or family history characteristic. Each LLT is linked to only one PT. Each PT has at least one LLT (itself) as well as synonyms and lexical variants (e.g., abbreviations, different word order). Related PTs are grouped together into "High Level Terms" (HLTs) based upon anatomy, pathology, physiology, aetiology or function. HLTs, related to each other by anatomy, pathology, physiology, aetiology or function, are in turn linked to "High Level Group Terms" (HLGTs). Finally, HLGTs are grouped into "System Organ Classes" (SOCs) which are groupings by aetiology (e.g., Infections and infestations), manifestation site (e.g., Gastrointestinal disorders) or purpose (e.g., Surgical and medical procedures)..." MedDRA Code Examples:

Code for osteonecrosis: 10031264 Code for osteoRADIOnecrosis: 10067352 Code for medication-related osteonecrosis of jaw: 10084881 Key takeaway points:

ORN is an LLT to 'radiation injury', 'bone disorders NEC (not elsewhere classified)', and 'necrosis and vascular insufficiency' Figure 2: MedDRA Hierarchy for ORN and ON

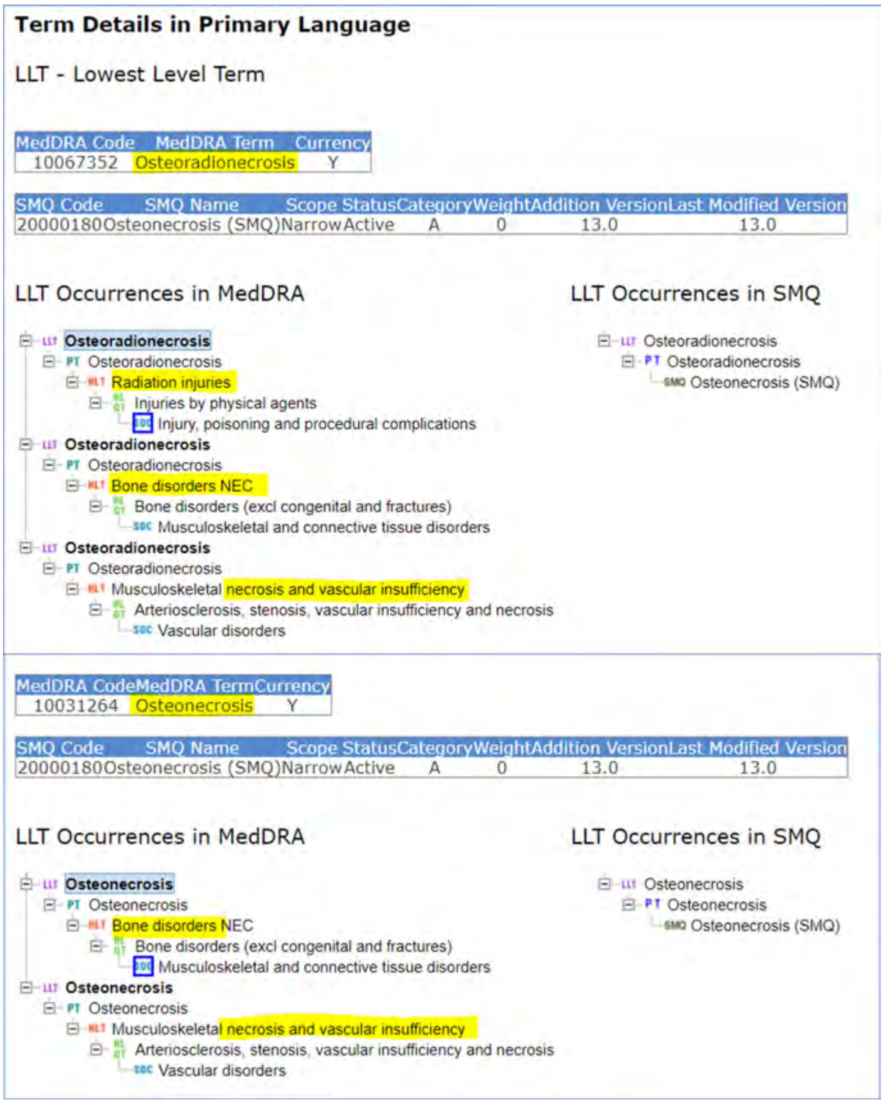

medRxiv preprint doi: https://doi.org/10.1101/2024.04.07.24305400; this version posted April 9, 2024. The copyright holder for this preprint (which was not certified by peer review) is the author/funder, who has granted medRxiv a license to display the preprint in perpetuity.
It is made available under a CC-BY 4.0 International license .

Do you agree that the Oral Consortium definition for ORN should be in alignment with the existing MedDRA hierarchical definition?

◯ Yes
◯ No

medRxiv preprint doi: https://doi.org/10.1101/2024.04.07.24305400; this version posted April 9, 2024. The copyright holder for this preprint (which was not certified by peer review) is the author/funder, who has granted medRxiv a license to display the preprint in perpetuity. It is made available under a CC-BY 4.0 International license .

SNOMED-CT is a comprehensive standard clinical terminology/ontology with concepts (aka: "a clinical meaning identified by a unique numeric identifier") formally defined by detailed relationships with other concepts.

Concepts and relationships, or attributes, are represented in SNOMED-CT via standardized concept diagrams, similar to the one below. For more information on diagram symbol definitions, please refer to this Diagramming Guideline: https://confluence.ihtsdotools.org/download/attachments/29951081/doc_DiagrammingGuideline_Current-en-US_INT_20140131.pdf?api=v2

SNOMED-CT Code Examples:

Code for osteonecrosis: 240196003 Code for osteoRADIOnecrosis: 109333005 Code for ORN of mandible: 109716001 Code for ORN of maxilla: 109715002 Key takeaway points:

SNOMED-CT allows for 'preferred' or 'acceptable' terms for the same concept such as 'radiation necrosis of bone' and 'osteoradionecrosis'. All share a 'finding site' in a bone structure and an associated morphology (attribute) of radiation injury WITH necrosis The 'causative agent (attribute)' is ionizing radiation and 'due to (attribute)' relationship is to exposure to ionizing radiation. Figure 3: SNOMED-CT Concept Diagrams for ORN of mandible and maxilla

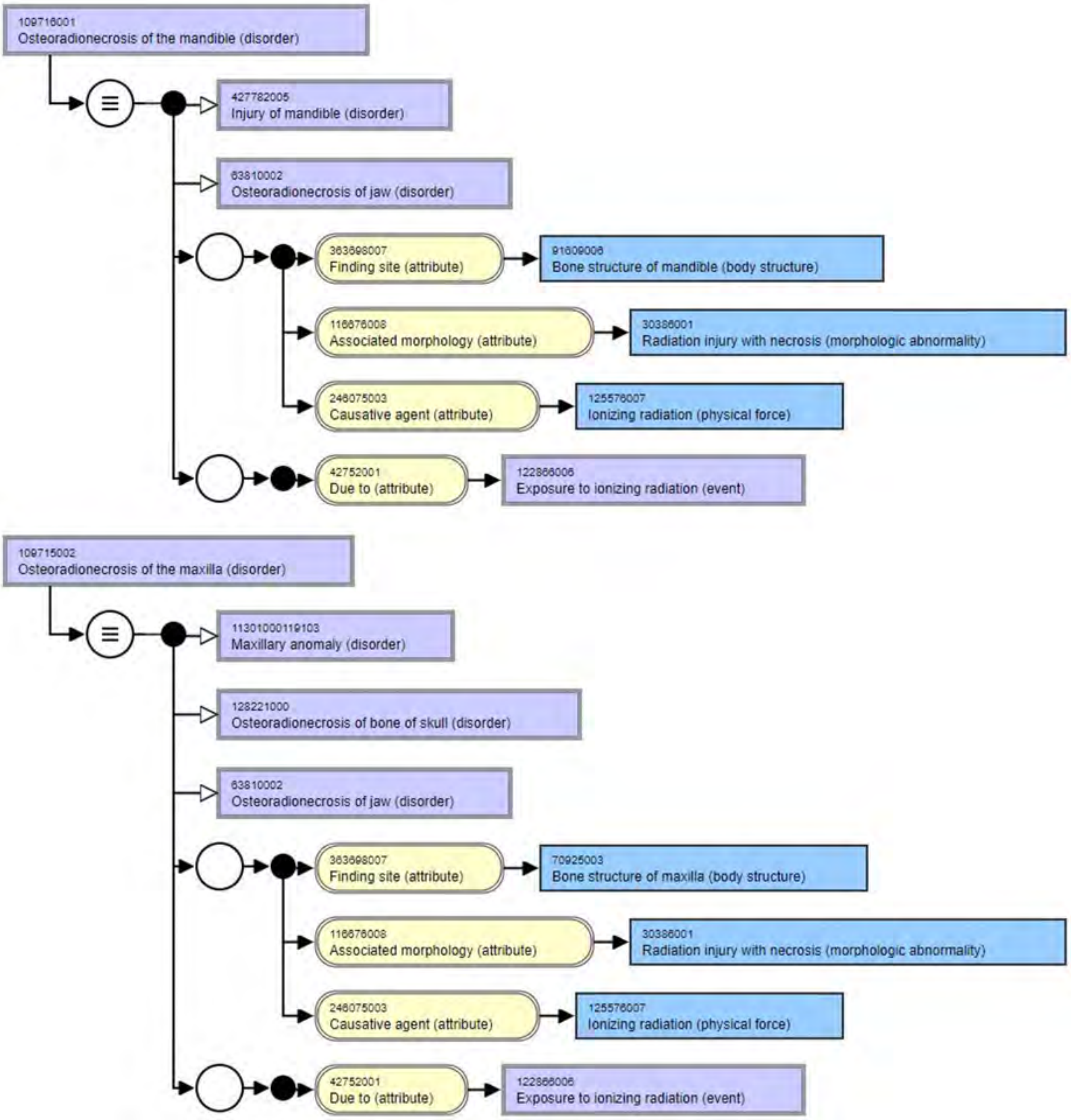

medRxiv preprint doi: https://doi.org/10.1101/2024.04.07.24305400; this version posted April 9, 2024. The copyright holder for this preprint (which was not certified by peer review) is the author/funder, who has granted medRxiv a license to display the preprint in perpetuity. It is made available under a CC-BY 4.0 International license .

Do you agree that the Oral Consortium definition for ORN should be in alignment with the existing SNOMED-CT concept definition for ORN?

◯ Yes
◯ No

International Classification of Disease - Clinical Modficiation (ICD-CM)
ICD diagnostic codes range from 3 to 7 characters with the first character always being an alpha (i.e. letter). Longer codes reflect more specific diagnoses. See Figure 4.

ICD-10-CM Code Examples:

Code for osteonecrosis: M87.9 Code for osteonecrosis, secondary necrosis (NEC), due to drugs: M87.10 Part of the definition for osteonecrosis (M87) from icd10data.com:
Clinical Information

A disorder characterized by necrotic changes in the bone tissue due to interruption of blood supply. Most often affecting the epiphysis of the long bones, the necrotic changes result in the collapse and the destruction of the bone structure. Death of a bone or part of a bone Death of a bone or part of a bone, either atraumatic or posttraumatic. Death of bone tissue caused by loss of blood supply to the bone. Death of bone tissue due to traumatic or nontraumatic causes. Of note, the ICD-10-CM Diagnosis Code 'M27.2' is often used for ORN of the jaw, which broadly captures 'inflammatory conditions of jaws'.

Reference: https://icd10cmtool.cdc.gov/?fy=FY2023&query=osteonecrosis

Figure 4: ICD-10-CM Index to ON

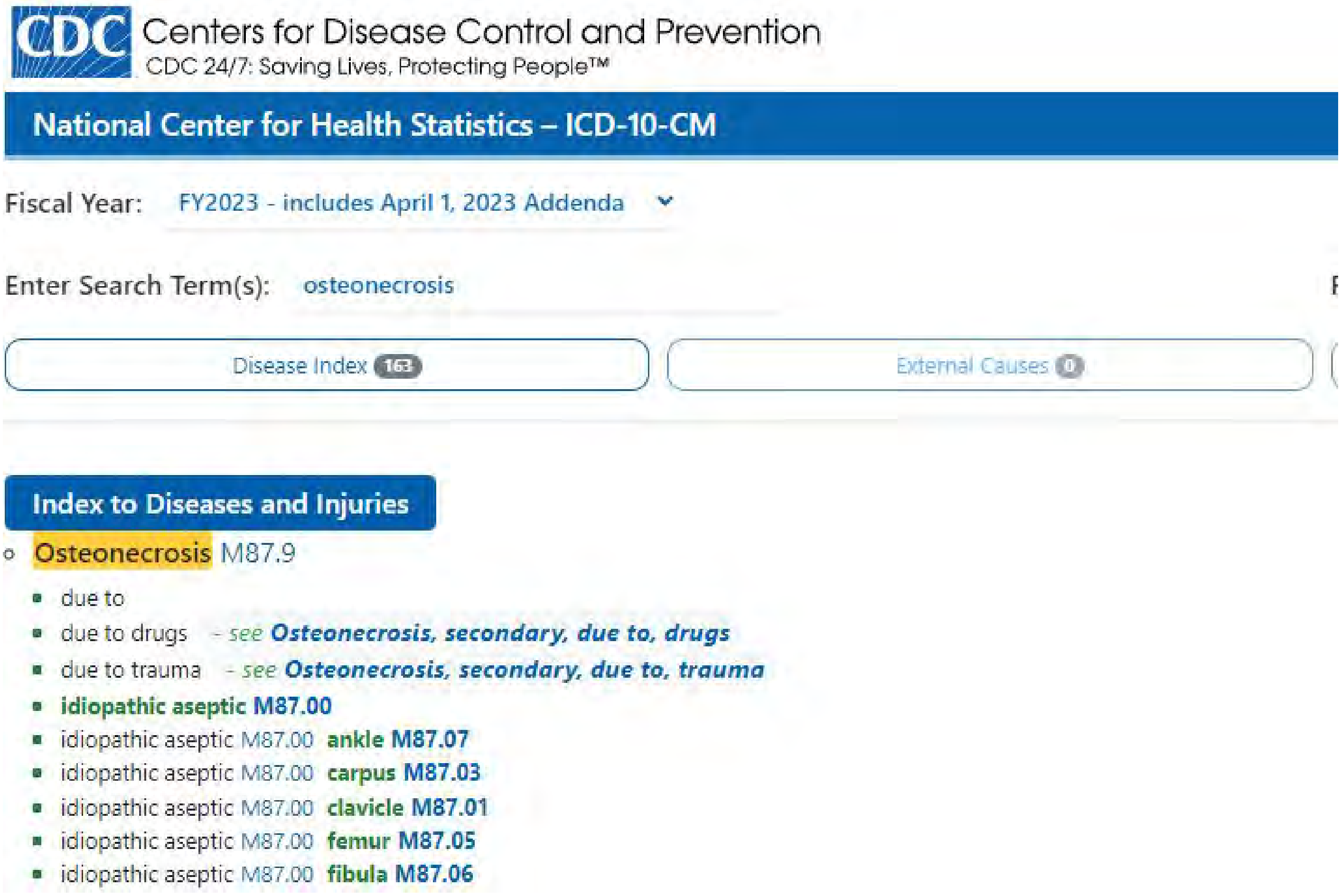

If building an ICD-10-CM code for ORN, do you agree that it should be nested under the M87 code for osteonecrosis, similar to ON secondary to drugs (i.e., M87.xx)?

◯ Yes
◯ No

Defining (Osteo)Necrosis

medRxiv preprint doi: https://doi.org/10.1101/2024.04.07.24305400; this version posted April 9, 2024. The copyright holder for this preprint (which was not certified by peer review) is the author/funder, who has granted medRxiv a license to display the preprint in perpetuity.
It is made available under a CC-BY 4.0 International license .

As necrosis is in several of the above definitions for ORN, consensus on the term 'necrosis' itself is needed.

Attaining consensus on the definition of necrosis itself is important when developing a formal definition for ORN. The National Cancer Institute (NCI) Dictionary of Cancer Terms describes osteoNECROSIS as the following:

"A condition in which there is a loss of blood flow to bone tissue, which causes the bone to die. It is most common in the hips, knees, shoulders, and ankles. It may be caused by long-term use of steroid medicines, alcohol abuse, joint injuries, and certain diseases, such as cancer and arthritis. It may also occur at some point in time after cancer treatment that included methotrexate, bisphosphonates, or corticosteroids. Also called aseptic necrosis, avascular necrosis, and ischemic necrosis."

Reference: https://www.cancer.gov/publications/dictionaries/cancer-terms/def/osteonecrosis

Do you agree with the NCI's definition for osteonecrosis?	○ Strongly agree<br>○ Somewhat agree<br>○ Neutral<br>○ Somewhat disagree<br>○ Strongly disagree
If you disagree, please state why	__________________________________
Do you agree with the definition of necrosis requiring loss/impairment of blood flow, or vascular insufficiency, or devascularization to any tissue?	○ Strongly agree<br>○ Somewhat agree<br>○ Neutral<br>○ Somewhat disagree<br>○ Strongly disagree
If you disagree, please state why	__________________________________
Are ALL cases of exposed bone also automatically necrotic bone?	○ Yes<br>○ No
Can ORN be diagnosed in cases with intact mucosa (i.e., diagnosis is supported by imaging findings)?	○ Yes<br>○ No

Disregarding a time feature, how would you classify the following scenarios related to a HN cancer patient treated with RT? For all cases, assume there is no evidence of active cancer in the evaluated site of irradiated bone. Questions are meant to vary on clinical and imaging findings.

Please read each scenario closely and answer to your best ability with the information provided.

CBCT shows lytic and sclerotic internal texture in mandible; exposed bone on exam. ______
CBCT shows lytic and sclerotic internal texture in mandible; no exposed bone on exam (mucosa intact). ______
CBCT shows presence of sequestrum; you can probe to bone. ______
CBCT shows periosteal reaction; you can probe to bone. ______
CBCT shows periosteal reaction; you cannot probe to bone. ______
Panorex report states "there is a sclerotic bone pattern within the alveolar processes of the maxillae, and slight periodontal ligament space widening involving the imaged maxillary dentition. The appearance is suggestive of changes related to therapeutic radiation exposure. " ______
A patient presents with a radiograph from their family dentist demonstrating bone loss to the apex of teeth 36, 37 in the absence of bone loss elsewhere in the mouth. You cannot probe to bone. ______
A patient presents with an image from their family dentist demonstrating bone loss to the apex of teeth 36, 37 in the absence of bone loss elsewhere in the mouth. You can probe to bone between the roots of the teeth. ______
A patient presents with an image from their family dentist demonstrating bone loss to the apex of teeth 36, 37 with radiographic regions of sclerosis and bone resorption extending to the inferior border of the mandible. There is no exposed bone. ______

medRxiv preprint doi: https://doi.org/10.1101/2024.04.07.24305400; this version posted April 9, 2024. The copyright holder for this preprint (which was not certified by peer review) is the author/funder, who has granted medRxiv a license to display the preprint in perpetuity.
It is made available under a CC-BY 4.0 International license .

Do you wish to provide additional case scenarios for the Consortium to review during Round 3?	◯ Yes<br>◯ No
Please elaborate on case scenarios to review.	__________________________
Repeated Q: Are ALL cases of exposed bone also automatically necrotic bone?	◯ Yes<br>◯ No
Repeated Q: Can ORN be diagnosed in cases with intact mucosa (i.e., diagnosis is supported by imaging findings)?	◯ Yes<br>◯ No

medRxiv preprint doi: https://doi.org/10.1101/2024.04.07.24305400; this version posted April 9, 2024. The copyright holder for this preprint (which was not certified by peer review) is the author/funder, who has granted medRxiv a license to display the preprint in perpetuity.
It is made available under a CC-BY 4.0 International license .

The 'Time Feature' and Refining the ORN Definition

The duration of ORN, while regarded as a highly important feature, remains controversial (i.e., when to use it and/or how to define useful parameters). Despite its inclusion in 6 of the 14 ORN staging/grading systems reviewed during Round 1, there is no consensus on the explicit definition for the time feature to declare a diagnosis of ORN (present/absent).

Round 1 Summary:

Harris, Schwartz, and Karagozoglu all reported different 'minimum' time periods of bone exposure in an irradiated field to diagnose ORN. Disagreement with the time feature was also seen in the group's response to Case 1 (1.5 cm of exposed bone for 2 months) whereby only 27 (46%) of members would diagnose the patient with ORN during that clinic visit. The majority (83%) agreed on diagnosing Case 2 with ORN, given a much longer time window (0.5 cm exposed bone for 7 months). When asked to provide an explicit time window (in months) for diagnosing ORN, 47% of panelists left this question blank while the remaining panelists listed 3 months (41%), 4 months (3%), or 6 months (9%). Additional considerations with regards to time:

Standardized diagnostic systems (i.e., ICD, MedDRA, SNOMED-CT, etc) do NOT include a time feature in their disease/disorder definitions. The true duration of necrotic bone (seen either clinically and/or on imaging) is difficult to measure as our observations heavily rely on the timing and frequency of patient visits (which can vary among providers). Consider the attached image scenario where clinical exams and imaging are performed every 3 months for a patient treated with RT. The asterisks represent suggested 'minimal duration of exposed bone' time windows after which one can 'diagnose' ORN. The red wording and time window represents changes occurring in between visits. Figure 5: Case Scenario

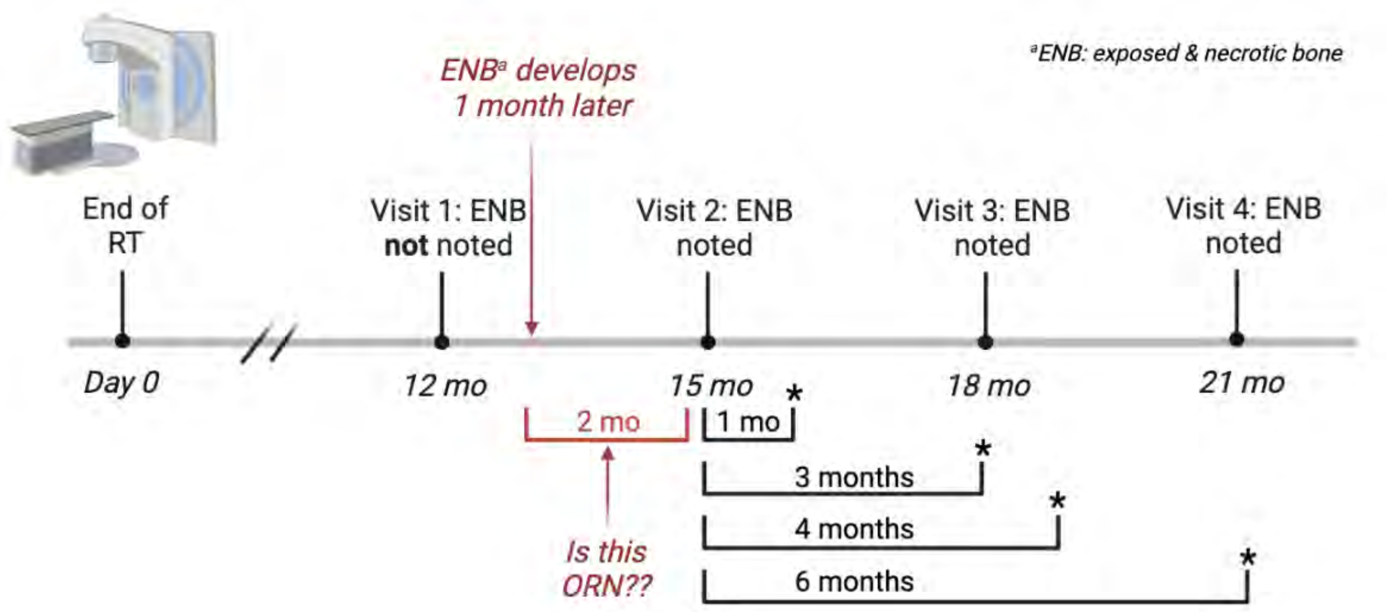

If the patient informs you during Visit #2 that he noticed exposed bone 2 months prior to that visit, would you:

- ◯ Diagnose ORN during visit 2
- ◯ Wait to diagnose ORN during visit 3
- ◯ Wait to diagnose ORN during visit 4
- ◯ None of the above

Please explain

____

With the information and group feedback provided above, please rate your level of agreement with the following statements:

The diagnostic criteria for a bone-based disorder like ORN can be met without the inclusion of a time feature.

______ A consensus-based staging system for ORN severity can be developed without the mandatory inclusion of a time feature (i.e., time features may be supplemental but not mandatory for describing ORN severity).

______ A time feature would be useful for assessing response to therapy. Response to therapy should be separated from a staging system for the disease entity, ORN.

medRxiv preprint doi: https://doi.org/10.1101/2024.04.07.24305400; this version posted April 9, 2024. The copyright holder for this preprint (which was not certified by peer review) is the author/funder, who has granted medRxiv a license to display the preprint in perpetuity.
It is made available under a CC-BY 4.0 International license .

Please comment on the question above, if possible.

Please comment on the question above, if possible.

Please comment on the question above, if possible.

Based on NCI's definition for osteonecrosis and existing diagnostic standards, please select which statement BEST defines ORN.

For reference again from NCI: "A condition in which there is a loss of blood flow to bone tissue, which causes the bone to die. It is most common in the hips, knees, shoulders, and ankles. It may be caused by long-term use of steroid medicines, alcohol abuse, joint injuries, and certain diseases, such as cancer and arthritis. It may also occur at some point in time after cancer treatment that included methotrexate, bisphosphonates, or corticosteroids. Also called aseptic necrosis, avascular necrosis, and ischemic necrosis."

- ◯ A condition in which there is a loss of blood flow to bone tissue, which causes the bone to die. It is caused by exposure to ionizing radiation.
- ◯ A condition in which there is a loss of blood flow to bone tissue, which causes the bone to die. It is caused by exposure to ionizing radiation and may occur at some point in time after radiation.
- ◯ A condition in which there is a loss of blood flow to bone tissue, which causes the bone to die. It is caused by exposure to ionizing radiation and occurs in the absence of active disease (i.e., cancer) in the site of bone death.
- ◯ A condition in which there is a loss of blood flow to bone tissue, which causes the bone to die. It is caused by exposure to ionizing radiation and may occur at some point in time after radiation and in the absence of active disease (i.e., cancer) in the site of bone death.

Any additional comments on the diagnostic criteria for ORN (not staging/grading)?

medRxiv preprint doi: https://doi.org/10.1101/2024.04.07.24305400; this version posted April 9, 2024. The copyright holder for this preprint (which was not certified by peer review) is the author/funder, who has granted medRxiv a license to display the preprint in perpetuity.
It is made available under a CC-BY 4.0 International license .

Staging Elements for Reporting Extent and Severity of ORN

During Round 1, a total of 15 staging/grading systems were reviewed. Members were asked to 1) state personal use of each system, 2) rate the utility of the system, and 3) apply the system to categorizing 3 different case scenarios.

Personal use: The top 3 systems used in practice were: CTCAE (n=41; 70%), Notani (n=18, 32%), and Marx (n=18, 31%).

Rating of effectiveness for classifying ORN: The top-rated systems, defined as 'somewhat/very important', for ORN were not in the top ones for personal use and included: Shwartz & Kagan (n=28, 52%), Karagozoglu (n=24, 49%), and Morton & Simpson (n=26, 48%). CTCAE, Notani, and Marx were considered effective by 46%, 42%, and 23% of respondants, repsectively.

Figure 6: Personal Use and Effectiveness Rating of Existing Staging/Grading Systems

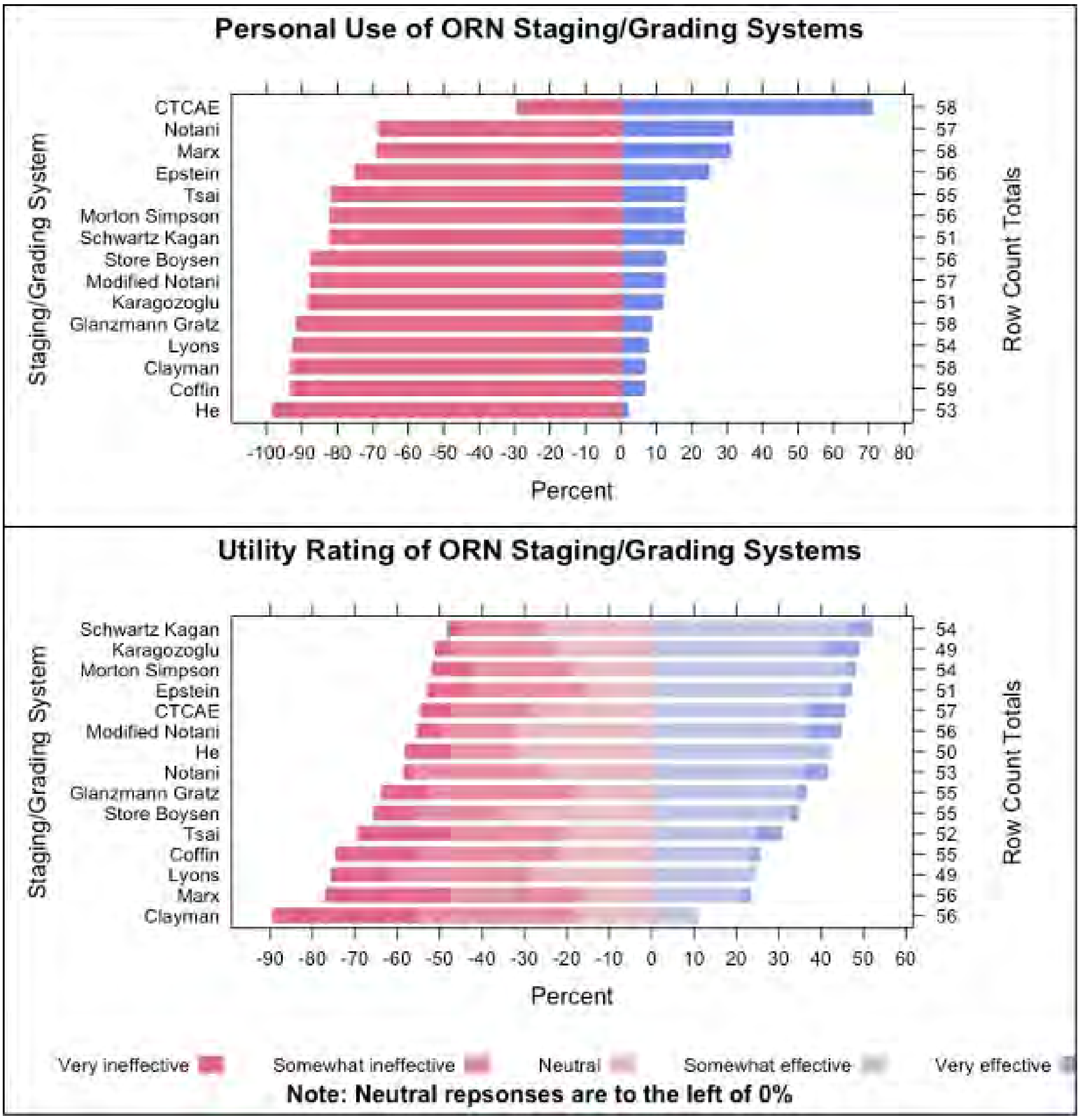

medRxiv preprint doi: https://doi.org/10.1101/2024.04.07.24305400; this version posted April 9, 2024. The copyright holder for this preprint (which was not certified by peer review) is the author/funder, who has granted medRxiv a license to display the preprint in perpetuity. It is made available under a CC-BY-4.0 International license .

Group feedback continued:
During Round 1, members were asked to categorize the following 3 cases using each staging/grading system.

Case 1: Patient with exposed bone (no measurement) not involving lower mandible, unknown duration. Pain present. Case 2: Patient with 1.2 cm exposed bone for 4 months, pain present. Case 3: Patient with 3 cm exposed bone with pathologic fracture. Unknown symptoms or duration. Fom a total of 59 responders:

The inability to classify cases was 57% for Case 1, 45% for Case 2, and 26% for Case 3. Pathological fracture (a finding in Case 3) was commonly classified as advanced ORN (major or stage III). Missing responses (i.e., no option was selected) was low at 3-4%. The distribution of case classification per system is shown in Figure 7. Overall, none of the existing systems were rated useful by more than 70% of the responders (the consensus threshold; top rated one was 52%), and the inconsistent classification of cases using these systems demonstrates an ongoing need for ORN data standardization.

Please click on the link below for Figure 7.

[Attachment: "Round1CasesbySystem.png"]

Additional grading systems recommended for review during Round 1:

In the comments section, four additional osteonecrosis / ORN systems were recommended for review. They include:

MRONJ (Medication-Related Osteonecrosis of the Jaws) LENT SOMA Scale Princess Margaret Cancer Center ORN Scoring System RTOG CTC (Common Toxicity Criteria) Summary:

MRONJ defines a non-exposed bone variant (stage 0), explicitly states 'exposed and necrotic bone or fistula' for advanced stages, and incorporates imaging findings. LENT-SOMA uses extent of exposed bone (2cm) or presence of limited sequestration or fracture for upgrading PMH defines grades as loss of mucosa with exposed bone requiring particular therapies for increasing time. Pathologic bone fracture is a grade 4. RTOG CTC: Vague and symptom based. In alignment with CTCAE Please review them in the attached file.

[Attachment: "ADDITIONAL GRADING SYSTEMS FOR REVIEW.docx"]

Have you used any of the osteonecrosis/ ORN systems listed?

- ☐ MRONJ (Medication-Related Osteonecrosis of the Jaws)
- ☐ LENT SOMA Scale
- ☐ Princess Margaret Cancer Center ORN Scoring System
- ☐ RTOG CTC (Common Toxicity Criteria)

Which of these systems, if any, do you find highly effective for staging ORN? Please select all that apply.

- ☐ MRONJ (Medication-Related Osteonecrosis of the Jaws)
- ☐ LENT SOMA Scale
- ☐ Princess Margaret Cancer Center ORN Scoring System
- ☐ RTOG CTC (Common Toxicity Criteria)

Clarification on the widely used Common Terminology Criteria for Adverse Events (CTCAE)
CTCAE is often used by the group (70%) but it is unclear if this is for staging (reporting ORN extent/severity) and/or toxicity grading (AEs after therapy).

For reference, the NCI states the following about CTCAE: it is a "descriptive terminology which can be utilized for Adverse Event (AE) reporting. A grading (severity) scale is provided for each AE term. An Adverse Event (AE) is any unfavorable and unintended sign (including an abnormal laboratory finding), symptom, or disease temporarily associated with the use of a medical treatment or procedure that may or may not be considered related to the medical treatment or procedure. An AE is a term that is a unique representation of a specific event used for medical documentation and scientific analyses."

Reference:
https://ctep.cancer.gov/protocoldevelopment/electronic_applications/docs/ctcae_v5_quick_reference_5x7.pdf

medRxiv preprint doi: https://doi.org/10.1101/2024.04.07.24305400; this version posted April 9, 2024. The copyright holder for this preprint (which was not certified by peer review) is the author/funder, who has granted medRxiv a license to display the preprint in perpetuity.
It is made available under a CC-BY 4.0 International license .

Given the CTCAE description, do you agree with the following statements?

CTCAE is a treatment-related toxicity (not disease) grading system ______
CTCAE can be used to stage ORN ______
CTCAE can be used in parallel with an ORN staging system for medical documentation after use of medical treatments or procedures ______

Rating of Data Elements in Existing ORN Staging/Grading Systems

During Round 1, the group was asked to rate the level of importance for each element extracted from all reviewed staging and grading systems. The results are shown in Figure 8.

The only elements (n=3) that were rated as 'somewhat/very important' by 100% of the group were all bone-related and included pathological fracture, exposed bone, and extent of exposed bone. Elements found to be of least importance when staging ORN included treatment (HBO, surgery, conservative therapy) and response to therapy (with the exception of persistent bone exposure). Comments from members: I think treatment can be considered separate from the classification as that may depend on what the patient or health care provider feels most comfortable with. A staging system based on primarily clinical factors is more useful, can consider including need for antibiotic treatment. If including response to therapy, would consider response to vitamin E/Trental as that defines conservative management vs surgical management. The classification should be independent on the response to treatment. There is limited evidence on the ideal treatment for ORN. It is therefore generally not appropriate to include these in a classification. The descriptors of extent of ORN should be sufficiently broad to be applicable to a wide range of cases (including 'pre-ORN') whilst also evaluating progression. Applying just to the mandible also limits external validity for ORN affecting maxilla or free flap. Figure 8

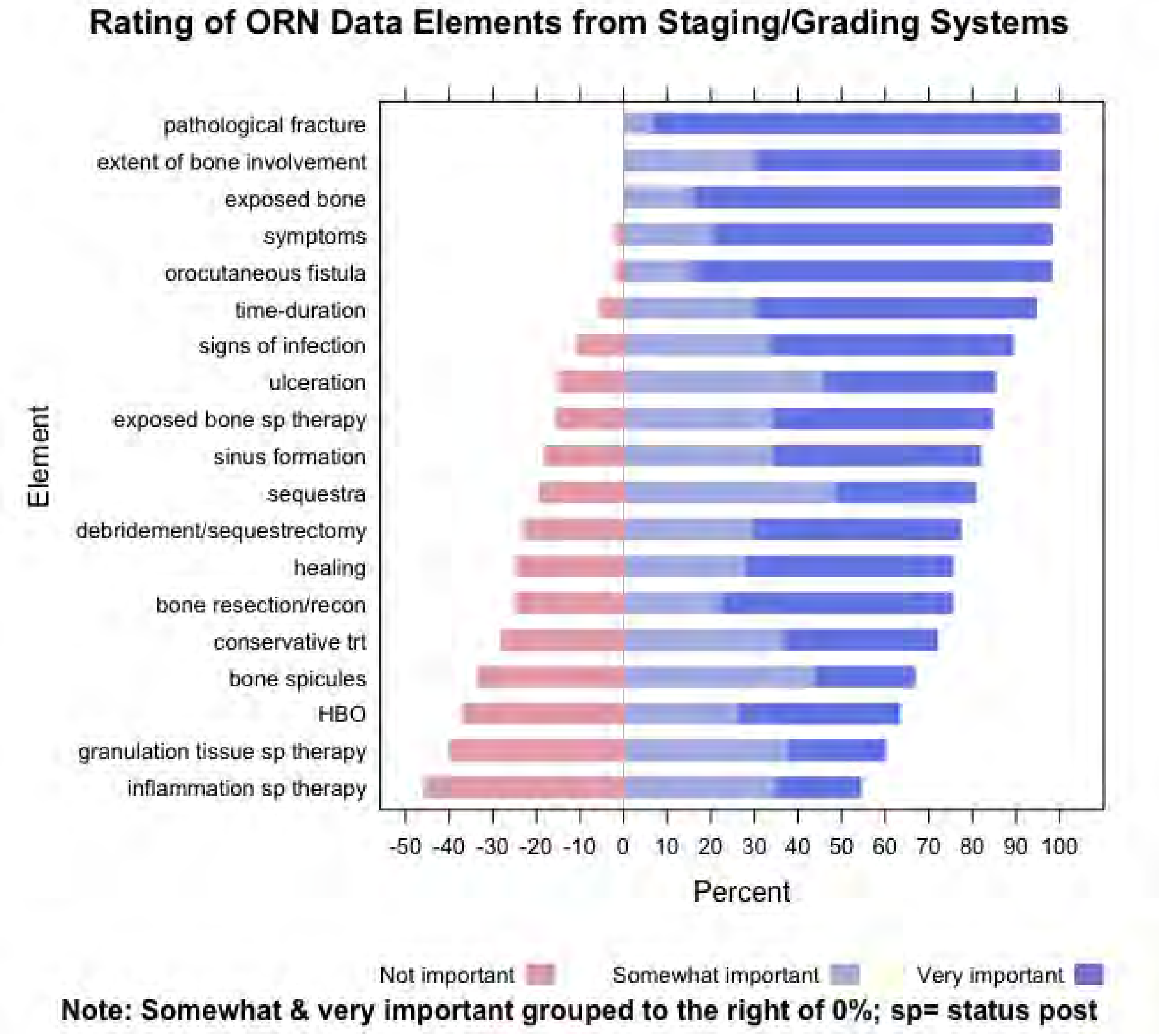

medRxiv preprint doi: https://doi.org/10.1101/2024.04.07.24305400; this version posted April 9, 2024. The copyright holder for this preprint (which was not certified by peer review) is the author/funder, who has granted medRxiv a license to display the preprint in perpetuity.
It is made available under a CC-BY 4.0 International license .

Element Clarification

If there is evidence of infection, then: ______
Since symptoms are not objective findings on clinical exam or imaging, they can be optional modifiers but not necessary factors for staging the extent/severity of ORN. ______
Since time is not an objective finding on clinical exam or imaging, it can be an optional modifier but not a necessary factor for staging the extent/severity of ORN. Note: this question relates to staging ORN, not diagnosing ORN which we previously covered. ______

Going through the objective elements rated within existing staging/grading systems, please state whether each most often represents early/limited ORN, advanced ORN, or neither (the latter suggesting that they should not be upstaging factors for ORN). Please leave a comment if desired for each.

Finding (based on exam and/or imaging)	Staging Influence	Comments
Pathologic fracture	______	______
Exposed and necrotic bone extent- limited to alveolar bone	______	______
Exposed and necrotic bone extent- beyond alveolar bone	______	______
Non-exposed bone but imaging findings limited to alveolar bone	______	______
Non-exposed bone but imaging findings beyond alveolar bone	______	______
Orocutaneous fistula	______	______
Signs of Infection	______	______
Mucosal ulceration	______	______
Sinus formation	______	______
Sequestra	______	______
Bone spicules	______	______

Extent of necrotic bone exposure revisited

Several systems include a vertical extent of disease (i.e., MRONJ, Notani) for staging and usually includes ORN limited to or extending beyond the alveolar bone/canal.

Should the extent of horizontal/superficial exposed & necrotic bone be considered and reported in ORN staging?

◯ Yes
◯ No

Please comment on how to report horizontal/superficial extent of exposed & necrotic bone ____________________________________

Other considerations on how to stage ORN based on exposed & necrotic bone extent? Please provide thresholds if possible, and include imaging type if relevant. ____

RADMAP: Radiation Dose Mapping to an Odontogram

medRxiv preprint doi: https://doi.org/10.1101/2024.04.07.24305400; this version posted April 9, 2024. The copyright holder for this preprint (which was not certified by peer review) is the author/funder, who has granted medRxiv a license to display the preprint in perpetuity.
It is made available under a CC-BY 4.0 International license .

A total of 55 panelists of the Consortium provided a response to the RADMAP (Radiation Odontogram) Section of Round 1. Consensus was defined as 70% of more agreement and percentages are based on the total count of responses per question.

The following table is a breakdown of the dental specialists within the oral consortium.

For reference to Round 1, the panel reviewed the following figure of a potential RADMAP report which displayed patient, cancer, and treatment information as well as the radiation odontogram and a snapshot of the RT plan.

Findings

The current layout for patient, cancer, and treatment is clear according to 85% (n=44) of the group. After reviewing comments left in Round 1, panelists pointed out a need to include chemotherapy history for the treatment information portion as well as any teeth missing or extracted on the RADMAP visual for each patient. In addition, panelists also made note of general material to add onto the patient information section.

If available, do you agree with adding smoking history/status to the patient information section of the RADMAP report?	◯ Yes<br>◯ No
If available, do you agree with adding gender to the patient information section to the RADMAP report?	◯ Yes<br>◯ No
If available, do you agree wtih adding chemotherapy to the treatment information section of the RADMAP report?	◯ Yes<br>◯ No
If available, do you agree with adding missing or extracted teeth to the RADMAP visual for each patient?	◯ Yes<br>◯ No
In your institution/practice, how easy is it for you to acquire data on missing teeth (i.e., tooth number)?	◯ Very easy<br>◯ Somewhat easy<br>◯ Neutral<br>◯ Somewhat difficult<br>◯ Very difficult

medRxiv preprint doi: https://doi.org/10.1101/2024.04.07.24305400; this version posted April 9, 2024. The copyright holder for this preprint (which was not certified by peer review) is the author/funder, who has granted medRxiv a license to display the preprint in perpetuity.
It is made available under a CC-BY 4.0 International license .

Radiation Dose Heat Maps

Group Feedback from Round 1:

89% (n=48) of the group agreed that a heat map over an odontogram (i.e., radiation odontogram) is a clinically useful visualization. Action: We will proceed with the build of a radiation odontogram   For cases with missing teeth, 96% (n=50) agreed that visualizing dose data to segmented/ tooth-bearing regions of the mandible and/or maxilla is helpful. When faxing or printing in only in black and white, 70% (n=38) agreed that grayscale colors may impede providers from accurately interpreting radiation dose distribution.   Action: We will provide an accompanying table with dose data.   80% (n=43) of the group responded that they are very confident or somewhat confident in their ability to interpret radiation doses on the radiation odontogram. The estimated mean and median dose delivered to tooth #31 was 14 Gy and 13 Gy, respectively (acceptable with one outlier of dmax ~35Gy). Action: No need for additional educational tools with RADMAP report

---

When comparing options A and B, 59% preferred option A whereas 32% (n=17) chose option B. A minority of 9% (n=5) opted for neither option stating that dose data to each tooth can be presented in the format of a table. Action: We will proceed with Option A as the background of the radiation odontogram.

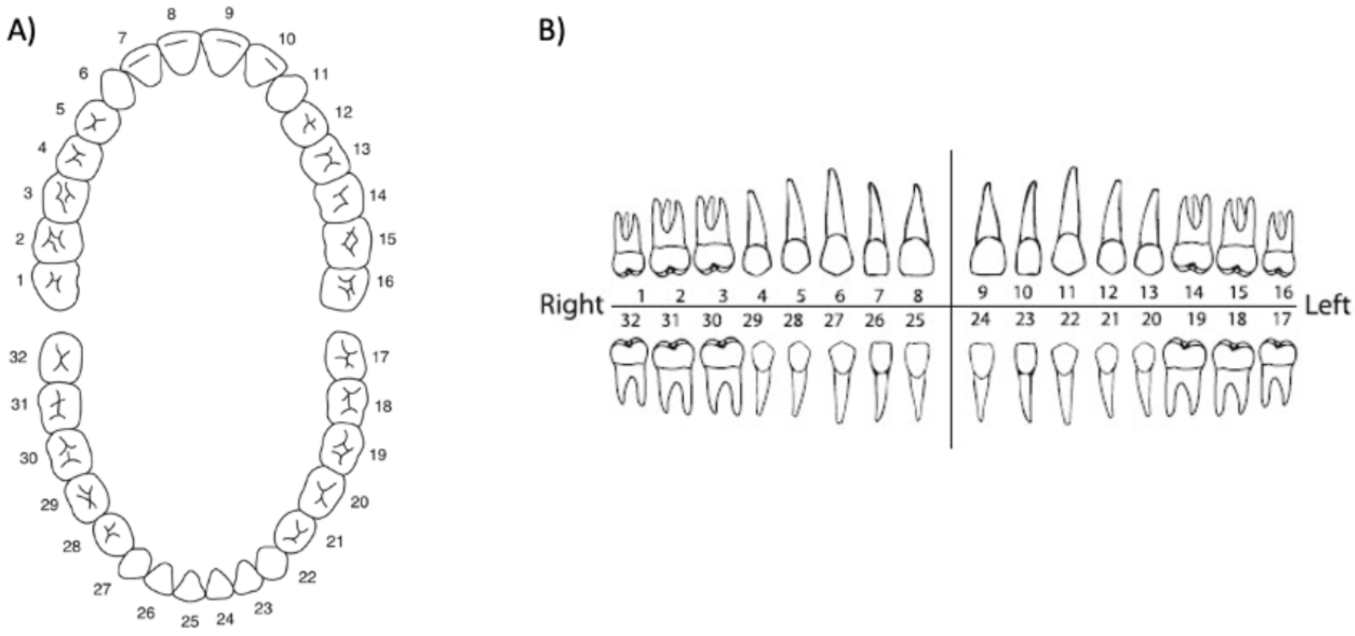

Evaluating dose data from an RT plan snapshot

96% (n=51) of the group reported seeing this type of radiation treatment plan snapshot before 72% (n=39) routinely request or review radiation therapy plans for evaluating post-RT care. When asked to evaluate the radiation dose delivered to tooth #8 (labeled A on image above), the mean, median and mode were 1000 cGy, 1000 cGy, 2000 cGy. Reported range: 0 to 2200 cGy.

Based on the findings above, how strongly do you agree with the following statement?

RT isodose lines on a treatment plan are potentially not as easy to interpret as a radiation odontogram heatmap/ table.

- ◯ Strongly Agree
- ◯ Somewhat Agree
- ◯ Neutral
- ◯ Somewhat Disagree
- ◯ Strongly Disagree

77% (n=41) of the group agreed that a 'heat map' of radiation dose on a table is useful.

medRxiv preprint doi: https://doi.org/10.1101/2024.04.07.24305400; this version posted April 9, 2024. The copyright holder for this preprint (which was not certified by peer review) is the author/funder, who has granted medRxiv a license to display the preprint in perpetuity.
It is made available under a CC-BY 4.0 International license .

If we were to apply a heat map, which parameter do you agree with incorporating? Please select all that apply.

☐ Use a single-hue color palette of varying saturation
☐ Use a diverging color palette
☐ Use a different color than red
☐ Other

Please comment on question above

____

In terms of presented dental dose data, dmean and dmax were ranked the highest in preference by 56% of the group (total). See the breakdown below.

Dosimetric Parameter	Count (%)
Mean dose	30 (31%)
Max point dose	24 (25%)
Dose going to 0.03cc (D0.033cc)	17 (18%)
Dose going to 95% of the tooth (D95)	15 (16%)
Dose going to 50% of the tooth (D50)	10 (10%)

If only given one option, which metric is desired the MOST?

◯ Mean dose (dmean) to each tooth/ tooth-bearing region
◯ Max dose (dmax) to each tooth / tooth-bearing region

Any last questions or comments for Round 2?

medRxiv preprint doi: https://doi.org/10.1101/2024.04.07.24305400; this version posted April 9, 2024. The copyright holder for this preprint (which was not certified by peer review) is the author/funder, who has granted medRxiv a license to display the preprint in perpetuity.
It is made available under a CC-BY 4.0 International license .

THE UNIVERSITY OF TEXAS
MD Anderson ~~Cancer~~ Center
Making Cancer History®

Intro

ORAL Consortium Round 3 Survey

This round of the ORAL Consortium Delphi study will build upon the prior round by providing group feedback on the definition of osteoradionecrosis (ORN), the diagnostic criteria for ORN, and the utility and categorization of clinical and radiographic features associated with ORN.

The goal of Round 3 is to focus on the nuances of the definition of ORN as well as classification criteria (clinical and/or radiographic) for ORN staging. **Any topics that have met consensus based on the pre-defined consensus threshold of 70% will be reported in blue font.**

ORN Definition

The Definition of ORN

In Round 2, **83%** (43/52) and **85%** (45/53) of panelists agreed that the ORAL Consortium's definition for ORN should align with existing MedDRA and SNOMED-CT ORN definitions, respectively, and **80%** (48/60) strongly/somewhat agreed with NCI's definition for osteonecrosis. **87% (52/60)** strongly/somewhat agreed that the term 'vascular insufficiency' or 'loss/impairment of blood flow' should be

medRxiv preprint doi: https://doi.org/10.1101/2024.04.07.24305400; this version posted April 9, 2024. The copyright holder for this preprint (which was not certified by peer review) is the author/funder, who has granted medRxiv a license to display the preprint in perpetuity.
It is made available under a CC-BY 4.0 International license .

included in definitions of necrosis (i.e., ORN).

To review these definitions again, click on the links below.
MedDRA
SNOMED-CT
NCI

Consensus: The Consortium's definition for ORN will reflect features in these existing definitions, including:

1. Bone disorder
2. Radiation injury and/or caused by ionizing radiation
3. Loss of blood flow or vascular insufficiency AND findings of bone death/necrosis

Time Feature

To refine the nuances of time and its relevance as a **diagnostic feature** for ORN, the case below was presented to the panel along with a question of when they would diagnose the patient with ORN. **79%** (44/56) of panelists would diagnose this patient during visit 2 (the first time observing features of exposed and necrotic bone) while 0 panelists would wait 6 months to diagnose during visit 4.

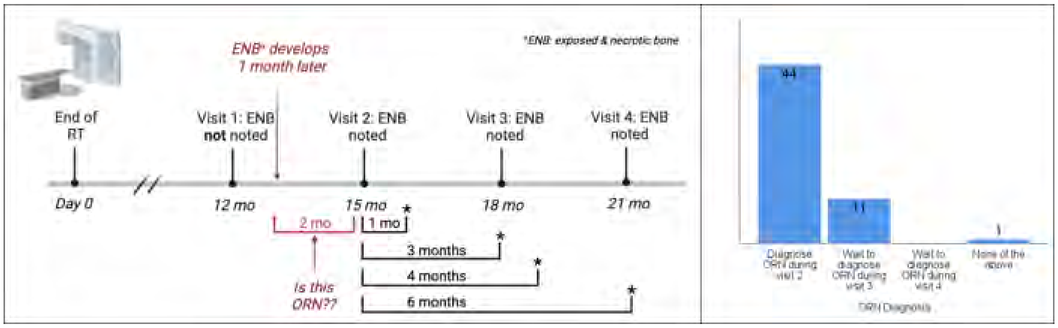

A time feature is not present in existing diagnostic codes for

medRxiv preprint doi: https://doi.org/10.1101/2024.04.07.24305400; this version posted April 9, 2024. The copyright holder for this preprint (which was not certified by peer review) is the author/funder, who has granted medRxiv a license to display the preprint in perpetuity.
It is made available under a CC-BY 4.0 International license .

ORN, and **70%** of the panel strongly/somewhat agreed that a diagnosis of ORN could be met without a time feature.

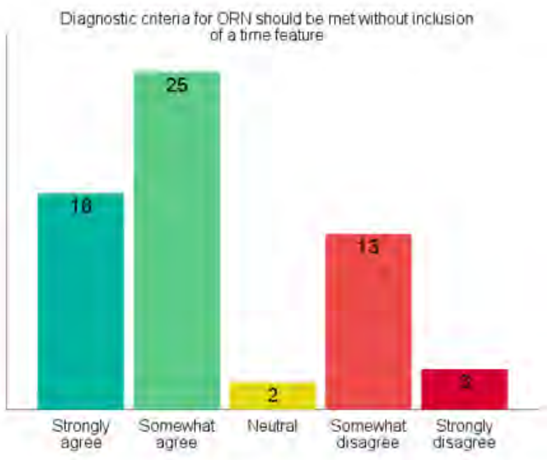

Consensus: While potentially valuable to report and reflect the duration of non-healing changes observed in bone after RT, time is not a defining diagnostic feature for ORN.

Consensus Statements

- **87%** do not consider all cases of exposed bone to automatically also be necrotic bone (i.e., minor bone spicules)
- **93%** agreed that a diagnosis of ORN can be made with **intact** mucosa

Consensus: Not all cases of exposed bone are automatically considered to be necrotic bone.
Consensus: A diagnosis of ORN can be made in a patient treated with RT and with intact mucosa (i.e., no bone exposure) if there is supporting radiographic evidence of bone death/necrosis.

medRxiv preprint doi: https://doi.org/10.1101/2024.04.07.24305400; this version posted April 9, 2024. The copyright holder for this preprint (which was not certified by peer review) is the author/funder, who has granted medRxiv a license to display the preprint in perpetuity.
It is made available under a CC-BY 4.0 International license .

Working ORN Definition

The working definition for ORN selected by **73%** (41/56) of the panel during Round 2 is:
A condition in which there is a loss of blood flow to bone tissue which causes the bone to die. It is caused by ionizing radiation and may occur at some point in time after radiation and in the absence of active disease (i.e., cancer) in the site of bone death.

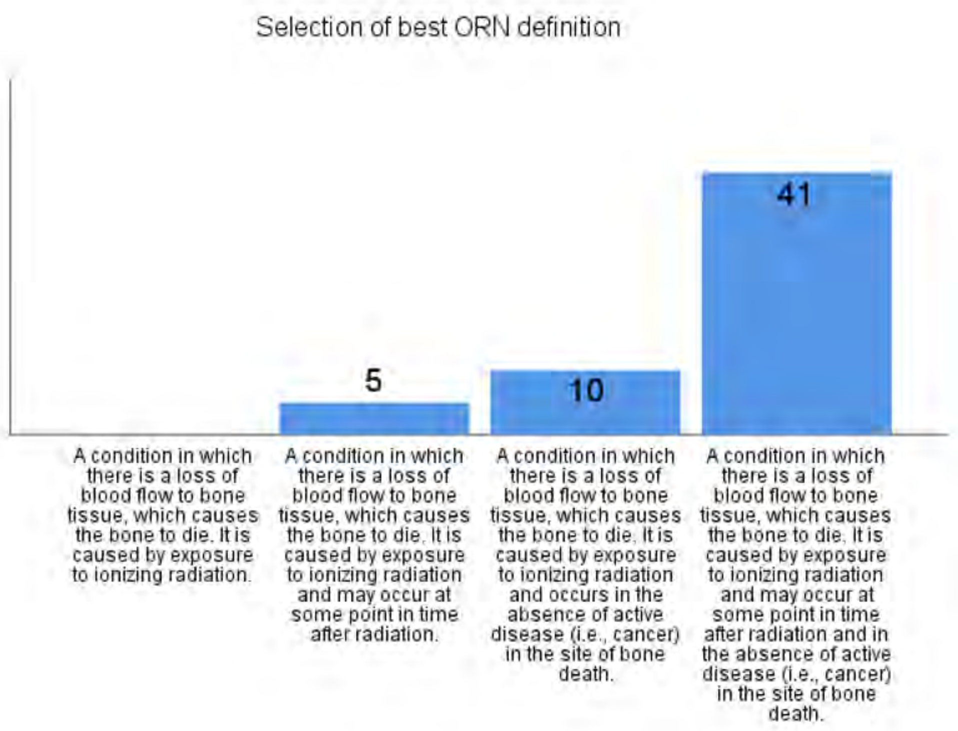

Comments by panelists (and comments by moderators) are listed below. Select any you agree should definitely be considered for addition in the official ORN definition.

medRxiv preprint doi: https://doi.org/10.1101/2024.04.07.24305400; this version posted April 9, 2024. The copyright holder for this preprint (which was not certified by peer review) is the author/funder, who has granted medRxiv a license to display the preprint in perpetuity.
It is made available under a CC-BY 4.0 International license .

	Definitely should be considered
**It should include the presence of exposed bone.** 93% of the panel agreed ORN can be diagnosed in the presence of intact mucosa. Can include with an 'AND/OR' statement (i.e., exposed bone and/or radiographic evidence of bone death).	○
**[Include] It may or may not be painful.** This is a neutral statement (pain is neither inclusive or exclusive criteria).	○
**[Include] Accompanying signs and triggering events may also be important to say that osteonecrosis is due to RT.** The phrase 'accompanying signs' is ambiguous, and the working definition already includes 'it is caused by exposure to ionizing radiation'.	○
**[Include] Progressive bone changes.** This is non-specific and would not include cases of ORN with stable findings. Specified progressive bone changes may be an upstaging, not diagnostic, feature.	○
**Include imaging criteria.** This statement is non-specific, and ORN can be diagnosed clinically. Can include an 'AND/OR' statement (i.e., ... and/or radiographic evidence of bone death).	○
**ORNJ is also observed in the jaw bones of patients with radiotherapy to the head and neck region, and it must be included in the definition of ORN.** The working ORN definition applies to any bone site exposed to ionizing radiation, including jaw bones.	○
**Exclude active diagnosis from the diagnosis.** The top 2 ORN definitions selected by the panel during Round 2 included the phrase "occurs in the absence of active disease (i.e., cancer)."	○

Revisions to the working ORN definition are shown below. Choose the **best** and **second best** definition and drag them to the appropriate box on the right (only one per box).

	Best option	Second best option
**Working:** A condition in which there is a loss of blood flow to bone tissue, which causes the bone to die. It is caused by exposure to ionizing radiation and may occur at some point in time after radiation and in the absence of active disease (i.e., cancer) in the site of bone death.		

medRxiv preprint doi: https://doi.org/10.1101/2024.04.07.24305400; this version posted April 9, 2024. The copyright holder for this preprint (which was not certified by peer review) is the author/funder, who has granted medRxiv a license to display the preprint in perpetuity.
It is made available under a CC-BY 4.0 International license .

v1: A condition in which there is a loss of blood flow to bone tissue, which causes the bone to die. It is caused by exposure to ionizing radiation and occurs in the site of bone death.

v2: A condition in which there is a loss of blood flow to bone tissue, which causes the bone to die. Findings of bone death may be clinical (i.e., exposed bone) and/or radiographic (i.e., sclerosis, pathologic fracture). It is caused by exposure to ionizing radiation and may occur at some point in time after radiation and in the absence of active disease (i.e., cancer) in the site of bone death.

v4: A condition in which there is a loss of blood flow to bone tissue, which causes the bone to die. Findings of bone death may be clinical (i.e., exposed bone) and/or radiographic (i.e., sclerosis, pathologic fracture). It is caused by exposure to ionizing radiation and occurs in the site of bone death.

Staging Features

medRxiv preprint doi: https://doi.org/10.1101/2024.04.07.24305400; this version posted April 9, 2024. The copyright holder for this preprint (which was not certified by peer review) is the author/funder, who has granted medRxiv a license to display the preprint in perpetuity.
It is made available under a CC-BY 4.0 International license .

Consensus on CTCAE

CTCAE is the most commonly used grading system for ORN by the panel but was only rated 4th in terms of utility (Round 1). Three questions were asked for clarification on how to use CTCAE.

1. **CTCAE can be used to stage ORN.** Only **52%** agreed.
2. **CTCAE is a toxicity (not disease grading system).** High consensus with **90%** agreeing.
3. **CTCAE can be used in parallel with an ORN staging system after use of treatments.** High consensus with **95%** agreeing.

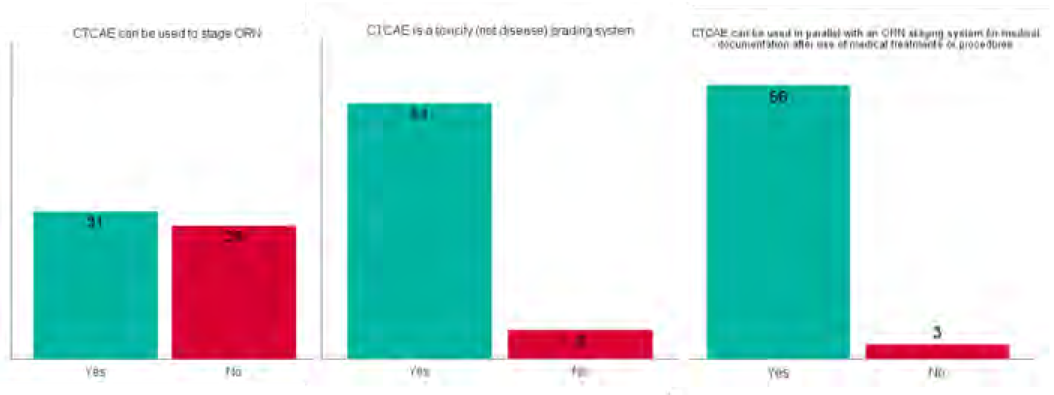

Consensus: CTCAE is a valuable toxicity grading system that should be used in parallel with, but not replace, an ORN staging system.

The following items focus on features relevant to staging the severity of ORN once it has been diagnosed.

medRxiv preprint doi: https://doi.org/10.1101/2024.04.07.24305400; this version posted April 9, 2024. The copyright holder for this preprint (which was not certified by peer review) is the author/funder, who has granted medRxiv a license to display the preprint in perpetuity.
It is made available under a CC-BY 4.0 International license .

Time Feature and Staging

Two questions related to time and its relevance as a **staging feature** for ORN were asked during Round 2. In the first question, **68% (40/59)** agreed that a staging system for ORN should be developed without a mandatory inclusion of a time feature. When rephrased to state that a time feature could be an optional modifier but not a necessary factor, **83% (47/57) agreed**.

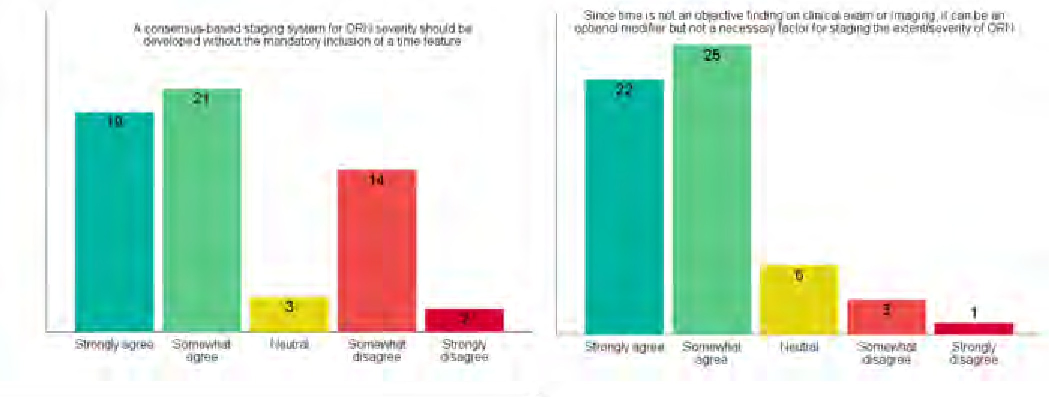

As far as reporting time for <u>assessing response to therapy</u> (considered to be distinct from reporting time as a staging feature for ORN), **85% (49/58) agreed.**

Consensus: A time feature is not necessary for staging ORN. However, reporting time is still beneficial for reporting the duration of observed ORN or response to therapy.

Symptoms and Staging

Symptoms such as pain are often reported in patients with ORN. For the purposes of staging the severity of ORN, **72%** (41/57) of the panel agreed that symptoms are not necessary features.

medRxiv preprint doi: https://doi.org/10.1101/2024.04.07.24305400; this version posted April 9, 2024. The copyright holder for this preprint (which was not certified by peer review) is the author/funder, who has granted medRxiv a license to display the preprint in perpetuity.
It is made available under a CC-BY 4.0 International license .

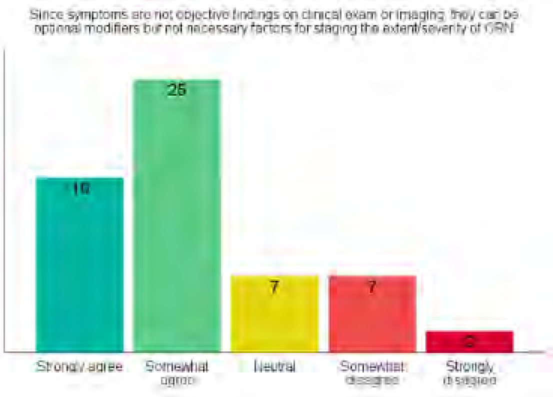

Consensus: Symptoms are not necessary for staging ORN. They can be reported as additional information but will not be considered as an upstaging feature.

Case Reviews

Case Review Summary

Several cases regarding a head & neck cancer patient treated with RT (with no evidence of cancer during surveillance) were reviewed during Round 2. Distribution of percent responses are shown per case.

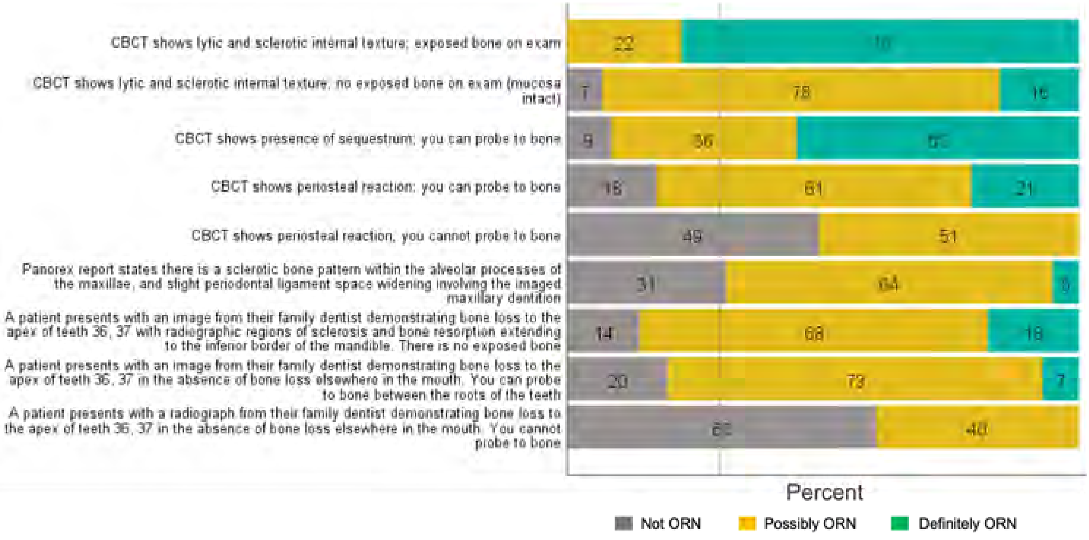

Reformatting cases based on clinical and imaging findings is

medRxiv preprint doi: https://doi.org/10.1101/2024.04.07.24305400; this version posted April 9, 2024. The copyright holder for this preprint (which was not certified by peer review) is the author/funder, who has granted medRxiv a license to display the preprint in perpetuity.
It is made available under a CC-BY 4.0 International license .

shown below. Minor variations to the same case are shaded in the same color. For example, exposed bone with lytic or sclerotic changes was considered ORN by 78% of the group whereas the same imaging findings with a clinical finding of intact mucosa (i.e., non-exposed bone) were rated as possible ORN (?ORN, 78%) or definitely ORN (16%).

Clinical Finding	Imaging Finding (location specified)	Result
Exposed bone	Lytic or sclerotic changes	ORN
Intact mucosa	Lytic or sclerotic changes	?ORN/ORN
Probe to bone	Sequestrum	?ORN/ORN
Probe to bone	Periosteal reaction	?ORN/ORN
Cannot probe to bone	Periosteal reaction	**Not ORN ?Precursor?**
No findings	Sclerosis, periodontal ligament widening (alveolar bone only)	**Not ORN ?Precursor?**
Intact mucosa	Sclerosis and bone resorption (to inferior mandible)	?ORN/ORN
Probe to bone	Bone loss at tooth apex (alveolar bone only)	?ORN/ORN
Cannot probe to bone	Bone loss at tooth apex (alveolar bone only)	**Not ORN ?Precursor?**

Early vs. Advanced Stage ORN

Another series of scenarios or specific features were reviewed and classified as 'not needed for ORN staging', 'early / limited ORN', or 'advanced ORN'. Group responses are shown below.

medRxiv preprint doi: https://doi.org/10.1101/2024.04.07.24305400; this version posted April 9, 2024. The copyright holder for this preprint (which was not certified by peer review) is the author/funder, who has granted medRxiv a license to display the preprint in perpetuity.
It is made available under a CC-BY 4.0 International license .

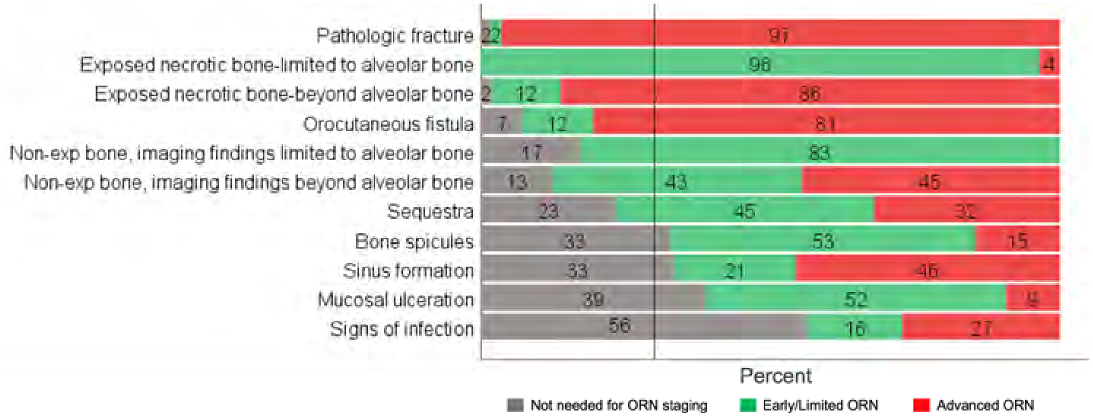

Features and feature combinations meeting consensus are:

	Clinical Findings	Imaging Findings	Extent of Bone Involvement
**Early**	Intact mucosa (non-exposed bone)	Any	Limited to alveolar bone
	Exposed necrotic bone	Any	Limited to alveolar bone
**Advanced**	Exposed necrotic bone	Any	Beyond alveolar bone
	Any	Pathologic Fracture	
	Any	Orocutaneous Fistula	

These items have been classified as **advanced ORN**. For further clarification, drag and drop <u>every item</u> to an intermediate or advanced stage box. If you consider all to be of same severity, drag them all to the advanced stage box.

medRxiv preprint doi: https://doi.org/10.1101/2024.04.07.24305400; this version posted April 9, 2024. The copyright holder for this preprint (which was not certified by peer review) is the author/funder, who has granted medRxiv a license to display the preprint in perpetuity.
It is made available under a CC-BY 4.0 International license .

	Intermediate Stage	Advanced Stage
Exposed necrotic bone with positive imaging findings **extending beyond the alveolar bone**		
Pathologic fracture		
Orocutaneous fistula		
Oro-antral or oro-nasal fistula		

These items have been classified as **possible or early ORN due to findings limited to alveolar bone**. For further clarification, drag and drop every item to a specific staging box. If you consider all to be of same severity, drag them all to the same stage box. Note: 'Advanced ORN' is not an option for these items.
Abbreviations: AB, alveolar bone; PTB, probe-to-bone test

A PTB test example is shown below. A positive PTB test is when a hard, bone-like surface is felt after gentle probing of a wound or ulcer.

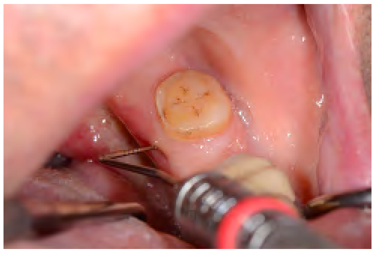

	**Not a precursor / Not related to ORN**	**Precursor / Stage 0**
Minor bone spicules		
Intact mucosa, any image finding in AB		

medRxiv preprint doi: https://doi.org/10.1101/2024.04.07.24305400; this version posted April 9, 2024. The copyright holder for this preprint (which was not certified by peer review) is the author/funder, who has granted medRxiv a license to display the preprint in perpetuity.
It is made available under a CC-BY 4.0 International license .

Early ORN

Exposed necrotic bone, any image finding in AB

PTB negative, periosteal reaction in AB

PTB negative, any image finding (except periosteal reaction) in AB

PTB positive, periosteal reaction in AB

PTB positive, any image finding (except periosteal reaction) in AB

Vascular damage after radiation therapy can be measured in patients with ORN using DCE-MRI (PMID: 32712257). How should we classify a case with vascular damage in bone seen on MRI without exposed bone and without other imaging findings (i.e., CT shows no bony abnormalities)?

Not a precursor / Not related to ORN	Precursor to ORN / Stage 0	Early ORN
○	○	○

How should we classify a case with vascular damage in bone seen on MRI without exposed bone and with other imaging findings limited to alveolar bone (i.e., x-ray shows sclerosis limited to alveolar bone)?

medRxiv preprint doi: https://doi.org/10.1101/2024.04.07.24305400; this version posted April 9, 2024. The copyright holder for this preprint (which was not certified by peer review) is the author/funder, who has granted medRxiv a license to display the preprint in perpetuity.
It is made available under a CC-BY 4.0 International license .

Not a precursor / Not related to ORN	Precursor to ORN / Stage 0	Early ORN
◯	◯	◯

Cases with Imaging

These items are case scenarios with clinical and radiographic information. Classify the severity of ORN and extent of bone involvement based on the information provided, and rate your level of confidence. *Note: Some images have the bony changes outlined in a red box while others do not.*

Case 1

Clinical exam:

Intact mucosa (no bone exposure)

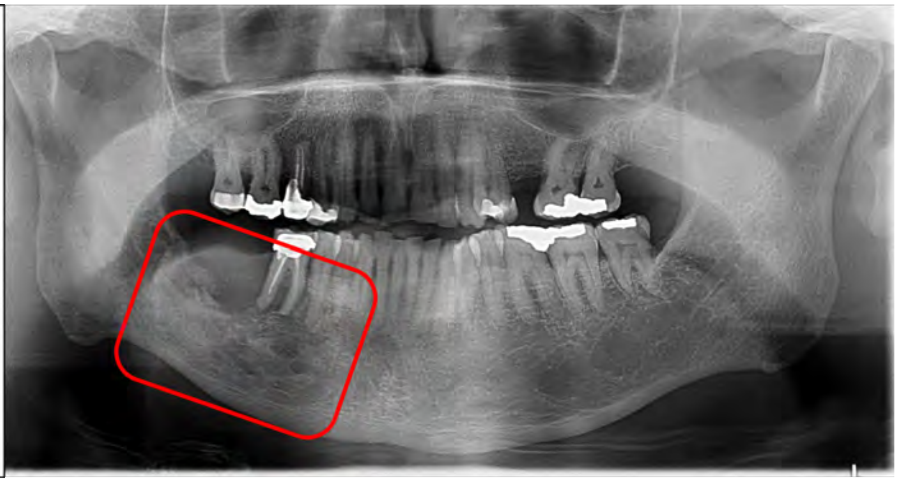

	Please select the best choice for this case	Extent of Bone Involvement	Level of Cor 1 = low ... 5		
			1	2	3
Case Classification	[dropdown]	[dropdown]	◯	◯	◯

medRxiv preprint doi: https://doi.org/10.1101/2024.04.07.24305400; this version posted April 9, 2024. The copyright holder for this preprint (which was not certified by peer review) is the author/funder, who has granted medRxiv a license to display the preprint in perpetuity.
It is made available under a CC-BY 4.0 International license .

Case 2

Clinical exam:

Exposed bone

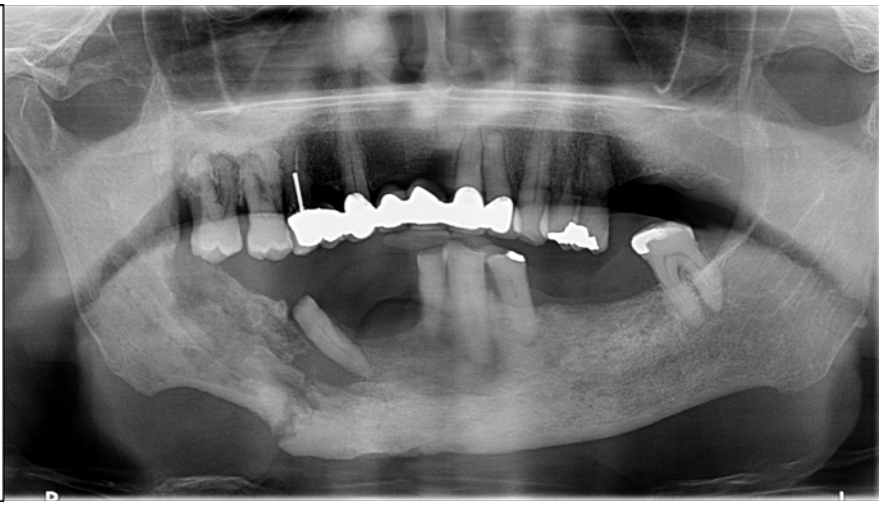

	Please select the best choice for this case	Extent of Bone Involvement	Level of Cont<br>1 = low ... 5		
			1	2	3
Case Classification			○	○	○

Case 3

Clinical exam:

Intact mucosa (no bone exposure)

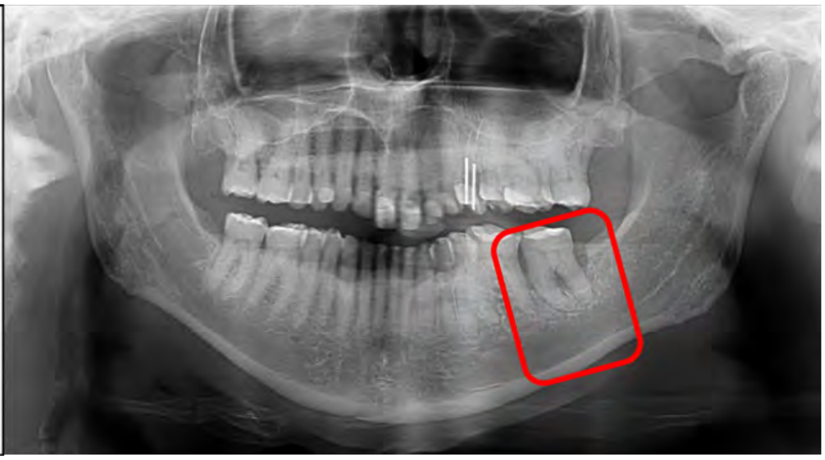

medRxiv preprint doi: https://doi.org/10.1101/2024.04.07.24305400; this version posted April 9, 2024. The copyright holder for this preprint (which was not certified by peer review) is the author/funder, who has granted medRxiv a license to display the preprint in perpetuity.
It is made available under a CC-BY 4.0 International license .

	Please select the best choice for this case	Extent of Bone Involvement	Level of Cor 1 = low ... 5		
			1	2	3
Case Classification			○	○	○

Case 4. PTB test is positive.

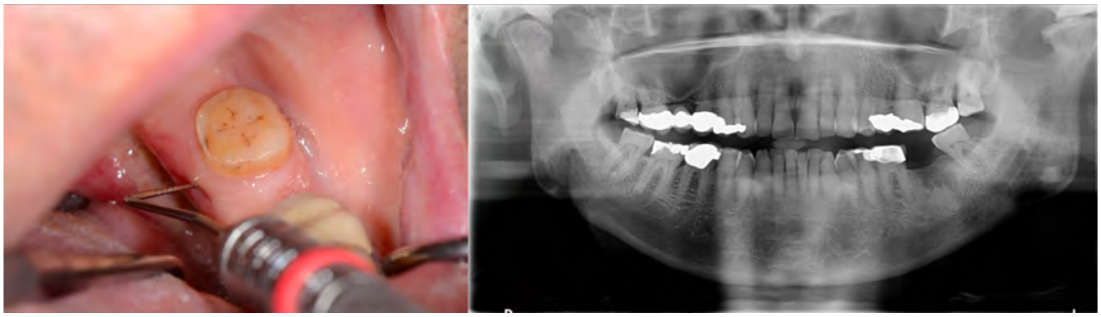

	Please select the best choice for this case	Extent of Bone Involvement	Level of Conf 1 = low ... 5 =		
			1	2	3
Case Classification			○	○	○

Case 5

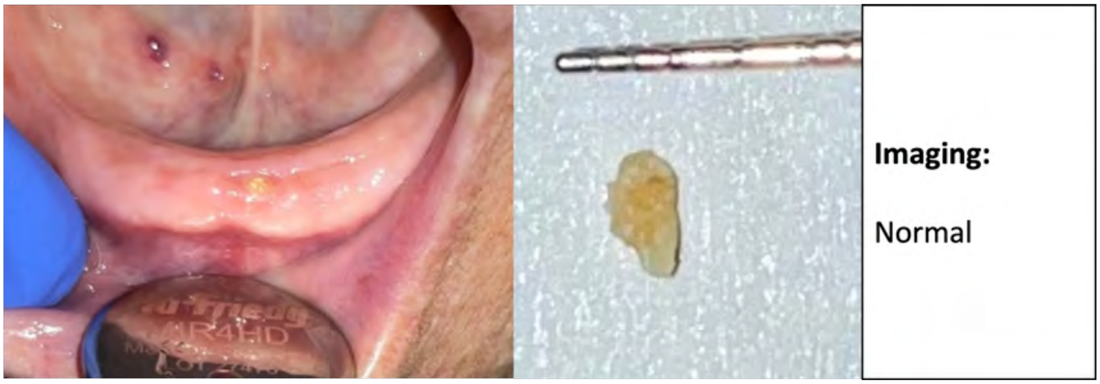

medRxiv preprint doi: https://doi.org/10.1101/2024.04.07.24305400; this version posted April 9, 2024. The copyright holder for this preprint (which was not certified by peer review) is the author/funder, who has granted medRxiv a license to display the preprint in perpetuity.
It is made available under a CC-BY 4.0 International license .

	Please select the best choice for this case	Extent of Bone Involvement	Level of Cor 1 = low ... 5		
			1	2	3
Case Classification			○	○	○

Case 6

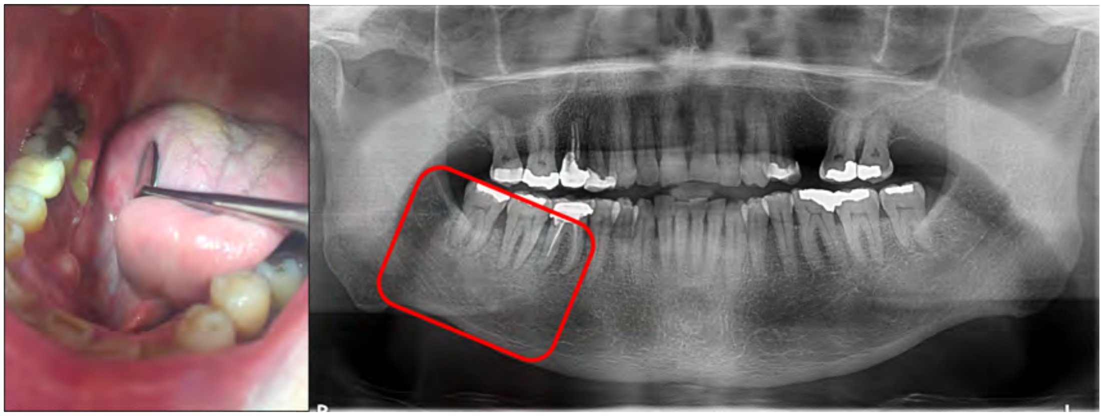

	Please select the best choice for this case	Extent of Bone Involvement	Level of Cor 1 = low ... 5		
			1	2	3
Case Classification			○	○	○

Case 7. A mucosal ulceration with no bone exposure is noted on clinical exam. MRI-DCE shows 'extensive loss of fat signal in the left mandible with marrow edema and associated gingival swelling and hyper-enhancement.' Black bone MRI shows intact cortical bone. CT shows 'soft tissue swelling and enhancement of the left mandibular gingiva and adjacent buccal space. Tooth #20 has been extracted. No suspicious bony abnormality is otherwise noted in the adjacent mandible

medRxiv preprint doi: https://doi.org/10.1101/2024.04.07.24305400; this version posted April 9, 2024. The copyright holder for this preprint (which was not certified by peer review) is the author/funder, who has granted medRxiv a license to display the preprint in perpetuity.
It is made available under a CC-BY 4.0 International license .

despite evidence of bone marrow edema on recent MRI.'

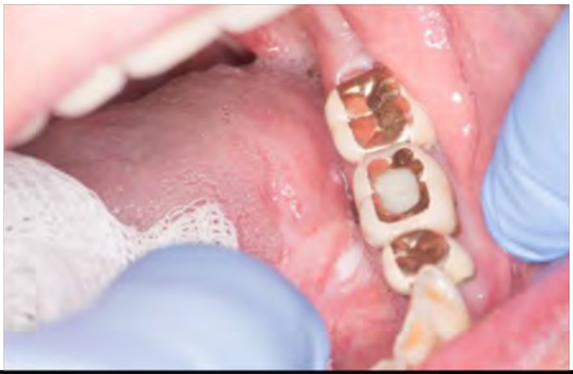

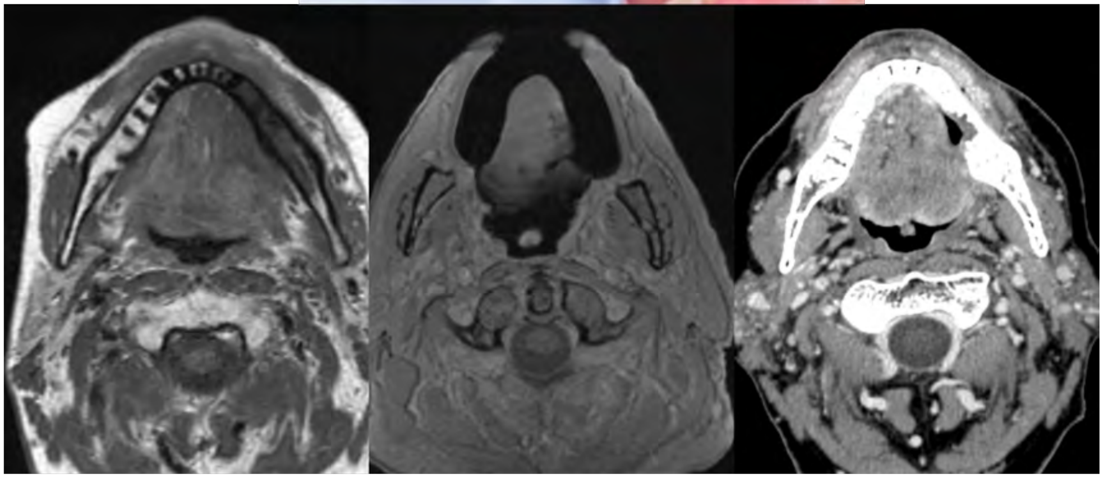

	Please select the best choice for this case	Extent of Bone Involvement	Level of Co<br>1 = low ... 5		
			1	2	3
Case Classification			○	○	○

Case 8

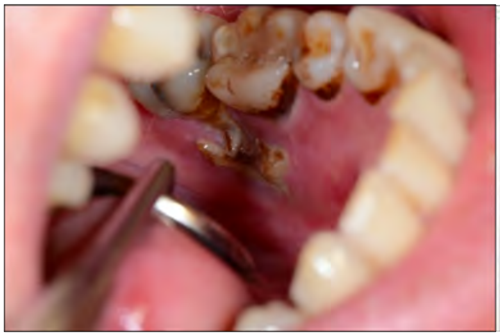

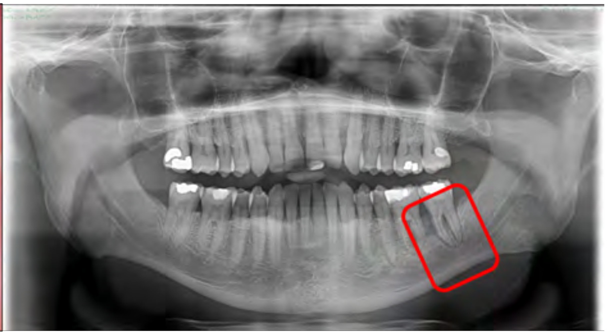

medRxiv preprint doi: https://doi.org/10.1101/2024.04.07.24305400; this version posted April 9, 2024. The copyright holder for this preprint (which was not certified by peer review) is the author/funder, who has granted medRxiv a license to display the preprint in perpetuity.
It is made available under a CC-BY 4.0 International license .

	Please select the best choice for this case	Extent of Bone Involvement	Level of Cor 1 = low ... 5 1	2	3
Case Classification			○	○	○

Case 9

**Clinical exam:**<br>Exposed bone

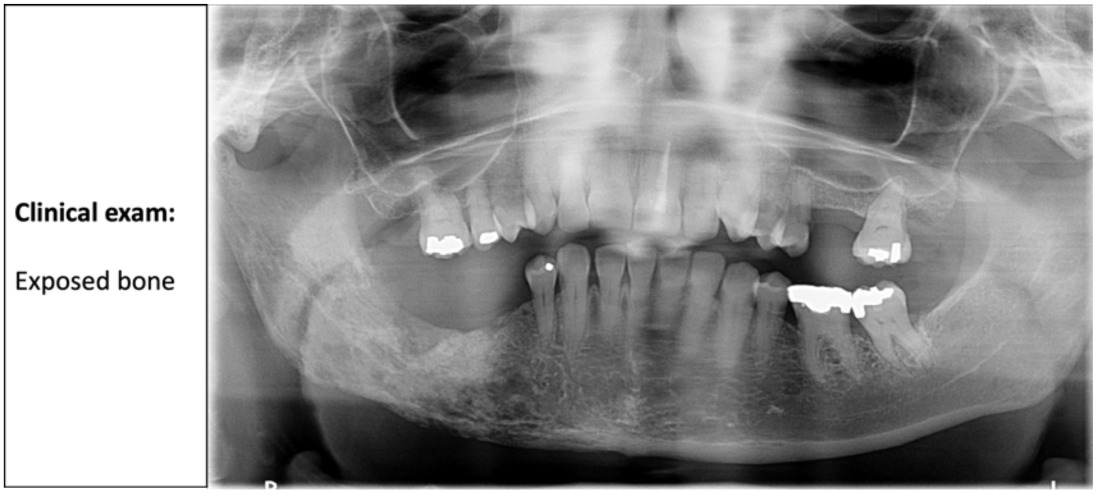

	Please select the best choice for this case	Extent of Bone Involvement	Level of Cor 1 = low ... 5 1	2	3
Case Classification			○	○	○

medRxiv preprint doi: https://doi.org/10.1101/2024.04.07.24305400; this version posted April 9, 2024. The copyright holder for this preprint (which was not certified by peer review) is the author/funder, who has granted medRxiv a license to display the preprint in perpetuity.
It is made available under a CC-BY 4.0 International license .

Case 10

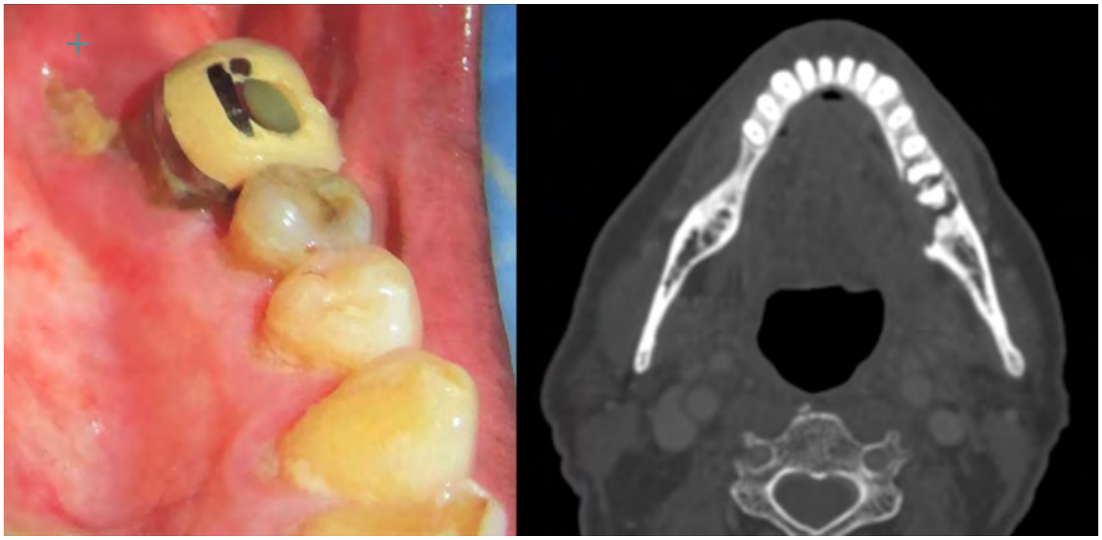

	Please select the best choice for this case	Extent of Bone Involvement	Level of Cor<br>1 = low ... 5		
			1	2	3
Case Classification			○	○	○

Case 11- Case Series

This is a patient diagnosed with ORN with serial bony changes seen on orthopantomagram (OPG). The photo below is the patient's teeth before RT.

Baseline OPG before RT:

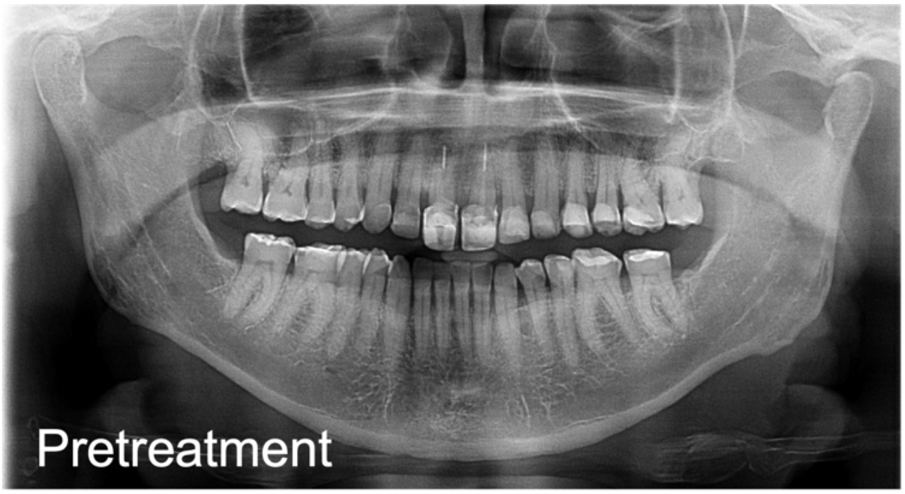

medRxiv preprint doi: https://doi.org/10.1101/2024.04.07.24305400; this version posted April 9, 2024. The copyright holder for this preprint (which was not certified by peer review) is the author/funder, who has granted medRxiv a license to display the preprint in perpetuity.
It is made available under a CC-BY 4.0 International license .

Click on the image links below to open surveillance imaging after RT. Upon review of the images, drag and drop the item names to the best option for stage. If more than one image could be staged the same, place them in the same staging box.

Links to surveillance imaging after RT:
OPG: 1 year (intact mucosa)
OPG: 3 years (exposed bone)
OPG: 4 years (exposed bone)

Items	Precursor / Stage 0	Early ORN
OPG: 1 year (intact mucosa)		
OPG: 3 years (exposed bone)	**Intermediate ORN**	**Advanced ORN**
OPG: 4 years (exposed bone)		

RADMAP Summary

RADMAP Summary

Regarding customization of a report with radiation dose data over an odontogram:

- **92%** (54/59) recommended inclusion of systemic therapy information
- **86%** (51/59) recommended inclusion of tobacco / smoking information
- **60%** (35/58) recommended inclusion of gender

medRxiv preprint doi: https://doi.org/10.1101/2024.04.07.24305400; this version posted April 9, 2024. The copyright holder for this preprint (which was not certified by peer review) is the author/funder, who has granted medRxiv a license to display the preprint in perpetuity.
It is made available under a CC-BY 4.0 International license .

The top-rated heat map preferences were: single hue color palette with varying saturation (n=33) followed by a diverging color palette (n=22). Heat map colors should be color blind friendly.

This officially closes the RADMAP section of the study with expert input being taken into consideration for future report building and testing. Round 4 will provide final group feedback related to ORN diagnosis and staging.

Any comments for Round 3?

Powered by Qualtrics

medRxiv preprint doi: https://doi.org/10.1101/2024.04.07.24305400; this version posted April 9, 2024. The copyright holder for this preprint (which was not certified by peer review) is the author/funder, who has granted medRxiv a license to display the preprint in perpetuity.
It is made available under a CC-BY 4.0 International license .

THE UNIVERSITY OF TEXAS
MD Anderson ~~Cancer~~ Center
Making Cancer History®

R4_Intro

ORAL Consortium Round 4 (Final) Survey

Hello everyone!
This will be the fourth and final round of the ORAL Consortium Delphi Study and will focus on finalizing the Consortium's definition of ORN and clinical/radiographic classification of ORN based on group feedback from the prior rounds. Beyond this round, any topics that do not meet a new pre-defined consensus threshold of 60% will be qualitatively discussed in a related manuscript about the ORAL Consortium consensus guidelines.

Any topics that have met consensus during Round 3 will be reported in blue font.

To confirm, please select your desired level of involvement in associated manuscript(s).

- ○ I would like to serve as a **group co-author.** *Selecting this option will include you in regular email updates on the manuscript and a request for completion of an ICMJE disclosure form. Your name will be associated with the ORAL Consortium group authorship.*
- ○ I would like to only be **acknowledged** in the manuscript. Please send manuscript updates to me.
- ○ I would like to only be **acknowledged** in the manuscript. Please **do NOT** send manuscript updates to me.

medRxiv preprint doi: https://doi.org/10.1101/2024.04.07.24305400; this version posted April 9, 2024. The copyright holder for this preprint (which was not certified by peer review) is the author/funder, who has granted medRxiv a license to display the preprint in perpetuity.
It is made available under a CC-BY 4.0 International license .

○ Please do not include my name in the manuscript and do not send me updates.

R3_Feedback & Int-Adv ORN

The Definition of ORN

A total of 56 panelists partially or fully completed Round 3.
In Round 3, the working ORN definition was further revised. Panelists were asked to: A) select which **additional statements** should be considered for inclusion in the definition from Round 2, and B) choose the **best** and **second-best** option out of 4 working definitions of ORN.

A) No additional comments met consensus and therefore will not be added to the final ORN definition. The highest scoring comment was: “it should include the presence of exposed bone” which was selected by 25 experts or 45% of the group. Distribution of selected statements is shown below.

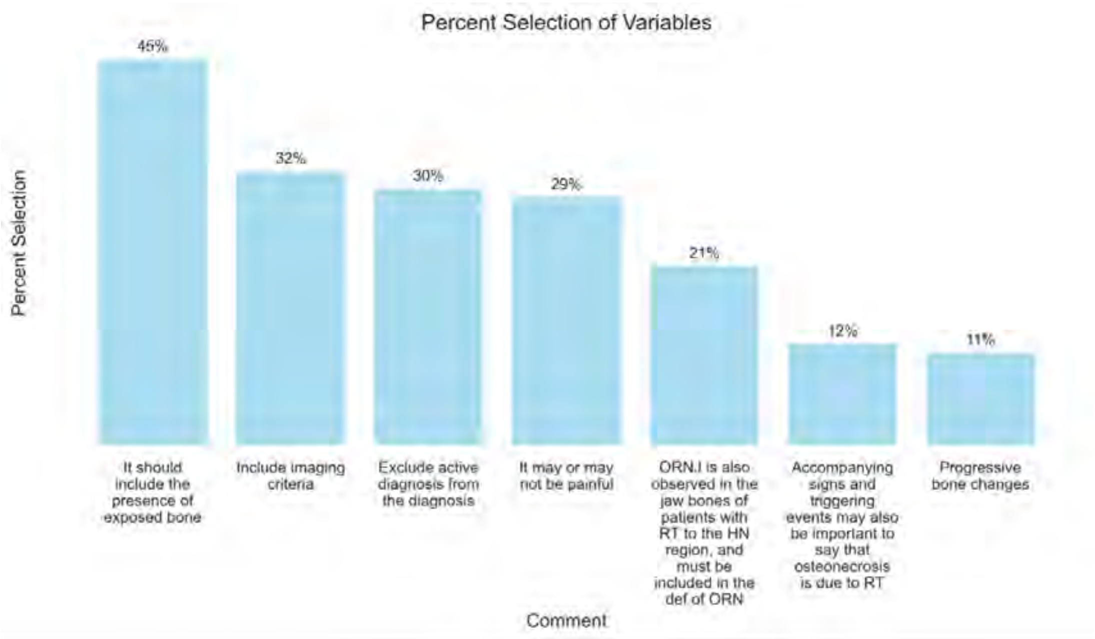

medRxiv preprint doi: https://doi.org/10.1101/2024.04.07.24305400; this version posted April 9, 2024. The copyright holder for this preprint (which was not certified by peer review) is the author/funder, who has granted medRxiv a license to display the preprint in perpetuity.
It is made available under a CC-BY 4.0 International license .

B) **Best and Second Best Option.** Version 1.2 was most selected for 'Best option' by 62% of responders followed by version 1.3 as 'Second-best option' selected by 47% of the group.

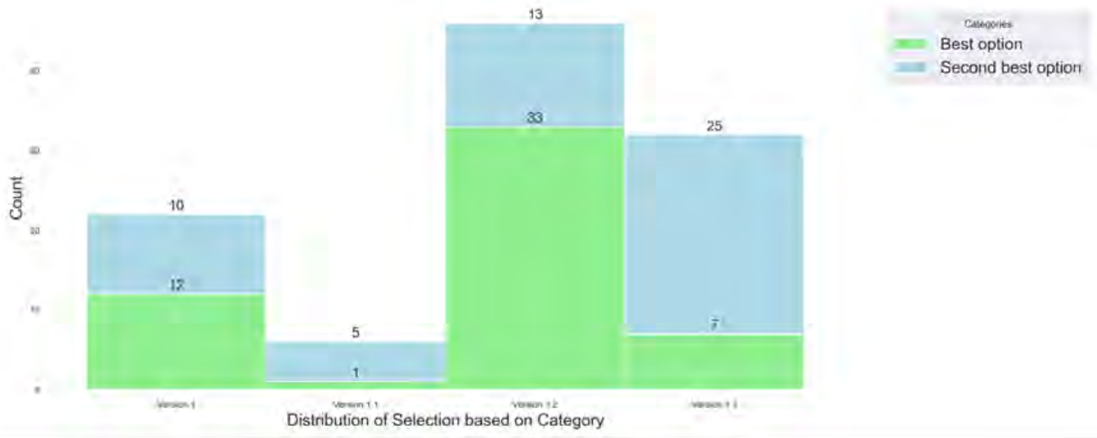

For this final round, please select your **most preferred ORN definition** derived from versions 1.2 and 1.3. Differences between definitions has been underlined.

- ◯ A condition in which there is a loss of blood flow to bone tissue, which causes the bone to die. Findings of bone death may be clinical (i.e., exposed bone) and/or radiographic (i.e., sclerosis, pathologic fracture). It is caused by exposure to ionizing radiation and may occur at some point in time after radiation and in the absence of active disease (i.e., cancer) in the site of bone death.
- ◯ A condition in which there is a loss of blood flow to bone tissue, which causes the bone to die. Findings of bone death may be clinical (i.e., exposed bone) and/or radiographic (i.e., sclerosis, pathologic fracture). It is caused by exposure to ionizing radiation and occurs in the site of bone death.

New Staging System Publication Update

A new risk-based model for ORN classification has been published by Watson et al. which incorporates both clinical and imaging features.

medRxiv preprint doi: https://doi.org/10.1101/2024.04.07.24305400; this version posted April 9, 2024. The copyright holder for this preprint (which was not certified by peer review) is the author/funder, who has granted medRxiv a license to display the preprint in perpetuity.
It is made available under a CC-BY 4.0 International license .

Please right click on the link and open a new tab: Watson ORN Staging System

After review of this publication, do you find this new system helpful?

- ◯ Strongly agree
- ◯ Somewhat agree
- ◯ Neither agree nor disagree
- ◯ Somewhat disagree
- ◯ Strongly disagree

Intermediate vs. Advanced Stage Features

In Round 3, panelists were asked to classify 4 features as intermediate or advanced stage. The following met the consensus threshold to be indicative of advanced ORN: **pathologic fracture** (49/51, 96%), **orocutaneous fistula** (47/51, 92%) and **oro-antral or oro-nasal fistula** (43/51, 86%).

Classification of '*exposed necrotic bone with positive imaging findings extending beyond the alveolar bone*' did not meet consensus (43% intermediate; 57% advanced). A significant association between specialty group and recommended ORN stage was observed for this feature with most dental specialists considering it as **intermediate stage** (69%) while other specialty groups (72% Rad Onc, 57% Surg Onc) considered it as **advanced stage** (P = 0.04).

medRxiv preprint doi: https://doi.org/10.1101/2024.04.07.24305400; this version posted April 9, 2024. The copyright holder for this preprint (which was not certified by peer review) is the author/funder, who has granted medRxiv a license to display the preprint in perpetuity.
It is made available under a CC-BY 4.0 International license .

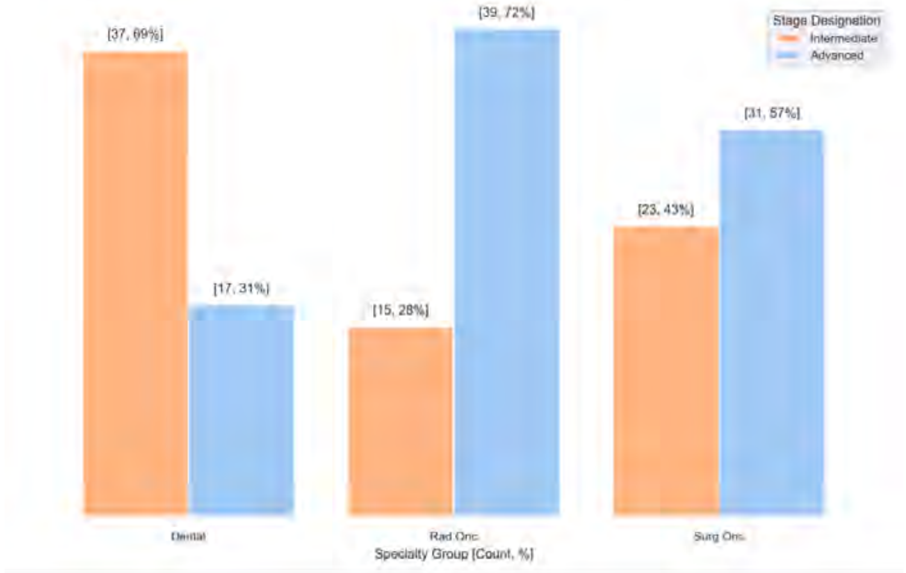

Case 9 represented a similar scenario of clinically exposed bone with radiographic features beyond the alveolar bone in the right mandible.

Clinical exam:

Exposed bone

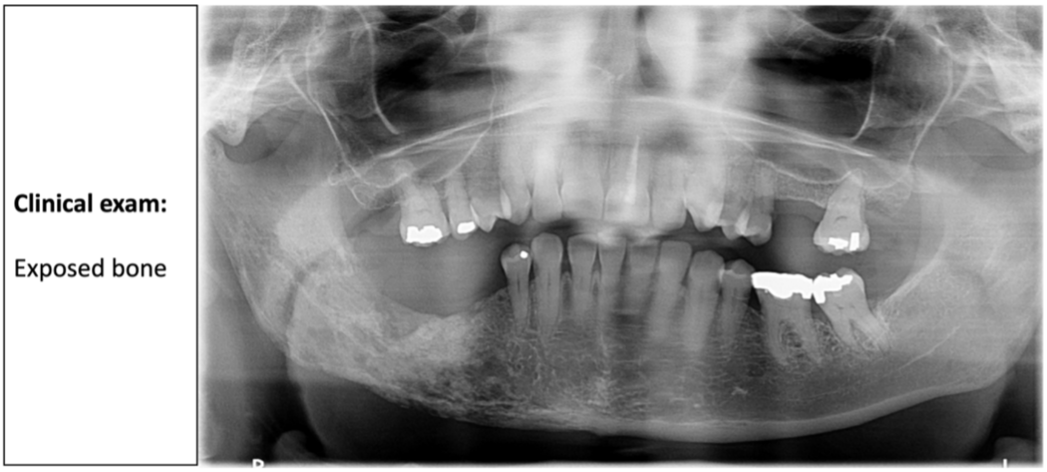

Group feedback for this case is as follows. Note: This case was classified with **moderate-to-high level of confidence** by 62% panelists in dentistry/oral medicine, 32% RadOnc, and 28% Surgery (56% overall).

Stage Designation	Count	%
Intermediate ORN	20	44%
Advanced ORN	18	40%
Early ORN	6	13%
Not precursor / Not ORN	1	2%

Bone Involvement	Count	%
Beyond alveolar bone	34	76%
Limited to alveolar bone	10	22%
No abnormality	1	2%

medRxiv preprint doi: https://doi.org/10.1101/2024.04.07.24305400; this version posted April 9, 2024. The copyright holder for this preprint (which was not certified by peer review) is the author/funder, who has granted medRxiv a license to display the preprint in perpetuity.
It is made available under a CC-BY 4.0 International license .

Considering the group feedback and consensus-based features of advanced ORN (pathologic fracture, orocutaneous fistula, oro-antral/oro-nasal fistula), please reclassify the feature:

'Exposed necrotic bone with positive imaging findings extending **beyond** the alveolar bone'

- ○ Intermediate ORN
- ○ Advanced ORN

Precursor vs Early ORN

To differentiate between unrelated, precursor, or early staging features of ORN, panelists were asked to classify 7 items as one of the following:
1. Not a precursor/not related to ORN
2. Precursor/stage 0
3. Early ORN

The figure below shows the distribution of stage designation per feature combination. Only 'exposed necrotic bone, any image finding in AB' met consensus to be classified as Early stage ORN (n=49, 100%).

medRxiv preprint doi: https://doi.org/10.1101/2024.04.07.24305400; this version posted April 9, 2024. The copyright holder for this preprint (which was not certified by peer review) is the author/funder, who has granted medRxiv a license to display the preprint in perpetuity.
It is made available under a CC-BY 4.0 International license .

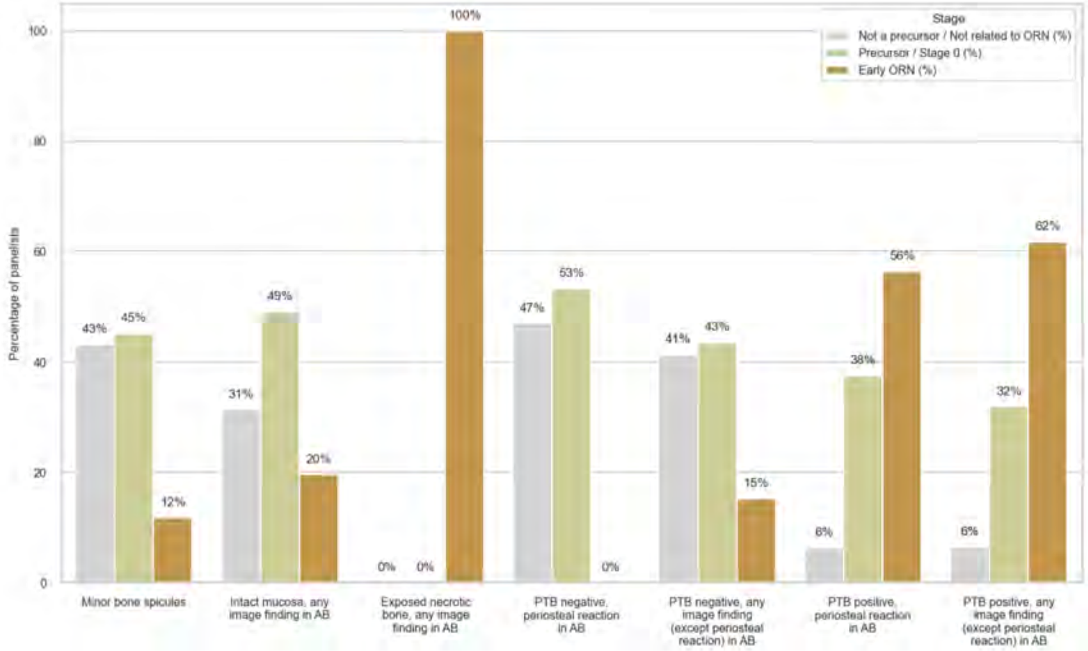

Abbreviations: AB, alveolar bone; PTB, probe to bone

The next series of questions will show group feedback for each feature combination, stratified by specialty. You will then be asked to classify the feature into one of the top 2 categories.

Group feedback for: **Minor bone spicules**

	Not a precursor/ Not Related to ORN	Precursor/ Stage 0	Early ORN
Entire Panel	43%	45%	12%

Classification per specialty:

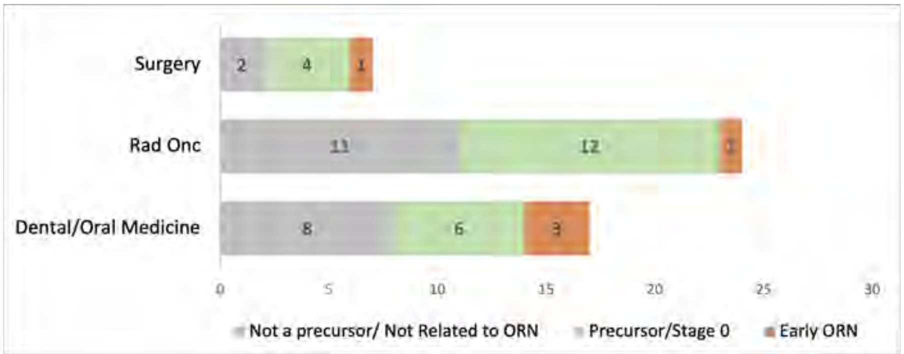

medRxiv preprint doi: https://doi.org/10.1101/2024.04.07.24305400; this version posted April 9, 2024. The copyright holder for this preprint (which was not certified by peer review) is the author/funder, who has granted medRxiv a license to display the preprint in perpetuity.
It is made available under a CC-BY 4.0 International license .

How should this feature be classified?

- ◯ Not a precursor / Not related to ORN
- ◯ Precursor / Stage 0

Group feedback for: **Intact mucosa, any imaging findings in alveolar bone**

	Not a precursor/ Not Related to ORN	Precursor/ Stage 0	Early ORN
Entire Panel	31%	49%	20%

Classification per specialty:

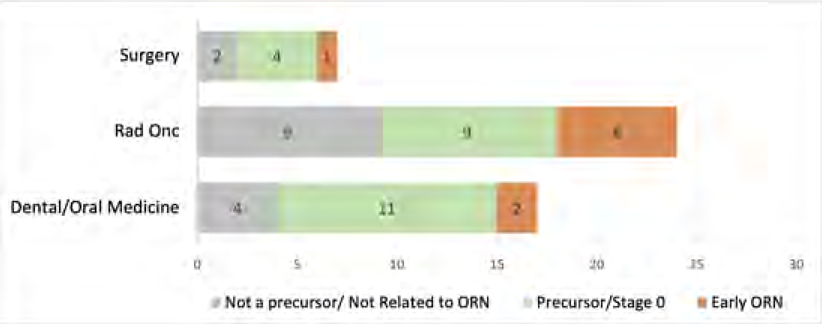

How should this feature be classified?

- ◯ Not a precursor / Not related to ORN
- ◯ Precursor / Stage 0

Group feedback for: **Probe-to-bone negative, periosteal reaction within alveolar bone seen on imaging**

	Not a precursor/ Not Related to ORN	Precursor/ Stage 0	Early ORN
Entire Panel	47%	53%	0%

medRxiv preprint doi: https://doi.org/10.1101/2024.04.07.24305400; this version posted April 9, 2024. The copyright holder for this preprint (which was not certified by peer review) is the author/funder, who has granted medRxiv a license to display the preprint in perpetuity.
It is made available under a CC-BY 4.0 International license .

Classification per specialty:

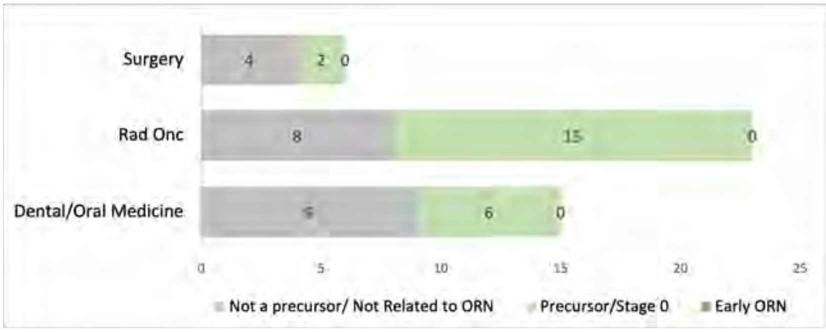

How should this feature be classified?

○ Not a precursor / Not related to ORN
○ Precursor / Stage 0

Group feedback for: **Probe-to-bone negative, any imaging finding (EXCEPT periosteal reaction) within alveolar bone**

	Not a precursor/ Not Related to ORN	Precursor/ Stage 0	Early ORN
Entire Panel	41%	43%	15%

Classification per specialty:

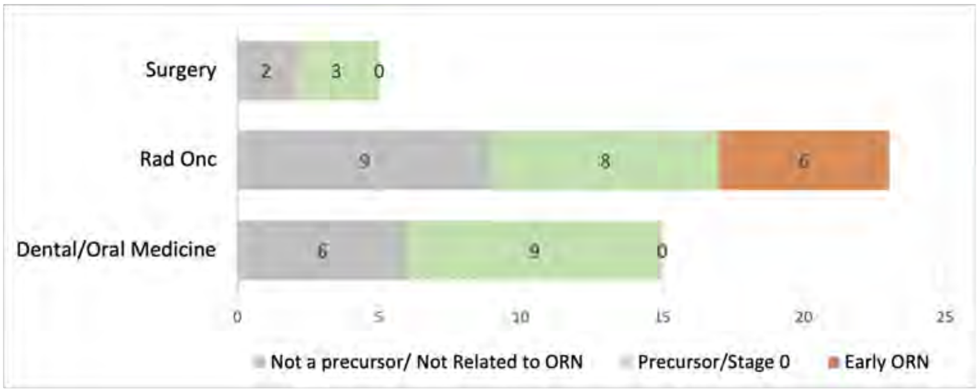

How should this feature be classified?

○ Not a precursor / Not related to ORN

medRxiv preprint doi: https://doi.org/10.1101/2024.04.07.24305400; this version posted April 9, 2024. The copyright holder for this preprint (which was not certified by peer review) is the author/funder, who has granted medRxiv a license to display the preprint in perpetuity.
It is made available under a CC-BY 4.0 International license .

◯ Precursor / Stage 0

Group feedback for: **Probe-to-bone POSITIVE, periosteal reaction within alveolar bone seen on imaging**

	Not a precursor/ Not Related to ORN	Precursor/ Stage 0	Early ORN
Entire Panel	6%	38%	56%

Classification per specialty:

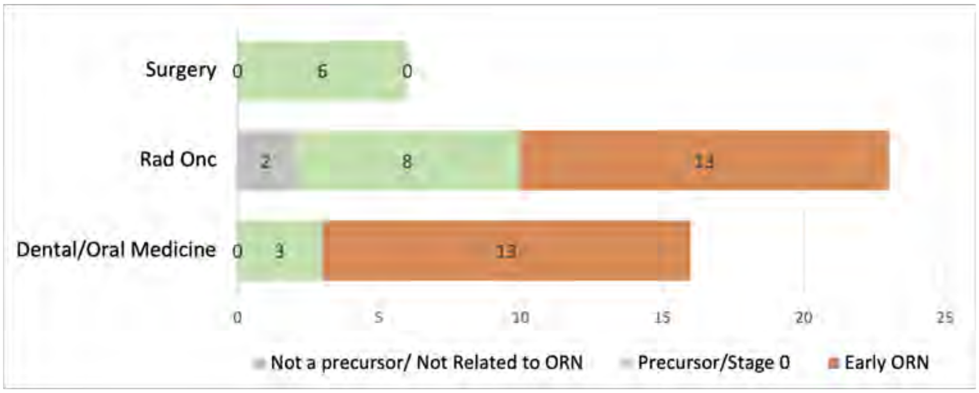

Specialty specific classifications meeting consensus threshold:
- Surgery: 100% favoring precursor / Stage 0
- Dental/Oral Medicine: 81% favoring early ORN

How should this feature be classified?

◯ Precursor / Stage 0
◯ Early ORN

Group feedback for: **Probe-to-bone POSITIVE, any imaging finding (EXCEPT periosteal reaction) within alveolar bone**

medRxiv preprint doi: https://doi.org/10.1101/2024.04.07.24305400; this version posted April 9, 2024. The copyright holder for this preprint (which was not certified by peer review) is the author/funder, who has granted medRxiv a license to display the preprint in perpetuity.
It is made available under a CC-BY 4.0 International license .

	Not a precursor/ Not Related to ORN	Precursor/ Stage 0	Early ORN
Entire Panel	6%	32%	62%

Classification per specialty:

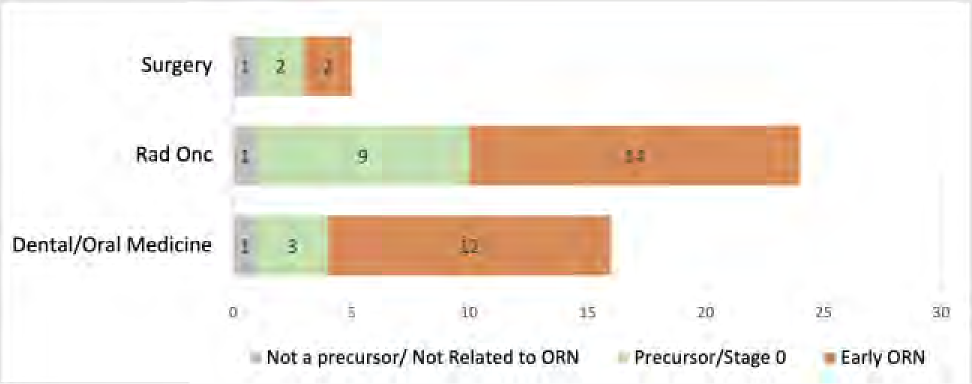

Specialty specific classifications meeting consensus threshold:

- Dental/Oral Medicine: 75% favoring early ORN

How should this feature be classified?

- ○ Precursor / Stage 0
- ○ Early ORN

Vascular Damage on MRI

Panelists were asked to classify two different scenarios whereby vascular damage could be seen on surveillance MRI after RT.

Scenario 1: How should we classify a case with **vascular damage in bone** seen on MRI **without** exposed bone and **without** other imaging findings (i.e., CT shows no bony abnormalities)?

Group feedback: 21 (41%) classified it as *Not a precursor/Not related to ORN*, and 29 (57%) classified it as *Precursor to ORN/Stage 0.*

medRxiv preprint doi: https://doi.org/10.1101/2024.04.07.24305400; this version posted April 9, 2024. The copyright holder for this preprint (which was not certified by peer review) is the author/funder, who has granted medRxiv a license to display the preprint in perpetuity.
It is made available under a CC-BY 4.0 International license .

Please reclassify this scenario

- ◯ Not a precursor / Not related to ORN
- ◯ Precursor / Stage 0

Vascular Damage on MRI

Scenario 2: How should we classify a case with **vascular damage in bone** seen on MRI **without** exposed bone and **with** other imaging findings **limited to alveolar bone** (i.e., x-ray shows sclerosis limited to alveolar bone)?

Group feedback: 26 (51%) classified it as *Precursor to ORN/Stage 0,* and 19 (37%) classified it as *Early ORN*.

Please reclassify this scenario

- ◯ Precursor / Stage 0
- ◯ Early ORN

10 Case Review

Review of 10 Cases with Clinical & Imaging Features

Round 3 ended with the panel reviewing 10 cases which included a clinical description and an image. Experts were asked to 1) stage the case, 2) report extent of bone involvement on imaging, and 3) rate their level of confidence (LOC).

The figure below summarizes the group **stage classification**

medRxiv preprint doi: https://doi.org/10.1101/2024.04.07.24305400; this version posted April 9, 2024. The copyright holder for this preprint
(which was not certified by peer review) is the author/funder, who has granted medRxiv a license to display the preprint in perpetuity.
It is made available under a CC-BY 4.0 International license .

(Fig A) and reporting of **extent of bone involvement** (Fig B) per case. The stacked bars and numbers represent percentages of the panel. Results for Case 2 (clinically exposed bone and pathological fracture on imaging) shows consistency with the panel classifying pathologic fracture as advanced ORN with bony involvement beyond the alveolar bone (AB). However, we can appreciate significant variations in staging assignment for the other cases. This is also true within specialties (figures not shown).

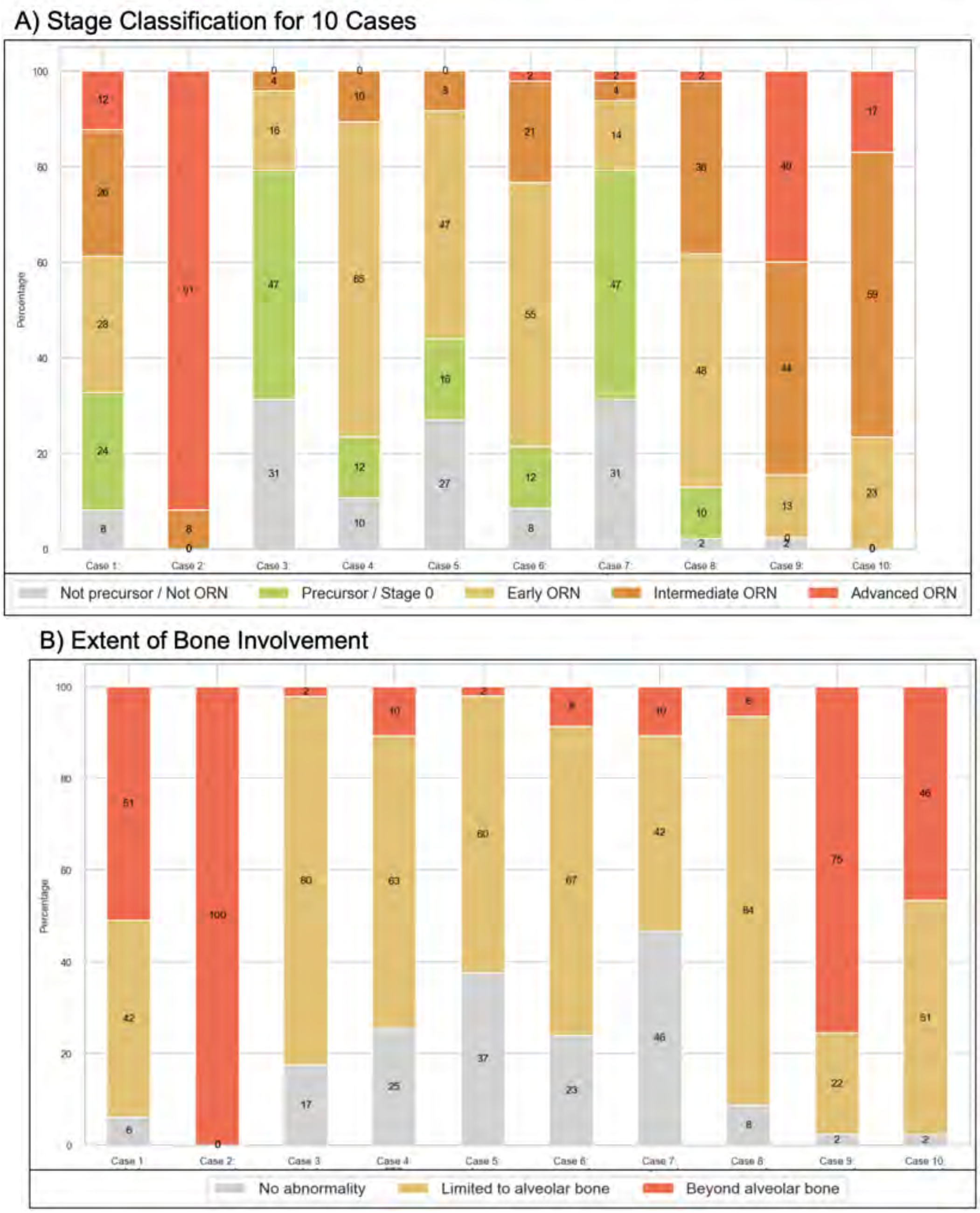

medRxiv preprint doi: https://doi.org/10.1101/2024.04.07.24305400; this version posted April 9, 2024. The copyright holder for this preprint (which was not certified by peer review) is the author/funder, who has granted medRxiv a license to display the preprint in perpetuity.
It is made available under a CC-BY 4.0 International license .

The LOC also varied among cases and **specialties** with the exception of Case 2. Note, Dental specialists all reported a LOC of 5 for Case 2.

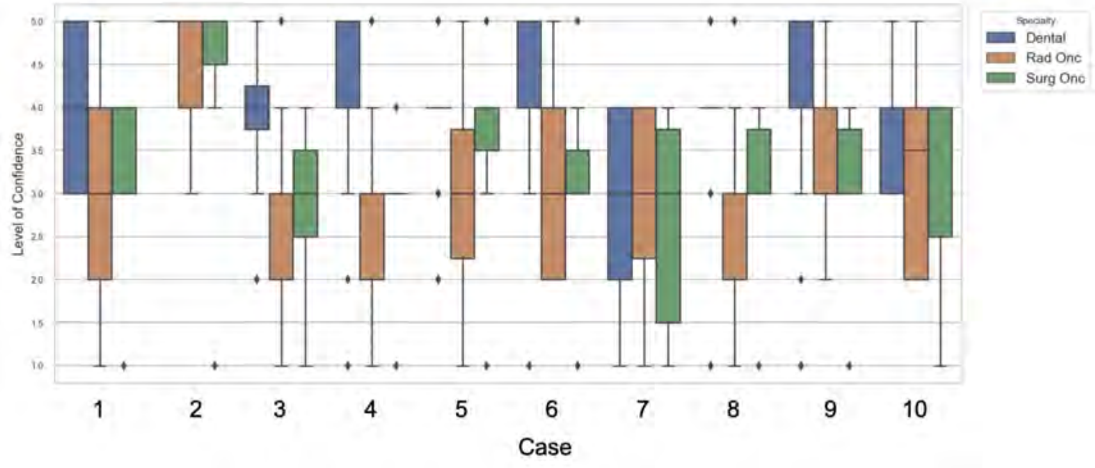

We will now re-review each case (except Case 2) and classify them based on the top 2-3 choices (i.e., minimum to reach 70%) selected during Round 3. Of note, some cases may not ask a question on extent of bone involvement as the panel already met consensus for that question. For cases with images, we will also ask you to **document what radiographic features you observe** (i.e., periodontal ligament space widening, sclerosis, lytic changes, periosteal reaction, etc). Please answer to your best ability as these details will be important to incorporate in an ORN ontology in the future.

Please right click on the links below to open educational resources on dental/mandibular changes on panoramic imaging after HN RT.

Mandibular changes on panoramic imaging after HN RT
Imaging of Radiation and Medication-related Osteonecrosis

If the pdfs above to not work, please use the following links:

medRxiv preprint doi: https://doi.org/10.1101/2024.04.07.24305400; this version posted April 9, 2024. The copyright holder for this preprint (which was not certified by peer review) is the author/funder, who has granted medRxiv a license to display the preprint in perpetuity.
It is made available under a CC-BY 4.0 International license .

Mandibular changes on panoramic imaging after head and neck radiotherapy
Imaging of Radiation- and Medication-Related Osteonecrosis

Case 1

Early ORN	Intermediate ORN
28%	26%

Clinical exam:

Intact mucosa (no bone exposure)

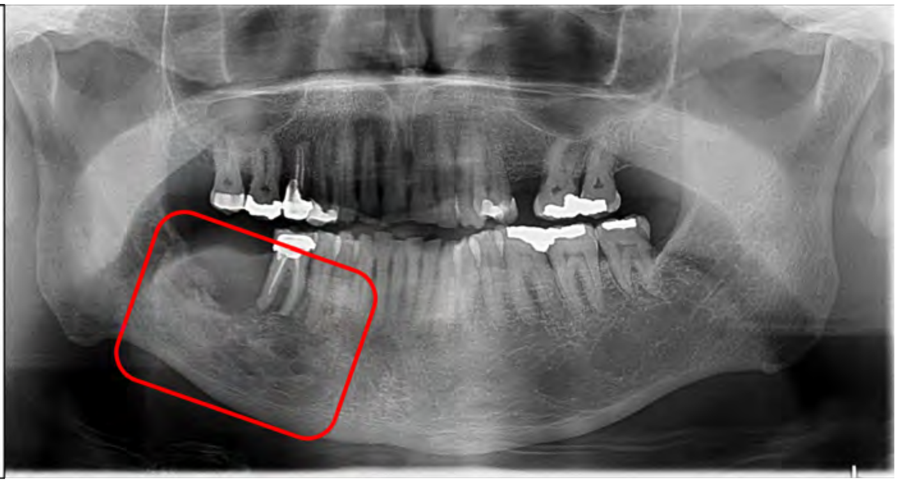

	Stage Assignment	Extent of Bone Involvement	Level of Confidence 1 = low ... 5 = high<br>1 2 3 4 5
Case Classification			○ ○ ○ ○ ○

What radiographic features do you observe in Case 1? Type **NA** if no abnormalities are seen.

medRxiv preprint doi: https://doi.org/10.1101/2024.04.07.24305400; this version posted April 9, 2024. The copyright holder for this preprint (which was not certified by peer review) is the author/funder, who has granted medRxiv a license to display the preprint in perpetuity.
It is made available under a CC-BY 4.0 International license .

Case 3 and 11 (1-year follow up)

Case	Not Precursor/ Not ORN	Precursor/ Stage 0	Early ORN
3	31%	47%	16%
11	-	61%	35%

Clinical exam:

Intact mucosa (no bone exposure)

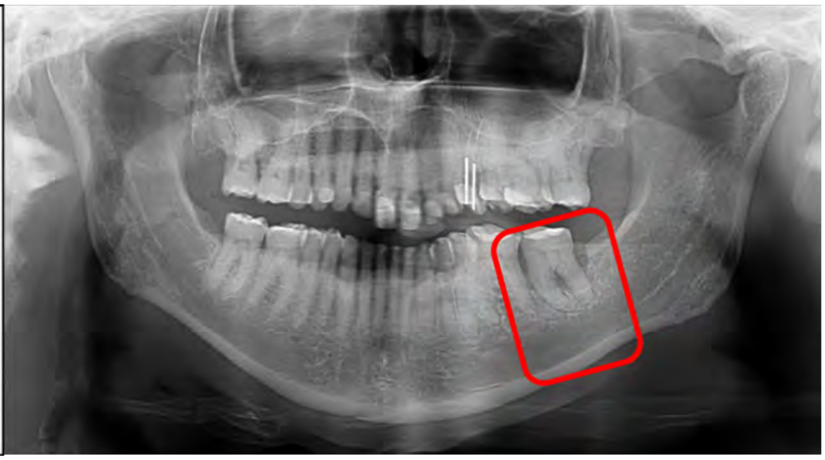

	Stage Assignment	Level of Confidence 1 = low ... 5 = high				
		1	2	3	4	5
Case Classification		○	○	○	○	○

What radiographic features do you observe in Case 3? Type **NA** if no abnormalities are seen.

Case 4. PTB test is positive.

Precursor/ Stage 0	Early ORN
12%	65%

medRxiv preprint doi: https://doi.org/10.1101/2024.04.07.24305400; this version posted April 9, 2024. The copyright holder for this preprint (which was not certified by peer review) is the author/funder, who has granted medRxiv a license to display the preprint in perpetuity.
It is made available under a CC-BY 4.0 International license .

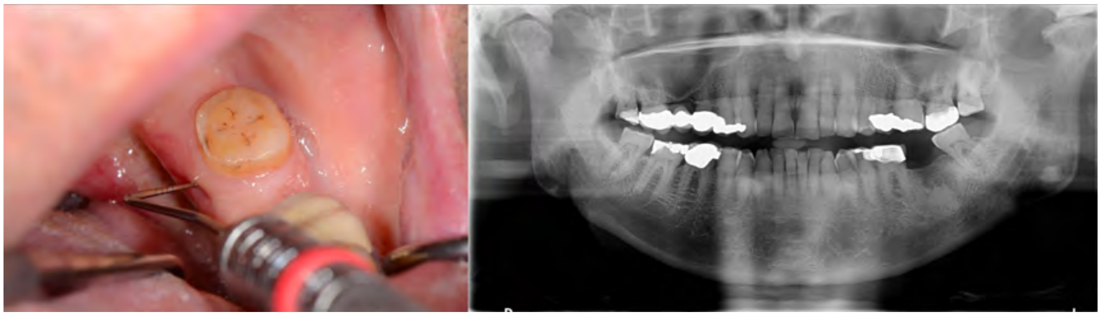

	Stage Assignment	Extent of Bone Involvement	Level of Confidence 1 = low ... 5 = high 1	2	3	4
Case Classification			○	○	○	○

What radiographic features do you observe in Case 4? Type **NA** if no abnormalities are seen.

Case 5

Question	Not precursor/ Not ORN	Precursor / Stage 0	Early ORN
Case 5	27%	16%	47%
Prior Q	43%	45%	12%

medRxiv preprint doi: https://doi.org/10.1101/2024.04.07.24305400; this version posted April 9, 2024. The copyright holder for this preprint (which was not certified by peer review) is the author/funder, who has granted medRxiv a license to display the preprint in perpetuity.
It is made available under a CC-BY 4.0 International license .

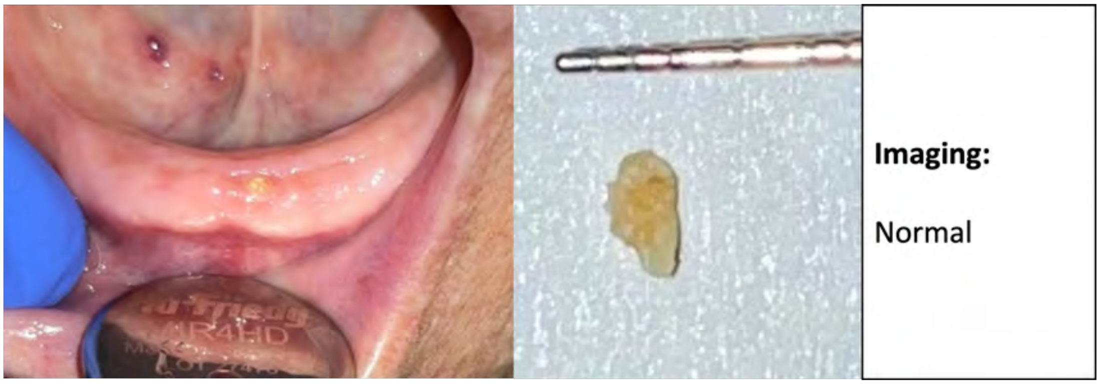

	Stage Assignment	Level of Confidence 1 = low ... 5 = high`				
		1	2	3	4	5
Case Classification		○	○	○	○	○

Case 6

Early ORN	Intermediate ORN
55%	21%

Note, 'exposed necrotic bone, any image finding in alveolar bone' was classified as Early stage ORN by 100% of the panel over precursor/Stage 0.

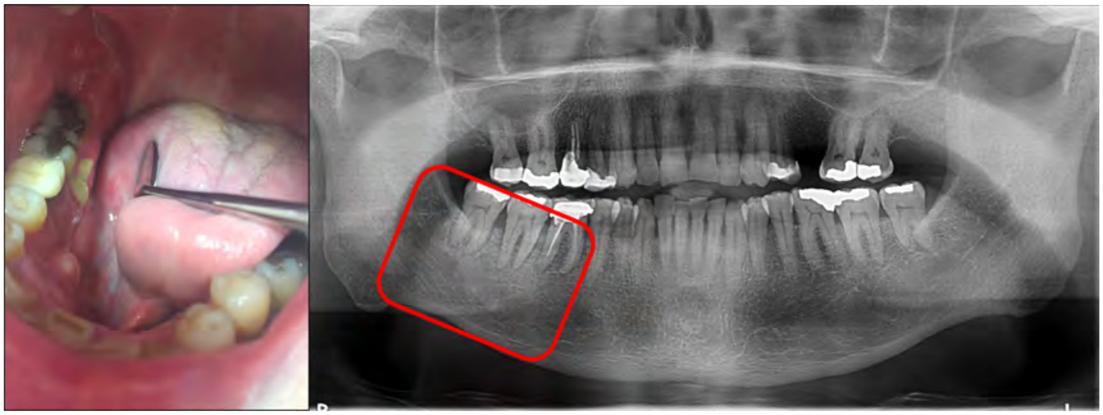

medRxiv preprint doi: https://doi.org/10.1101/2024.04.07.24305400; this version posted April 9, 2024. The copyright holder for this preprint (which was not certified by peer review) is the author/funder, who has granted medRxiv a license to display the preprint in perpetuity.
It is made available under a CC-BY 4.0 International license .

	Stage Assignment	Extent of Bone Involvement	Level of Confidence 1 = low ... 5 = high			
			1	2	3	4
Case Classification			○	○	○	○

What radiographic features do you observe in Case 6? Type **NA** if no abnormalities are seen.

Case 7. A mucosal ulceration with no bone exposure is noted on clinical exam. MRI-DCE shows 'extensive loss of fat signal in the left mandible with marrow edema and associated gingival swelling and hyper-enhancement.' Black bone MRI shows intact cortical bone. CT shows 'soft tissue swelling and enhancement of the left mandibular gingiva and adjacent buccal space. Tooth #20 has been extracted. No suspicious bony abnormality is otherwise noted in the adjacent mandible despite evidence of bone marrow edema on recent MRI.'

**Not precursor / Not ORN**	**Precursor / Stage 0**
31%	47%

medRxiv preprint doi: https://doi.org/10.1101/2024.04.07.24305400; this version posted April 9, 2024. The copyright holder for this preprint (which was not certified by peer review) is the author/funder, who has granted medRxiv a license to display the preprint in perpetuity.
It is made available under a CC-BY 4.0 International license .

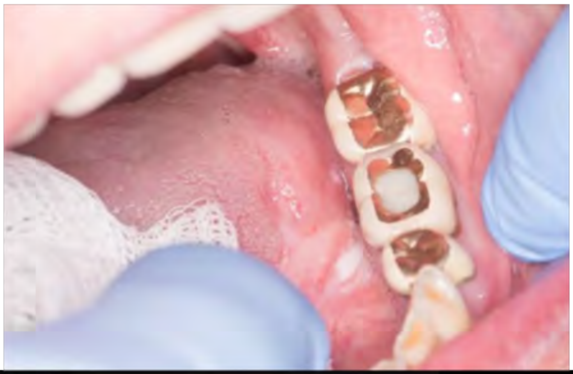

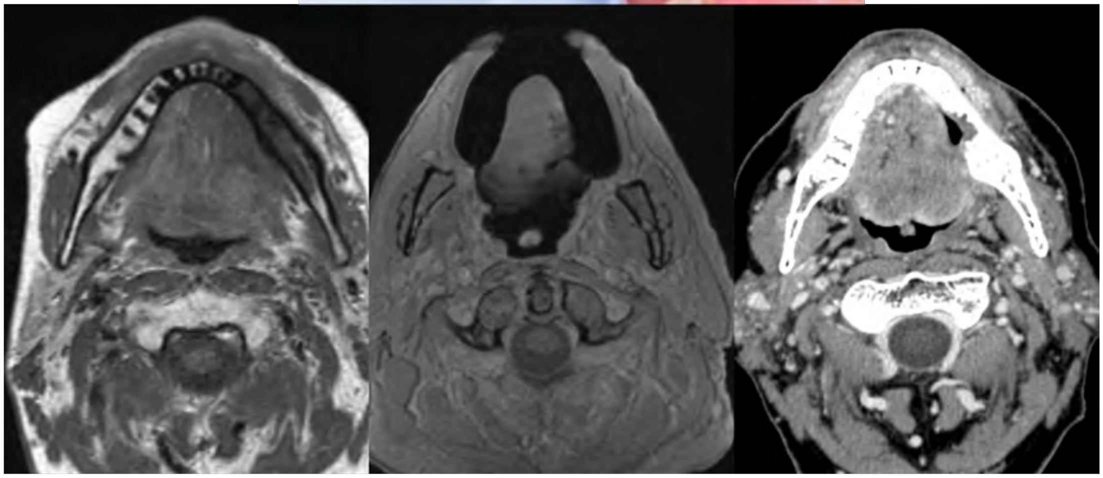

	Stage Assignment	Extent of Bone Involvement	Level of Co<br>1 = low ... 5<br>1	 <br>2	 <br>3
Case Classification			○	○	○

What radiographic features do you observe in Case 7? Type **NA** if no abnormalities are seen.

Case 8

Early ORN	Intermediate ORN
48%	36%

medRxiv preprint doi: https://doi.org/10.1101/2024.04.07.24305400; this version posted April 9, 2024. The copyright holder for this preprint (which was not certified by peer review) is the author/funder, who has granted medRxiv a license to display the preprint in perpetuity.
It is made available under a CC-BY 4.0 International license .

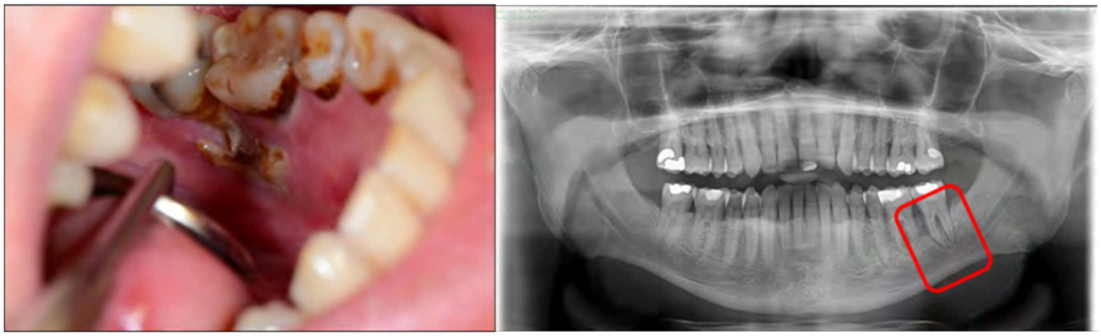

	Case Assignment	Level of Confidence 1 = low ... 5 = high				
		1	2	3	4	5
Case Classification		○	○	○	○	○

What radiographic features do you observe in Case 8? Type **NA** if no abnormalities are seen.

Case 9

Intermediate ORN	Advanced ORN
44%	40%

Clinical exam:

Exposed bone

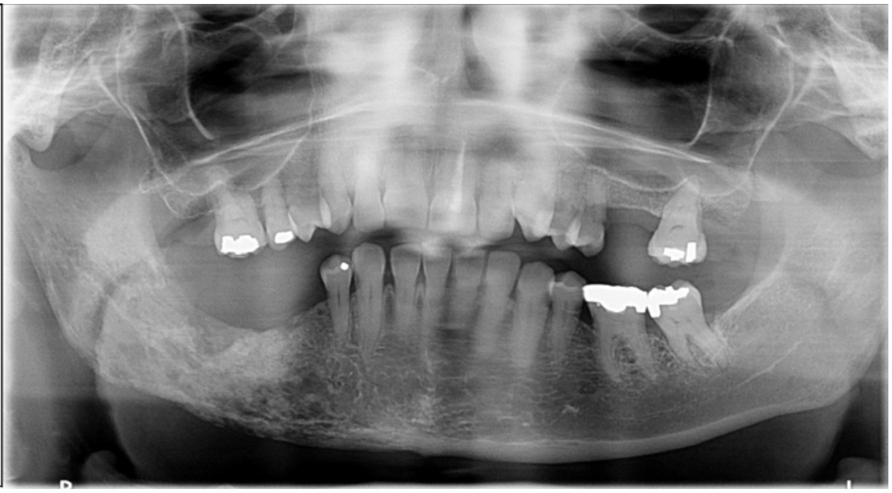

medRxiv preprint doi: https://doi.org/10.1101/2024.04.07.24305400; this version posted April 9, 2024. The copyright holder for this preprint (which was not certified by peer review) is the author/funder, who has granted medRxiv a license to display the preprint in perpetuity.
It is made available under a CC-BY 4.0 International license .

	Case Assignment	Level of Confidence 1 = low ... 5 = high				
		1	2	3	4	5
Case Classification		○	○	○	○	○

What radiographic features do you observe in Case 9? Type **NA** if no abnormalities are seen.

Case 10

Early ORN	Intermediate ORN
23%	59%

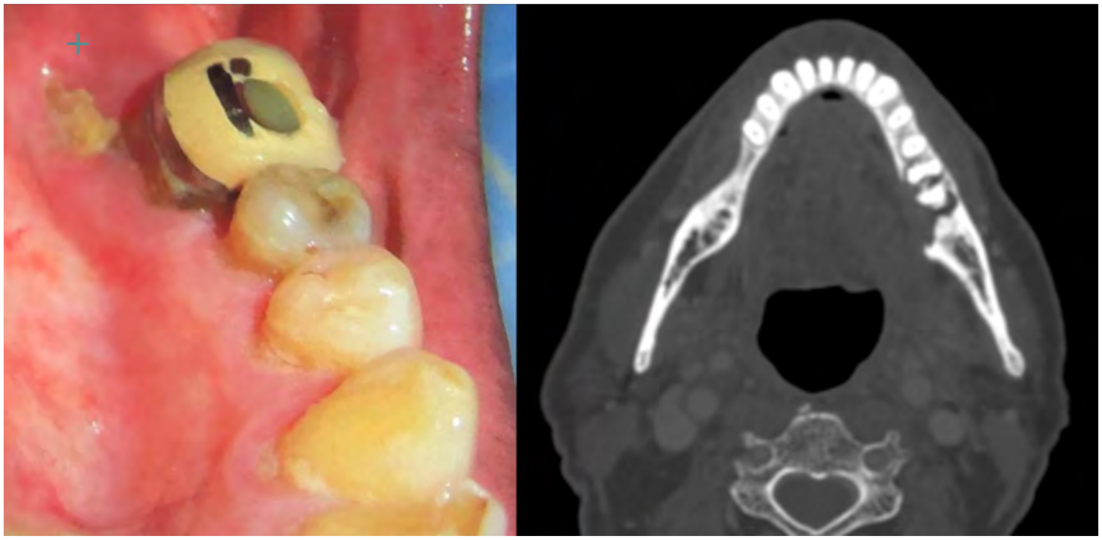

medRxiv preprint doi: https://doi.org/10.1101/2024.04.07.24305400; this version posted April 9, 2024. The copyright holder for this preprint (which was not certified by peer review) is the author/funder, who has granted medRxiv a license to display the preprint in perpetuity.
It is made available under a CC-BY 4.0 International license .

	Case Assignment	Extent of Bone Involvement	Level of Confidence 1 = low ... 5 = high 1	2	3	4
Case Classification			○	○	○	○

What radiographic features do you observe in Case 10? Type **NA** if no abnormalities are seen.

New Case 12

Imaging: sclerotic changes **within the alveolar bone**

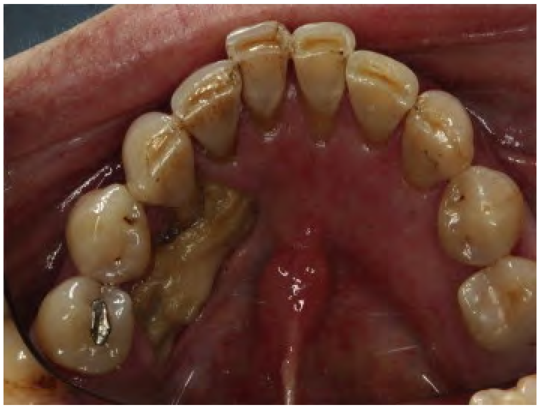

○ Early ORN
○ Intermediate ORN

New Case 13

Imaging: Lytic changes **beyond the alveolar bone**

medRxiv preprint doi: https://doi.org/10.1101/2024.04.07.24305400; this version posted April 9, 2024. The copyright holder for this preprint (which was not certified by peer review) is the author/funder, who has granted medRxiv a license to display the preprint in perpetuity.
It is made available under a CC-BY 4.0 International license .

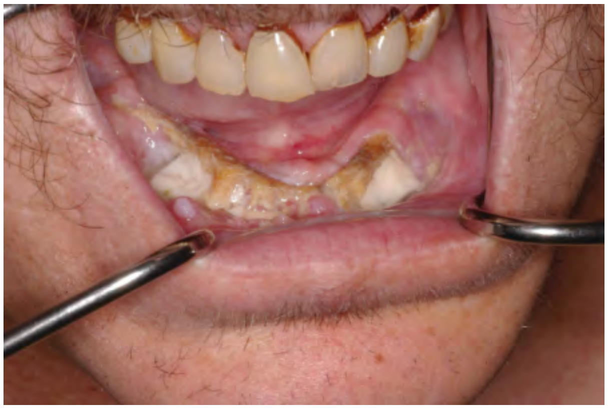

○ Intermediate ORN
○ Advanced

Did you find the educational resources on panoramic x-ray dental changes informative for your assessment of cases with dental imaging?

○ Yes
○ No
○ I did not open the links

Would you be interested in an educational resource with more detailed multidimensional (i.e., CT, MRI, panoramic xray) imaging features explained to demonstrate the full spectrum of ORN and its precursor stages?

○ Yes
○ No

Any additional commentary in preparation of our manuscript?

medRxiv preprint doi: https://doi.org/10.1101/2024.04.07.24305400; this version posted April 9, 2024. The copyright holder for this preprint (which was not certified by peer review) is the author/funder, who has granted medRxiv a license to display the preprint in perpetuity.
It is made available under a CC-BY 4.0 International license .

Powered by Qualtrics

medRxiv preprint doi: https://doi.org/10.1101/2024.04.07.24305400; this version posted April 9, 2024. The copyright holder for this preprint (which was not certified by peer review) is the author/funder, who has granted medRxiv a license to display the preprint in perpetuity.
It is made available under a CC-BY 4.0 International license .

S3_Methods

The process flow chart for this study is shown in Figure 1. A group of international multidisciplinary oncology and dental specialists were sent an electronic invitation to participate in the study (n=75). Members of the ORAL Consortium participated in at least one Delphi survey (n=69). ACM and CDF served as study coordinators and participants in the ORAL Consortium with no extra oversight voting rights throughout the study. No financial incentives were provided to participants. Surveys were developed in REDCap® and Qualtrics (see Supplementary Materials for all questionnaires) and sent to experts using personalized links to preserve annonymity.[1,2] Survey instruments were piloted by the study coordinator (ACM) to ensure correct formatting and delivery. Every questionnaire was in English and included an introduction, primary objectives for the round, and aggregated group feedback for consensus building. After each round, items meeting consensus were reported in following rounds as "consensus reports" and no further questions were asked on those items. Study preparation began in January 2023 with round 1 launching in March 2023, round 2 in May 2023, round 3 in August-September 2023, round 4 in February 2024, and manuscript review in March 2024 with final approval completed in April 2024. No deviations to study protocol were performed (MDA PA 2020-1096).

A comprehensive literature review was performed as preparatory research. Specifically, a PubMed search was performed in February 2023 for "(osteoradionecrosis) AND (staging OR grading)" which identified 447 publications since 1973 to 2023. Titles were screened for relevance resulting in abstract screening of 193 articles. Further screening was performed on full manuscript of original articles or reviews on one or more staging/grading systems for ORN/ORNJ. This resulted in the formal review and rating of 15 grading/staging systems by the ORAL Consortium. Round 1 included questions on definition preference, data element consideration within a formal ORNJ definition, level of agreement with each system, user preference, and rated effectiveness of systems for classifying ORNJ. Based on features identified in published systems, experts were also asked to classify the following three cases using 14 published staging systems: 1) *a patient with exposed bone (no measurement) not involving lower mandible, unknown duration. Pain present*, 2) a *patient with 1.2cm exposed bone for 4 months,*

medRxiv preprint doi: https://doi.org/10.1101/2024.04.07.24305400; this version posted April 9, 2024. The copyright holder for this preprint
(which was not certified by peer review) is the author/funder, who has granted medRxiv a license to display the preprint in perpetuity.
It is made available under a CC-BY 4.0 International license .

pain present, and 3) *a patient with 3cm exposed bone with pathologic fracture. Unknown symptoms or duration*. Imaging correlates of ORNJ were also introduced, and experts rated the level of importance of individual data elements (i.e., ulceration, exposed bone, pathologic fracture) on a 3-point ordinal scale matrix (i.e., not important, somewhat important, very important).

The second questionnaire provided anonymized group feedback on existing ORNJ definition rankings and asked experts whether the Consortium's definition for ORNJ should be in alignment with existing hierarchical international terminologies such as the Medical Dictionary of Regulatory Activities (MedDRA),[3] the Systematized Nomenclature of Medicine-Clinical Terms (SNOMED-CT),[4] and the International Classification of Disease (ICD),[5] the latter which had diagnostic codes for osteonecrosis as a general condition, but not ORNJ at the time of the study (ICD-10). Group feedback was both quantitative (i.e., ranked items) and qualitative (i.e., thematic grouping of commentary). Further clarification on the importance of a time feature for diagnosing ORNJ was asked, and experts reviewed and selected the best of four proposed ORNJ definitions. Open-ended questions were included for experts to provide commentary. Additional scenarios describing explicit combinations of clinical exam and imaging findings were presented for more granular stratification of *not related to ORNJ* versus *early/limited* and *advanced ORNJ* features. Of note, rounds 1 and 2 also included questions related to the parallel development of an information tool for visual representation of received radiation doses onto an odontogram (results not reported in this manuscript).

The third questionnaire presented several consensus statements to the Consortium related to what definitional concepts and criteria should and should not be considered mandatory for diagnosing ORNJ, and how implement the Common Terminology Criteria for Adverse Events (CTCAE) grading system when assessing patients for ORNJ.[6] Case review summaries were provided, and features rated as *early/limited ORNJ* were refined on a spectrum of *not related to ORNJ*, *precursor/stage 0*, and *early ORNJ* while *advanced ORNJ* features were reclassified as *intermediate ORNJ* or *advanced ORNJ*. Ten new cases with 2-dimensional (i.e., orthopantogram or OPG) or 3D (i.e., computed tomography [CT], magnetic resonance imaging [MRI]) diagnostic imaging snapshots were included for 1) stage

medRxiv preprint doi: https://doi.org/10.1101/2024.04.07.24305400; this version posted April 9, 2024. The copyright holder for this preprint (which was not certified by peer review) is the author/funder, who has granted medRxiv a license to display the preprint in perpetuity.
It is made available under a CC-BY 4.0 International license .

assignment, 2) reporting extent of bone involvement as seen on imaging (no abnormality, limited to the alveolar bone [AB], or beyond the AB), and 3) expert-rated level of confidence (LOC) on a 5-point scale (1=low, 5=high). Given recent publications of early MRI-based imaging biomarkers for vascular damage and potential precursor stages of ORNJ,[7] experts also provided opinion on how to classify vascular damage findings in the presence or absence of additional clinical and imaging findings.

Items on the fourth survey focused on finalizing a consensus-derived definition of ORNJ and reclassifying all cases that did not meet consensus during round 3. Prior to reclassifying cases, the ClinRad model,[8] a recently published risk-based classification system for ORNJ endorsed by ASCO/IAOO/MASCC expert guidelines,[9] was presented as well as two educational resources on how to interpret abnormal radiographic imaging findings.[10,11] Two additional cases with 'extensive bone exposure' seen on a clinical photograph and reported imaging findings within (Case 12) or beyond (Case 13) the alveolar bone were also reviewed to investigate the impact of clinical photographs on staging. Lastly, using a yes/no question format, experts were asked if they 1) found the educational resources on dental imaging informative for case review, and 2) whether they would be interested in having an educational resource with more detailed multidimensional (i.e., CT, MRI, panoramic x-ray) imaging features explained to demonstrate the full spectrum of ORNJ and its related precursor stages.

Descriptive statistics were performed to summarize the International ORAL Consortium expert demographics, staging systems review, and case classification as a collective cohort and by grouped specialties. During the second and third rounds, high-yield topics not meeting a pre-defined consensus threshold of 70% were represented in round 4 with a lowered consensus threshold of 60%. Experts were informed of thresholds prior to initiating each survey. The Fleiss' kappa statistic[12] was used to assess inter-rater agreement between all experts (n>2) when classifying the ten image-based cases on a categorical scale which included *Not precursor/Not ORNJ, Precursor/Stage 0, Early ORNJ, Intermediate ORNJ, Advanced ORNJ*. Interpretation of the kappa value is as follows: <0, poor agreement; 0.0-0.20, slight agreement; 0.21-0.40, fair agreement; 0.41-0.60, moderate agreement; 0.61-0.80, substantial agreement; and 0.81-1.0, almost perfect agreement.[13] Only ACM had access to

medRxiv preprint doi: https://doi.org/10.1101/2024.04.07.24305400; this version posted April 9, 2024. The copyright holder for this preprint (which was not certified by peer review) is the author/funder, who has granted medRxiv a license to display the preprint in perpetuity.
It is made available under a CC-BY 4.0 International license .

individual panelist response data which was used to summarize group feedback for presentation to the panel on subsequent rounds. All calculations and graphs were performed using R statistical software (Version 2023.06.1+524, R Foundation for Statistical Computing, Vienna, Austria; packages hmisc, table1, irr) and Python/Visual Studio Code.[14,15]

medRxiv preprint doi: https://doi.org/10.1101/2024.04.07.24305400; this version posted April 9, 2024. The copyright holder for this preprint (which was not certified by peer review) is the author/funder, who has granted medRxiv a license to display the preprint in perpetuity.
It is made available under a CC-BY 4.0 International license .

References

1. REDCap. https://www.project-redcap.org/.
2. Qualtrics XM: The Leading Experience Management Software. https://www.qualtrics.com/.
3. MSSO Updates | MedDRA. https://www.meddra.org/.
4. SNOMED - Home | SNOMED International. https://www.snomed.org/.
5. New ICD-10-CM & ICD-10-PCS Codes | NC Medicaid. https://medicaid.ncdhhs.gov/blog/2023/01/31/new-icd-10-cm-icd-10-pcs-codes.
6. CTCAE Files. https://evs.nci.nih.gov/ftp1/CTCAE/About.html.
7. Mohamed, A. S. R. *et al.* Quantitative Dynamic Contrast-Enhanced MRI Identifies Radiation-Induced Vascular Damage in Patients With Advanced Osteoradionecrosis: Results of a Prospective Study. *Int J Radiat Oncol Biol Phys* **108**, 1319–1328 (2020).
8. Watson, E. E. *et al.* Development and Standardization of a Classification System for Osteoradionecrosis: Implementation of a Risk-Based Model. *medRxiv* 2023.09.12.23295454 (2023) doi:10.1101/2023.09.12.23295454.
9. Peterson, D. *et al.* Prevention and Management of Osteoradionecrosis in Patients with Head and Neck Cancer Treated with Radiation Therapy: ISOO-MASCC-ASCO Guideline. *Journal of Clinical Oncology* (2024).
10. Mallya, S. M. & Tetradis, S. Imaging of Radiation- and Medication-Related Osteonecrosis. *Radiol Clin North Am* **56**, 77–89 (2018).
11. Chan, K. C. *et al.* Mandibular changes on panoramic imaging after head and neck radiotherapy. *Oral Surg Oral Med Oral Pathol Oral Radiol* **121**, 666–672 (2016).
12. McHugh, M. L. Interrater reliability: the kappa statistic. *Biochem Med (Zagreb)* **22**, 276 (2012).
13. Table 2, Interpretation of Fleiss' kappa (κ) (from Landis and Koch 1977). (2012).
14. R: The R Project for Statistical Computing. https://www.r-project.org/.
15. Download Python | Python.org. https://www.python.org/downloads/.

medRxiv preprint doi: https://doi.org/10.1101/2024.04.07.24305400; this version posted April 9, 2024. The copyright holder for this preprint (which was not certified by peer review) is the author/funder, who has granted medRxiv a license to display the preprint in perpetuity.
It is made available under a CC-BY 4.0 International license .

S4_Results

Staging Data Element Extraction and Classification

During round 1, a data element tracker flowsheet was developed for reporting all clinical, radiographic, therapy, and treatment response data elements identified in all staging/grading systems (Figure S3). Experts were then asked to rate the importance of each feature (not important, somewhat important, very important). Figure S4 shows the distribution of expert responses with only 3 data elements being considered somewhat/very important by 100% of the group: pathologic fracture, extent of bone involvement, and exposed bone. Other clinical (or radiographic) features achieving >70% rating of importance included orocutaneous fistula (98%), [mucosal] ulceration (85%), sinus formation (82%) and sequestra (81%). All remaining clinical/radiographic features underwent a first pass classification by experts into the following categories- *Not needed for ORNJ staging, Early/Limited ORNJ,* and *Advanced ORNJ* (Figure S5)*.* Five features met high consensus (81-97%) while 2 were equivocal (non-exposed bone/imaging findings beyond the AB; sequestra) and 4 were considered unnecessary for ORNJ staging including bone spicules. All these initially classified features, in addition to dental-based clinical scenarios with probe-to-bone [PTB] assessments (Figure S6), were reclassified in round 3 into tiered groups as follows: Group 1 [*Not a precursor/Not related to ORNJ; Precursor/Stage 0; Early ORNJ*], Group 2 [*Early ORNJ; Intermediate ORNJ*], and Group 3 [*Intermediate ORNJ; Advanced ORNJ*]. Items or cases not meeting the consensus threshold during round 3 were repeated in round 4. Additionally, 10 imaged-based cases (i.e., clinical photographs and/or 2D/3D radiographic images) were reviewed during the last two surveys to reinforce classification of MDE combinations (Figure 4).

<u>Not a Precursor / Not related to ORNJ</u>

- *PTB test <u>negative</u> with periosteal reaction <u>within</u> AB seen on imaging:* A comparable division in classifying this feature was noted during round 3 (47% *Not a precursor*) and round 4 (50% *Not a precursor*). RadOncs were the drivers of classifying this feature as a precursor stage compared to surgeons and dentists who favored it as being unrelated. The Consortium recommends this

medRxiv preprint doi: https://doi.org/10.1101/2024.04.07.24305400; this version posted April 9, 2024. The copyright holder for this preprint (which was not certified by peer review) is the author/funder, who has granted medRxiv a license to display the preprint in perpetuity.
It is made available under a CC-BY 4.0 International license .

feature combination to be considered as unrelated to ORNJ to differentiate it from consensus-approved precursor features. Of more importance, 2 MDEs are valuable in this description and include **clinical:PTB_test_result** (positive or negative) and **imaging:morphology** (i.e., periosteal reaction, sclerosis, lysis). The PTB test is a commonly utilized dental probing exam that provides diagnostic information on periodontal health;[1,2] therefore the Consortium recommends standardized documentation of this procedure whenever performed. Moreover, these MDEs are required for other upstaging feature combinations.

Precursor / Stage 0

- *Intact mucosa (no clinical bone exposure) with any imaging findings within AB*: This text-only description met high consensus during round 4 with 85% (45/53) of experts favoring a precursor assignment over being unrelated to ORNJ. Final round classification of image-based Case 3 further supported this designation with consensus being met at 67% (33/49). Identified MDEs are **clinical:mucosal_status** (i.e., intact, ulcerated) and the dichotomous **imaging:vertical_ab_abnormality** (i.e., within/above AB, beyond/below AB).
- *Minor bone spicules (MBS)*: This feature posed a significant challenge for classification across all rounds using text-only or image-based (Case 5) descriptions. When asked to classify MBS (text-only) during round 3, there was a slight preference for precursor stage (45%, favored by surgeons) over being unrelated to ORNJ (43%, favored by dentists; RadOncs equivocal) or representing early ORNJ. In round 4 (when *Early ORNJ* was removed as an option), the precursor status was again favored at 55%. However, when presented with clinical photography indicative of MBS, there was consistent upstaging of MBS to *Early ORNJ* in both rounds (49% and 50% *Early ORNJ*) with less than 30% assignment to precursor or unrelated stages. To mitigate classification ambiguity when clinically exposed bone is detected, serial clinical photographs and quantitative measurements of exposed bone (MDE **clinical:exposed_bone_length_in_mm**) are strongly recommended. An additional MDE for

medRxiv preprint doi: https://doi.org/10.1101/2024.04.07.24305400; this version posted April 9, 2024. The copyright holder for this preprint (which was not certified by peer review) is the author/funder, who has granted medRxiv a license to display the preprint in perpetuity.
It is made available under a CC-BY 4.0 International license .

minor_bone_spicules is proposed to explicitly report MBS which should be accompanied by a quantitative measurement.

- *PTB test negative with any imaging findings (EXCEPT periosteal reaction) within AB seen on imaging*: The Consortium achieved consensus during the final round for this feature combination (76%, 41/54). Associated MDEs are **clinical:PTB_test_result** (negative), **imaging:morphology,** and **imaging:vertical_ab_abnormality** (within AB)**.**
- *Vascular damage in bone seen on MRI without exposed bone and without other imaging findings (i.e., CT shows no bony abnormalities):* This scenario is considered a precursor stage as it met consensus during the final round as a text-only description (60%, 32/53) and in Case 7 which depicted minor mucosal ulceration (i.e., no bone exposure) with reported dynamic contrast-enhanced (DCE) MRI changes (63% agree, 31/49).
- *Vascular damage in bone seen on MRI without exposed bone and with other imaging findings limited to AB (i.e., x-ray shows sclerosis limited to AB):* The Consortium demonstrated consensus convergence with classifying this combination as a precursor stage (78%) over early ORNJ. This is also in alignment with classification of cases of intact mucosa with abnormal imaging findings restricted within the alveolar bone.

Early Stage ORNJ

- *PTB test positive with imaging findings within AB seen on imaging:* Compared to intact mucosa classification, a positive PTB test was considered to be an upstaging feature by the Consortium when combined with localized periosteal reactions (91%, 49/54) or other morphological radiographic changes (89%, 48/54) limited to the alveolar bone. This classification was supported by Case 4 which showed a photograph of a positive PTB test and OPG changes within the AB (86%, 43/50, staged it as *Early ORNJ*). Associated MDEs are **clinical:PTB_test_result** (positive), **imaging:morphology,** and **imaging:vertical_ab_abnormality** (within AB)**.**

medRxiv preprint doi: https://doi.org/10.1101/2024.04.07.24305400; this version posted April 9, 2024. The copyright holder for this preprint
(which was not certified by peer review) is the author/funder, who has granted medRxiv a license to display the preprint in perpetuity.
It is made available under a CC-BY 4.0 International license .

- *Exposed bone with any imaging findings within AB*: During round 3, this text-only presentation was unanimously classified as an *Early ORNJ* when limited to options ranging within Group 1. When asked to classify Case 6, a photograph with clinical bone exposure spanning the width of one molar and an OPG image showing periodontal ligament space widening, experts converged to consensus in assigning this feature combination as *Early ORNJ* (round 3, 55%; round 4, 74%). While these examples are in alignment with the ClinRad classification model for ORNJ,[3] it became evident that the extent of visualized bone exposure influences stage designation. For example, when presented with another case with similar imaging findings but extended clinical bone exposure spanning the length of two molars (Case 8), there was reduced consensus at classifying it as *Early ORNJ* (64%) over *Intermediate ORNJ* (36%). Case 12 showing clinical bone exposure beyond two teeth with text-only 'reported imaging findings within AB', was presented once at the end of round 4 after review of the ClinRad model. The clinical photograph influenced the upstaging of this case of extensive bone exposure to *Intermediate ORNJ* (76%, 39/51). To reduce mis-classification risks for the same or nearly identical patients, the Consortium again strongly recommends the use of a quantitative MDE (i.e., **clinical:exposed_bone_length_in_mm**) for facilitated data harmonization across disciplines and evolving staging systems. Using predefined cutoffs (i.e., </> 2cm) is discouraged as they limit future analysis of stage subclassification or reclassification as new knowledge on ORNJ emerges. A separate quantitative measurement (clinical:exposed_bone_width_in_mm) can be considered to capture 2-dimensional data on extent of bony exposure as needed for the area classification in the modified Shaw system.[4] Additional MDEs for this presentation include **clinical:mucosal_status** (absent), **imaging:morphology, imaging:vertical_ab_abnormality** (beyond AB).

medRxiv preprint doi: https://doi.org/10.1101/2024.04.07.24305400; this version posted April 9, 2024. The copyright holder for this preprint
(which was not certified by peer review) is the author/funder, who has granted medRxiv a license to display the preprint in perpetuity.
It is made available under a CC-BY 4.0 International license .

Intermediate Stage ORNJ

- *Exposed bone with any imaging findings beyond AB:* This feature combination was challenging to classify. When prompted with a text description in round 3, experts were split (44% intermediate, 40% advanced). A significant association between specialty group and classification was observed with most dental specialists considering it intermediate stage (69%) while others (72% RadOnc, 57% surgery) favored upstaging the feature combination (*P*=0.04). The repeated question in round 4, limited to these two stage options, resulted in convergence towards classification as intermediate stage (63%, 34/54, consensus threshold met). Case 9, a clinical report of exposed bone with OPG images showing full thickness sclerosis of the right mandible, and Case 10, a photograph of clinical bone exposure extending less than a molar width with axial CT image showing cortical bone changes, were presented as variations of this combination, with convergence towards classifying both also as *Intermediate ORNJ* during the final round (Case 9, 58%; Case 10, 86%). However, the upstaging effects of visualizing more extensive clinical bone exposure was reproducible using Case 13 which was staged as *Advanced ORNJ* by 71% (36/51) of experts over *Intermediate ORNJ* (29%). A more accurate summarization of such scenarios is through the use of standardized MDEs such as **clinical:exposed_bone_length_in_mm**, **clinical:mucosal_status** (absent)**,** **imaging:morphology,** and **imaging:vertical_ab_abnormality** (beyond AB)**.**

Advanced Stage ORNJ

The Consortium exhibited high agreement in classifying pathologic fracture (96%, 49/51), orocutaneous fistula (92%, 47/51), and oro-antral or oro-nasal fistula (86%, 43/51) as advanced features of ORNJ. These results are in alignment with advanced features reported in the ClinRad model and other staging systems, and their presence on clinical examination should be explicitly reported in a standardized fashion such as a **disorder_present** MDE with unique identifiers for each disorder (Table 2).

medRxiv preprint doi: https://doi.org/10.1101/2024.04.07.24305400; this version posted April 9, 2024. The copyright holder for this preprint (which was not certified by peer review) is the author/funder, who has granted medRxiv a license to display the preprint in perpetuity. It is made available under a CC-BY 4.0 International license .

Specialty-Specific Knowledge Siloes & Inter-Rater Reliability

Within the ORAL Consortium, a significant difference was found in the utilization of OPGs with experts from Oral Medicine/Oncology and OMFS (i.e., Oral/Dental) using them twice as often as oncologists (84% v 43%, *P=0.008*; Table S1). CT scans were commonly used by dental and oncology groups (75% v 90%; *P*=0.166) whereas MRI was used more frequently by oncologists than oral specialists (38% v 21%; *P*=0.334). When questioned on the effectiveness of each modality for diagnosing ORNJ (Table S2), CT scans were rated the most effective by more than 86% of the Consortium while approximately one-third of both specialty-condensed groups were neutral on the effectiveness of OPGs for ORNJ surveillance. While the least adopted modality, MRI had comparable effectiveness ratings to OPG, with experts favoring the following sequences: T1-weighted with contrast (36%), T2-weighted (29%), and DCE (22%).

The potential impact of familiarity with various imaging modalities on level of confidence in diagnosing imaged-based cases is seen in Figure 4. Out of 9 scenarios presented in round 3 with a radiographic image included (excluding case 5-clinical MBS), dental specialists had the highest LOC for all OPG-based cases but exhibited lowered LOC interquartile ranges comparable to RadOnc and surgery for Case 7 (MRI and CT) and Case 10 (CT). Cases with limited OPG-based abnormalities within the alveolar bone (i.e., periodontal ligament space widening; Cases 3, 4, 6, 8) evoked lower LOC among oncologists in diagnostic capabilities whereas radical changes seen on OPG such as a pathologic fracture (Case 2) were consistently classified the same for stage and extent of bone involvement by all specialists with a reported high LOC.

During round 3, the inter-rater reliability (IRR) for staging image-based cases (Table S3) varied per specialty with slight agreement among RadOnc (IRR 0.13, n=22) and fair or better agreement between surgeons (IRR 0.29, n=6) and dental specialists (IRR 0.34, n=14). Based on likelihood of categorizing the same cases per specific category, all experts showed the highest specialty group-level agreement for categorizing cases of *Advanced ORNJ* (RadOnc IRR 0.43; Surgery IRR 0.57;

medRxiv preprint doi: https://doi.org/10.1101/2024.04.07.24305400; this version posted April 9, 2024. The copyright holder for this preprint (which was not certified by peer review) is the author/funder, who has granted medRxiv a license to display the preprint in perpetuity.
It is made available under a CC-BY 4.0 International license .

Oral/Dental IRR 0.67) whereas the least agreement, if any, was around what cases should be considered unrelated to ORNJ (RadOnc IRR 0.1; Surgery IRR -0.07; Oral/Dental IRR 0.15), a precursor stage (Oral/Dental IRR 0.27), or an intermediate stage (RadOnc IRR 0.17; Surgery IRR 0.17). After providing group feedback from round 3, an introduction to the ClinRad risk-based model, and educational imaging resources for how to interpret OPG images, the overall specialty-level IRR during the last round improved for all groups (Oral/Dental IRR 0.38, n=18; RadOnc IRR 0.39, n=22; Surgery IRR 0.58, n=5). Moderate to substantial agreement was achieved for classifying cases as *Precursor/ Stage 0, Early ORNJ,* and *Intermediate ORNJ* but at a cost of decreasing agreement on non-fistula and non-pathologic fracture cases of *Advanced ORNJ*.

medRxiv preprint doi: https://doi.org/10.1101/2024.04.07.24305400; this version posted April 9, 2024. The copyright holder for this preprint
(which was not certified by peer review) is the author/funder, who has granted medRxiv a license to display the preprint in perpetuity.
It is made available under a CC-BY 4.0 International license .

REFERENCES

1. Listgarten, M. A. Periodontal probing: what does it mean? *J Clin Periodontol* **7**, 165–176 (1980).

2. Hefti, A. F. Periodontal probing. *Crit Rev Oral Biol Med* **8**, 336–356 (1997).

3. Watson, E. E. *et al.* Development and Standardization of a Classification System for Osteoradionecrosis: Implementation of a Risk-Based Model. *medRxiv* 2023.09.12.23295454 (2023) doi:10.1101/2023.09.12.23295454.

4. Shaw, R., Tesfaye, B., Bickerstaff, M., Silcocks, P. & Butterworth, C. Refining the definition of mandibular osteoradionecrosis in clinical trials: The cancer research UK HOPON trial (Hyperbaric Oxygen for the Prevention of Osteoradionecrosis). *Oral Oncol* **64**, 73–77 (2017).

medRxiv preprint doi: https://doi.org/10.1101/2024.04.07.24305400; this version posted April 9, 2024. The copyright holder for this preprint (which was not certified by peer review) is the author/funder, who has granted medRxiv a license to display the preprint in perpetuity.
It is made available under a CC-BY 4.0 International license .

S5_Supplemental Tables

Table S1. Use of Imaging Modalities per Condensed Specialties

	Oncology (N=40)	Oral/OMFS (N=19)	P-value
**Use of CT for ORNJ eval in past 1 year**			
Yes	30 (75.0%)	17 (89.5%)	0.166
No	10 (25.0%)	1 (5.3%)	
Missing	0 (0%)	1 (5.3%)	
**Use of MRI for ORNJ eval in past 1 year**			
Yes	15 (37.5%)	4 (21.1%)	0.334
No	25 (62.5%)	15 (78.9%)	
**Use of OPG for ORNJ eval in past 1 year**			
Yes	17 (42.5%)	16 (84.2%)	0.008
No	22 (55.0%)	3 (15.8%)	
Missing	1 (2.5%)	0 (0%)	

CT, Computed Tomography; MRI, Magnetic Resonance Imaging; OMFS, Oral and Maxillofacial Surgery;OPG, Orthopantomogram; ORNJ, Osteoradionecrosis of the Jaw

Table S2. Rated Effectiveness of Each Imaging Modality for ORNJ Surveillance

	Oncology (N=40)	Oral/OMFS (N=19)	Overall (N=59)
**Effectiveness of CT for ORNJ Surveillance**			
Very/somewhat effective	35 (87.5%)	16 (84.2%)	51 (86.4%)
Neutral	2 (5.0%)	2 (10.5%)	4 (6.8%)
Somewhat ineffective	1 (2.5%)	0 (0%)	1 (1.7%)
Missing	2 (5.0%)	1 (5.3%)	3 (5.1%)
**Effectiveness of MRI for ORNJ Surveillance**			
Very/somewhat effective	25 (62.5%)	8 (42.1%)	33 (55.9%)
Neutral	9 (22.5%)	7 (36.8%)	16 (27.1%)
Somewhat/very ineffective	3 (7.0%)	2 (10.5%)	5 (8.5%)
Missing	3 (7.5%)	2 (10.5%)	5 (8.5%)
**Effectiveness of OPG for ORNJ Surveillance**			
Very/somewhat effective	21 (62.5%)	10 (52.7%)	31 (52.6%)
Neutral	13 (32.5%)	6 (31.6%)	19 (32.2%)
Somewhat/very ineffective	3 (7.5%)	3 (15.8%)	6 10.2%)
Missing	3 (7.5%)	0 (0%)	3 (5.1%)

CT, Computed Tomography; MRI, Magnetic Resonance Imaging; OMFS, Oral and Maxillofacial Surgery;OPG, Orthopantomogram; ORNJ, Osteoradionecrosis of the Jaw

medRxiv preprint doi: https://doi.org/10.1101/2024.04.07.24305400; this version posted April 9, 2024. The copyright holder for this preprint (which was not certified by peer review) is the author/funder, who has granted medRxiv a license to display the preprint in perpetuity.
It is made available under a CC-BY 4.0 International license .

Table S3. Inter-Rater Reliability for Staging Image-Based Cases

	**Round 3**	**Round 4***
**ALL CASES**		
Oral/Dental	0.34	0.38
Rad Onc	0.13	0.39
Surgery	0.29	**0.58**
**PER CATEGORY**		
Not Precursor/ Not ORNJ		
Oral/Dental	0.15	0.2
Rad Onc	0.01	0.22
Surgery	-0.07	-0.02
Precursor/ Stage 0		
Oral/Dental	0.27	0.36
Rad Onc	0.1	**0.44**
Surgery	0.27	**0.62**
Early ORNJ		
Oral/Dental	0.28	0.39
Rad Onc	0.06	**0.44**
Surgery	0.31	**0.56**
Intermediate ORNJ		
Oral/Dental	0.32	**0.46**
Rad Onc	0.07	**0.42**
Surgery	0.17	**0.65**
Advanced ORNJ		
Oral/Dental	**0.67**	0.38
Rad Onc	**0.43**	0.4
Surgery	**0.57**	NA**

*Round 4 excludes Case 2 – pathologic fracture where there was high agreement during Round 3.
**No surgeon selected *Advanced ORN* as a stage category for all cases.
All values in **bold** reflect moderate or higher agreement (IRR $\geq$ 0.41).
Abbreviations; ORNJ, Osteoradionecrosis of the Jaw; Rad Onc, Radiation Oncology
